# Pain profile during orthodontic levelling and alignment with fixed appliances reported in randomized trials: a systematic review with meta-analyses

**DOI:** 10.1007/s00784-023-04931-5

**Published:** 2023-03-06

**Authors:** Deborah Susanne Inauen, Alexandra K. Papadopoulou, Theodore Eliades, Spyridon N. Papageorgiou

**Affiliations:** 1 Clinic of Orthodontics and Pediatric Dentistry, Center of Dental Medicine, University of Zurich, Plattenstr. 11, 8032 Zurich, Switzerland; 2 Division of Orthodontics, Faculty of Medicine, University Clinics of Dental Medicine, University of Geneva, Rue Michel-Servet 1, 1206 Geneva, Switzerland; 3 Discipline of Orthodontics and Paediatric Dentistry, Sydney Dental School, The University of Sydney, Sydney, Australia

**Keywords:** Orthodontic treatment, Pain, Discomfort, Visual analogue scale, Clinical trial, Systematic review

## Abstract

**Objective:**

To assess the pain profile of patients in the levelling/alignment phase of orthodontic treatment, as reported from randomized clinical trials.

**Materials and methods:**

Five databases were searched in September 2022 for randomized clinical trials assessing pain during levelling/alignment with a visual analogue scale (VAS). After duplicate study selection, data extraction, and risk-of-bias assessment, random effects meta-analyses of mean differences (MDs) and their 95% confidence intervals (CIs) were performed, followed by subgroup/meta-regression, and certainty analyses.

**Results:**

A total of 37 randomized trials including 2277 patients (40.3% male; mean age 17.5 years) were identified. Data indicated quick pain initiation after insertion of orthodontic appliances (*n* = 6; average = 12.4 mm VAS), a quick increase to a peak at day 1 (*n* = 29; average = 42.4 mm), and gradually daily decrease the first week until its end (*n* = 23; average = 9.0 mm). Every second patient reported analgesic use at least once this week (*n* = 8; 54.5%), with peak analgesic use at 6 h post-insertion (*n* = 2; 62.3%). Patients reported reduced pain in the evening compared to morning (*n* = 3; MD =  − 3.0 mm; 95%CI =  − 5.3, − 0.6; *P* = 0.01) and increased pain during chewing (*n* = 2; MD = 19.2 mm; 95% CI = 7.9, 30.4; *P* < 0.001) or occlusion of the back teeth (*n* = 2; MD = 12.4 mm; 95% CI = 1.4, 23.4; *P* = 0.3), while non-consistent effects were seen for patient age, sex, irregularity, or analgesic use. Subgroup analyses indicated increased pain among extraction cases and during treatment of the lower (rather than the upper) arch, while certainty around estimates was moderate to high.

**Conclusions:**

Evidence indicated a specific pain profile during orthodontic levelling/alignment, without signs of consistent patient-related influencing factors.

**Supplementary Information:**

The online version contains supplementary material available at 10.1007/s00784-023-04931-5.

## Introduction

### Rationale

The initial phase of comprehensive orthodontic treatment with fixed appliances almost always consists of the levelling/alignment of the dental arch and is dependent on the rapid and predictable response of the orthodontic appliance to the deformation of the orthodontic archwire. In order for the levelling/alignment phase to be considered efficient, the often prolonged duration of alignment [1] should be kept as low as possible, while minimizing treatment-induced adverse effects like apical root resorption [2, 3] and pain or discomfort (hereafter simply termed pain) [4, 5].

It is well documented [4, 6] that placement and activation of orthodontic appliances (brackets and wires) is associated with a pain response that is associated with both physical and psychological aspects [7]. This uncomfortable response might negatively influence patients’ willingness to initiate orthodontic treatment and their cooperation during treatment, treatment outcome quality or subsequent patient satisfaction, and the overall quality of life [8–12]. Several factors have been proposed to be associated with orthodontically induced pain, with varying robustness of underlying evidence, including among others the following: patient age, sex, previous pain experience, emotional or cognitive aspects, physical activity levels, baseline irregularity, and magnitude/timing of applied orthodontic force [13–18].

Previous systematic reviews on the subject have assessed pharmacological or non-pharmacological interventions or adjuncts to alleviate orthodontic pain [19–22], have compared different types of orthodontic appliances [23–25], or have focused on the impact of orthodontic pain on everyday life and overall quality of life [26]. However, to our knowledge, there is no critical appraisal of existing evidence on the expected pain profile for the average patient in terms of pain initiation, expected peak of pain response, duration of pain, and the average magnitude of pain at each timepoint. At the same time, it is important to have a benchmark about expected pain values in a future experimental clinical setting to be used when designing future trials and performing sample size calculations, as well as develop a core outcome set relevant to both orthodontists and patients in order to minimize the use of surrogate endpoints of little clinical relevance [27].

### Objectives

The aim of this systematic review was to critically assess the evidence derived from randomized clinical studies on the pain profile of human patients during the first levelling/alignment phase of fixed appliance orthodontic treatment.

## Materials and methods

### Registration and protocol

This review’s protocol was made a priori, registered in PROSPERO (CRD375515) with all post hoc changes having been transparently reported (Appendix 1). The conduct and reporting of this review is guided by the Cochrane Handbook [28] and the PRISMA statement [29], respectively. The focused question this review tried to answer is: “What is the pain profile of orthodontic patients during the initial levelling/alignment phase of fixed appliance treatment and which patient-related factors affect it?”.

### Eligibility criteria

Based on the Participants‐Intervention‐Comparison‐Outcome‐Study design (PICOS) schema, the included studies were randomized clinical trials (S) on human patients of any age, sex, ethnicity, or malocclusion (P) receiving comprehensive orthodontic treatment with fixed appliances on one or both jaws (I), for any randomized comparison with at least one trial arm of plain fixed appliances (C), without any limitations on language, publication year, or status. Included were any kinds of fixed appliances (conventionally- or self-ligated and labially- or lingually-placed), since little effects on the pain profile were expected [23, 24]. Excluded were non-clinical studies, animal studies, case reports/series, and non-randomized studies. Excluded were also within-person randomized studies (as carry-over effects were expected), studies with pharmacological interventions, and studies with surgical/non-surgical adjunct procedures/appliances not aimed at pain alleviation. The primary outcome (O) for this review was the patient-reported pain at the various timepoints of the levelling and alignment phase. Secondary outcomes included maximum pain, use of analgesics, and time to pain’s onset/decline.

### Search strategy

Five electronic databases were searched without restrictions from inception to October 1st, 2022 (Appendix 2), while open-access databases specifically covering gray literature (Directory of Open Access Journals, Digital Dissertations, metaRegister of Controlled Trials, WHO, Google Scholar), and the reference/citation lists of included articles or existing systematic reviews were manually searched.

### Selection process, data collection process, and risk of bias assessment

Two authors (DSI, AKP) screened the titles and/or abstracts of search hits to exclude obviously inappropriate studies, prior to checking their full texts. Any differences between the two reviewers were resolved by discussion with another author (TE).

Data from included studies was collected independently by two authors (DSI, AKP) with the same way to resolve discrepancies using pre‐defined/piloted forms covering the following: (a) study characteristics (design, clinical setting, country), (b) patient characteristics (age, sex, irregularity), (c) treatment details (jaw treated, incorporation of extractions, bracket slot size, wire used, performed comparisons), and (d) outcome details (type of outcome and time of measurement).

As the primary aim of this review was to quantify the average pain profile of patients at each timepoint, purely observational data were to be used from the included randomized studies and their comparisons were ignored. Therefore, the internal validity (with extension to the risk of bias) of these single-group study arms was assessed with a custom tool based on the Joanna Briggs Institute checklist for cohort studies (http://joannabriggs-webdev.org/research/critical-appraisal-tools.html), after checking with the editor of the Cochrane Handbook. All studies were appraised independently by three authors (DSI, AKP, SNP) with any differences being resolved by a third author (TE).

### Effect measures and synthesis methods

An effort was made to maximize data output from included studies by extracting or calculating missing data, whenever possible (Appendix 1). As the outcome of treatment-induced orthodontic pain was expected to be affected by patient-, treatment-, and measurement-related characteristics [14, 16, 30–32], a random-effects model was a priori deemed appropriate to calculate the average distribution of true effects, based on clinical and statistical reasoning [33], and a restricted maximum likelihood (REML) variance estimator with improved performance was used according to recent guidance [34].

The primary analysis was based on indirect meta-analyses from randomized trials calculating average pooled averages (for mean pain, maximum pain, and time) or frequencies (for analgesic use), using only the trial arm of plain fixed appliances (without adjuncts) from each trial (or combining multiple similar arms prior to pooling, if needed). Secondarily, direct comparisons were performed to identify the influence of several characteristics using mean differences (MDs) or odds ratios (ORs) and their corresponding 95% confidence intervals (CIs). The produced forest plots for direct comparisons were augmented with contours denoting the magnitude of the observed effects (Appendix 1) to assess heterogeneity, clinical relevance, and imprecision [35].

The extent and impact of between‐study heterogeneity was assessed by inspecting the forest plots and by calculating the τ^2^ (absolute heterogeneity) or the I^2^ statistics (relative heterogeneity). I^2^ defines the proportion of total variability in the result explained by heterogeneity, and not chance. For all heterogeneity metrics, the heterogeneity’s direction (localization on the forest plot) and uncertainty around heterogeneity estimates [36] was also considered, while 95% random-effects predictive intervals were used to incorporate observed heterogeneity and give a range of plausible effects [37].

Possible sources of heterogeneity were a priori planned to be sought through several mixed-effects subgroup and mixed‐effects meta‐regression analyses (both with the REML estimator) in meta‐analyses of at least five trials for patient age, sex, baseline irregularity, incorporation of extractions, bracket slot size, and treated jaw, while some planned factors were ultimately dropped (Appendix 1).

Robustness of the results was checked for meta-analyses ≥ 5 studies with sensitivity analyses based on (i) the inclusion of selected patients rather than broad inclusion criteria, (ii) the assessment of analgesic use rather than ignoring it, and (iii) studies with adequate versus inadequate samples, with the cut-off arbitrarily set at 40 patients/study. All analyses were run in R (version 4.0.4) by one author (SNP) and the dataset was openly provided [38]. All *P* values were two‐sided with *α* = 5%, except for the test of between‐studies or between‐subgroups heterogeneity where *α*‐value was set as 10% [39].

### Reporting bias assessment

Reporting biases (including small-study effects and the possibility of publication bias) were assessed with contour-enhanced funnel plots and Egger’s test for meta-analyses with ≥ 10 studies [29].

### Certainty assessment

The overall quality of evidence (i.e., the strength of clinical recommendations) from direct meta-analyses (MDs and ORs) was rated using the Grades of Recommendations, Assessment, Development and Evaluation (GRADE) approach [40] and an improved Summary Of Findings table format [41].

## Results

### Study selection

**Fig. 1:**
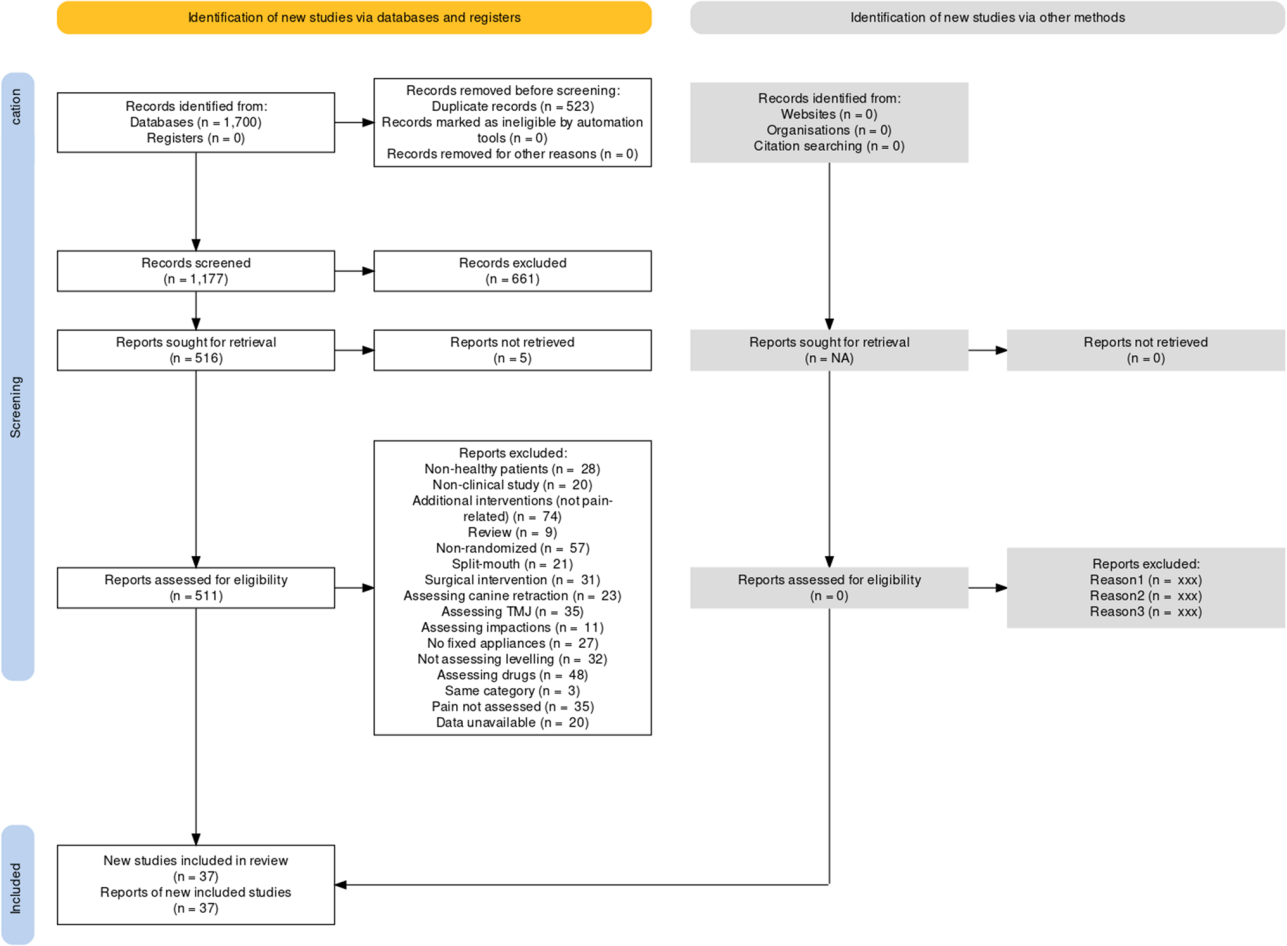
PRISMA 2020 flow diagram

A total of 1700 hits were retrieved by the literature search of 5 databases (Fig. 1). After removing duplicates and eliminating non-relevant reports by title/abstracts not relevant to orthodontics, 517 full-text papers were checked against the eligibility criteria (Appendix 3). In the end 37 publications (36 in journals and 1 as Master thesis) pertaining to 37 unique trials were included in this review.

### Study characteristics

These 37 included studies were all parallel-group randomized trials performed in university clinics (73%; 27/37), hospitals (15%; 4/27), private practices (8%; 3/37), or multiple centers (5%; 2/37) (Table 1). The included trials were conducted in 16 different countries (Australia, Brazil, China, Egypt, Great Britain, India, Iran, Italy, Norway, Poland, Saudi Arabia, Spain, Syria, Turkey, United Arab Emirates, and United States of America) and published as journal papers in English (95%; 35/37), journal papers in Portuguese (3%; 1/37), or Master thesis in Portuguese (3%; 1/37).

**Table 1:** Characteristics of included studies

Nr	Study	Setting	*n* (M/F); mAge	LII	Jaw	Ex	Slot	Wire	Comparisons	Pain
1	Al Shayea 2020	Uni; SA	60 (0/60); 23.8	NR	Both	No	NR	0.016″ NiTi	Wafer—chewing gum	VAS
2	Al-Okla 2020	Uni; AE	34 (9/25); 19.0	NR	Both	No	0.022”	0.016″ HA-NiTi	Vibration—placebo	VAS
3	AlSayed Hasan 2020	Uni; SY	26 (6/20); 20.1	9.9	Max	Yes	0.022”	0.014″ NiTi	Laser—placebo	VAS
4	Azizi 2021	Uni; IR	88 (44/44); 18.6	8.5	Mnd	No	0.022”	0.014″ SE/HA NiTi	SE NiTi—HA NiTi	VAS
5	Bartlett 2005	Uni; US	150 (69/81); 16.0	NR	Both	NR	NR	NR	Call	VAS
6	Brito 2022	Uni; BR	54 (24/30); 27.9	5.1	Both	No	0.022”	0.012″ HA NiTi	Laser	VAS
7	Casteluci 2021	Uni; BR	39 (25/14); 22.2	4.8	NR	No	0.022”	0.014″ & 0.016″ & 0.016 × 0.022″ NiTi	Aligners	VAS
8	Cioffi 2012	Uni; IT	30 (11/19); 14.7	1.5	Either	Yes	0.022”	0.016″ SE/HA NiTi	SE NiTi—HA NiTi	VAS
9	Cozzani 2016	Hosp; IT	84 (43/41); 13.3	NR	Max	No	NR	NR	Message—call	VAS
10	Curto 2020	Uni; ES	90 (35/55); 21.7	2.8	Both	No	0.022”	0.014″ SE NiTi	Low-friction bracket—lubricating gel	VAS
11	de Mendonça 2020	Uni; BR	103 (40/63); 20.6	NR	Max	NR	NR	0.012″ or 0.014″ NiTi	Message	VAS
12	Erdinc 2004	Uni; TR	109 (52/57); 14.2	NR	Both/Max	NR	0.018”	0.014″ or 0.016″ NiTi	0.014″ NiTi—0.016″ NiTi	VAS
13	Farzanegan 2012	Uni; IR	40 (0/40); NR	NR	Both	Yes	0.018”	0.016″ NiTi	Wafer—chewing gum—placebo	VAS
14	Fernandes 1998	Uni; NO	128 (56/72); 12.5	NR	Either	NR	NR	0.014″ NiTi/SE NiTi	NiTi—SE NiTi	VAS
15	Fleming 2009	Hosp; GB	48 (16/32); 16.0	16.5	Mnd	No	0.022″ SL/CB	0.016″ HA NiTi	SL—CB	VAS
16	Ghaffar 2022	Uni; EG	32 (0/32); NR	6.1	Mnd	No	0.022”	0.014″ & 0.016″ & 0.016 × 0.022″ HA NiTi	Laser	VAS
17	González-Sáez 2021	Uni; ES	90 (34/56); 21.7	3.7	Both	No	0.022″ SL/CB	0.014″ SE NiTi	Low-friction bracket—SL—CB	VAS
18	Huang 2016	Hosp; CN	54 (NR); 22.0	NR	NR	NR	NR	NR	Music—CT	VAS
19	Keith 2013	Uni; US	39 (14/25); 13.4	NR	Max	NR	NR	NR	Message	VAS
20	Kishore 2019	Uni; IN	14 (NR); NR	NR	Mnd	Yes	0.022”	0.016″ (HA) NiTi	NiTi—HA NiTi	VAS
21	Lo Giudice 2019	Uni; IT	84 (41/43); 16.5	5.9	Mnd	No	0.022″ SL	0.014″ HA NiTi	Laser—placebo	NRS
22	Matys 2020	Uni; PL	76 (21/55); 35.1	NR	Max	NR	0.018”	0.014″ NiTi	Laser	NRS
23	Miles 2010	Pract; AU	60 (22/38);13.5	7.1	Max	No	0.018″ SL/CB	0.014″ NiTi	Ceramic SL—ceramic CB	Likert
24	Miles 2012	Pract; AU	66 (26/40); 13.1	5.6	Mnd	No	0.018”	0.014″ HA NiTi	Vibration	VAS
25	Miles 2016	Pract; AU	40 (14/26); 12.9	4.2	Both	Yes	0.018”	0.014″ HA NiTi	Vibration	VAS
26	Montebugnoli 2020	Uni; IT	60 (26/34); 15.1	NR	Max	No	0.022”	0.012″ A-SS	Verbal/written information	NRS
27	Otasevic 2006	Hosp; GB	84 (37/47); 14.0	NR	Either	Mix	0.022”	0.016″ NiTi	Wafer—reduced mastication (placebo)	VAS
28	Pinheiro 2015	Uni; BR	30 (NR); NR	NR	NR	NR	0.018”	0.012″ HA NiTi	Laser—placebo	VAS
29	Pringle 2009	Hosp; GB	52 (24/28); 15.7	6.2	Both	Mix	0.022″ SL/CB	0.014″ HA NiTi	SL—CB	VAS
30	Sandhu 2013	Uni; IN	85 (42/43); 14.0	6.6	Mnd	Mix	0.022”	0.016″ SE NiTi/0.0175″ MS-SS	0.016″ SE NiTi—0.0175″ MS-SS	VAS
31	Scott 2008	Uni/Hosp; GB	62 (32/30); 16.3	11.8	Mnd	Yes	0.022″ SL/CB	0.014″ HA NiTi	SL—CB	VAS
32	Serritella 2021	Uni; IT	36 (14/22); 19.5	NR	Either	NR	NR	NR	Auriculotherapy	VAS
33	Sfondrini 2020	Uni; IT	26 (9/17); 11.8	NR	Max	NR	NR	NR	Laser—placebo	VAS
34	Silva-Santos 2019	Uni; BR	42 (18/24); 20.4	0.7	Max	No	0.022”	0.014″ NiTi	Chewing gum	VAS
35	Tortamano 2009	Uni; BR	60 (18/42); 15.9	NR	Either	Mix	NR	0.014″ SS	Laser—placebo	NRS
36	White 2017	Uni; US	41 (17/24); NR	NR	Mnd	No	0.018”	0.016″ HA NiTi & 0.017 × 0.028″ HA NiTi & 0.016 × 0.022″ SS	Aligners	VAS
37	Woodhouse 2015	Uni/Hosp; GB	81 (40/41); 13.9	8.4	Mnd	Yes	0.022”	0.014″ NiTi & 0.018″ NiTi	Vibration—placebo	VAS

*A-SS*, Australian stainless steel wire; *CB*, conventional brackets; *F*, female; *HA*, heat-activated; *LII*, Little’s Irregularity Index; *M*, male; *mAge*, mean age; *Max*, maxillary arch; *Mnd*, mandibular arch; *MS-SS*, multi-stranded stainless steel wire; *n*, number of patients; *NiTi*, nickel-titanium arch; *NR*, not reported; Pract, practice; *SE*, superelastic; *SL*, self-ligated brackets; *SS*, stainless steel wire; *Uni*, university clinic; *Hosp*, hospital

The included trials covered a total of 2277 patients, to a median sample size of 54 patients/trial (range 14 to 150 patients/trial). Among the 34 trials reporting the patients’ gender, 40.3% were male (879/2179), while from the 32 trials reporting age, the average across trials was 17.5 years (range of average age per trial 11.8 to 35.1 years). From the 18 trials reporting on it, average baseline irregularity across trials was 6.41 mm.

Among the included trials, 34 reported which jaw was treated and from them 29% (10/34) treated the mandible, 26% (9/34) the maxilla, 26% (9/34) both jaws, and the remaining 18% (6/34) combinations thereof. As far as premolar extractions are concerned, from trials reporting on extractions (27/37) the majority of trials (59%; 16/27) included non-extraction cases, 26% (7/27) included only extraction cases, and 15% (4/27) had a mix of extraction/non-extraction cases. Among trials reporting slot size (27/37), the majority used a 0.022-inch bracket (70%; 19/27) and the remaining 30% (8/27) used a 0.018-inch bracket, while 6 trials had at least one group with self-ligating brackets. From the 31/37 trials reporting on wires used, the majority of the included studies (55%; 17/31) used initially a 0.014-inch wire, a 0.0160-inch wire (26%; 8/31), a 0.012-inch wire (10%; 3/31) or other wires, with most of them being Nickel-Titanium (NiTi) wires.

Various interventions were assessed in the included randomized trials, including laser adjuncts (22%; 8/37), different brackets (16%; 6/37), different wires (16%; 6/37), patient management methods (16%; 6/37), vibrational adjuncts (11%; 4/37), occlusal relief measures (11%; 4/37), clear aligners (5%; 2/37), or alternative medicine methods (3%; 1/37). However, these comparisons fall not within the scope of the present review and only trial arms with simple fixed appliances without the use of any adjunct procedures/appliances were used for the analyses.

The vast majority of trials used a visual analogue scale (VAS) (86%; 32/37) for patient-reported pain (either on the 10-cm or 100-mm scale) or a similar numeric rating scale (11%; 4/37) and could be combined, after appropriate modifications, in meta-analyses. One trial used a Likert scale, but without specifying the actual values on it, and was therefore excluded from the analyses.

### Risk of bias in studies

The assessment of included trials in terms of internal validity/reporting completeness (with possible ties to risk of bias) was assessed using a customized tool for cohort studies (Table 2). Only about half of included trials (54%; 20/37) selected patients to include in the trial without any pain-related eligibility criteria, while the remaining used criteria at least partly related to pain response, which might limit the trial’s generalizability to the average patient. Reporting of important patient- or treatment-related characteristics was often suboptimal, with omissions being seen for patient age (14%; 5/37), patient sex (8%; 3/37), baseline irregularity (54%; 20/37), treated jaw (24%; 9/37), extractions (27%; 10/37), used wire (16%; 6/37), or used brackets (24%; 9/37). Only a very small minority of included trials (14%; 5/37) adequately assessed patient anxiety, which could exert an influence on orthodontic pain. Potential confounding by the use of analgesic medication was adequately covered in only 41% (15/37) of included trials, where analgesics were either prohibited or their use during alignment/levelling was completely reported. Complete description of orthodontic pain trajectory (judged as reporting pain for more than 3 days post-insertion) was done in half of the included trials (51%; 19/37). Finally, an adequate patient sample was included in 62% (23/37) of included trials.

**Table 2:** Assessment of internal validity/risk of bias of included trials

Nr	Study	Prospective	Selected patients	Age rep’d	Sex rep’d	Crowding rep’d	Jaw rep’d	Ex rep’d	Wire rep’d	Slot rep’d	Anxiety assessed	Analgesics prohibited	> 3 days rep’d	Adequate sample
1	Al Shayea 2020	Yes	No	Yes	Yes	No	Yes	Yes	Yes	Yes	No	Yes	Yes	Yes
2	Al-Okla 2020	Yes	No	Yes	Yes	No	Yes	Yes	Yes	Yes	No	Yes	No	No
3	AlSayed Hasan 2020	Yes	No	Yes	Yes	Yes	Yes	Yes	Yes	Yes	No	No	No	No
4	Azizi 2021	Yes	Partly	Yes	Yes	Yes	Yes	Yes	Yes	Yes	No	No, but reported	No	Yes
5	Bartlett 2005	Yes	Yes	Yes	Yes	No	Yes	No	No	No	Yes	No; partly reported	Yes	Yes
6	Brito 2022	Yes	Unclear	Yes	Yes	Yes	Yes	Yes	Yes	Yes	No	No	No	Yes
7	Casteluci 2021	Yes	Partly	Yes	Yes	Yes	No	Yes	Yes	Yes	Yes	No, but reported	Yes	No
8	Cioffi 2012	Yes	Partly	Yes	Yes	Yes	No	Yes	Yes	Yes	Partly	No, but reported	Yes	No
9	Cozzani 2016	Yes	No	Yes	Yes	No	Yes	Yes	No	No	No	No; partly reported	Yes	Yes
10	Curto 2020	Yes	No	Yes	Yes	Yes	Yes	Yes	Yes	Yes	No	No	Yes	Yes
11	de Mendonça 2020	Yes	Partly	Yes	Yes	No	Yes	No	Yes	No	Yes	No, but reported	Yes	Yes
12	Erdinc 2004	Yes	Unclear	Yes	Yes	No	No	No	Yes	Yes	No	No, but reported	Yes	Yes
13	Farzanegan 2012	Yes	Partly	No	Yes	No	Yes	Yes	Yes	Yes	No	Yes	Partly	No
14	Fernandes 1998	Yes	No	Yes	Yes	No	No	No	Yes	No	No	No, but reported	Yes	Yes
15	Fleming 2009	Yes	No	Yes	Yes	Yes	Yes	Yes	Yes	Yes	Yes	Yes	No	No
16	Ghaffar 2022	Yes	No	No	Yes	Yes	Yes	Yes	Yes	Yes	No	No	Yes	No
17	González-Sáez 2021	Yes	Partly	Yes	Yes	Yes	Yes	Yes	Yes	Yes	No	No	Yes	Yes
18	Huang 2016	Yes	Yes	Yes	No	No	No	No	No	No	No	No	Yes	Yes
19	Keith 2013	Yes	Partly	Yes	Yes	No	Yes	No	No	No	Partly	No; partly reported	Yes	No
20	Kishore 2019	Yes	Yes	No	No	No	Yes	Yes	Yes	Yes	No	No	No	No
21	Lo Giudice 2019	Yes	No	Yes	Yes	Yes	Yes	Yes	Yes	Yes	No	Unclear	Yes	Yes
22	Matys 2020	Yes	Partly	Yes	Yes	No	Yes	No	Yes	Yes	No	No	No	Yes
23	Miles 2010	Yes	No	Yes	Yes	Yes	Yes	Yes	Yes	Yes	No	No	Partly	Yes
24	Miles 2012	Yes	No	Yes	Yes	Yes	Yes	Yes	Yes	Yes	No	No	Partly	Yes
25	Miles 2016	Yes	No	Yes	Yes	Yes	Yes	Yes	Yes	Yes	No	No, but reported	Partly	Yes
26	Montebugnoli 2020	Yes	Partly	Yes	Yes	No	Yes	Yes	Yes	Yes	Yes	No, but reported	Yes	Yes
27	Otasevic 2006	Yes	Unclear	Yes	Yes	No	No	Yes	Yes	Yes	Partly	No; partly reported	No	Yes
28	Pinheiro 2015	Yes	No	No	No	No	No	No	Yes	Yes	No	No	Yes	No
29	Pringle 2009	Yes	No	Yes	Yes	Yes	Yes	Yes	Yes	Yes	No	No; partly reported	Yes	Yes
30	Sandhu 2013	Yes	Yes	Yes	Yes	Yes	Yes	Yes	Yes	Yes	No	No	Yes	Yes
31	Scott 2008	Yes	No	Yes	Yes	No	Yes	Yes	Yes	Yes	No	No, but reported	Partly	Yes
32	Serritella 2021	Yes	No	Yes	Yes	No	No	No	No	No	No	No	No	No
33	Sfondrini 2020	Yes	No	Yes	Yes	No	Yes	No	No	No	No	No	No	No
34	Silva-Santos 2019	Yes	No	Yes	Yes	Yes	Yes	Yes	Yes	Yes	No	Yes (excluded)	Partly	No
35	Tortamano 2009	Yes	Unclear	Yes	Yes	No	No	Yes	Yes	No	No	Yes	No	Yes
36	White 2017	Yes	No	No	Yes	No	Yes	Yes	Yes	Yes	No	No, but reported	Yes	No
37	Woodhouse 2015	Yes	No	Yes	Yes	Yes	Yes	Yes	Yes	Yes	No	No, but reported	Partly	Yes

*Ex*, extractions; *rep’d*, reported

### Results of individual studies

This review included aggregate data provided in published reports of included studies, except for four studies [42–45] where raw data were already available to the senior author and these were re-analyzed in Appendix 4a-d. Re-analysis of individual patient data with linear regressions failed to find a significant effect of patient age, patient sex, baseline mandibular irregularity, and incorporation of extractions in the treatment plan. The data from one trial however [43] found an effect of baseline maxillary irregularity on maximum pain intensity, with additional 1.47 mm in VAS for each additional irregularity mm (95% CI = 0.29 to 2.04 mm; *P* = 0.02). Additionally, data from the same trial found that patients who had taken analgesics reported higher maximum pain (+ 27.07 mm; 95% CI = 14.10 to 40.04 mm; *P* < 0.001) and pain at days 1–2 (+ 24.98 mm; 95% CI = 14.17 to 35.80 mm; *P* < 0.001) than patients that did not take.

The results of outcomes/comparisons reported by single studies that could not be incorporated in meta-analyses are given as indirect analyses of pooled averages/rates in Appendix 5 and as direct analyses of various comparisons in Appendix 6. One trial reported significantly higher pain for female patients compared to male patients 6 h post-insertion. Results from single trials also indicated that patients consuming analgesics reported significantly higher pain than non-consumers at 6 h, 2 days, and 8 days post-insertion. Some data indicated different pain reading regarding time of day (morning or afternoon or evening), but these were not consistent and of little clinical relevance. Additionally, one trial reported that patients reported higher pain at the anterior rather than the posterior teeth. Finally, data indicated that pain during chewing or biting was significantly higher than pain during occlusion of the posterior teeth, while pain during occlusion of the posterior teeth was significantly higher than spontaneous pain.

### Results of syntheses

**Fig. 2:**
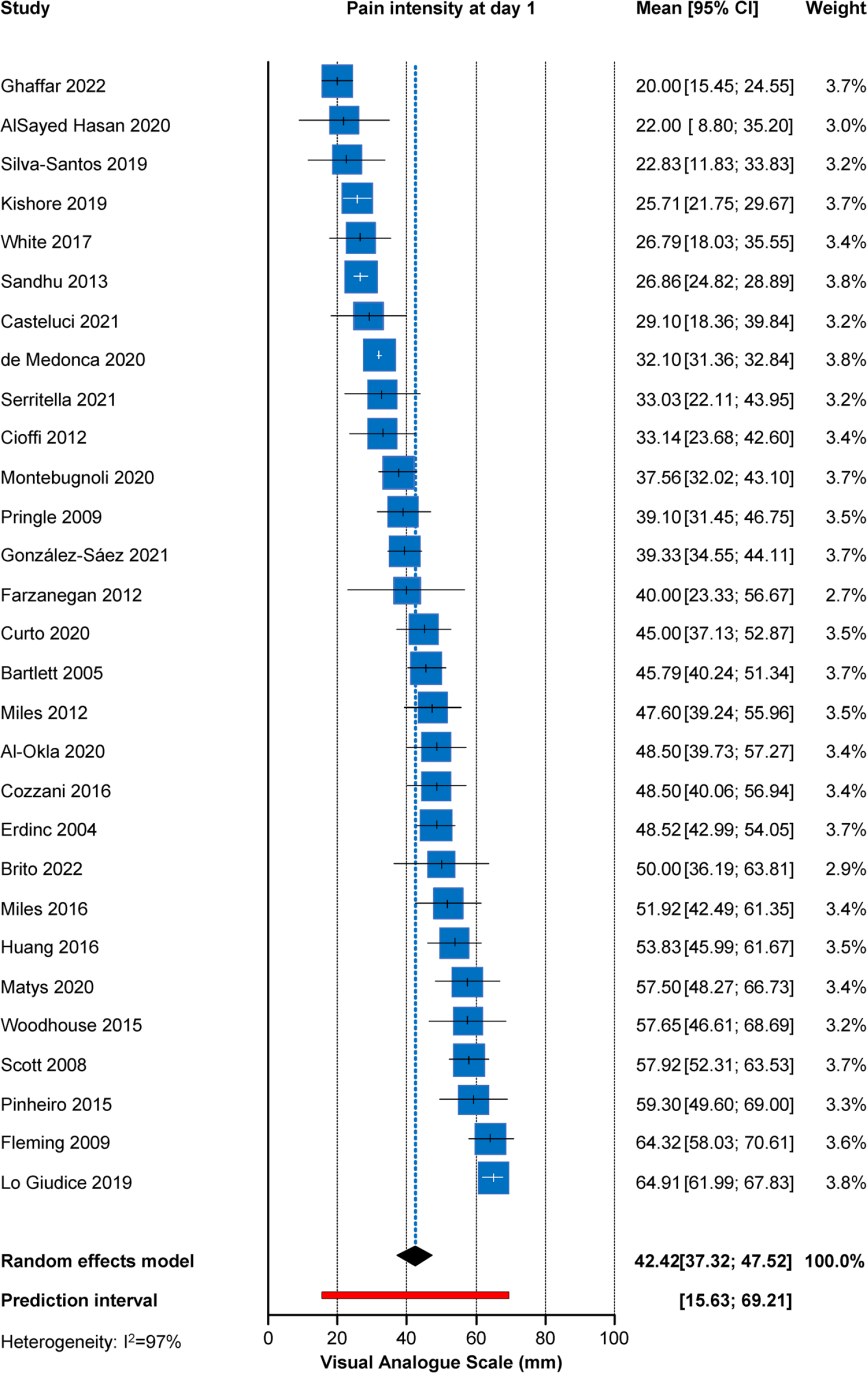
Forest plot for the indirect pooling of average patient-reported pain (in a 100-mm visual analogue scale) at day 1 after appliance insertion

Meta-analyses of the average pain profile during orthodontic levelling/alignment (indirect analysis) in mm of 100-mm VAS are given in Table 3. The average time to pain onset was calculated to be 4.1 h (2 trials; 95% CI = 0, 25.1 h). Results indicate that there was a steady increase in average pain felt in the first hours after insertion of the orthodontic appliances with a possible peak around the first day (29 trials; average = 42.4 mm; 95% CI = 37.3, 47.5 mm) (Fig. 2), and a gradual decline to day 2 (24 trials; average = 37.4 mm; 95% CI = 32.5, 42.4 mm), day 3 (30.2 trials; average = 30.2 mm; 95% CI = 26.1, 34.3 mm), day 4 (17 trials; average = 22.6 mm; 95% CI = 18.6, 26.7 mm), day 5 (16 trials; average = 16.1 mm; 95% CI = 12.4, 19.8 mm), day 6 (16 trials; average = 11.1 mm; 95% CI = 7.8, 14.4 mm), and day 7 (23 trials; average = 9.0 mm; 95% CI = 6.5, 11.6 mm). However, very high heterogeneity was seen for all indirect meta-analyses of Table 3, both in absolute terms (τ^2^) and in terms of inconsistency (I^2^), which makes interpretations based solely on point estimates (pooled average) or their 95% CIs questionable, possibly making the 95% predictions that incorporate this heterogeneity preferable. As such, the 95% predictions showed a similar profile of gradual decline from day 1 (15.6 to 69.2 mm), to day 2 (13.8 to 61.1 mm), day 3 (9.4 to 51.1 mm), day 4 (7.7 to 37.6 mm), day 5 (2.4 to 29.9 mm), day 6 (up to 23.3 mm), and day 7 (up to 19.8 mm). Maximum pain intensity after insertion of the first archwire was calculated at a pooled average of 68.2 mm (4 trials; 95% CI = 47.1, 89.3 mm), but with again high heterogeneity and extremely wide 95% prediction (6.1 to 100 mm). At the first adjustment appointment 1 month post-insertion during which the wire was changed, meta-analysis of two trials indicated the average pain at day 1 to be much lower than post-insertion (average = 25.4 mm) and still reduced to day 3 (average = 14.8 mm). Reported use of analgesics was 62.3% 6 h post-insertion (2 trials; 95% CI = 42.1%, 80.6%) and reduced at day 1 to 43.1% (6 trials; 95% CI = 17.7, 70.5%), at day 2 to 26.5% (4 trials; 95% = 21.0%, 32.4%), at day 3 to 11.9% (5 trials; 95% CI = 8.0%, 16.3%) and then fell to less than 10%. However, about every second patient (8 trials; average = 54.5%; 95% CI = 29.7%, 78.2%) reported taking at least once analgesic during the first week post-insertion.

**Table 3:** Indirect meta-analyses of pain outcomes

Outcome	*n*	Measure	Effect (95% CI)	*P*	tau^2^ (95% CI)	*I*^2^ (95% CI)	95% prediction
Pain pre-insertion	5	Mean	1.83 (− 1.31, 4.98)	0.18	3.10 (0, 49.47)	47% (0%, 81%)	− 4.82, 8.49
Pain post-insertion	13	Mean	12.38 (7.78, 16.99)	< 0.001	44.25 (19.15, > 100)	97% (96%, 98%)	− 2.98, 27.75
Pain at 2 h	6	Mean	22.98 (4.83, 41.13)	0.02	> 100 (> 100, > 100)	99% (99%, 99%)	− 27.75, 73.71
Pain at 4 h	11	Mean	31.91 (19.22, 44.60)	0.002	> 100 (> 100, > 100)	99% (99%, 99%)	− 12.32, 76.13
Pain at 6 h	11	Mean	40.86 (28.56, 53.15)	< 0.001	> 100 (> 100, > 100)	99% (99%, 99%)	− 1.19, 82.90
Pain at 8 h	5	Mean	29.85 (21.02, 38.68)	0.007	41.38 (9.53, > 100)	90% (80%, 95%)	7.01, 52.69
Pain at 12 h	3	Mean	47.70 (− 2.21, 97.60)	0.05	> 100 (95.02, > 100)	97% (95%, 99%)	< − 100, > 100
Pain at day 1	29	Mean	42.42 (37.32, 47.52)	< 0.001	> 100 (96.07, > 100)	97% (96%, 98%)	15.64, 69.20
Pain at day 2	24	Mean	37.44 (32.48, 42.41)	< 0.001	> 100 (67.68, > 100)	99% (99%, 99%)	13.75, 61.13
Pain at day 3	30	Mean	30.22 (26.14, 34.29)	< 0.001	99.72 (57.16, > 100)	94% (93%, 95%)	9.36, 51.07
Pain at day 4	17	Mean	22.63 (18.60, 26.66)	< 0.001	45.51 (20.57, > 100)	94% (92%, 96%)	7.69, 37.57
Pain at day 5	16	Mean	16.13 (12.44, 19.82)	< 0.001	37.91 (16.19, > 100)	94% (92%, 96%)	2.41, 29.85
Pain at day 6	16	Mean	11.09 (7.78, 14.39)	< 0.001	29.98 (13.09, 85.92)	94% (91%, 95%)	− 1.12, 23.29
Pain at day 7	23	Mean	9.03 (6.47, 11.59)	< 0.001	25.14 (12.97, 67.64)	93% (90%, 95%)	− 1.71, 19.77
Pain at day 8	2	Mean	5.95 (− 45.78, 57.68)	0.38	21.62 (-)	54% (-)	-
Pain at day 10	2	Mean	3.62 (− 30.51, 37.76)	0.41	9.32 (-)	45% (-)	-
Pain at day 14	3	Mean	1.52 (− 0.16, 3.19)	0.06	0.17 (0.03, > 100)	94% (85%, 97%)	-5.67, 8.71
Pain at 1 mo post-insertion	2	Mean	24.67 (− 87.75, > 100)	0.22	> 100 (-)	79% (-)	-
Pain at 1 mo day 1	2	Mean	25.40 (− 86.67, > 100)	0.21	> 100 (-)	87% (-)	-
Pain at 1 mo day 3	2	Mean	14.75 (− 49.91, 79.41)	0.21	38.00 (-)	71% (-)	-
Maximum pain	4	Mean	68.22 (47.12, 89.32)	0.002	> 100 (45.50, > 100)	94% (87%, 97%)	6.14, > 100
Hrs to pain onset	2	Mean	4.11 (− 16.85, 25.07)	0.24	5.13 (-)	94% (-)	-
Analgesic use at 6 h	2	Rate	62.3% (42.1%, 80.6%)	-	0.01 (-)	66% (-)	-
Analgesic use at day 1	6	Rate	43.1% (17.7%, 70.5%)	-	0.11 (0.04, 0.71)	96% (93%, 98%)	0%, 100.0%
Analgesic use at day 2	4	Rate	26.5% (21.0%, 32.4%)	-	0 (0, 0.08)	0% (0%, 85%)	15.0%, 100.0%
Analgesic use at day 3	5	Rate	11.9% (8.0%, 16.3%)	-	0 (0, 0.06)	0% (0%, 79%)	5.9%, 100.0%
Analgesic use at day 4	4	Rate	7.3% (2.1%, 14.8%)	-	0.01 (0, 0.28)	58% (0%, 86%)	0%, 46.9%
Analgesic use at day 5	3	Rate	7.9% (0.5%, 21.1%)	-	0.02 (0, 1.15)	81% (42%, 94%)	0%, 100.0%
Analgesic use at day 6	3	Rate	6.0% (1.3%, 13.1%)	-	0.01 (0, 0.35)	59% (0%, 88%)	0%, 100.0%
Analgesic use at day 7	4	Rate	2.5% (0%, 10.7%)	-	0.02 (0, 0.38)	74% (27%, 91%)	0%, 60.0%
Analgesic use during wk 1	8	Rate	54.5% (29.7%, 78.2%)	-	0.12 (0.05, 0.53)	96% (95%, 98%)	0%, 100.0%

*CI*, confidence interval; *hr*, hour; *n*, studies included; *wk*, week

**Fig. 3:**
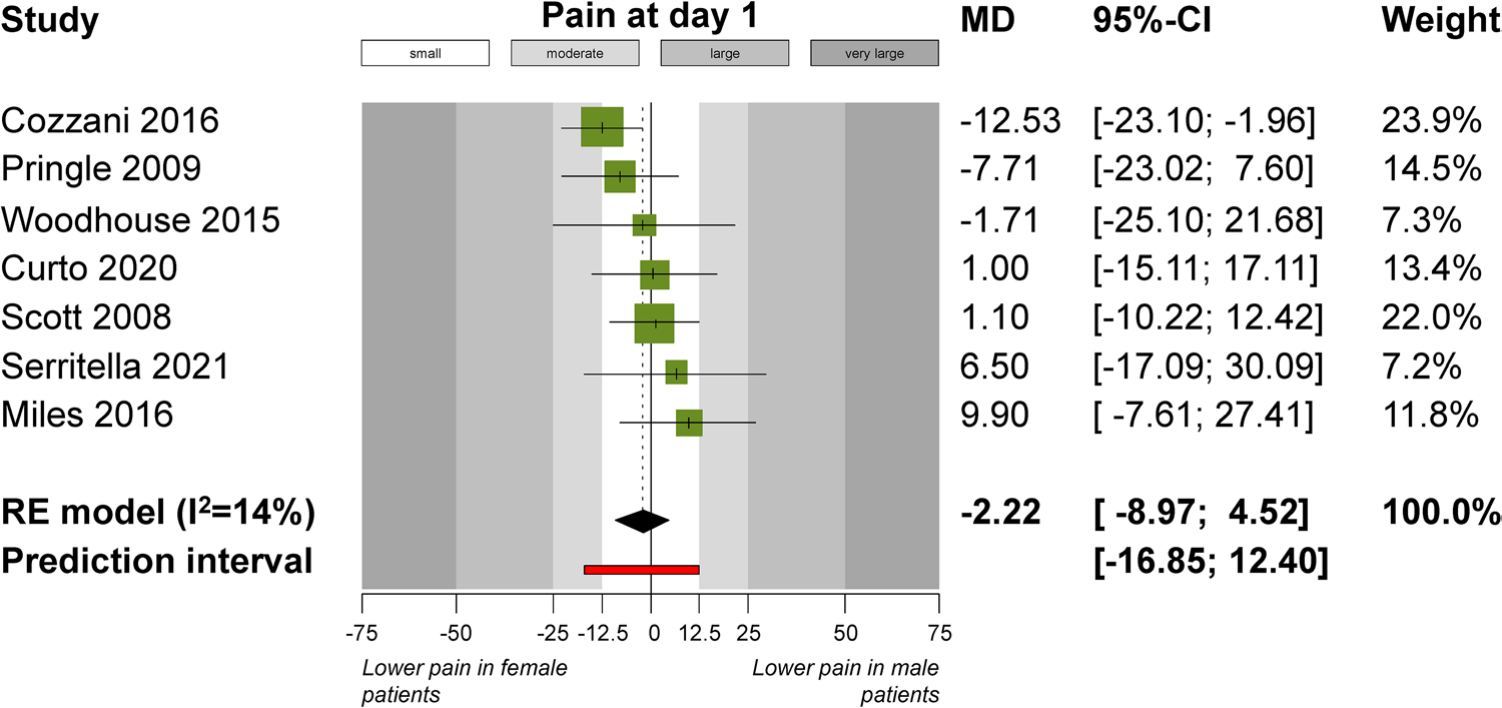
Forest plot for the direct comparisons of pain at day 1 after appliance insertion (in a 100-mm visual analogue scale) between female and male patients

Direct comparisons of pain outcomes according to various patient- or measurement-related characteristics through meta-analyses are seen in Table 4 and Fig. 3. Meta-analysis of three trials indicated that female patients reported lower pain than male patients at day 3 (MD =  − 6.3 mm; 95% CI =  − 11.9, − 0.7 mm; *P* = 0.03), but this was not consistent for any other timepoints before or after. Significant differences were seen according to the time of the day, with lower pain being reported in the evening than in the morning for day 2 (3 trials; MD =  − 3.0 mm; 95% CI =  − 5.3, − 0.6 mm; *P* = 0.01), day 3 (3 trials; MD =  − 3.1 mm; 95% CI =  − 5.0, − 1.1 mm; *P* = 0.002), day 4 (3 trials; MD =  − 2.7 mm; 95% CI =  − 4.0, − 1.3 mm; *P* < 0.001), and day 6 (2 trials; MD =  − 1.1 mm; 95% CI =  − 1.8, − 0.4 mm; *P* = 0.002). Pain during chewing was significantly higher than spontaneous pain at day 1 (2 trials; MD = 19.2 mm; 95% CI = 7.9, 30.4 mm; *P* < 0.001) and at day 3 (2 trials; MD = 21.1 mm; 95% CI = 8.0, 34.3 mm; *P* = 0.002). Finally, occlusion of the front teeth resulted in greater pain compared to pain during occlusion of the back teeth at day 2 (2 trials; MD = 14.6 mm; 95% CI = 2.3, 27.0 mm; *P* = 0.02), while pain during occlusion of the back teeth was greater than spontaneous pain (2 trials; MD = 12.4 mm; 95% CI = 1.4, 23.4 mm; *P* = 0.03).

**Table 4:** Direct meta-analyses

Outcome	Experimental vs reference group	*n*	Effect* (95% CI)	*P*	tau^2^ (95% CI)	*I*^2^ (95% CI)	95% prediction
Pain post-insertion	Female vs male	3	2.72 (− 17.75, 23.19)	0.79	> 100 (25.11, > 100)	81% (42%, 94%)	< − 100, > 100
Pain at 4 h	Female vs male	5	1.03 (− 9.28, 11.34)	0.84	75.19 (0, 11.34)	55% (0%, 84%)	− 31.25, 33.31
Pain at 8 h	Female vs male	2	9.48 (− 3.31, 22.26)	0.15	0 (-)	0% (-)	-
Pain at day 1	Female vs male	7	− 2.22 (− 8.97, 4.52)	0.52	20.52 (0, > 100)	14% (0%, 75%)	− 16.85, 12.40
Pain at day 2	Female vs male	3	− 3.60 (− 11.40, 4.21)	0.37	0 (0, > 100)	0% (0%, 90%)	− 54.21, 47.02
Pain at day 3	Female vs male	7	0.22 (− 6.07, 6.52)	0.94	18.40 (0, > 100)	33% (0%, 72%)	− 13.55, 14.00
Pain at day 4	Female vs male	3	− 4.76 (− 10.45, 0.93)	0.10	0 (0, > 100)	0% (0%, 90%)	− 41.63, 32.11
Pain at day 5	Female vs male	3	− 6.28 (− 11.89, − 0.68)	0.03	6.29 (0, > 100)	18% (0%, 91%)	− 54.64, 42.07
Pain at day 6	Female vs male	3	− 3.97 (− 7.94, 0.01)	0.05	0 (0, > 100)	0% (0%, 90%)	− 29.71, 21.78
Pain at day 7	Female vs male	6	− 2.35 (− 5.20, 0.50)	0.11	0 (0, 92.91)	0% (0%, 75%)	− 6.38, 1.69
Maximum pain	Female vs male	2	− 7.60 (− 18.25, 3.05)	0.16	0 (-)	0% (-)	-
Analgesic use during wk 1	Female vs male	4	OR 0.98 (0.37, 2.56)	0.96	0.40 (0, 11.57)	40% (0%, 80%)	0.03, 30.55
Pain post-insertion	Analgesic used vs no analgesic	2	1.62 (− 7.32, 10.56)	0.72	0 (-)	0% (-)	-
Pain at 4 h	Analgesic used vs no analgesic	2	13.73 (− 3.25, 30.71)	0.11	81.60 (-)	50% (-)	-
Pain at day 1	Analgesic used vs no analgesic	4	14.27 (− 2.30, 30.83)	0.09	> 100 (20.93, > 100)	79% (43%, 92%)	− 56.02, 84.56
Pain at day 3	Analgesic used vs no analgesic	4	8.69 (− 7.70, 25.08)	0.30	> 100 (34.60, > 100)	84% (58%, 94%)	− 63.98, 81.36
Pain at day 7	Analgesic used vs no analgesic	4	16.20 (− 15.08, 47.49)	0.31	> 100 (> 100, > 100)	100% (100%, 100%)	< − 100, > 100
Maximum pain	Analgesic used vs no analgesic	2	17.12 (− 7.67, 41.91)	0.18	> 100 (-)	75% (-)	-
Pain at day 2	Evening vs morning	3	− 2.98 (− 5.34, − 0.61)	0.01	0 (0, > 100)	0% (0%, 90%)	− 18.28, 12.33
Pain at day 3	Evening vs morning	3	− 3.07 (− 5.03, − 1.11)	0.002	0 (0, > 100)	0% (0%, 90%)	− 15.77, 9.64
Pain at day 4	Evening vs morning	3	− 2.66 (− 4.04, − 1.29)	< 0.001	0 (0, 83.16)	0% (0%, 90%)	− 11.58, 6.25
Pain at day 5	Evening vs morning	2	− 2.24 (− 4.91, 0.43)	0.10	2.15 (-)	42% (-)	-
Pain at day 6	Evening vs morning	2	− 1.11 (− 1.81, − 0.40)	0.002	0 (-)	0% (-)	-
Pain at day 7	Evening vs morning	2	− 0.78 (− 1.95, 0.39)	0.19	0.36 (-)	14% (-)	-
Pain post-insertion	Chewing vs spontaneous	2	− 0.45 (− 6.24, 5.34)	0.88	0 (-)	0% (-)	-
Pain at day 1	Chewing vs spontaneous	2	19.16 (7.91, 30.41)	< 0.001	0 (-)	0% (-)	-
Pain at day 2	Chewing vs spontaneous	2	20.90 (− 1.32, 43.11)	0.07	> 100 (-)	71% (-)	-
Pain at day 3	Chewing vs spontaneous	2	21.11 (7.96, 34.26)	0.002	0 (-)	0% (-)	-
Pain at day 1	Occluding front teeth vs occluding back teeth	2	11.01 (− 0.78, 22.81)	0.07	0 (-)	0% (-)	-
Pain at day 2	Occluding front teeth vs occluding back teeth	2	14.63 (2.28, 26.97)	0.02	0 (-)	0% (-)	-
Pain at day 3	Occluding front teeth vs occluding back teeth	2	12.97 (− 1.02, 26.96)	0.07	0 (-)	0% (-)	-
Pain at day 7	Occluding front teeth vs occluding back teeth	2	0.04 (− 10.37, 10.44)	0.99	0 (-)	0% (-)	-
Pain at day 1	Occluding back teeth vs spontaneous	2	12.40 (1.44, 23.36)	0.03	0 (-)	0% (-)	-
Pain at day 2	Occluding back teeth vs spontaneous	2	7.15 (− 6.77, 21.07)	0.31	58.95 (-)	54% (-)	-
Pain at day 3	Occluding back teeth vs spontaneous	2	6.56 (− 9.66, 22.78)	0.43	66.37 (-)	48% (-)	-
Pain at day 7	Occluding back teeth vs spontaneous	2	5.70 (− 1.79, 13.20)	0.14	0 (-)	0% (-)	-

^*^Given as mean difference, except if otherwise noted

*CI*, confidence interval; *hr*, hour; *n*, studies; *wk*, week

Meta-regression and subgroup analyses were used to identify potential sources of heterogeneity in the indirect meta-analyses according to patient- or treatment-related characteristics (Appendix 7–10). No significant effect of patient age or patient sex was seen across studies, while significant effects of baseline irregularity were seen for days 1, 5, and 6 (Appendix 7). However, the influence of irregularity on pain was not consistent in direction across the various timepoints and caution is needed while interpreting these. Incorporation of extractions into the treatment was associated with significantly higher pain scores at 6 h and days 3–7 (*P* < 0.10), with relative consistency. Bracket slot size was likewise associated with reported pain, as 0.018-inch brackets were associated with higher pain scores than 0.022-inch brackets at day 1 (averages of 50.2 mm vs 39.6 mm, respectively; *P* = 0.01) and in terms of analgesic use during the 1st week (rates of 100% vs 56%, respectively; *P* < 0.001). Finally, significantly higher pain was reported during treatment of the lower arch compared to the upper arch for pain at 6 h and pain at 7 days, but these effects were not consistent for other timepoints.

### Reporting biases and sensitivity analyses

Assessment of reporting biases (including small-study effects) for meta-analyses with at least 10 studies is seen in Appendix 11 with contour-enhanced funnel plots and in Appendix 12 with Egger’s linear regression test. For 3 of the 10 tested meta-analyses, signs of small-study effects were seen (*P* < 0.10) and sensitivity analyses according to the study precision were performed. For the outcome of pain at 4 h, the most precise half of the studies showed significantly lower pain scores compared to the least precise half (averages of 16.9 mm vs 45.4 mm, respectively; *P* < 0.001). For the other two outcomes (pain at 6 h and pain at day 1), no significant differences were found. Using the results of the sensitivity analysis, a clearer stepwise increase in pain intensity was found from 6 h to day 1.

Sensitivity analysis according to whether the patients recruited in the trial were selected based on any pain-related eligibility criteria (or not) did not find any significant threats to robustness (Appendix 13). Sensitivity analysis according to adequacy of the sample size (judged arbitrarily with the cut-off of 40 patients/trial) found significant differences for the outcomes of pain at days 4–6, where trials with adequate sample size reported significantly lower pain values (probably due to higher precision). Still, observing the pain profile obtained solely from trials with adequate sample size, a similar pain pattern was seen, with increasing pain intensity from post-insertion (on average 12.2 mm) to a peak at day 1 (on average 46.5 mm), and then gradually reducing to day 3 (on average 30.3 mm) until day 7 (on average 6.9 mm).

### Certainty of evidence

Our certainty in the evidence from direct comparisons was assessed using the GRADE approach in Table 5. Moderate quality of evidence due to inconsistency was found for the lack of effect of (i) patient sex or (ii) use of analgesic, as well as for the increased pain (a) in the morning (compared to the evening), (b) when occluding the back teeth (compared to occluding the front teeth), and (c) when occluding the back teeth (compared to spontaneously). High-quality evidence supported the increased pain during chewing compared to spontaneous pain.

**Table 5:**
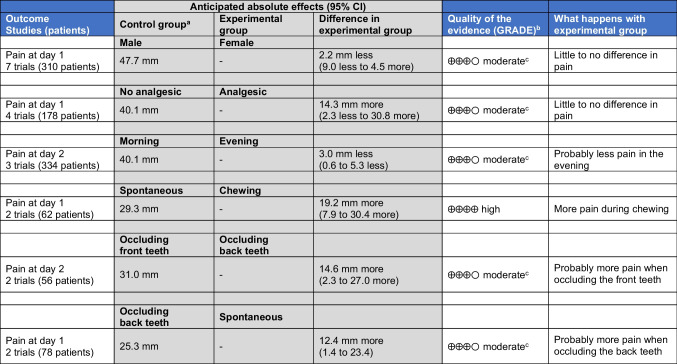
Summary of findings table according to the GRADE approach.

Intervention: orthodontic treatment with fixed appliances without adjuncts (initial phase of levelling/alignment)/Population: adolescent and adult patients with any kind of malocclusion/Setting: university clinics, hospitals and private practice (Australia, Brazil, China, Egypt, Great Britain, India, Iran, Italy, Norway, Poland, Saudi Arabia, Spain, Syria, Turkey, United Arab Emirates, and United States of America)

^a^Response in the control group is based on random-effects meta-analysis pain among the control groups

^b^Starts from “high”

^c^Downgraded by one level for bias due to inconsistency

*CI*, confidence interval; *GRADE*, Grading of Recommendations Assessment, Development and Evaluation

## Discussion

### Results in context

The present review summarizes the evidence from randomized clinical trials in orthodontics that assessed patient-reported pain during orthodontic levelling/alignment with fixed appliances. A total of 37 parallel-group randomized trials were ultimately included in the review that covered 2277 patients with a mean age of 17.5 years. These trials assessed various interventions (different appliances, techniques, adjuncts, or management strategies) and their comparative effects on reported pain, but this was not within the scope of this review. Rather, the aim of this systematic review was to assess the pattern and expected intensity of orthodontically induced pain during levelling/alignment from randomized clinical trials that can be used both to inform clinical practice and as a benchmark for new randomized trials.

Based on the VAS, the pain pattern that emerged from the evidence indicated a quick initiation of pain response with an average pain reading of 12.9 mm post-insertion (Table 3) that gradually increased with a peak at day 1 of around 42.4 mm on average and then a gradual daily reduction, so that at the end of the 1st week an average reading of 9.0 mm was observed, while the maximum felt pain was on average 68.2 mm. Interestingly, about half of all patients (54.%) took analgesics at least once during the 1st week with their use peaking at 6 h post-insertion and then diminishing; that is to say before the peak daily reading has been reached. Pain during orthodontic treatment has been suggested to be due to an inflammatory response in the periodontal ligament [46, 47], while others suggest it is due to a combination of pressure, ischemia, inflammation, edema [48], and the release of proinflammatory mediators, which sensitize nociceptors in the periodontal ligament and reduce the pain threshold [49].

Available evidence indicated that within the whole first week a daily fluctuation in reported pain was seen, so that pain gradually diminished each day from morning to evening up to at least the 6th day post-insertion (Table 4). This can be attributed to the reduction of inflammatory mediators within the periodontal ligament post-activation of the archwire. This gradual reduction within the day is probably not a temporal observation of circadian effects [50, 51] within the day, but rather the effect of greater amounts of time having elapsed in the evening since the original archwire activation and is consistent with other studies [43, 52, 53].

Interestingly no significant modifying effect was seen from patient age on pain levels reported by the patient. This finding corroborates with many studies reporting no overall effect of patient age on pain [16, 42–45]; however, other studies reported contradicting results with more intense pain noticed in patients older than 13 years [54] or 16 years of age [6]. Likewise, no sign of a consistent modifying effect was seen from patient sex on pain levels as reported by the patient, which is similar to many studies also failing to find gender-specific differences [55–58]. Sandhu and Leckie [16] though found significant differences in pain trajectory and peak pain intensity when dividing their sample both by age (12–15 and 15–18 years of age) and sex with girls experiencing greater orthodontic pain than boys, and this difference increased with age. However, this is just a single cohort study with moderate sample size (30 patients per subgroup) and with varying use of analgesics while the performed dichotomization of patient age might be questionable if this is not dictated by clear biological differences [59]. Additionally, re-analysis of the 4 available trial datasets failed to find any significant effect for patient age or sex, even after using sex and age (continuous or with the 15-years cut-off) as moderators (data not shown), making therefore the effect of these confounders on orthodontic pain inconclusive.

Pain induced by orthodontic appliances was increased when the activity of the masticatory system increased (chewing or biting) compared to spontaneous pain and then it was also increased when the patient fitted (brought in contact) either the posterior or the anterior teeth (but to a lesser extent compared to chewing or biting). It is possible that masticatory activity such as chewing and biting might exert compressive forces on the previously sensitized nociceptors of the periodontal ligament and lead to an increase pain response than at rest.

Analgesic use was also reported by some of the included studies, while other studies either prohibited the use of analgesics by the patients, or did not report at all on this aspect. It is also important to note here that studies assessing only pharmacological interventions were excluded from the present review, while study-arms of pharmacological interventions were omitted if other study-arms without those could be used. Although prohibiting the patients from the use of analgesics might benefit the experimental design of assessing the actual pain levels felt by the patients, some might find the avoidance of a proved effective pain control means [22] difficult to justify ethically—not to mention that it might hamper the generalizability of the trial’s results. In this review, it was decided not to limit study inclusion according to the prohibition of self-administered analgesics, but to report this for transparency reasons. The use of analgesics, apart from being a surrogate endpoint for felt pain intensity, might also impact the duration of orthodontic treatment, since their short-term use has been reported to potentially influence tooth movement rate in animals [60]. However, whether this effect might also be applicable to humans and for the doses/administration frequency used during orthodontic levelling/alignment remains questionable—as does the clinical relevance of any such theoretical effect.

In all studies included in the analyses, patient-reported pain was measured with a VAS, which is one of the most commonly used tools to assess pain/discomfort associated with orthodontic procedures. The VAS has several advantages, including among others being easily understood by patients, having adequate sensitivity to small changes, and being adequately reproducible [61, 62]. It is important to stress though that the VAS measures global discomfort as reported from the patient but does not specifically differentiate between different tissues, areas, or movements.

### Strengths and limitations

This review has several strengths, including its a priori protocol [63], a comprehensive literature search, the use of modern up-to-date methods for data analysis [34], the application of the GRADE approach to assess the strength of provided recommendations [40], and the transparent provision of all data [64].

Some limitations though do exist. Even though randomized trials were included, these were handled as observational studies to assess the average pain trajectory and non-randomized cohort studies could have also been considered to increase the review’s sample. However, (a) it has been reported that the results from randomized and non-randomized trials might vary considerably [65, 66] and (b) more often than not, randomized and non-randomized studies include different populations due to their nature [67, 68] and this would make the review’s results not directly applicable to future randomized trials. In addition, many trials assessed only a handful of timepoints pre- and post-insertion of the appliances, and this could distort the observed pain profile if daily readings were not available for the whole week. Another limitation derives from the fact that many included trials suffered from selective reporting of both patient characteristics and potential effect modifiers and many planned analyses could not ultimately be performed. Finally, many trials were of limited sample size and some meta-analyses were based on few and/or small trials and this might have affected the precision of the estimates [69].

## Conclusions

Evidence from this systematic review of randomized trials on pain during levelling/alignment indicates that orthodontic pain starts shortly after appliance insertion, increases quickly within the first hours to a peak at day 1 post-insertion, and then gradually diminishes (but does not completely disappear) within the first week. Several important factors related to the patient, the treatment, or outcome measurement were identified that can influence patient-reported pain during orthodontic levelling/alignment. These data can be used to adequately inform patients prior to treatment and to properly inform future clinical trials on this subject.

## Supplementary Information

Below is the link to the electronic supplementary material.

Supplementary file1 (PDF 1279 KB)

## Data Availability

Data is available via Zenodo (https://doi.org/10.5281/zenodo.7315512).
