# Supplementary material for: Pain profile during orthodontic levelling and alignment with fixed appliances reported in randomized trials: a systematic review with meta-analyses
Source: Clin Oral Investig. 2023 Mar 6;27(5):1851–68. doi: 10.1007/s00784-023-04931-5 (PMC10159949; doi:10.1007/s00784-023-04931-5)
Supplement: Supplementary file 1 — Supplementary file1 (PDF 1279 KB) [file 784_2023_4931_MOESM1_ESM.pdf]

# Supplementary material

**Appendix 1.** Supplementary methods for this systematic review.

Additional details on review methods and post hoc deviations from the review protocol.

Additional details on methods.

- Within-persons (split-mouth) randomized trials were excluded, since it was assumed that different pain responses from different sides of the mouth of a patient might not be easily differentiated, while a previous pain stimulus was expected to influence a subsequent measurement.
- In instances where medians and interquartile ranges were provided, we took the median to be a close approximation of the mean. The SD was calculated from the interquartile range according to Wan et al. (Wan, Wang, Liu, & Tong, 2014).
- Raw data available for two studies (provided for previous reviews) were analyzed by calculating summary statistics and running linear regressions using unstandardized coefficients with their 95% Confidence Intervals (CI).
- The produced forest plots for direct comparisons were augmented with contours denoting the magnitude of expected effects: small (up to half a Standard Deviation [SD]), moderate (half to one SD), large (one to two SDs) and very large (more than 2 SDs). The SD for each outcome's forest plot was based on the average pre-treatment SD of the response variable across all eligible studies for each meta-analysis (rounded up to 2 decimals).
- The program WebPlotDigitizer was used to extract numerical data from figures provided in the paper for Cioffi 2012 and Montebugnoli 2020.
- The outcome 'pain 1 hour after insertion' was collated with the outcome 'pain directly after insertion of the appliance'.
- The data from the Miles 2010 trial was not used in the analyses, since a Likert scale was reportedly used, but the authors didn't specify from which values the scale started (0 or 1).
- The data from the trial Sfondrini 2020 was not used, since the authors didn't report how marks were converted to numerical data.

Deviations from the review protocol.

- Initially, additional subgroup / meta-regression analyses were planned, but could ultimately not be conducted due to lack of data: subsets according to patient ethnicity, age, smoking, body mass index, sport activity level.

## REFERENCES

Wan, X., Wang, W., Liu, J., & Tong, T. (2014). Estimating the sample mean and standard deviation from the sample size, median, range and/or interquartile range. *BMC Medical Research Methodology*, *14*, 135.

**Appendix 2.** Literature searches performed together with the used search strategies (as of October 1st, 2022).

| Database | Search Strategy | Filters | Hits |
|---|---|---|---|
| PubMed | [PubMed precision maximizing strategy]* AND (orthodon* OR "fixed appliances" OR "braces" OR "brackets") AND (pain OR discomfort) | Randomized Controlled Trial; Humans | 548 |
| Scopus | (orthodon* OR "fixed appliances" OR "braces" OR "brackets") AND (pain OR discomfort) | Subject area: Dentistry<br>Keywords: Human; Humans; Controlled Study; Randomized Controlled Trial; Comparative Study; Controlled Clinical Trial; Prospective Study; Prospective Studies; Double-Blind Method; Single Blind Procedure; Single-Blind Method | 477 |
| Embase | (orthodon* OR 'fixed appliances' OR 'braces'/exp OR 'braces' OR 'brackets') AND ('pain'/exp OR pain OR 'discomfort'/exp OR discomfort) | Controlled Clinical Trial; Randomized Controlled Trial; Embase | 216 |
| Web of Science | (orthodon* OR "fixed appliances" OR "braces" OR "brackets") AND (pain OR discomfort) | Clinical Trial; Dentistry Oral Surgery Medicine | 357 |
| Virtual Health Library | (orthodon* OR "fixed appliances" OR "braces" OR "brackets") AND (pain OR discomfort) AND (random* OR blind* OR control*) | | 102 |
| **Sum with overlap** | | | **1700** |
| **Sum without overlap** | | | **1177** |

## **Appendix 3.** List of studies identified from the literature search, with their inclusion / exclusion status.

| Nr | Paper | Status |
|---|---|---|
| 1 | Harazaki M, Isshiki Y. Soft laser irradiation effects on pain reduction in orthodontic treatment. Bull Tokyo Dent Coll. 1997;38(4):291-5. | Full-text unavailable |
| 2 | Jia Y, Chen B, Cai S, Hu T. [TEAS for prevention and treatment of orthodontic toothache and oral dysfunction: a randomized controlled trial]. Zhongguo Zhen Jiu. 2016;36(5):485-90. | Full-text unavailable |
| 3 | Pinheiro ALB, Bittencourt MAV, Cangussu MCT, Ferreira RFA, Ferreira Filho RFA. Avaliação clínica da ação antiálgica do laser de baixa potência após instalação de separadores ortodônticos. Rev Assoc Paul Cir Dent. 2008;62(2):98-104. | Full-text unavailable |
| 4 | Xiao L, Chen RX, Luo N. [Effect of single low level laser therapy on initial pain during fixed orthodontic treatment]. Shanghai Kou Qiang Yi Xue. 2019;28(5):549-52. | Full-text unavailable |
| 5 | Xu X, Zhang L, Jiang Y, Huang Y, Huang S, Yang S. [Clinical research of music in relieving orthodontic pain]. Hua Xi Kou Qiang Yi Xue Za Zhi. 2013;31(4):365-8. | Full-text unavailable |
| 6 | [No authors] 2018 Sports Medicine Australia Conference Perth Convention and Exhibition Centre 10th – 13th October 2018. Journal of Science and Medicine in Sport. 2018;21. | Not relevant to orthodontics |
| 7 | Abbott A, Halvorsen M, Dedering A. Is there a need for cervical collar usage post anterior cervical decompression and fusion using interbody cages? A randomized controlled pilot trial. Physiother Theory Pract. 2013;29(4):290-300. | Not relevant to orthodontics |
| 8 | AbdelRady MM, Ali WN, Younes KT, Talaat EA, AboElfadl GM. Analgesic efficacy of single- shot adductor canal block with levobupivacaine and dexmedetomidine in total knee arthroplasty: A randomized clinical trial. Egyptian Journal of Anaesthesia. 2021;37(1):386-93. | Not relevant to orthodontics |
| 9 | Abdul Wahab PU, Madhu Laxmi M, Senthil Nathan P. Wound infection after therapeutic tooth extraction with and without antibiotics. International Journal of Pharma and Bio Sciences. 2013;4(4):B1277-B81. | Not relevant to orthodontics |
| 10 | Abduljabbar T, Mehta NR, Forgione AG, Clark RE, Kronman JH, Munsat TL, et al. Effect of increased maxillo-mandibular relationship on isometric strength in TMD patients with loss of vertical dimension of occlusion. Cranio - Journal of Craniomandibular and Sleep Practice. 1997;15(1):57-66. | Not relevant to orthodontics |
| 11 | Abotaleb B, Bi R, Telha W, Zhao W, Li Y, Zhu S. Treatment measures of hemimandibular hyperplasia and associated facial deformities. Journal of Cranio-Maxillofacial Surgery. 2021;49(2):126-34. | Not relevant to orthodontics |
| 12 | Abreu AVd, Mello APd, Trovão GS, Fontenelle CRdC. Avaliação clínico-radiográfica da mobilidade da lordose lombar. Rev bras ortop. 2007;42(10):313-23. | Not relevant to orthodontics |
| 13 | Abu-Mostafa N-A, Alqahtani A, Abu-Hasna M, Alhokail A, Aladsani A. A randomized clinical trial compared the effect of intra-alveolar 0.2 % Chlorohexidine bio-adhesive gel versus 0.12% Chlorohexidine rinse in reducing alveolar osteitis following molar teeth extractions. Med oral patol oral cir bucal (Internet). 2015;20(1):e82-e7. | Not relevant to orthodontics |
| 14 | Added MAN, Added C, Kasawara KT, Rotta VP, de Freitas DG. Effects of a Knee Brace With a Patellar Hole Versus Without a Patellar Hole in Patients With Knee Osteoarthritis: A Double-Blind, Randomized Controlled Trial. Eval Health Prof. 2018;41(4):512-23. | Not relevant to orthodontics |
| 15 | Agarwal N, Dhawan J, Kumar D, Anand A, Tangri K. Effectiveness of two topical anaesthetic agents used along with audio visual aids in paediatric dental patients. Journal of Clinical and Diagnostic Research. 2017;11(1):ZC80-ZC3. | Not relevant to orthodontics |
| 16 | Agarwal S, Sharma A. A clinicaltrial comparing functional outcomes inmedial compartment OA knee patients treated with a pneumatic unloading brace and resisted hipand knee exercises. Osteoporosis International. 2018;29(1):S206-S7. | Not relevant to orthodontics |
| 17 | Akeel R, Al-Jasser N. Temporomandibular disorders in Saudi females seeking orthodontic treatment. Journal of Oral Rehabilitation. 1999;26(9):757-62. | Not relevant to orthodontics |
| 18 | Akhter R, Morita M, Esaki M, Nakamura K, Kanehira T. Development of temporomandibular disorder symptoms: A 3-year cohort study of university students. Journal of Oral Rehabilitation. 2011;38(6):395-403. | Not relevant to orthodontics |
| 19 | Aksoy MK, Altan L, Güner A. The effectiveness of soft and semi-rigid cervical collars on acute cervical radiculopathy. European Research Journal. 2018;4(1):16-25. | Not relevant to orthodontics |
| 20 | Al Quran FAM, Kamal MS. Anterior midline point stop device (AMPS) in the treatment of myogenous TMDs: Comparison with the stabilization splint and control group. Oral Surgery, Oral Medicine, Oral Pathology, Oral Radiology and Endodontology. 2006;101(6):741-7. | Not relevant to orthodontics |
| 21 | Alajbeg I, Brakus RB, Brakus I. Comparison of amitriptyline with stabilization splint and placebo in chronic TMD patients: A pilot study. Acta Stomatologica Croatica. 2018;52(2):114-22. | Not relevant to orthodontics |
| 22 | Al-Ani Z, Gray RJ, Davies SJ, Sloan P, Glenny AM. Stabilization splint therapy for the treatment of temporomandibular myofascial pain: a systematic review. Journal of dental education. 2005;69(11):1242-50. | Not relevant to orthodontics |
| 23 | Albayrak Aydin N, Yazicioğlu K. Cervical intermittent traction: Does it really work incervical radiculopathy due to herniated disc? Turkiye Fiziksel Tip ve Rehabilitasyon Dergisi. 2012;58(4):277-82. | Not relevant to orthodontics |
| 24 | Al-Bluwi MT, Sadat-Ali M, Al-Habdan IM, Azam MQ. Efficacy of EZStep in the management of plantar fasciitis: a prospective, randomized study. Foot Ankle Spec. 2011;4(4):218-21. | Not relevant to orthodontics |
| 25 | Albrecht-Olsen P, Kristensen G, Burgaard P, Joergensen U, Toerholm C. The arrow versus horizontal suture in arthroscopic meniscus repair. A prospective randomized study with arthroscopic evaluation. Knee Surg Sports Traumatol Arthrosc. 1999;7(5):268-73. | Not relevant to orthodontics |
| 26 | AlDhelai TA, Khalil AM, Elhamouly Y, Dowidar KML. Influence of active versus passive parental presence on the behavior of preschoolers with different intelligence levels in the dental operatory: a randomized controlled clinical trial. BMC Oral Health. 2021;21(1):420. | Not relevant to orthodontics |
| 27 | Aleksiev AR. Ten-year follow-up of strengthening versus flexibility exercises with or without abdominal bracing in recurrent low back pain. Spine (Phila Pa 1976). 2014;39(13):997-1003. | Not relevant to orthodontics |
| 28 | Alencar F, Jr., Becker A. Evaluation of different occlusal splints and counselling in the management of myofascial pain dysfunction. J Oral Rehabil. 2009;36(2):79-85. | Not relevant to orthodontics |
| 29 | Alexander CM. Character development: A value-added concept. Seminars in Orthodontics. 2001;7(2):67-73. | Not relevant to orthodontics |
| 30 | Alimanovic D, Pedersen TK, Matzen LH, Stoustrup P. Comparing Clinical and Radiological Manifestations of Adolescent Idiopathic Condylar Resorption and Juvenile Idiopathic Arthritis in the Temporomandibular Joint. Journal of Oral and Maxillofacial Surgery. 2021;79(4):774-85. | Not relevant to orthodontics |
| 31 | Al-Khotani A, Bello LA, Christidis N. Effects of audiovisual distraction on children's behaviour during dental treatment: a randomized controlled clinical trial. Acta Odontol Scand. 2016;74(6):494-501. | Not relevant to orthodontics |
| 32 | Allen CS, Flynn TW, Kardouni JR, Hemphill MH, Schneider CA, Pritchard AE, et al. The use of a pneumatic leg brace in soldiers with tibial stress fractures--a randomized clinical trial. Mil Med. 2004;169(11):880-4. | Not relevant to orthodontics |
| 33 | Almubarak H, Alzahrani FA, Kaleem SM, Zakirulla M. Prevalence of temporomandibular disorders (TMDs) in relation to estrogen levels among females in aseer region, Saudi Arabia. Annals of Tropical Medicine and Public Health. 2020;23(16). | Not relevant to orthodontics |
| 34 | Alnassar I, Altinawi M, Rekab MS, Katbeh I, Khasan A, Almokaddam H. Pain assessment following endodontic treatment using two automated systems compared to manual treatment in primary molars. Dent Med Probl. 2021;58(3):305-10. | Not relevant to orthodontics |
| 35 | Al-Omiri MK, Al Nazeh AA, Kielbassa AM, Lynch E. Randomized controlled clinical trial on bleaching sensitivity and whitening efficacy of hydrogen peroxide versus combinations of hydrogen peroxide and ozone. Sci Rep. 2018;8(1):2407. | Not relevant to orthodontics |
| 36 | Al-Omiri MK, Alqahtani NM, Alahmari NM, Hassan RA, Al Nazeh AA, Lynch E. Treatment of symptomatic, deep, almost cariously exposed lesions using ozone. Sci Rep. 2021;11(1):11166. | Not relevant to orthodontics |
| 37 | Alpaslan C, Kahraman S, Güner B, Cula S. Does the use of soft or hard splints affect the short-term outcome of temporomandibular joint arthrocentesis? Int J Oral Maxillofac Surg. 2008;37(5):424-7. | Not relevant to orthodontics |
| 38 | Alshaharani MS, Lohman EB, Bahjri K, Harp T, Alameri M, Jaber H, et al. Comparison of Protonics™ Knee Brace With Sport Cord on Knee Pain and Function in Patients With Patellofemoral Pain Syndrome: A Randomized Controlled Trial. J Sport Rehabil. 2020;29(5):547-54. | Not relevant to orthodontics |
| 39 | Alwali AA. Spinal brace in tuberculosis of spine. Neurosciences. 2003;8(1):17-22. | Not relevant to orthodontics |
| 40 | Amorfini L, Migliorati M, Drago S, Silvestrini-Biavati A. Immediately Loaded Implants in Rehabilitation of the Maxilla: A Two-Year Randomized Clinical Trial of Guided Surgery versus Standard Procedure. Clin Implant Dent Relat Res. 2017;19(2):280-95. | Not relevant to orthodontics |
| 41 | Annaswamy TM, Cunniff KJ, Kroll M, Yap L, Hasley M, Lin CK, et al. Lumbar Bracing for Chronic Low Back Pain: A Randomized Controlled Trial. Am J Phys Med Rehabil. 2021;100(8):742-9. | Not relevant to orthodontics |
| 42 | Antoniazzi RP, Vieira AR, Da Rosa JL, Ferrazo KL, Zanatta FB, Feldens CA. Periodontal dressing after surgical crown lengthening: a randomized clinical trial. Acta odontologica Scandinavica. 2014;72(8):1025-31. | Not relevant to orthodontics |
| 43 | Arazpour M, Hutchins SW, Bani MA, Curran S, Aksenov A. The influence of a bespoke unloader knee brace on gait in medial compartment osteoarthritis: a pilot study. Prosthet Orthot Int. 2014;38(5):379-86. | Not relevant to orthodontics |
| 44 | Arima T, Tomonaga A, Toyota M, Inoue SI, Ohata N, Svensson P. Does restriction of mandibular movements during sleep influence jaw-muscle activity? Journal of Oral Rehabilitation. 2012;39(7):545-51. | Not relevant to orthodontics |
| 45 | Armstrong BM, Hall M, Crawfurd E, Smith TO. A feasibility study for a pragmatic randomised controlled trial comparing cast immobilisation versus no immobilisation for patients following first-time patellar dislocation. Knee. 2012;19(5):696-702. | Not relevant to orthodontics |
| 46 | Aroca JP, Cardoso PMF, Favarão J, Zanini MM, Camilotti V, Busato MCA, et al. Auricular acupuncture in TMD - A sham-controlled, randomized, clinical trial. Complement Ther Clin Pract. 2022;48:101569. | Not relevant to orthodontics |
| 47 | Arslan H, Doganay E, Karatas E, Unlu MA, Ahmed HMA. Effect of Low-level Laser Therapy on Postoperative Pain after Root Canal Retreatment: A Preliminary Placebo-controlled, Triple-blind, Randomized Clinical Trial. Journal of Endodontics. 2017;43(11):1765-9. | Not relevant to orthodontics |
| 48 | Aslam S, Francis PG, Rao BHS, Ummar M, Issac JK, Nair RB. A double blind study on the efficacy of local application of hemocoagulase solution in wound healing. Journal of Contemporary Dental Practice. 2013;14(3):394-400. | Not relevant to orthodontics |
| 49 | Asutay F, Yolcu Ü, Geçör O, Acar AH, Öztürk SA, Malkoç S. An evaluation of effects of platelet-rich-fibrin on postoperative morbidities after lower third molar surgery. Niger J Clin Pract. 2017;20(12):1531-6. | Not relevant to orthodontics |
| 50 | Aufwerber S, Heijne A, Edman G, Grävare Silbernagel K, Ackermann PW. Early mobilization does not reduce the risk of deep venous thrombosis after Achilles tendon rupture: a randomized controlled trial. Knee Surg Sports Traumatol Arthrosc. 2020;28(1):312-9. | Not relevant to orthodontics |
| 51 | Avila-Ortiz G, Gubler M, Romero-Bustillos M, Nicholas CL, Zimmerman MB, Barwacz CA. Efficacy of Alveolar Ridge Preservation: A Randomized Controlled Trial. J Dent Res. 2020;99(4):402-9. | Not relevant to orthodontics |
| 52 | Awawdeh L, Lundy FT, Shaw C, Lamey PJ, Linden GJ, Kennedy JG. Quantitative analysis of substance P, neurokinin A and calcitonin gene-related peptide in pulp tissue from painful and healthy human teeth. International Endodontic Journal. 2002;35(1):30-6. | Not relevant to orthodontics |
| 53 | Azevedo Lemos G, Lopes Pedro da Silva P, Ribeiro Paulino M, Gomes Moreira V, Soares Beltrão RT, Dantas Batista AU. Prevalência de disfunção temporomandibular e associação com fatores psicológicos em estudantes de Odontologia. Rev cuba estomatol. 2015;52(4):0-. | Not relevant to orthodontics |
| 54 | Bågesund M, Tabrizi P. Lidocaine 20% patch vs lidocaine 5% gel for topical anaesthesia of oral mucosa. Int J Paediatr Dent. 2008;18(6):452-60. | Not relevant to orthodontics |
| 55 | Bahrami-Taghanaki H, Azizi H, Hasanabadi H, Jokar MH, Iranmanesh A, Khorsand-Vakilzadeh A, et al. Acupuncture for Carpal Tunnel Syndrome: A Randomized Controlled Trial Studying Changes in Clinical Symptoms and Electrodiagnostic Tests. Altern Ther Health Med. 2020;26(2):10-6. | Not relevant to orthodontics |
| 56 | Bali Y, Singh R, Gill T, Rela R, Vatsa R, Priyadarshni P. Bone graft and intraosseous anchorage of dental implants for reconstruction of the residual alveolar ridge. Journal of Pharmacy and Bioallied Sciences. 2021;13(5):S465-S8. | Not relevant to orthodontics |
| 57 | Ballastreire MCFF. Avaliação morfofuncional em pacientes adultos com perdas dentárias posteriores. 2012. p. 138-. | Not relevant to orthodontics |
| 58 | Barker DK. Occlusal interferences and temporomandibular dysfunction. General Dentistry. 2004;52(1):56-61. | Not relevant to orthodontics |
| 59 | Barnett PL, Lee MH, Oh L, Cull G, Babl F. Functional outcome after air-stirrup ankle brace or fiberglass backslab for pediatric low-risk ankle fractures: a randomized observer-blinded controlled trial. Pediatr Emerg Care. 2012;28(8):745-9. | Not relevant to orthodontics |
| 60 | Barrington J, Hage WD. Lateral epicondylitis (tennis elbow): Nonoperative, open, or arthroscopic treatment? Current Opinion in Orthopaedics. 2003;14(4):291-5. | Not relevant to orthodontics |
| 61 | Bateman M, Saunders B, Littlewood C, Davis D, Beckhelling J, Cooper K, et al. Comparing an optimised physiotherapy treatment package with usual physiotherapy care for people with tennis elbow — protocol for the OPTimisE pilot and feasibility randomised controlled trial. Pilot and Feasibility Studies. 2022;8(1). | Not relevant to orthodontics |
| 62 | Batista Jr EL, Batista FC, Novaes Jr AB. Management of soft tissue ridge deformities with acellular dermal matrix. Clinical approach and outcome after 6 months of treatment. Journal of Periodontology. 2001;72(2):265-73. | Not relevant to orthodontics |
| 63 | Battecha KH, Kamel DM, Tantawy SA. Investigating the effectiveness of adding microcurrent therapy to a traditional treatment program in myofascial pain syndrome in terms of neck pain and function. Physiotherapy Quarterly. 2021;29(1):17-23. | Not relevant to orthodontics |
| 64 | Behbehani F, Al-Aryan H, Al-Attar A, Al-Hamad N. Perceived effectiveness and side effects of intermaxillary fixation for diet control. International Journal of Oral and Maxillofacial Surgery. 2006;35(7):618-23. | Not relevant to orthodontics |
| 65 | Behr M, Stebner K, Kolbeck C, Faltermeier A, Driemel O, Handel G. Outcomes of temporomandibular joint disorder therapy: Observations over 13 years. Acta Odontologica Scandinavica. 2007;65(5):249-53. | Not relevant to orthodontics |
| 66 | Belzile EL, Côté M, Jacob MJ. The effects of combining viscosupplementation and knee bracing on pain reduction and increased function in patients with medial knee osteoarthritis. Clinical Journal of Sport Medicine. 2014;24(3):e49. | Not relevant to orthodontics |
| 67 | Bensaha T. A new approach for the surgical exposure of impacted canines by ultrasonic surgery through soft tissue. International Journal of Oral and Maxillofacial Surgery. 2013;42(12):1557-61. | Not relevant to orthodontics |
| 68 | Besch S, Dupré JP, Rodineau J, Dupuis JP, Saïdi K, Luciani JF, et al. Lateral ankle sprain. Multicenter study of a new concept ankle orthosis: Splint A2T. Journal de Traumatologie du Sport. 2016;33(4):198-208. | Not relevant to orthodontics |
| 69 | Bhargava D, Thomas S, Beena S. Comparison Between Efficacy of Transdermal Ketoprofen and Diclofenac Patch in Patients Undergoing Therapeutic Extraction-A Randomized Prospective Split Mouth Study. J Oral Maxillofac Surg. 2019;77(10):1998-2003. | Not relevant to orthodontics |
| 70 | Bhatia S, Karvannan H, Prem V. The effect of bio psychosocial model of rehabilitation on pain and quality of life after total knee replacement: A randomized controlled trial. Journal of Arthroscopy and Joint Surgery. 2020;7(4):177-83. | Not relevant to orthodontics |
| 71 | Bhave A, Mont M, Chugthai M, Starr R. Clinical and gait outcomes of novel pneumatic knee brace with extension assist. Osteoarthritis and Cartilage. 2017;25:S395-S6. | Not relevant to orthodontics |
| 72 | Bhayat A, Cleaton-Jones P. Dental clinic attendance in Soweto, South Africa, before and after the introduction of free primary dental health services. Community Dentistry and Oral Epidemiology. 2003;31(2):105-10. | Not relevant to orthodontics |
| 73 | Bisset LM, Collins NJ, Offord SS. Immediate effects of 2 types of braces on pain and grip strength in people with lateral epicondylalgia: a randomized controlled trial. J Orthop Sports Phys Ther. 2014;44(2):120-8. | Not relevant to orthodontics |
| 74 | Bitar AC, Demange MK, D'Elia CO, Camanho GL. Traumatic patellar dislocation: nonoperative treatment compared with MPFL reconstruction using patellar tendon. Am J Sports Med. 2012;40(1):114-22. | Not relevant to orthodontics |

| | | |
|---|---|---|
| 75 | Bjorne A, Agerberg G. Symptom relief after treatment of temporomandibular and cervical spine disorders in patients with Meniere's disease: A three-year follow-up. Cranio - Journal of Craniomandibular and Sleep Practice. 2003;21(1):50-60. | Not relevant to orthodontics |
| 76 | Blinkhorn A, Zadeh-Kabir R. Dental care of a child in pain - A comparison of treatment planning options offered by GDPs in California and the North-west of England. International Journal of Paediatric Dentistry. 2003;13(3):165-71. | Not relevant to orthodontics |
| 77 | Bonato LL, Quinelato V, Borojevic R, Vieira AR, Modesto A, Granjeiro JM, et al. Haplotypes of the RANK and OPG genes are associated with chronic arthralgia in individuals with and without temporomandibular disorders. Int J Oral Maxillofac Surg. 2017;46(9):1121-9. | Not relevant to orthodontics |
| 78 | Bonin FA, Silva RDd, Shimizu RH, Shimizu IA. Avaliação da percepção e da satisfação estética de pacientes que foram tratados com toxina botulínica tipo A para correção do sorriso gengival. Full dent sci. 2019;10(39):150-4. | Not relevant to orthodontics |
| 79 | Borchgrevink GE, Kaasa A, McDonagh D, Stiles TC, Haraldseth O, Lereim I. Acute treatment of whiplash neck sprain injuries. A randomized trial of treatment during the first 14 days after a car accident. Spine (Phila Pa 1976). 1998;23(1):25-31. | Not relevant to orthodontics |
| 80 | Bowen RL, Rupp NW, Eichmiller FC, Stanley HR. Clinical biocompatibility of an experimental dentine-enamel adhesive for composites. International dental journal. 1989;39(4):247-52. | Not relevant to orthodontics |
| 81 | Boyce SH, Quigley MA, Campbell S. Management of ankle sprains: a randomised controlled trial of the treatment of inversion injuries using an elastic support bandage or an Aircast ankle brace. Br J Sports Med. 2005;39(2):91-6. | Not relevant to orthodontics |
| 82 | Boyd R. Lessons learnt from RCTs of interventions for severe cerebral palsy. Developmental Medicine and Child Neurology. 2013;55:6-7. | Not relevant to orthodontics |
| 83 | Brandsson S, Faxén E, Kartus J, Eriksson BI, Karlsson J. Is a knee brace advantageous after anterior cruciate ligament surgery? A prospective, randomised study with a two-year follow-up. Scand J Med Sci Sports. 2001;11(2):110-4. | Not relevant to orthodontics |
| 84 | Brink O, Staunstrup H, Sommer J. Stable lateral malleolar fractures treated with aircast ankle brace and DonJoy R.O.M.-Walker brace: a prospective randomized study. Foot Ankle Int. 1996;17(11):679-84. | Not relevant to orthodontics |
| 85 | Brito LdS, Carinhenas CF. Estudo do índice de Disfunção Temporomandibular (DTM) em pacientes da Clínica Infantil da Universidade Cidade de São Paulo. Rev odontol Univ Cid São Paulo (Online). 2010;22(1):12-8. | Not relevant to orthodontics |
| 86 | Brouwer RW, van Raaij TM, Verhaar JA, Coene LN, Bierma-Zeinstra SM. Brace treatment for osteoarthritis of the knee: a prospective randomized multi-centre trial. Osteoarthritis Cartilage. 2006;14(8):777-83. | Not relevant to orthodontics |
| 87 | Brumini C, Natour J, Miura LY, Jones A. Effectiveness of bracing in elderly with knee osteoarthritis: A randomized controlled trial. Annals of the Rheumatic Diseases. 2015;74:1311. | Not relevant to orthodontics |
| 88 | Bucci R, Koutris M, Simeon V, Lobbezoo F, Michelotti A. Effects of acute pain and strain of the periodontium due to orthodontic separation on the occlusal tactile acuity of healthy individuals. Clin Oral Investig. 2021;25(12):6833-40. | Not relevant to orthodontics |
| 89 | Bukhari OM. Dental Caries Experience and Oral Health Related Quality of Life in Working Adults. Saudi Dental Journal. 2020;32(8):382-9. | Not relevant to orthodontics |
| 90 | Burkus JK, Gornet MF, Dickman CA, Zdeblick TA. Anterior lumbar interbody fusion using rhBMP-2 with tapered interbody cages. Journal of Spinal Disorders. 2002;15(5):337-49. | Not relevant to orthodontics |
| 91 | Cacho A, Tordera C, Colmenero C. Use of Transcutaneous Electrical Nerve Stimulation (TENS) for the Recovery of Oral Function after Orthognathic Surgery. Journal of Clinical Medicine. 2022;11(12). | Not relevant to orthodontics |
| 92 | Caglayan F, Altun O, Miloglu O, Kaya MD, Yilmaz AB. Correlation between oral health-related quality of life (OHQoL) and oral disorders in a Turkish patient population. Medicina Oral, Patologia Oral y Cirugia Bucal. 2009;14(11):e573-e8. | Not relevant to orthodontics |
| 93 | Cagnin A, Choinière M, Bureau NJ, Durand M, Mezghani N, Gaudreault N, et al. Impact of a personalized care approach on 3D gait impairments in knee osteoarthritis patients (a cluster randomized controlled trial). Osteoarthritis and Cartilage. 2020;28:S25. | Not relevant to orthodontics |
| 94 | Cagnin A, Choinière M, Bureau NJ, Eriau N, Hagemeister N. Flexion contracture in knee osteoarthritis patients is not indicative of flexion/extension impairments during gait. Osteoarthritis and Cartilage. 2020;28:S241-S2. | Not relevant to orthodontics |
| 95 | Cai H, Xi P, Zhong L, Chen J, Liang X. Efficacy of aromatherapy on dental anxiety: A systematic review of randomised and quasi-randomised controlled trials. Oral Diseases. 2021;27(4):829-47. | Not relevant to orthodontics |
| 96 | Caldas W, Furquim BDA, Andrighetto AR. Relação entre DTM e tratamento ortodôntico na visão de clínicos gerais, especialistas em Ortodontia e especialistas em Disfunção Temporomandibular e Dor Orofacial. Rev Clín Ortod Dent Press. 2013;11(6):20-6. | Not relevant to orthodontics |
| 97 | Callaghan MJ, Parkes MJ, Felson DT. The Effect of Knee Braces on Quadriceps Strength and Inhibition in Subjects With Patellofemoral Osteoarthritis. J Orthop Sports Phys Ther. 2016;46(1):19-25. | Not relevant to orthodontics |
| 98 | Callaghan MJ, Parkes MJ, Hutchinson CE, Gait AD, Forsythe LM, Marjanovic EJ, et al. A randomised trial of a brace for patellofemoral osteoarthritis targeting knee pain and bone marrow lesions. Ann Rheum Dis. 2015;74(6):1164-70. | Not relevant to orthodontics |
| 99 | Calmels P, Queneau P, Hamonet C, Le Pen C, Maurel F, Lerouvreur C, et al. Effectiveness of a lumbar belt in subacute low back pain: an open, multicentric, and randomized clinical study. Spine (Phila Pa 1976). 2009;34(3):215-20. | Not relevant to orthodontics |
| 100 | Campbell MJ, Carreon LY, Traynelis V, Anderson PA. Use of cervical collar after single-level anterior cervical fusion with plate: is it necessary? Spine (Phila Pa 1976). 2009;34(1):43-8. | Not relevant to orthodontics |
| 101 | Carlson CR, Bertrand PM, Dale Ehrlich A, Maxwell AW, Burton RG. Physical Self-Regulation Training for the Management of Temporomandibular Disorders. Journal of Orofacial Pain. 2001;15(1):47-55. | Not relevant to orthodontics |
| 102 | Carter N, Asadi H, Kok H, Maingard J, Anselmetti G, Chandra R, et al. Using artificial intelligence to predict vertebroplasty outcome. Journal of Medical Imaging and Radiation Oncology. 2018;62:22-3. | Not relevant to orthodontics |
| 103 | Carvalho RTd, Braga FSFF, Brito F, Capelli Junior J, Figueredo CM, Sztajnbok FR. Alterações da articulação temporomandibular e suas repercussões orofaciais em pacientes portadores de artrite idiopática juvenil. Rev bras reumatol. 2012;52(6):907-11. | Not relevant to orthodontics |
| 104 | Cavallo P, Savarese G, Carpinelli L. Bruxism and health related quality of life in Southern Italy's prison inmates. Community Dental Health. 2014;31(2):117-22. | Not relevant to orthodontics |
| 105 | Cavassaki I, Suazo Galdames I, Guimarães AS. Comfort evaluation of non occlusal intraoral appliance use during sleep in patients with muscular temporomandibular dysfunction. Int j odontostomatol (Print). 2013;7(3):351-7. | Not relevant to orthodontics |
| 106 | Caviedes-Bucheli J, Moreno JO, Carreno CP, Delgado R, Garcia DJ, Solano J, et al. The effect of single-file reciprocating systems on Substance P and Calcitonin gene-related peptide expression in human periodontal ligament. International Endodontic Journal. 2013;46(5):419-26. | Not relevant to orthodontics |
| 107 | Cavina SR, Vedovello SAS, Dos Santos PR, Carneiro DPA, Venezian GC, CustÓDio W, et al. Affective relationships as predictors of TMD symptoms in young adults. Rev odontol UNESP (Online). 2021;50:e20210018-e. | Not relevant to orthodontics |
| 108 | Cecchetti S, Pereira B, Roche A, Deschaumes C, Abdi D, Coudeyre E, et al. Efficacy and safety of pamidronate in Modic type 1 changes: study protocol for a prospective randomized controlled clinical trial. Trials. 2014;15:117. | Not relevant to orthodontics |
| 109 | Celestini M, Marchese A, Serenelli A, Graziani G. A randomized controlled trial on the efficacy of physical exercise in patients braced for instability of the lumbar spine. Eura Medicophys. 2005;41(3):223-31. | Not relevant to orthodontics |
| 110 | Cen X, Liu Y, Wang S, Yang X, Shi Z, Liang X. Glucosamine oral administration as an adjunct to hyaluronic acid injection in treating temporomandibular joint osteoarthritis. Oral Dis. 2018;24(3):404-11. | Not relevant to orthodontics |
| 111 | Ceneviz C, Mehta NR, Forgione A, Sands MJ, Abdallah EF, Lobo Lobo S, et al. The immediate effect of changing mandibular position on the EMG activity of the masseter, temporalis, sternocleidomastoid, and trapezius muscles. Cranio. 2006;24(4):237-44. | Not relevant to orthodontics |
| 112 | Cepeda MS, Carr DB, Sarquis T, Miranda N, Garcia RJ, Zarate C. Static magnetic therapy does not decrease pain or opioid requirements: a randomized double-blind trial. Anesth Analg. 2007;104(2):290-4. | Not relevant to orthodontics |
| 113 | Chan A, Armati P, Moorthy AP. Pulsed Nd: YAG laser induces pulpal analgesia: a randomized clinical trial. J Dent Res. 2012;91(7 Suppl):79s-84s. | Not relevant to orthodontics |
| 114 | Chan HL, Ng GY. Effect of counterforce forearm bracing on wrist extensor muscles performance. Am J Phys Med Rehabil. 2003;82(4):290-5. | Not relevant to orthodontics |
| 115 | Chandu A, Suvinen TI, Reade PC, Borromeo GL. The effect of an interocclusal appliance on bite force and masseter electromyography in asymptomatic subjects and patients with temporomandibular pain and dysfunction. Journal of Oral Rehabilitation. 2004;31(6):530-7. | Not relevant to orthodontics |
| 116 | Chaushu G, Becker A, Zeltser R, Vasker N, Branski S, Chaushu S. Patients' perceptions of recovery after routine extraction of healthy premolars. American Journal of Orthodontics and Dentofacial Orthopedics. 2007;131(2):170-5. | Not relevant to orthodontics |
| 117 | Chen CF, Hu CC, Wu CT, Wu HH, Chang CS, Hung YP, et al. Treatment of knee osteoarthritis with intra-articular injection of allogeneic adipose-derived stem cells (ADSCs) ELIXCYTE®: a phase I/II, randomized, active-control, single-blind, multiple-center clinical trial. Stem Cell Res Ther. 2021;12(1):562. | Not relevant to orthodontics |
| 118 | Chen M, Li P, Lin F. Influence of structured telephone follow-up on patient compliance with rehabilitation after total knee arthroplasty. Patient Preference and Adherence. 2016;10:257-64. | Not relevant to orthodontics |
| 119 | Chen W, Sun J, Zhao Y, Song M, Xue Z, Chen Q, et al. Topical Chinese patent medicine for knee osteoarthritis pain. Osteoarthritis and Cartilage. 2021;29:S267. | Not relevant to orthodontics |
| 120 | Cherian JJ, Bhave A, Kapadia BH, Starr R, McElroy MJ, Mont MA. Strength and Functional Improvement Using Pneumatic Brace with Extension Assist for End-Stage Knee Osteoarthritis: A Prospective, Randomized trial. J Arthroplasty. 2015;30(5):747-53. | Not relevant to orthodontics |
| 121 | Cherian JJ, Kapadia BH, Bhave A, McElroy MJ, Cherian C, Harwin SF, et al. Use of Transcutaneous Electrical Nerve Stimulation Device in Early Osteoarthritis of the Knee. J Knee Surg. 2015;28(4):321-7. | Not relevant to orthodontics |
| 122 | Choi KH, Kwon OS, Jerng UM, Lee SM, Kim LH, Jung J. Development of electromyographic indicators for the diagnosis of temporomandibular disorders: a protocol for an assessor-blinded cross-sectional study. Integrative Medicine Research. 2017;6(1):97-104. | Not relevant to orthodontics |
| 123 | Chompu-Inwai P, Simprasert S, Chuveera P, Nirunsittirat A, Sastraruji T, Srisuwan T. Effect of Nitrous Oxide on Pulpal Anesthesia: A Preliminary Study. Anesth Prog. 2018;65(3):156-61. | Not relevant to orthodontics |
| 124 | Christanell F, Hoser C, Huber R, Fink C, Luomajoki H. The influence of electromyographic biofeedback therapy on knee extension following anterior cruciate ligament reconstruction: A randomized controlled trial. Sports Medicine, Arthroscopy, Rehabilitation, Therapy and Technology. 2012;4(1). | Not relevant to orthodontics |
| 125 | Christidis N, Doepel M, Ekberg E, Ernberg M, Le Bell Y, Nilner M. Effectiveness of a prefabricated occlusal appliance in patients with temporomandibular joint pain: a randomized controlled multicenter study. J Oral Facial Pain Headache. 2014;28(2):128-37. | Not relevant to orthodontics |
| 126 | Christidis N, Smedberg E, Hägglund H, Hedenberg-Magnusson B. Patients' experience of care and treatment outcome at the department of clinical oral physiology, dental public service in stockholm. Swedish Dental Journal. 2010;34(1):43-50. | Not relevant to orthodontics |
| 127 | Christovam ERP, Boeck EM, Vedovello SAS, Valdrighi HC, Degan VV, Vedovello Filho M. Signs and symptoms of temporomandibular disorder and patients' satisfaction before and after orthognathic surgery. Braz j oral sci. 2016;15(2):137-43. | Not relevant to orthodontics |
| 128 | Chuang SH, Huang MH, Chen TW, Weng MC, Liu CW, Chen CH. Effect of knee sleeve on static and dynamic balance in patients with knee osteoarthritis. Kaohsiung J Med Sci. 2007;23(8):405-11. | Not relevant to orthodontics |
| 129 | Chughtai M, Bhave A, Khan SZ, Khlopas A, Ali O, Harwin SF, et al. Clinical Outcomes of a Pneumatic Unloader Brace for Kellgren-Lawrence Grades 3 to 4 Osteoarthritis: A Minimum 1-Year Follow-Up Study. J Knee Surg. 2016;29(8):634-8. | Not relevant to orthodontics |
| 130 | Cimino R, Farella M, Michelotti A, Pugliese R, Martina R. Does the ovarian cycle influence the pressure-pain threshold of the masticatory muscles in symptom-free women? J Orofac Pain. 2000;14(2):105-11. | Not relevant to orthodontics |
| 131 | Cohen D. Balloon kyphoplasty was effective and safe for vertebral compression fractures compared with nonsurgical care: Commentary. Journal of Bone and Joint Surgery. 2009;91(11):2747. | Not relevant to orthodontics |
| 132 | Conti M, Garofalo R, Castagna A. Does a brace influence clinical outcomes after arthroscopic rotator cuff repair? Musculoskelet Surg. 2015;99 Suppl 1:S31-5. | Not relevant to orthodontics |
| 133 | Conti PC, de Alencar EN, da Mota Corrêa AS, Lauris JR, Porporatti AL, Costa YM. Behavioural changes and occlusal splints are effective in the management of masticatory myofascial pain: a short-term evaluation. J Oral Rehabil. 2012;39(10):754-60. | Not relevant to orthodontics |
| 134 | Conti PCR, CorrÊA ASdM, Lauris JRP, Stuginski-Barbosa J. Management of painful temporomandibular joint clicking with different intraoral devices and counseling: a controlled study. J appl oral sci. 2015;23(5):529-35. | Not relevant to orthodontics |
| 135 | Cooke MW, Marsh JL, Clark M, Nakash R, Jarvis RM, Hutton JL, et al. Treatment of severe ankle sprain: a pragmatic randomised controlled trial comparing the clinical effectiveness and cost-effectiveness of three types of mechanical ankle support with tubular bandage. The CAST trial. Health Technol Assess. 2009;13(13):iii, ix-x, 1-121. | Not relevant to orthodontics |
| 136 | Corrigan P, Felson DT, Lewis CL, Gross KD, Nevitt MC, Lewis CE, et al. In those with unilateral frequent knee pain, between-limb differences in stance time during walking increase the risk of pain in the other knee: the most study. Osteoarthritis and Cartilage. 2020;28:S56-S7. | Not relevant to orthodontics |
| 137 | Crawford JR, Khan RJ, Varley GW. Early management and outcome following soft tissue injuries of the neck-a randomised controlled trial. Injury. 2004;35(9):891-5. | Not relevant to orthodontics |
| 138 | Cudejko T, van der Esch M, van den Noort JC, Rijnhart JJM, van der Leeden M, Roorda LD, et al. Decreased Pain and Improved Dynamic Knee Instability Mediate the Beneficial Effect of Wearing a Soft Knee Brace on Activity Limitations in Patients With Knee Osteoarthritis. Arthritis Care Res (Hoboken). 2019;71(8):1036-43. | Not relevant to orthodontics |
| 139 | Cudejko T, Van Der Esch M, Van Der Leeden M, Douw-Van Den Noort J, Roorda L, Lems W, et al. The effect of soft brace on self-reported knee instability and confidence, pain and physical function in patients with knee osteoarthritis. Osteoarthritis and Cartilage. 2017;25:S392-S3. | Not relevant to orthodontics |
| 140 | da Silva GSQ, Raggio DP, Machado GFR, Mello-Moura ACV, Gimenez T, Floriano I, et al. Impact of different restorative treatments for deep caries lesion in primary teeth (CEPECO 1) - study protocol for a noninferiority randomized clinical trial. BMC Oral Health. 2019;19(1):6. | Not relevant to orthodontics |
| 141 | da Silva Neves FL, Silveira CA, Dias SB, Santamaria Júnior M, de Marco AC, Kerbauy WD, et al. Comparison of two power densities on the healing of palatal wounds after connective tissue graft removal: randomized clinical trial. Lasers Med Sci. 2016;31(7):1371-8. | Not relevant to orthodontics |
| 142 | da Silveira GE, Andrade RM, Guilhermino GG, Schmidt AV, Neves LM, Ribeiro AP. The Effects of Short- and Long-Term Spinal Brace Use with and without Exercise on Spine, Balance, and Gait in Adolescents with Idiopathic Scoliosis. Medicina (Kaunas). 2022;58(8). | Not relevant to orthodontics |
| 143 | Dahl BL, Krogstad BS, øgaard B, Eckersberg T. Differences in functional variables, fillings, and tooth wear in two groups of 19-year-old individuals. Acta Odontologica Scandinavica. 1989;47(1):35-40. | Not relevant to orthodontics |
| 144 | Dahl BL, Krogstad BS, øgaard B, Eckersberg T. Signs and symptoms of craniomandibular disorders in two groups of 19-year-old individuals, one treated orthodontically and the other not. Acta Odontologica Scandinavica. 1988;46(2):89-93. | Not relevant to orthodontics |
| 145 | Dalichau S, Scheele K. [Effects of elastic lumbar belts on the effect of a muscle training program for patients with chronic back pain]. Z Orthop Ihre Grenzgeb. 2000;138(1):8-16. | Not relevant to orthodontics |
| 146 | Davies M, Dorenbaum A, Wang S, Mittendorfer B. REN001, PPARδ Agonist, Preserves Muscle Strength and Promotes Recovery of Muscle Atrophy After Leg Immobilization. Journal of Neuromuscular Diseases. 2022;9:S129-S30. | Not relevant to orthodontics |
| 147 | Day JM, Lucado AM, Dale RB, Merriman H, Marker CD, Uhl TL. The Effect of Scapular Muscle Strengthening on Functional Recovery in Patients With Lateral Elbow Tendinopathy: A Pilot Randomized Controlled Trial. J Sport Rehabil. 2021;30(5):744-53. | Not relevant to orthodontics |
| 148 | De Angelis MV, Pierfelice F, Di Giovanni P, Staniscia T, Uncini A. Efficacy of a soft hand brace and a wrist splint for carpal tunnel syndrome: a randomized controlled study. Acta Neurol Scand. 2009;119(1):68-74. | Not relevant to orthodontics |

| | | |
|---|---|---|
| 149 | De Boever JA, Van Den Berghe L, De Boever AL, Keersmaekers K. Comparison of clinical profiles and treatment outcomes of an elderly and a younger temporomandibular patient group. The Journal of prosthetic dentistry. 1999;81(3):312-7. | Not relevant to orthodontics |
| 150 | De Giorgi I, Castroflorio T, Sartoris B, Deregibus A. The use of conventional transcutaneous electrical nerve stimulation in chronic facial myalgia patients. Clin Oral Investig. 2017;21(1):275-80. | Not relevant to orthodontics |
| 151 | de Haan J, den Hartog D, Tuinebreijer WE, Iordens GI, Breederveld RS, Bronkhorst MW, et al. Functional treatment versus plaster for simple elbow dislocations (FuncSiE): a randomized trial. BMC Musculoskelet Disord. 2010;11:263. | Not relevant to orthodontics |
| 152 | de Vries A, Zwerver J, Diercks R, Tak I, van Berkel S, van Cingel R, et al. Effect of patellar strap and sports tape on pain in patellar tendinopathy: A randomized controlled trial. Scand J Med Sci Sports. 2016;26(10):1217-24. | Not relevant to orthodontics |
| 153 | Della Croce U, Crapanzano F, Li L, Kasi PK, Patritti BL, Mancinelli C, et al. A preliminary assessment of a novel pneumatic unloading knee brace on the gait mechanics of patients with knee osteoarthritis. Pm r. 2013;5(10):816-24. | Not relevant to orthodontics |
| 154 | Denton J, Willson JD, Ballantyne BT, Davis IS. The addition of the Protonics brace system to a rehabilitation protocol to address patellofemoral joint syndrome. J Orthop Sports Phys Ther. 2005;35(4):210-9. | Not relevant to orthodontics |
| 155 | Dessery Y, Belzile EL, Turmel S, Corbeil P. Comparison of three knee braces in the treatment of medial knee osteoarthritis. Knee. 2014;21(6):1107-14. | Not relevant to orthodontics |
| 156 | Devi J, Verma M, Gupta R. Assessment of treatment response to splint therapy and evaluation of TMJ function using joint vibration analysis in patients exhibiting TMJ disc displacement with reduction: A clinical study. Indian J Dent Res. 2017;28(1):33-43. | Not relevant to orthodontics |
| 157 | Diaz A, Morisset C, Fournel I, Soilly AL, Bussiere C, Cherasse A, et al. Efficacy and safety of a distraction-rotation knee brace (ODRA) in medial knee osteoarthritis-a phase iii randomised controlled trial (ergonomie study). Annals of the Rheumatic Diseases. 2018;77:79. | Not relevant to orthodontics |
| 158 | Dib A, Montero J, Sanchez JM, López-Valverde A. Electromyographic and patient-reported outcomes of a computer-guided occlusal adjustment performed on patients suffering from chronic myofascial pain. Medicina Oral, Patologia Oral y Cirugia Bucal. 2015;20(2):e135-e43. | Not relevant to orthodontics |
| 159 | Dickinson BP, Ashley RK, Wasson KL, O'Hara C, Gabbay J, Heller JB, et al. Reduced morbidity and improved healing with bone morphogenic protein-2 in older patients with alveolar cleft defects. Plastic and Reconstructive Surgery. 2008;121(1):209-17. | Not relevant to orthodontics |
| 160 | Diernberger S, Bernhardt O, Schwahn C, Kordass B. Self-reported chewing side preference and its associations with occlusal, temporomandibular and prosthodontic factors: results from the population-based Study of Health in Pomerania (SHIP-0). J Oral Rehabil. 2008;35(8):613-20. | Not relevant to orthodontics |
| 161 | Dieter V, Haupt G, Janssen P, Krauss I. RANDOMIZED-CONTROLLED TRIAL TO EVALUATE A 12-WEEK APP- AND BRACE-ASSISTED EXERCISE INTERVENTION IN PATIENTS WITH KNEE OSTEOARTHRITIS. Osteoarthritis and Cartilage. 2022;30:S401-S2. | Not relevant to orthodontics |
| 162 | Diwan A, Purohit S. A Comparative Study of Orotracheal Intubation Guided by Airtraq and McCoy Laryngoscope in Patients with Rigid Cervical Collar In-situ Simulating Cervical Immobilization for Traumatic Cervical Spine Injury. Indian Journal of Neurosurgery. 2019;8(3):161-7. | Not relevant to orthodontics |
| 163 | Doepel M, Nilner M, Ekberg E, Vahlberg T, Bell Y. Headache: short- and long-term effectiveness of a prefabricated appliance compared to a stabilization appliance. Acta Odontol Scand. 2011;69(3):129-36. | Not relevant to orthodontics |
| 164 | Doepel M, Nilner M, Vahlberg T, Le Bell Y. Similar treatment outcome in myofascial TMD patients with localized and widespread pain. Acta Odontol Scand. 2018;76(3):175-82. | Not relevant to orthodontics |
| 165 | Dolan L, Weinstein S. To BrAIST or not to BrAIST: Decisions and characteristics of 1131 patients eligible for the Bracing in Adolescent Idiopathic Scoliosis Trial. Scoliosis. 2012;7. | Not relevant to orthodontics |
| 166 | Dolwick MF, Diaz D, Freburg-Hoffmeister DL, Widmer CG. A Randomized, Double-Blind, Placebo-Controlled Study of the Efficacy of Steroid Supplementation After Temporomandibular Joint Arthrocentesis. J Oral Maxillofac Surg. 2020;78(7):1088-99. | Not relevant to orthodontics |
| 167 | Donnell A, T DN, Lawrence M, Gupta V, Zieba T, Truong DQ, et al. High-Definition and Non-invasive Brain Modulation of Pain and Motor Dysfunction in Chronic TMD. Brain Stimul. 2015;8(6):1085-92. | Not relevant to orthodontics |
| 168 | Draganich L, Reider B, Rimington T, Piotrowski G, Mallik K, Nasson S. The effectiveness of self-adjustable custom and off-the-shelf bracing in the treatment of varus gonarthrosis. J Bone Joint Surg Am. 2006;88(12):2645-52. | Not relevant to orthodontics |
| 169 | Du S, Wei L, He B, Fang Z, Zhou E, Ma X, et al. Dynamic fixation using rigid tape in rehabilitation after surgery of terrible triad injury of the elbow: A randomized trial. J Back Musculoskelet Rehabil. 2021;34(6):957-64. | Not relevant to orthodontics |
| 170 | Duetzmann S, Cole T, Senft C, Seifert V, Ratliff JK, Park J. Clavicle pain and reduction of incisional and fascial pain after posterior cervical surgery. J Neurosurg Spine. 2015;23(6):684-9. | Not relevant to orthodontics |
| 171 | Duruk G, Akküç S, Gümü, bo, a Z, eyma. Children's Primary Dental Treatment Needs According to Their Parents. Pesqui bras odontopediatria clín integr. 2021;21:e0201-e. | Not relevant to orthodontics |
| 172 | Dwarakanathan R, Mohanty RK, Sahoo S, Prasad S. Efficacy of unloader knee orthosis and lateral wedge insole on static balance in medial knee osteoarthritis. Journal of Orthopaedics, Trauma and Rehabilitation. 2022;29(1). | Not relevant to orthodontics |
| 173 | Ege B, Ege M, Koparal M, Alan H. Comparison of the Anesthetic Efficiency of Lidocaine and Tramadol Hydrochloride in Orthodontic Extractions: A Split-Mouth, Prospective, Randomized, Double-Blind Study. J Oral Maxillofac Surg. 2020;78(1):52-62. | Not relevant to orthodontics |
| 174 | Egermark-Eriksson I, Carlsson GE, Ingervall B. Prevalence of mandibular dysfunction and orofacial parafunction in 7-, 11- and 15-year-old swedish children. European Journal of Orthodontics. 1981;3(3):163-72. | Not relevant to orthodontics |
| 175 | Eiff MP, Smith AT, Smith GE. Early mobilization versus immobilization in the treatment of lateral ankle sprains. Am J Sports Med. 1994;22(1):83-8. | Not relevant to orthodontics |
| 176 | El-Ansary D, Waddington G, Adams R. Control of separation in sternal instability by supportive devices: a comparison of an adjustable fastening brace, compression garment, and sports tape. Arch Phys Med Rehabil. 2008;89(9):1775-81. | Not relevant to orthodontics |
| 177 | Ellis PE, Bradley RL, Sandy JR, Deacon SA, Griffiths HS, Atack NE, et al. Do I have enough time? The impact of recruiting patients to a randomised controlled trial at recruiting centres. Br Dent J. 2012;213(9):467-70. | Not relevant to orthodontics |
| 178 | Eshghpour M, Ahrari F, Takallu M. Is Low-Level Laser Therapy Effective in the Management of Pain and Swelling After Mandibular Third Molar Surgery? J Oral Maxillofac Surg. 2016;74(7):1322.e1-8. | Not relevant to orthodontics |
| 179 | Espinosa Alba F, Avalos Equihua YM, Martínez Cardona PC, Hernández Bozada M, Sayavedra Jaimes J, Varela Flores JS, et al. Ameloplastia positiva en relación céntrica como método auxiliar durante la finalización en el tratamiento de ortodoncia en un paciente bruxista. Rev ADM. 2020;77(4):209-15. | Not relevant to orthodontics |
| 180 | Evcik D, Kuru I, Ay S, Maralcan G. Home-based exercise and patellar bracig in the treatment of patellofemoral pain syndrome. Turkiye Fiziksel Tip ve Rehabilitasyon Dergisi. 2010;56(3):100-4. | Not relevant to orthodontics |
| 181 | Ey-Chmielewska H. [An attempt to use ultrasonic technique for confirming the diagnosis, planning and observation of long-term treatment results of painful temporo-mandibular joint dysfunction]. Annales Academiae Medicae Stetinensis. 1998;44:223-36. | Not relevant to orthodontics |
| 182 | Eyles JP, Murphy NJ, Virk S, Spiers L, Molnar R, O'Donnell J, et al. Can a Hip Brace Improve Short-Term Hip-Related Quality of Life for People With Femoroacetabular Impingement and Acetabular Labral Tears: An Exploratory Randomized Trial. Clin J Sport Med. 2022;32(3):e243-e50. | Not relevant to orthodontics |
| 183 | Faes M, van den Akker B, de Lint JA, Kooloos JG, Hopman MT. Dynamic extensor brace for lateral epicondylitis. Clin Orthop Relat Res. 2006;442:149-57. | Not relevant to orthodontics |
| 184 | Farag S, Rosen L, Ascher-Walsh C. Comparison of the Memory Foam Pad Versus the Bean Bag with Shoulder Braces in Preventing Patient Displacement during Gynecologic Laparoscopic Surgery. J Minim Invasive Gynecol. 2018;25(1):153-7. | Not relevant to orthodontics |
| 185 | Farella M, Bakke M, Michelotti A, Martina R. Effects of prolonged gum chewing on pain and fatigue in human jaw muscles. Eur J Oral Sci. 2001;109(2):81-5. | Not relevant to orthodontics |
| 186 | Farronato G, Maspero C, Russo E, Periti G, Farronato D. Headache and transverse maxillary discrepancy. Journal of Clinical Pediatric Dentistry. 2008;33(1):67-74. | Not relevant to orthodontics |
| 187 | Farronato G, Re D, Augusti G, Butti A, Augusti D. Biomimetic orthodontic treatments: Preferences of adult patients and analysis of the Willingness-To-Pay index. Dental Cadmos. 2016;84(7):408-17. | Not relevant to orthodontics |
| 188 | Fary R, Carroll GJ, Briffa TG, Briffa NK. Is pulsed electrical stimulation a viable treatment option in managing pain from osteoarthritis of the knee? Physiotherapy (United Kingdom). 2011;97:eS325-eS6. | Not relevant to orthodontics |
| 189 | Fary RE, Carroll G, Briffa TG, Briffa NK. Effectiveness of pulsed electrical stimulation in the treatment of osteoarthritis of the knee: A randomized controlled trial. Arthritis and Rheumatism. 2009;60:846. | Not relevant to orthodontics |
| 190 | Fasunloro A, Owotabe FJ. Occupational hazards among clinical dental staff. Journal of Contemporary Dental Practice. 2004;5(2):134-52. | Not relevant to orthodontics |
| 191 | Fayed MM, El-Mangoury NH, El-Bokle DN, Belal AI. Occlusal splint therapy and magnetic resonance imaging. World J Orthod. 2004;5(2):133-40. | Not relevant to orthodontics |
| 192 | Ferreira R, Cunha PdO, Karam PSBH, Sant'Ana ACP, Rezende MLRd, Greghi SLA, et al. Cimento cirúrgico periodontal: usar ou não usar? Perionews. 2014;8(3):251-5. | Not relevant to orthodontics |
| 193 | Filippi A, von Arx T, Lussi A. Comfort and discomfort of dental trauma splints - a comparison of a new device (TTS) with three commonly used splinting techniques. Dent Traumatol. 2002;18(5):275-80. | Not relevant to orthodontics |
| 194 | Finestone A, Radin EL, Lev B, Shlamkovitch N, Wiener M, Milgrom C. Treatment of overuse patellofemoral pain. Prospective randomized controlled clinical trial in a military setting. Clin Orthop Relat Res. 1993(293):208-10. | Not relevant to orthodontics |
| 195 | Finsterwald M, Dao Trong ML, Hollo D, Müller A, Riede U, Bouaicha S. Conservative treatment of Rockwood type III acromioclavicular joint dislocation with two different types of braces: A comparative prospective randomized trial (9894). Swiss Medical Weekly. 2021;151(SUPPL 250):7S. | Not relevant to orthodontics |
| 196 | Flack NA, Hay-Smith EJ, Stringer MD, Gray AR, Woodley SJ. Adherence, tolerance and effectiveness of two different pelvic support belts as a treatment for pregnancy-related symphyseal pain - a pilot randomized trial. BMC Pregnancy Childbirth. 2015;15:36. | Not relevant to orthodontics |
| 197 | Foley-Nolan D, Moore K, Codd M, Barry C, O'Connor P, Coughlan RJ. Low energy high frequency pulsed electromagnetic therapy for acute whiplash injuries. A double blind randomized controlled study. Scand J Rehabil Med. 1992;24(1):51-9. | Not relevant to orthodontics |
| 198 | Fontana CE, da Silveira Bueno CE, de Moura JDM, da Rocha Bastida Pinheiro G, Trevensoli VC, Pelegrine RA, et al. Influence of operator experience on apical debris extrusion after endodontic instrumentation with different single-file systems. Giornale Italiano di Endodonzia. 2022;36(1):151-8. | Not relevant to orthodontics |
| 199 | Fraccari F, Bogini A, Spangaro F. Use of distraction plates in the pathology of the temporo-mandibular articulation. Minerva stomatologica. 1989;38(3):345-51. | Not relevant to orthodontics |
| 200 | França DdSGd, Residência Multiprofissional eO. Terapia do espelho em pacientes amputados por câncer com dor e sensação de membro fantasma  estudo piloto. 2019. p. 40 f- f. | Not relevant to orthodontics |
| 201 | Fujimura K, Segami N, Sato J, Kanayama K, Nishimura M, Demura N. Advantages of intraoral verticosagittal ramus osteotomy in skeletofacial deformity patients with temporomandibular joint disorders. Journal of Oral and Maxillofacial Surgery. 2004;62(10):1246-52. | Not relevant to orthodontics |
| 202 | Fulling HJ, Andreasen JO. Influence of splints and temporary crowns upon electric and thermal pulp-testing procedures. European Journal of Oral Sciences. 1976;84(5):291-6. | Not relevant to orthodontics |
| 203 | Furukawa Y. Tasuki for neck pain: An individually-randomized, open-label, waiting-list-controlled trial. J Occup Health. 2020;62(1):e12097. | Not relevant to orthodontics |
| 204 | Garcia-Pola MJ, Garcia-Martin JM, Varela-Centelles P, Bilbao-Alonso A, Cerero-Lapiedra R, Seoane J. Oral and facial piercing: Associated complications and clinical repercussion. Quintessence International. 2008;39(1):51-9. | Not relevant to orthodontics |
| 205 | Garg R, Adamson GJ, Dawson PA, Shankwiler JA, Pink MM. A prospective randomized study comparing a forearm strap brace versus a wrist splint for the treatment of lateral epicondylitis. J Shoulder Elbow Surg. 2010;19(4):508-12. | Not relevant to orthodontics |
| 206 | Gawlak D, Mierzwińska-Nastalska E, Mańka-Malara K, Kamiński T. Assessment of custom and standard, self-adapted mouthguards in terms of comfort and users subjective impressions of their protective function. Dental Traumatology. 2015;31(2):113-7. | Not relevant to orthodontics |
| 207 | Gazal G, Alharbi R, Fareed WM, Omar E, Alolayan AB, Al-Zoubi H, et al. Comparison of onset anesthesia time and injection discomfort of 4% articaine and 2% mepivacaine during teeth extractions. Saudi Journal of Anaesthesia. 2017;11(2):152-7. | Not relevant to orthodontics |
| 208 | Gennis P, Miller L, Gallagher EJ, Giglio J, Carter W, Nathanson N. The effect of soft cervical collars on persistent neck pain in patients with whiplash injury. Acad Emerg Med. 1996;3(6):568-73. | Not relevant to orthodontics |
| 209 | Gerasimidou O, Watson T, Millar B. Effect of placing intentionally high restorations: Randomized clinical trial. J Dent. 2016;45:26-31. | Not relevant to orthodontics |
| 210 | Ghabraei S, Bolhari B, Nashtaie HM, Noruzian M, Niavarzi S, Chiniforush N. Effect of photobiomodulation on pain level during local anesthesia injection: a randomized clinical trial. J Cosmet Laser Ther. 2020;22(4-5):180-4. | Not relevant to orthodontics |
| 211 | Ghaderi F, Ahmadbeigi M, Vossoughi M, Sardarian A. The efficacy of administrating a sweet-tasting solution for reducing the pain related to dental injections in children: A randomized controlled trial. Int J Paediatr Dent. 2021;31(2):184-90. | Not relevant to orthodontics |
| 212 | Ghandour TM, Ibrahim A, Abdelrahman AA, Elgammal A, Hammad MH. Does The Type of Shoulder Brace Affect Postoperative Pain and Clinical Outcome After Arthroscopic Rotator Cuff Repair? Arthroscopy. 2019;35(4):1016-23. | Not relevant to orthodontics |
| 213 | Giannakopoulos NN, Katsikogianni EN, Hellmann D, Eberhard L, Leckel M, Schindler HJ, et al. Comparison of three different options for immediate treatment of painful temporomandibular disorders: a randomized, controlled pilot trial. Acta Odontol Scand. 2016;74(6):480-6. | Not relevant to orthodontics |
| 214 | Gilheaney Ó, Stassen LF, Walshe M. Prevalence, Nature, and Management of Oral Stage Dysphagia in Adults With Temporomandibular Joint Disorders: Findings From an Irish Cohort. Journal of Oral and Maxillofacial Surgery. 2018;76(8):1665-76. | Not relevant to orthodontics |
| 215 | Gimbel M, Ashley RK, Sisodia M, Gabbay JS, Wasson KL, Heller J, et al. Repair of alveolar cleft defects: Reduced morbidity with bone marrow stem cells in a resorbable matrix. Journal of Craniofacial Surgery. 2007;18(4):895-901. | Not relevant to orthodontics |
| 216 | Gomes CA, Politti F, Andrade DV, de Sousa DF, Herpich CM, Dibai-Filho AV, et al. Effects of massage therapy and occlusal splint therapy on mandibular range of motion in individuals with temporomandibular disorder: a randomized clinical trial. J Manipulative Physiol Ther. 2014;37(3):164-9. | Not relevant to orthodontics |
| 217 | Gómez ÁM, Valencia MA, Marín DA, Agudelo JA, Álvarez E, Medina ME. Influencia del rofecoxib en los niveles de pg-e2 e il-1B; el movimiento dentario y el dolor durante la retracción de caninos. CES odontol. 2003;16(2):15-25. | Not relevant to orthodontics |
| 218 | Gorlitzer M, Folkmann S, Meinhart J, Poslussny P, Thalmann M, Weiss G, et al. A newly designed thorax support vest prevents sternum instability after median sternotomy. Eur J Cardiothorac Surg. 2009;36(2):335-9; discussion 9. | Not relevant to orthodontics |
| 219 | Graham HK, Boyd R, Carlin JB, Dobson F, Lowe K, Nattrass G, et al. Does botulinum toxin a combined with bracing prevent hip displacement in children with cerebral palsy and "hips at risk"? A randomized, controlled trial. J Bone Joint Surg Am. 2008;90(1):23-33. | Not relevant to orthodontics |
| 220 | Grauers A, Topalis C, Möller H, Normelli H, Karlsson MK, Danielsson A, et al. Prevalence of back problems in 1069 adults with idiopathic scoliosis and 158 adults without scoliosis. Spine. 2014;39(11):886-92. | Not relevant to orthodontics |
| 221 | Griffin C, Faieta J, Persch A, Peters H, Page S. Portable, EMG-triggered, myoelectric bracing: Restoring arm use and participation in stroke. Archives of Physical Medicine and Rehabilitation. 2016;97(10):e137-e8. | Not relevant to orthodontics |
| 222 | Groetelaers RPTGC, Janssen L, Van Der Velden J, Wieland AWJ, Amendt AGFM, Geelen PHJ, et al. Functional treatment or cast immobilization after minimally invasive repair of an acute achilles tendon rupture: Prospective, randomized trial. Foot and Ankle International. 2014;35(8):771-8. | Not relevant to orthodontics |

| | | |
|---|---|---|
| 223 | Gueugnon M, Fournel I, Soilly AL, Diaz A, Baulot E, Bussière C, et al. Effectiveness, safety, and cost-utility of a knee brace in medial knee osteoarthritis: the ERGONOMIE randomized controlled trial. Osteoarthritis Cartilage. 2021;29(4):491-501. | Not relevant to orthodontics |
| 224 | Gupta N, Reddy UN, Vasundhar PL, Ramarao KS, Varma KP, Vinod V. Effectiveness of desensitizing agents in relieving the pre- and postcementation sensitivity for full coverage restorations: a clinical evaluation. J Contemp Dent Pract. 2013;14(5):858-65. | Not relevant to orthodontics |
| 225 | Gupta S, Kumar A, Sharma AK, Purohit J, Narula JS. "Sodium bicarbonate": an adjunct to painless palatal anesthesia. Oral Maxillofac Surg. 2018;22(4):451-5. | Not relevant to orthodontics |
| 226 | Guruprasada M, Thapliyal GK, Pawar VR. A comparative analysis of periimplant bone levels of immediate and conventionally loaded implants. Medical Journal Armed Forces India. 2013;69(1):41-7. | Not relevant to orthodontics |
| 227 | Güttinger K. Quality of Life in adolescents with idiopathic scoliosis: A comparison measured by the Kidscreen 27 between scoliotic patients and healthy controls. Scoliosis and Spinal Disorders. 2016;11. | Not relevant to orthodontics |
| 228 | Haddas R, Yang J, Lieberman IH. Effects of volitional spine stabilization on lifting task in recurrent low back pain population. Spine Journal. 2015;15(10):116S. | Not relevant to orthodontics |
| 229 | Haketa T, Kino K, Sugisaki M, Takaoka M, Ohta T. Randomized clinical trial of treatment for TMJ disc displacement. J Dent Res. 2010;89(11):1259-63. | Not relevant to orthodontics |
| 230 | Hall M, Starkey S, Hinman RS, Diamond LE, Lenton GK, Knox G, et al. Valgus knee bracing for medial knee osteoarthritis and varus malalignment: a pilot study. Osteoarthritis and Cartilage. 2021;29:S173-S4. | Not relevant to orthodontics |
| 231 | Hamata MM, Zuim PRJ, Garcia AR. Comparative evaluation of the efficacy of occlusal splints fabricated in centric relation or maximum intercuspation in temporomandibular disorders patients. J appl oral sci. 2009;17(1):32-8. | Not relevant to orthodontics |
| 232 | Hanna M, Kotsias A, Suess O. Empty peek cages in ACDF: Does partial titanium coating improve the radiographic fusion? Spine Journal. 2016;16(10):S115. | Not relevant to orthodontics |
| 233 | Hansen PB, Hansen TB. The treatment of fractures of the ring and little metacarpal necks. A prospective randomized study of three different types of treatment. J Hand Surg Br. 1998;23(2):245-7. | Not relevant to orthodontics |
| 234 | Harding IJ, Parry D, Barrington RL. The use of a moulded metacarpal brace versus neighbour strapping for fractures of the little finger metacarpal neck. J Hand Surg Br. 2001;26(3):261-3. | Not relevant to orthodontics |
| 235 | Hariharan S, Narayanan V, Soh CL. Split-mouth comparison of physics forceps and extraction forceps in orthodontic extraction of upper premolars. Br J Oral Maxillofac Surg. 2014;52(10):e137-40. | Not relevant to orthodontics |
| 236 | Hart HF, Crossley KM, Ackland DC, Cowan SM, Collins NJ. Effects of an unloader knee brace on knee-related symptoms and function in people with post-traumatic knee osteoarthritis after anterior cruciate ligament reconstruction. Knee. 2016;23(1):85-90. | Not relevant to orthodontics |
| 237 | Harte H, Palmer NOA, Martin MV. An investigation of therapeutic antibiotic prescribing for children referred for dental general anaesthesia in three community national health service trusts. British Dental Journal. 2005;198(4):227-31. | Not relevant to orthodontics |
| 238 | Harvey WF, Lo GH, McAdams E, Ripley MA, Nuite M, McAlindon TE. Varus thrust is associated with worse physical function. Arthritis and Rheumatism. 2010;62:2101. | Not relevant to orthodontics |
| 239 | Hasler A, Beeler S, Götschi T, Catanzaro S, Jost B, Gerber C. No difference in long-term outcome between open and arthroscopic rotator cuff repair: a prospective, randomized study. JSES International. 2020;4(4):818-25. | Not relevant to orthodontics |
| 240 | Hassan J, Ashar A, Abdilahi AM, Naqash A, Zahid F, Sajid AH, et al. Dry Socket in Patients Receiving Acetaminophen Versus Ibuprofen for Dental Extraction. Pakistan Journal of Medical and Health Sciences. 2021;15(12):3666-9. | Not relevant to orthodontics |
| 241 | Heidari M, Fekrazad R, Sobouti F, Moharrami M, Azizi S, Nokhbatolfoghahaei H, et al. Evaluating the effect of photobiomodulation with a 940-nm diode laser on post-operative pain in periodontal flap surgery. Lasers Med Sci. 2018;33(8):1639-45. | Not relevant to orthodontics |
| 242 | Hejne AJ. [The influence of occlusal interferences on pulp vitality threshold in permanent dentition]. Annales Academiae Medicae Stetinensis. 2003;49:277-89. | Not relevant to orthodontics |
| 243 | Helm S, Kreiborg S, Solow B. Malocclusion at adolescence related to self-reported tooth loss and functional disorders in adulthood. American Journal of Orthodontics. 1984;85(5):393-400. | Not relevant to orthodontics |
| 244 | Heredia Rizo AM, Pascual-Vaca Á O, Cabello MA, Blanco CR, Pozo FP, Carrasco AL. Immediate effects of the suboccipital muscle inhibition technique in craniocervical posture and greater occipital nerve mechanosensitivity in subjects with a history of orthodontia use: a randomized trial. J Manipulative Physiol Ther. 2012;35(6):446-53. | Not relevant to orthodontics |
| 245 | Herget GW, Patermann S, Strohm PC, Zwingmann J, Eichelberger P, Südkamp NP, et al. Spinal Orthoses: The Crucial Role of Comfort on Compliance of Wearing - Monocentric Prospective Pilot Study of Randomized Cross-Over Design. Acta Chir Orthop Traumatol Cech. 2017;84(2):91-6. | Not relevant to orthodontics |
| 246 | Herrero Solano Y, Arias Molina Y. Trastorno de la personalidad y disfunción de la articulación temporomandibular. Rev cuba estomatol. 2019;56(2):e1857-e. | Not relevant to orthodontics |
| 247 | Hesse D, de Araujo MP, Olegário IC, Innes N, Raggio DP, Bonifácio CC. Atraumatic Restorative Treatment compared to the Hall Technique for occluso-proximal cavities in primary molars: study protocol for a randomized controlled trial. Trials. 2016;17:169. | Not relevant to orthodontics |
| 248 | Heydari Z, Aminian G, Biglarian A, Shokrpour M, Mardani MA. Comparison of the Modified Lumbar Pelvic Belt with the Current Belt on Low Back and Pelvic Pain in Pregnant Women. Journal of Biomedical Physics and Engineering. 2022;12(3):309-18. | Not relevant to orthodontics |
| 249 | Hickey D, Sharif O, Janjua F, Brunton PA. Bulk dentine replacement versus incrementally placed resin composite: A randomised controlled clinical trial. J Dent. 2016;46:18-22. | Not relevant to orthodontics |
| 250 | Hill CM, Walker RV. Conservative, non-surgical management of patients presenting with impacted lower third molars: A 5-year study. British Journal of Oral and Maxillofacial Surgery. 2006;44(5):347-50. | Not relevant to orthodontics |
| 251 | Hindocha N, Manhem F, Bäckryd E, Bågesund M. Ice versus lidocaine 5% gel for topical anaesthesia of oral mucosa - a randomized cross-over study. BMC Anesthesiol. 2019;19(1):227. | Not relevant to orthodontics |
| 252 | Hjartarson HF, Toksvig-Larsen S. The clinical effect of unloader One® knee brace on patients with osteoarthritis of the knee. A randomized placebo controlled trial. Osteoarthritis and Cartilage. 2017;25:S173. | Not relevant to orthodontics |
| 253 | Hollman F, Wolterbeek N, Zijl JAC, van Egeraat SPM, Wessel RN. Abduction Brace Versus Antirotation Sling After Arthroscopic Cuff Repair: The Effects on Pain and Function. Arthroscopy. 2017;33(9):1618-26. | Not relevant to orthodontics |
| 254 | Hovelius L. The natural history of primary anterior dislocation of the shoulder in the young. J Orthop Sci. 1999;4(4):307-17. | Not relevant to orthodontics |
| 255 | Hsieh CY, Phillips RB, Adams AH, Pope MH. Functional outcomes of low back pain: comparison of four treatment groups in a randomized controlled trial. J Manipulative Physiol Ther. 1992;15(1):4-9. | Not relevant to orthodontics |
| 256 | Hsu JR, Owens JG, DeSanto J, Fergason JR, Kuhn KM, Potter BK, et al. Patient Response to an Integrated Orthotic and Rehabilitation Initiative for Traumatic Injuries: The PRIORITI-MTF Study. J Orthop Trauma. 2017;31 Suppl 1:S56-s62. | Not relevant to orthodontics |
| 257 | Hunter D, Gross KD, McCree P, Li L, Hirko K, Harvey WF. Realignment treatment for medial tibiofemoral osteoarthritis: randomised trial. Ann Rheum Dis. 2012;71(10):1658-65. | Not relevant to orthodontics |
| 258 | Hunter DJ, Harvey W, Gross KD, Felson D, McCree P, Li L, et al. A randomized trial of patellofemoral bracing for treatment of patellofemoral osteoarthritis. Osteoarthritis Cartilage. 2011;19(7):792-800. | Not relevant to orthodontics |
| 259 | Hutton CG, Johnson GK, Barwacz CA, Allareddy V, Avila-Ortiz G. Comparison of two different surgical approaches to increase peri-implant mucosal thickness: A randomized controlled clinical trial. J Periodontol. 2018;89(7):807-14. | Not relevant to orthodontics |
| 260 | Inose H, Kato T, Ichimura S, Nakamura H, Hoshino M, Togawa D, et al. Risk factors for subsequent vertebral fracture after acute osteoporotic vertebral fractures. Eur Spine J. 2021;30(9):2698-707. | Not relevant to orthodontics |
| 261 | Inose H, Kato T, Okawa A. A prospective, randomized multicenter study comparing rigid-and soft-brace treatment for acute osteoporotic vertebral compression fracture. Osteoporosis International. 2019;30(SUPPL 2):S316. | Not relevant to orthodontics |
| 262 | Iulia SC, Cosmin-Gabriel P, Daniela A, Ecaterina SA, Constantin CC, Loredana G, et al. Cross-Sectional Study to Evaluate the Influence of Nutritional Behavior on the Dental Health of Romanian Teenagers. Progress in Nutrition. 2021;23(4). | Not relevant to orthodontics |
| 263 | Ize-Iyamu IN, Saheeb BD, Edetanlen BE. Comparing the 810nm diode laser with conventional surgery in orthodontic soft tissue procedures. Ghana Med J. 2013;47(3):107-11. | Not relevant to orthodontics |
| 264 | Jackman M, Novak I, Lannin N. Effectiveness of hand splints in children with cerebral palsy: A systematic review with metaanalysis. Developmental Medicine and Child Neurology. 2014;56:38. | Not relevant to orthodontics |
| 265 | Jadhao VA, Lokhande N, Habbu SG, Sewane S, Dongare S, Goyal N. Efficacy of botulinum toxin in treating myofascial pain and occlusal force characteristics of masticatory muscles in bruxism. Indian J Dent Res. 2017;28(5):493-7. | Not relevant to orthodontics |
| 266 | Jadhao VA, Rao A, Hande P, Mahajani M, Raktade PP, Gedam R, et al. The Efficiency of Three Irrigating Solutions after Surgical Removal of Impacted Mandibular Third Molars: A Cross-sectional Study. J Contemp Dent Pract. 2018;19(9):1147-51. | Not relevant to orthodontics |
| 267 | Jadhav S, Deshpande S, Gormley M. Treatment of pediatric idiopathic toe walking with and without botulinum toxin and ankle foot orthosis. Developmental Medicine and Child Neurology. 2017;59:81. | Not relevant to orthodontics |
| 268 | Jain N, Garg S, Dhindsa A, Joshi S, Khatria H. Impact of 6% citric acid and endoactivator as irrigation adjuncts on obturation quality and pulpectomy outcome in primary teeth. Pediatric Dental Journal. 2019;29(2):59-65. | Not relevant to orthodontics |
| 269 | Jajarm HH, Falaki F, Sanatkhani M, Ahmadzadeh M, Ahrari F, Shafaee H. A comparative study of toluidine blue-mediated photodynamic therapy versus topical corticosteroids in the treatment of erosive-atrophic oral lichen planus: a randomized clinical controlled trial. Lasers in Medical Science. 2015;30(5):1475-80. | Not relevant to orthodontics |
| 270 | Jakobsen BW, Johannsen HV, Suder P, Søjbjerg JO. Primary repair versus conservative treatment of first-time traumatic anterior dislocation of the shoulder: a randomized study with 10-year follow-up. Arthroscopy. 2007;23(2):118-23. | Not relevant to orthodontics |
| 271 | Janssen KW, van Mechelen W, Verhagen EA. Ankles back in randomized controlled trial (ABrCt): braces versus neuromuscular exercises for the secondary prevention of ankle sprains. Design of a randomised controlled trial. BMC Musculoskelet Disord. 2011;12:210. | Not relevant to orthodontics |
| 272 | Januzzi E, Alves BMF, Grossmann E, Leite FMG, Vieira PdSR, Flecha OD. Occlusion and temporomandibular disorders: a critical analysis of the literature. Rev dor. 2010;11(4). | Not relevant to orthodontics |
| 273 | Janzing HMJ, Bessems SAM, Ligthart MAP, Van Lieshout EMM, Theeuwes HP, Barten DG, et al. Treatment of dorsally dislocated distal radius fractures with individualized 3D printed bracing: An exploratory study. 3D Printing in Medicine. 2020;6(1). | Not relevant to orthodontics |
| 274 | Javed O, Bernabé E. Oral impacts on quality of life in adult patients with class I, II and III malocclusion. Oral Health and Preventive Dentistry. 2016;14(1):27-32. | Not relevant to orthodontics |
| 275 | Jensen U, Ruf S. Longitudinal changes in temporomandibular disorders in young adults: Indication for systematic temporomandibular joint screening. Journal of Orofacial Orthopedics. 2007;68(6):501-9. | Not relevant to orthodontics |
| 276 | John SS, Mohanty S, Chaudhary Z, Sharma P, Kumari S, Verma A. Comparative evaluation of Low Level Laser Therapy and cryotherapy in pain control and wound healing following orthodontic tooth extraction: A double blind study. J Craniomaxillofac Surg. 2020;48(3):251-60. | Not relevant to orthodontics |
| 277 | Johnson S. Patellar bracing for patellofemoral pain syndrome: Commentary. Clinical Journal of Sport Medicine. 2006;16(6):530-1. | Not relevant to orthodontics |
| 278 | Jokstad A, Mo A, Krogstad BS. Clinical comparison between two different splint designs for temporomandibular disorder therapy. Acta Odontol Scand. 2005;63(4):218-26. | Not relevant to orthodontics |
| 279 | Jones A, Brumini C, Natour J, Miura LY. Effectiveness of bracing in elderly with knee osteoartritis: A randomized controlled trial. Archives of Physical Medicine and Rehabilitation. 2016;97(12):e12. | Not relevant to orthodontics |
| 280 | Jones RK, Nester CJ, Richards JD, Kim WY, Johnson DS, Jari S, et al. A comparison of the biomechanical effects of valgus knee braces and lateral wedged insoles in patients with knee osteoarthritis. Gait Posture. 2013;37(3):368-72. | Not relevant to orthodontics |
| 281 | Jonker BP, Gil A, Naenni N, Jung RE, Wolvius EB, Pijpe J. Soft tissue contour and radiographic evaluation of ridge preservation in early implant placement: A randomized controlled clinical trial. Clin Oral Implants Res. 2021;32(1):123-33. | Not relevant to orthodontics |
| 282 | Jost-Brinkmann PG, Stein H, Miethke RR, Nakata M. Histologic investigation of the human pulp after thermodebonding of metal and ceramic brackets. American Journal of Orthodontics and Dentofacial Orthopedics. 1992;102(5):410-7. | Not relevant to orthodontics |
| 283 | Kaciroti N, DosSantos MF, Moura B, Bellile EL, Nascimento TD, Maslowski E, et al. Sensory-Discriminative Three-Dimensional Body Pain Mobile App Measures Versus Traditional Pain Measurement With a Visual Analog Scale: Validation Study. JMIR Mhealth Uhealth. 2020;8(8):e17754. | Not relevant to orthodontics |
| 284 | Kapadia BH, Cherian JJ, Starr R, Chughtai M, Mont MA, Harwin SF, et al. Gait Using Pneumatic Brace for End-Stage Knee Osteoarthritis. J Knee Surg. 2016;29(3):218-23. | Not relevant to orthodontics |
| 285 | Karppinen K, Eklund S, Suoninen E, Eskelin M, Kirveskari P. Adjustment of dental occlusion in treatment of chronic cervicobrachial pain and headache. Journal of Oral Rehabilitation. 1999;26(9):715-21. | Not relevant to orthodontics |
| 286 | Kato T, Inose H, Ichimura S, Tokuhashi Y, Nakamura H, Hoshino M, et al. Comparison of rigid and soft-brace treatments for acute osteoporotic vertebral compression fracture: A prospective, randomized, multicenter study. Journal of Clinical Medicine. 2019;8(2). | Not relevant to orthodontics |
| 287 | Katpattil S. A risk assessment study on work-related musculoskeletal disorders among dentists in mangalore city. Osteoporosis International. 2017;28:S378-S9. | Not relevant to orthodontics |
| 288 | Katz MS, Peters F, Elvers D, Winterhalder P, Kniha K, Möhlhenrich SC, et al. Effect of drain application on postoperative complaints after surgical removal of impacted wisdom teeth-a randomized observer-blinded split-mouth clinical trial. Clin Oral Investig. 2021;25(1):345-53. | Not relevant to orthodontics |
| 289 | Kawadler J, Liossi C, Inusa B, Clark C, Rees D, Pelidis M, et al. Reduced cortical thickness in patients with Sickle Cell Disease and a high pain burden: Baseline results from the Prevention of Morbidity in Sickle Cell Anaemia (POMS2b) trial. British Journal of Haematology. 2017;176:18. | Not relevant to orthodontics |
| 290 | Kaya MS, Taran PK, Bakkal M. Temporomandibular dysfunction assessment in children treated with the Hall Technique: A pilot study. International Journal of Paediatric Dentistry. 2020;30(4):429-35. | Not relevant to orthodontics |
| 291 | Keene DJ, Willett K, Lamb SE. The Immediate Effects of Different Types of Ankle Support Introduced 6 Weeks After Surgical Internal Fixation for Ankle Fracture on Gait and Pain: A Randomized Crossover Trial. J Orthop Sports Phys Ther. 2016;46(3):157-67. | Not relevant to orthodontics |
| 292 | Kerstein RB, Chapman R, Klein M. A comparison of ICAGD (Immediate Complete Anterior Guidance Development) to mock ICAGD for symptom reductions in chronic myofascial pain dysfunction patients. Cranio - Journal of Craniomandibular and Sleep Practice. 1997;15(1):21-37. | Not relevant to orthodontics |
| 293 | Kerstein RB. Disclusion time measurement studies: A comparison of disclusion time between chronic myofascial pain dyfunction patients and nonpatients: A population analysis. The Journal of Prosthetic Dentistry. 1994;72(5):473-80. | Not relevant to orthodontics |
| 294 | Khan JA, Nileshwar A. Comparison of the intubating laryngeal mask airway with the bullard laryngoscope for endotracheal intubation in patients with simulated difficult airway using the philadelphia cervical collar. Journal of Anaesthesiology Clinical Pharmacology. 2009;25(1):33-7. | Not relevant to orthodontics |
| 295 | Khan RA, Khan FA, Azam M. Comparison of the stylet and the gum elastic bougie in tracheal intubation in a simulated difficult airway. Anaesthesia, Pain and Intensive Care. 2014;18(3):256-9. | Not relevant to orthodontics |
| 296 | Kim DH, Yoo JY, Ha SY, Chae YJ. Comparison of the paediatric blade of the Pentax-AWS and Ovassapian airway in fibreoptic tracheal intubation in patients with limited mouth opening and cervical spine immobilization by a semi-rigid neck collar: a randomized controlled trial. Br J Anaesth. 2017;119(5):993-9. | Not relevant to orthodontics |
| 297 | Kim H, Racette B, Dunn C, Mukherjee S, McCusker E, Liu C, et al. Lower limb injections of onabotulinumtoxinA: Improvement in gait and treatment goal achievement in pediatric patients with cerebral palsy. Toxicon. 2021;190:S40-S1. | Not relevant to orthodontics |
| 298 | Kim HJ, Yi JM, Cho HG, Chang BS, Lee CK, Kim JH, et al. Comparative study of the treatment outcomes of osteoporotic compression fractures without neurologic injury using a rigid brace, a soft brace, and no brace: a prospective randomized controlled non-inferiority trial. J Bone Joint Surg Am. 2014;96(23):1959-66. | Not relevant to orthodontics |
| 299 | Kim JG, Bang SH, Kang GH, Jang YS, Kim W, Choi HY, et al. Comparison of the efficacy of three cervical collars in restricting cervical range of motion: A randomized study. Hong Kong Journal of Emergency Medicine. 2020;27(1):24-9. | Not relevant to orthodontics |
| 300 | Kim JK, Kim JA, Kim CS, Ahn HJ, Yang MK, Choi SJ. Comparison of tracheal intubation with the Airway Scope or Clarus Video System in patients with cervical collars. Anaesthesia. 2011;66(8):694-8. | Not relevant to orthodontics |
| 301 | King L, Waugh E, Marshall D, Hawker G. The relationship between comorbidity and non-surgical treatment of knee osteoarthritis. Journal of Rheumatology. 2017;44(6):939. | Not relevant to orthodontics |

| | | |
|---|---|---|
| 302 | Kirkley A, Webster-Bogaert S, Litchfield R, Amendola A, MacDonald S, McCalden R, et al. The effect of bracing on varus gonarthrosis. J Bone Joint Surg Am. 1999;81(4):539-48. | Not relevant to orthodontics |
| 303 | Kirnbauer B, Jakse N, Truschnegg A, Dzidic I, Mukaddam K, Payer M. Is perioperative antibiotic prophylaxis in the case of routine surgical removal of the third molar still justified? A randomized, double-blind, placebo-controlled clinical trial with a split-mouth design. Clin Oral Investig. 2022;26(10):6409-21. | Not relevant to orthodontics |
| 304 | Kirveskari P, Jamsa T, Alanen P. Occlusal adjustment and the incidence of demand for temporomandibular disorder treatment. Journal of Prosthetic Dentistry. 1998;79(4):433-8. | Not relevant to orthodontics |
| 305 | Kirveskari P, Jämsä T. Health risk from occlusal interferences in females. European Journal of Orthodontics. 2009;31(5):490-5. | Not relevant to orthodontics |
| 306 | Kirveskari P. Prediction of demand for treatment of temporomandibular disorders. Journal of Oral Rehabilitation. 2001;28(6):572-5. | Not relevant to orthodontics |
| 307 | Klur T, Hasan I, Ottersbach K, Stark H, Fichte M, Dirk C, et al. PEKK-made indirect temporary crowns and bridges: a clinical pilot study. Clin Oral Investig. 2019;23(2):771-7. | Not relevant to orthodontics |
| 308 | Knobloch K, Schreibmueller L, Longo UG, Vogt PM. Eccentric exercises for the management of tendinopathy of the main body of the Achilles tendon with or without an AirHeel Brace. A randomized controlled trial. B: Effects of compliance. Disabil Rehabil. 2008;30(20-22):1692-6. | Not relevant to orthodontics |
| 309 | Knobloch K, Schreibmueller L, Longo UG, Vogt PM. Eccentric exercises for the management of tendinopathy of the main body of the Achilles tendon with or without the AirHeel Brace. A randomized controlled trial. A: effects on pain and microcirculation. Disabil Rehabil. 2008;30(20-22):1685-91. | Not relevant to orthodontics |
| 310 | Koçer A, Özkan HD, Turk T. Postoperative pain intensity and incidence following single visit root canal treatment with different obturation techniques: a randomized clinical trial. PeerJ. 2022;10. | Not relevant to orthodontics |
| 311 | Kölle T, Alt W, Wagner D. Immediate effects of an elastic patellar brace on pain, neuromuscular activity and knee kinematics in subjects with patellofemoral pain. Arch Orthop Trauma Surg. 2020;140(7):905-12. | Not relevant to orthodontics |
| 312 | Koos B, Stoustrup P, Spiegel L, Tzaribachev N, Herlin T, Pedersen T, et al. Clinical examination of the temporomandibular joint; A eurotmjoint initiative. Journal of Rheumatology. 2016;43(6):1203. | Not relevant to orthodontics |
| 313 | Koparal M, Kucuk AO, Alan H, Asutay F, Avci M. Effects of low-level laser therapy following surgical extraction of the lower third molar with objective measurement of swelling using a three-dimensional system. Experimental and Therapeutic Medicine. 2018;15(4):3820-6. | Not relevant to orthodontics |
| 314 | Korovessis P, Repantis T, Baikousis A, Iliopoulos P. Posterolateral versus circumferential instrumented fusion for monosegmental lumbar degenerative disc disease using an expandable cage. European Journal of Orthopaedic Surgery and Traumatology. 2012;22(8):639-45. | Not relevant to orthodontics |
| 315 | Krahwinkel T, Theiss P, Willershausen B. Clinical effectiveness of a potassium chloride containing chewing gum in the treatment of hypersensitive teeth. European Journal of Medical Research. 2001;6(11):483-7. | Not relevant to orthodontics |
| 316 | Kroslak M, Murrell G. Counterforce bracing of lateral epicondylitis: A prospective, randomised, double blinded, placebo controlled clinical trial. Journal of Science and Medicine in Sport. 2018;21:S8. | Not relevant to orthodontics |
| 317 | Kroslak M, Pirapakaran K, Murrell GAC. Counterforce bracing of lateral epicondylitis: a prospective, randomized, double-blinded, placebo-controlled clinical trial. J Shoulder Elbow Surg. 2019;28(2):288-95. | Not relevant to orthodontics |
| 318 | Kruger LR, van der Linden WJ, Cleaton-Jones PE. Transcutaneous electrical nerve stimulation in the treatment of myofascial pain dysfunction. S Afr J Surg. 1998;36(1):35-8. | Not relevant to orthodontics |
| 319 | Kurita H, Ikeda K, Kurashina K. Evaluation of the effect of a stabilization splint on occlusal force in patients with masticatory muscle disorders. Journal of Oral Rehabilitation. 2000;27(1):79-82. | Not relevant to orthodontics |
| 320 | Kurnaz S, Bag I, Gulbandilar A. Apical extrusion of bacteria following the use of reciprocating single-file and rotary multi-file instrumentation systems in oval root canals. Braz dent sci. 2020;23(4):1-8. | Not relevant to orthodontics |
| 321 | Ladny M, Szarpak L, Smereka J, Ladny JR. A comparison of comfort assessment of NECKLITE vs. NeXsplint cervical collar. Pilot data. Am J Emerg Med. 2018;36(11):2127-8. | Not relevant to orthodontics |
| 322 | Lamb SE, Marsh JL, Hutton JL, Nakash R, Cooke MW. Mechanical supports for acute, severe ankle sprain: a pragmatic, multicentre, randomised controlled trial. Lancet. 2009;373(9663):575-81. | Not relevant to orthodontics |
| 323 | Lardenoye S, Theunissen E, Cleffken B, Brink PR, de Bie RA, Poeze M. The effect of taping versus semi-rigid bracing on patient outcome and satisfaction in ankle sprains: a prospective, randomized controlled trial. BMC Musculoskelet Disord. 2012;13:81. | Not relevant to orthodontics |
| 324 | Laszlo J, Pivec N. Effect of Inhomogeneous Static Magnetic Field on Dental Pain in Humans. Clinical Journal of Pain. 2010;26(1):49-55. | Not relevant to orthodontics |
| 325 | Laszlo JF, Farkas P, Reiczigel J, Vago P. Effect of local exposure to inhomogeneous static magnetic field on stomatological pain sensation - a double-blind, randomized, placebo-controlled study. International Journal of Radiation Biology. 2012;88(5):430-8. | Not relevant to orthodontics |
| 326 | Le Pluart A, Guillaume C, Darrieutort C, Cormier G, Sophie G, Ottaviani S, et al. "no Benefit from a Strict Immobilization in Vertebral Osteomyelitis" Vertebral Immobilization and Neurological Complication in Acute Pyogenic Vertebral Osteomyelitis: SPONDIMMO, a Prospective Cohort of 250 Patients. Arthritis and Rheumatology. 2020;72(SUPPL 10):1266-8. | Not relevant to orthodontics |
| 327 | Learreta JA, Bono AE, Maffia G, Beas J. Identificación de patologías articulares por medio del estudio del ciclo masticatorio, en pacientes preortodóncicos. Parte I: descripción del ciclo masticatorio. Ortodoncia. 2003;67(133):8-13. | Not relevant to orthodontics |
| 328 | Learreta JA, Bono AE, Maffia G, Beas J. Identificación de patologías articulares por medio del estudio del ciclo masticatorio. Ortodoncia. 2004;68(135):8-15. | Not relevant to orthodontics |
| 329 | Lee DJ, Ahmed SA, Tang OY, Yang DS, Alsoof D, McDonald CL, et al. Comparative Effectiveness of Sacroiliac Belt versus Lumbar Orthosis Utilization on Nonspecific Low Back Pain: a Crossover Randomized Clinical Trial. Orthopedic Reviews. 2022;14(3). | Not relevant to orthodontics |
| 330 | Lee DY, Kim JI, Song MH, Choi ES, Park MS, Yoo WJ, et al. Fibular lengthening for the management of translational talus instability in hereditary multiple exostoses patients. J Pediatr Orthop. 2014;34(7):726-32. | Not relevant to orthodontics |
| 331 | Lee HH, Kim ST, Lee KJ, Baik HS. Effect of a second injection of botulinum toxin on lower facial contouring, as evaluated using 3-dimensional laser scanning. Dermatol Surg. 2015;41(4):439-44. | Not relevant to orthodontics |
| 332 | Leib AM. Patient Preference for Light-Cured Composite Bite Splint Compared to Heat-Cured Acrylic Bite Splint. Journal of Periodontology. 2001;72(8):1108-12. | Not relevant to orthodontics |
| 333 | Li J, Chen L, Zeng C, Liu Y, Gong Q, Jiang H. Clinical outcome of bioceramic sealer iRoot SP extrusion in root canal treatment: a retrospective analysis. Head and Face Medicine. 2022;18(1). | Not relevant to orthodontics |
| 334 | Li M, Law SW, Cheng J, Kee HM, Wong MS. A comparison study on the efficacy of SpinoMed® and soft lumbar orthosis for osteoporotic vertebral fracture. Prosthet Orthot Int. 2015;39(4):270-6. | Not relevant to orthodontics |
| 335 | Libermann S, Etchepareborde S, Gautherot A, Etienne-Raffestin CL, Coste M, Moreau S, et al. Laparoscopic castration in dogs: Complications, outcomes and long-term follow-up. Revue Veterinaire Clinique. 2020;55(1):1-10. | Not relevant to orthodontics |
| 336 | Liedl B, Inoue H, Sekiguchi Y, Haverfield M, Richardson P, Yassourides A, et al. Is overactive bladder in the female surgically curable by ligament repair? Central European Journal of Urology. 2017;70(1):53-9. | Not relevant to orthodontics |
| 337 | Lima TC, Vieira-Barbosa NM, Grasielle de Sá Azevedo C, de Matos FR, Douglas de Oliveira DW, de Oliveira ES, et al. Oral Health-Related Quality of Life Before and After Treatment of Dentin Hypersensitivity With Cyanoacrylate and Laser. J Periodontol. 2017;88(2):166-72. | Not relevant to orthodontics |
| 338 | Limchaichana N, Nilsson H, Petersson A, Ekberg E. Resilient appliance-therapy treatment outcome in patients with TMD pain correlated to MRI-determined changes in condyle position. Cranio. 2009;27(3):185-93. | Not relevant to orthodontics |
| 339 | Lin YC, Gau ML, Kao GH, Lee HC. Efficacy of an Ergonomic Ankle Support Aid for Squatting Position in Improving Pushing Skills and Birth Outcomes During the Second Stage of Labor: A Randomized Controlled Trial. J Nurs Res. 2018;26(6):376-84. | Not relevant to orthodontics |
| 340 | Lind M, Lund B, Faunoe P, Christiansen SE, Nielsen T. The impact of free or restricted rehabilitation after meniscus repair. A prospective randomized clinical trial. Arthroscopy - Journal of Arthroscopic and Related Surgery. 2013;29(10):e148. | Not relevant to orthodontics |
| 341 | Lind M, Nielsen T, Faunø P, Lund B, Christiansen SE. Free rehabilitation is safe after isolated meniscus repair: a prospective randomized trial comparing free with restricted rehabilitation regimens. Am J Sports Med. 2013;41(12):2753-8. | Not relevant to orthodontics |
| 342 | Lindfors E, Nilsson H, Helkimo M, Magnusson T. Treatment of temporomandibular disorders with a combination of hard acrylic stabilisation appliance and a soft appliance in the opposing jaw. A retro- and prospective study. Swedish Dental Journal. 2008;32(1):9-16. | Not relevant to orthodontics |
| 343 | Lippold C, Danesh G, Schilgen M, Drerup B, Hackenberg L. Sagittal jaw position in relation to body posture in adult humans - a rasterstereographic study. Bmc Musculoskeletal Disorders. 2006;7. | Not relevant to orthodontics |
| 344 | Liu J, Wang X, Shan P, Hu S, Liu D, Ma J, et al. A randomized controlled trial: evaluation of efficiency and safety of a novel surgical guide in the extraction of deeply impacted supernumerary teeth in the anterior maxilla. Annals of Translational Medicine. 2022;10(6). | Not relevant to orthodontics |
| 345 | Liu Y, Li CY, Shao HF, Liang S. Analysis clinical effect of negative pressure drainage in treatment of acute periapical periodontitis. Journal of Jilin University Medicine Edition. 2011;37(2):331-4. | Not relevant to orthodontics |
| 346 | Lo GH, McAlindon T, Carr KA, Ripley MA, Nuite M, Harvey WF. Varus thrust is associated with pain in knee osteoarthritis. Arthritis and Rheumatism. 2009;60:831. | Not relevant to orthodontics |
| 347 | Lohle PNM, Firanescu CE, De Vries J. Update on VERTOS IV. CardioVascular and Interventional Radiology. 2016;39(3):S123-S4. | Not relevant to orthodontics |
| 348 | Longo UG, Loppini M, Denaro L, Brandi ML, Maffulli N, Denaro V. The effectiveness and safety of vertebroplasty for osteoporotic vertebral compression fractures. A double blind, prospective, randomized, controlled study. Clinical Cases in Mineral and Bone Metabolism. 2010;7(2):109-13. | Not relevant to orthodontics |
| 349 | Lopez-Jornet P, Juan H, Alvaro P-F. Mineral and trace element analysis of saliva from patients with BMS: a cross-sectional prospective controlled clinical study. Journal of Oral Pathology & Medicine. 2014;43(2):111-6. | Not relevant to orthodontics |
| 350 | Lovell MR, Birch MR, Luckett T, Davidson PM, Phillips J, Agar M, et al. Pilot of pain indicator audit tool as part of a complex intervention to improve cancer pain outcomes. Asia-Pacific Journal of Clinical Oncology. 2014;10:45. | Not relevant to orthodontics |
| 351 | Lun VM, Wiley JP, Meeuwisse WH, Yanagawa TL. Effectiveness of patellar bracing for treatment of patellofemoral pain syndrome. Clin J Sport Med. 2005;15(4):235-40. | Not relevant to orthodontics |
| 352 | Luong P, King F, Li ZM, Dickason M, Diamond M, Son J. Pilot clinical study of a novel unobtrusive carpal tunnel tissue manipulation device in reducing symptoms of carpal tunnel syndrome. Arthritis and Rheumatology. 2017;69. | Not relevant to orthodontics |
| 353 | MacDermid J. A hand brace improve symptoms and function in carpal tunnel syndrome. Aust J Physiother. 2002;48(2):134. | Not relevant to orthodontics |
| 354 | Macfarlane TV, Blinkhorn AS, Davies RM, Worthington HV. Association between local mechanical factors and orofacial pain: Survey in the community. Journal of Dentistry. 2003;31(8):535-42. | Not relevant to orthodontics |
| 355 | Machado E, Bonotto D, Cunali PA. Intra-articular injections with corticosteroids and sodium hyaluronate for treating temporomandibular joint disorders: A systematic review. Dental Press Journal of Orthodontics. 2013;18(5):128-33. | Not relevant to orthodontics |
| 356 | Madara K, Aljehani M, Pozzi F, Colonna E, Zeni JA. The effect of extension assist orthosis with pneumatic bladders on pain and function for patients with early knee osteoarthritis. Annals of Translational Medicine. 2019;7. | Not relevant to orthodontics |
| 357 | Magnusson T, Egermark I, Carlsson GE. A prospective investigation over two decades on signs and symptoms of temporomandibular disorders and associated variables. A final summary. Acta Odontologica Scandinavica. 2005;63(2):99-109. | Not relevant to orthodontics |
| 358 | Mahmood A, Khan ZR, Saleem B, Memon ZA, Abro B, Hassan G. Post-Operative Complications after Simple Tooth Extraction in Diabetic Patients with and without Antibiotic Prophylaxis. Pakistan Journal of Medical and Health Sciences. 2021;15(12):3614-6. | Not relevant to orthodontics |
| 359 | Maleki M, Arazpour M, Ahmadi Bani M. Comparison of the efficacy of laterally wedged insoles and bespoke unloader knee orthoses in treating medial compartment knee osteoarthritis. Osteoporosis International. 2018;29(1):S186. | Not relevant to orthodontics |
| 360 | Manfredini D, Favero L, Cocilovo F, Monici M, Guarda-Nardini L. A comparison trial between three treatment modalities for the management of myofascial pain of jaw muscles: A preliminary study. Cranio. 2018;36(5):327-31. | Not relevant to orthodontics |
| 361 | Mangano F, Gandolfi A, Luongo G, Logozzo S. Intraoral scanners in dentistry: A review of the current literature. BMC Oral Health. 2017;17(1). | Not relevant to orthodontics |
| 362 | Marbach F, Odorizzi M, Binda T, Previtali D, Filardo G, Candrian C. Sling vs brace after surgery for rotator cuff tears: A random-ized controlled trial. (8373). Swiss Medical Weekly. 2020;150(SUPPL 244):47S-8S. | Not relevant to orthodontics |
| 363 | Marbach F, Schönweger F, Feltri P, Filardo G, Candrian C. Sling vs Brace after Surgery for Supraspinatus Tears: A Randomized Controlled Trial (11332). Swiss Medical Weekly. 2022;152:9S. | Not relevant to orthodontics |
| 364 | Marini I, Bartolucci ML, Bortolotti F, Gatto MR, Bonetti GA. Palmitoylethanolamide versus a nonsteroidal anti-inflammatory drug in the treatment of temporomandibular joint inflammatory pain. J Orofac Pain. 2012;26(2):99-104. | Not relevant to orthodontics |
| 365 | Marini I, Gatto MR, Bonetti GA. Effects of superpulsed low-level laser therapy on temporomandibular joint pain. Clin J Pain. 2010;26(7):611-6. | Not relevant to orthodontics |
| 366 | Marras WS, Jorgensen MJ, Davis KG. Effect of foot movement and an elastic lumbar back support on spinal loading during free-dynamic symmetric and asymmetric lifting exertions. Ergonomics. 2000;43(5):653-68. | Not relevant to orthodontics |
| 367 | Martins Junior RL, Kerber FdC, Stuginski-Barbosa J. Attitudes of a group of Brazilian orthodontists towards the diagnosis and management of primary headache (migraine): an electronic-based survey. J appl oral sci. 2011;19(6):674-8. | Not relevant to orthodontics |
| 368 | Masood M, Masood Y, Newton T. Cross-bite and oral health related quality of life in young people. Journal of Dentistry. 2014;42(3):249-55. | Not relevant to orthodontics |
| 369 | Mateo-Castillo JF, Olano-Dextre TL, Teixeira das Neves L, Kenji Nishiyama C, de Souza Faco RA, de Castro Pinto L. Abordagem endodôntica em transplante dentário autógeno em indivíduo com fissura labiopalatina. Rev cuba estomatol. 2017;54(4):1-12. | Not relevant to orthodontics |
| 370 | Matsunaga FT, Tamaoki MJ, Matsumoto MH, dos Santos JB, Faloppa F, Belloti JC. Treatment of the humeral shaft fractures--minimally invasive osteosynthesis with bridge plate versus conservative treatment with functional brace: study protocol for a randomised controlled trial. Trials. 2013;14:246. | Not relevant to orthodontics |
| 371 | Matsunaga FT, Tamaoki MJ, Matsumoto MH, Netto NA, Faloppa F, Belloti JC. Minimally Invasive Osteosynthesis with a Bridge Plate Versus a Functional Brace for Humeral Shaft Fractures: A Randomized Controlled Trial. J Bone Joint Surg Am. 2017;99(7):583-92. | Not relevant to orthodontics |
| 372 | Mauricio E, Sliepen M, Rosenbaum D. Biomechanical immediate and mid-term (6-week) effects of an ankle foot orthosis in knee osteoarthritis patients. Gait and Posture. 2017;57:234-5. | Not relevant to orthodontics |
| 373 | Mayr HO, Stüeken P, Münch EO, Wolter M, Bernstein A, Suedkamp NP, et al. Brace or no-brace after ACL graft? Four-year results of a prospective clinical trial. Knee Surg Sports Traumatol Arthrosc. 2014;22(5):1156-62. | Not relevant to orthodontics |
| 374 | McKeon BP, Zaslav KR, Alfred RH, Alley RM, Edelson RH, Gersoff WK, et al. Preliminary Results From a US Clinical Trial of a Novel Synthetic Polymer Meniscal Implant. Orthopaedic Journal of Sports Medicine. 2020;8(9). | Not relevant to orthodontics |
| 375 | McMahon SB, Dargan P, Lanas A, Wiffen P. The burden of musculoskeletal pain and the role of topical non-steroidal anti-inflammatory drugs (NSAIDs) in its treatment. Ten underpinning statements from a global pain faculty. Curr Med Res Opin. 2021;37(2):287-92. | Not relevant to orthodontics |
| 376 | Melo L, De Meneses SF, Johnson VL, Deveza LA, Hunter DJ. Stepped care approach for medial tibiofemoral osteoarthritis. Osteoarthritis and Cartilage. 2017;25:S176. | Not relevant to orthodontics |
| 377 | Menezes-Silva R, Velasco SRM, Bastos RS, Molina G, Honório HM, Frencken JE, et al. Randomized clinical trial of class II restoration in permanent teeth comparing ART with composite resin after 12 months. Clin Oral Investig. 2019;23(9):3623-35. | Not relevant to orthodontics |
| 378 | Menon VD, Muthusekhar MR. Comparison of vazirani akinosi and gow gates technique in mandibular anesthesia. International Journal of Pharmaceutical Research. 2020;12:4191-5. | Not relevant to orthodontics |

| | | |
|---|---|---|
| 379 | Menziletoglu D, Guler AY, Basturk F, Isik BK, Erdur EA. Comparison of two different flap designs for bilateral impacted mandibular third molar surgery. J Stomatol Oral Maxillofac Surg. 2020;121(4):368-72. | Not relevant to orthodontics |
| 380 | Merli M, Nieri M, Mariotti G, Merli M, Franchi L, Quiroga Souki B. The fence technique: Autogenous bone graft versus 50% deproteinized bovine bone matrix / 50% autogenous bone graft-A clinical double-blind randomized controlled trial. Clin Oral Implants Res. 2020;31(12):1223-31. | Not relevant to orthodontics |
| 381 | Metz R, Verleisdonk EJ, van der Heijden GJ, Clevers GJ, Hammacher ER, Verhofstad MH, et al. Acute Achilles tendon rupture: minimally invasive surgery versus nonoperative treatment with immediate full weightbearing--a randomized controlled trial. Am J Sports Med. 2008;36(9):1688-94. | Not relevant to orthodontics |
| 382 | Michelotti A, Farella M, Martina R. Sensory and motor changes of the human jaw muscles during induced orthodontic pain. Eur J Orthod. 1999;21(4):397-404. | Not relevant to orthodontics |
| 383 | Michelotti A, Farella M, Steenks MH, Gallo LM, Palla S. No effect of experimental occlusal interferences on pressure pain thresholds of the masseter and temporalis muscles in healthy women. Eur J Oral Sci. 2006;114(2):167-70. | Not relevant to orthodontics |
| 384 | Michelotti A, Steenks MH, Farella M, Parisini F, Cimino R, Martina R. The additional value of a home physical therapy regimen versus patient education only for the treatment of myofascial pain of the jaw muscles: short-term results of a randomized clinical trial. J Orofac Pain. 2004;18(2):114-25. | Not relevant to orthodontics |
| 385 | Mikkelsen C, Cerulli G, Lorenzini M, Bergstrand G, Werner S. Can a post-operative brace in slight hyperextension prevent extension deficit after anterior cruciate ligament reconstruction? A prospective randomised study. Knee Surg Sports Traumatol Arthrosc. 2003;11(5):318-21. | Not relevant to orthodontics |
| 386 | Miotto E, Salvatore Freitas KM, Mori AA, Valarelli FP, Gobbi de Oliveira RC, Oliveira RC. Effect of botulinum toxin on quality of life of patients with chronic myofascial pain. Pain Manag. 2021;11(5):583-93. | Not relevant to orthodontics |
| 387 | Miyamoto T, Lang M, Khan S, Kumagai K, Nunn ME. The clinical efficacy of deproteinized bovine bone mineral with 10% collagen in conjunction with localized piezosurgical decortication enhanced orthodontics: A prospective observational study. Journal of Periodontology. 2019;90(10):1106-15. | Not relevant to orthodontics |
| 388 | Mobilio N, Catapano S. Effect of experimental jaw muscle pain on occlusal contacts. Journal of Oral Rehabilitation. 2011;38(6):404-9. | Not relevant to orthodontics |
| 389 | Moncada G, Sepúlveda D, Elphick K, Contente M, Estay J, Bahamondes V, et al. Effects of light activation, agent concentration, and tooth thickness on dental sensitivity after bleaching. Oper Dent. 2013;38(5):467-76. | Not relevant to orthodontics |
| 390 | Mont MA, Cherian JJ, Bhave A, Starr R, Elmallah RK, Beaver WB, Jr., et al. Unloader Bracing for Knee Osteoarthritis: A Pilot Study of Gait and Function. Surg Technol Int. 2015;27:287-93. | Not relevant to orthodontics |
| 391 | Mosannen Mozafari P, Aboutorabzadeh SM, Azizi H, Khorasanchi M, Lotfinia Z, Motaghi S. Is laser acupuncture effective in controlling gag reflex while taking dental impressions? A randomized double-blinded sham-controlled clinical trial. J Evid Based Dent Pract. 2022;22(3):101733. | Not relevant to orthodontics |
| 392 | Müller CW, Gösling T, Mameghani A, Stier R, Klein M, Hüfner T, et al. Vertebral fractures due to osteoporosis - Kyphoplasty and vertebroplasty vs conservative treatment. Orthopade. 2010:1-7. | Not relevant to orthodontics |
| 393 | Müller-Rath R, Cho HY, Siebert CH, Miltner O. [Clinical and gait analytical investigation of valgus knee bracing in therapy for medial degenerative joint disease of the knee]. Z Orthop Unfall. 2011;149(2):160-5. | Not relevant to orthodontics |
| 394 | Munoz F, Salmochi JF, Faouën P, Rougier P. Low back pain sufferers: is standing postural balance facilitated by a lordotic lumbar brace? Orthop Traumatol Surg Res. 2010;96(4):362-6. | Not relevant to orthodontics |
| 395 | Muralidharan G, Prabhusankar K, Premkumar KS, Usha V, Muthuraman V. CLINICAL EVALUATION OF GELATAMP DENTAL DRESSING FOLLOWING DENTAL EXTRACTION IN PATIENTS UNDER ASPIRIN AND CLOPIDOGREL THERAPY-A RANDOMIZED CONTROL CLINICAL STUDY. NeuroQuantology. 2022;20(9):4469-78. | Not relevant to orthodontics |
| 396 | Murshid EZ. Children's ages and reasons for receiving their first dental visit in a Saudi community. Saudi Dental Journal. 2016;28(3):142-7. | Not relevant to orthodontics |
| 397 | Myung E, Domingos Neto J, Murta GA, Vieira A, de Lima PRG, Lessa L, et al. Diretriz Técnica da ANAMT (DT 05): prevenção de lombalgia ocupacional por uso de cinta, suporte ou órtese lombar. Rev bras med trab. 2018;16(4):524-31. | Not relevant to orthodontics |
| 398 | Nabecker S, Greif R, Kleine-Brueggeney M, Riggenbach C, Theiler L. Three unchannelled videolaryngoscopes and the macintosh laryngoscope in patients with a simulated difficult airway-a randomised controlled trial involving 480 patients. Swiss Medical Weekly. 2016;146:2S. | Not relevant to orthodontics |
| 399 | Naikmasur V, Bhargava P, Guttal K, Burde K. Soft occlusal splint therapy in the management of myofascial pain dysfunction syndrome: a follow-up study. Indian J Dent Res. 2008;19(3):196-203. | Not relevant to orthodontics |
| 400 | Nazem K, Mehrbod M, Borjian A, Sadeghian H. Anterior cruciate ligament reconstruction with or without bracing. Iranian Journal of Medical Sciences. 2006;31(3):151-5. | Not relevant to orthodontics |
| 401 | Negrini S, Minozzi S, Bettany-Saltikov J, Chockalingam N, Grivas TB, Kotwicki T, et al. Braces for Idiopathic Scoliosis in Adolescents. Spine (Phila Pa 1976). 2016;41(23):1813-25. | Not relevant to orthodontics |
| 402 | Nérot A, Nicholls M. Clinical study on the unloading effect of hip bracing on gait in patients with hip osteoarthritis. Prosthet Orthot Int. 2017;41(2):127-33. | Not relevant to orthodontics |
| 403 | Newell TM, Kumar S. Comparison of instantaneous and cumulative loads on the low back and neck in orthodontists. Clinical Biomechanics. 2005;20(2):130-7. | Not relevant to orthodontics |
| 404 | Ng GY, Chan HL. The immediate effects of tension of counterforce forearm brace on neuromuscular performance of wrist extensor muscles in subjects with lateral humeral epicondylosis. J Orthop Sports Phys Ther. 2004;34(2):72-8. | Not relevant to orthodontics |
| 405 | Ng SKS, Leung WK. Oral health-related quality of life and periodontal status. Community Dentistry and Oral Epidemiology. 2006;34(2):114-22. | Not relevant to orthodontics |
| 406 | Niazi NS, Niazi SNK, Niazi KNK, Siddique M, Iqbal M. Comparison of the effectiveness of knee braces and lateral wedge insole in the management of medial compartment knee osteoarthritis. Pakistan Journal of Medical and Health Sciences. 2014;8(1):37-40. | Not relevant to orthodontics |
| 407 | Nitecka-Buchta A, Buchta P, Tabeńska-Bosakowska E, Walczyńska-Dragoń K, Baron S. Myorelaxant effect of bee venom topical skin application in patients with RDC/TMD Ia and RDC/TMD Ib: a randomized, double blinded study. Biomed Res Int. 2014;2014:296053. | Not relevant to orthodontics |
| 408 | Nitecka-Buchta A, Marek B, Baron S. CGRP plasma level changes in patients with temporomandibular disorders treated with occlusal splints - A randomised clinical trial. Endokrynologia Polska. 2014;65(3):217-22. | Not relevant to orthodontics |
| 409 | Njio BJ, ter Heege GJ, Prahl-Andersen B. Quality development in a dental practice environment: A Web-based System for Measuring Patient Satisfaction. Journal of Orofacial Orthopedics. 2008;69(6):448-62. | Not relevant to orthodontics |
| 410 | Novaes TF, Matos R, Raggio DP, Imparato JC, Braga MM, Mendes FM. Influence of the discomfort reported by children on the performance of approximal caries detection methods. Caries Res. 2010;44(5):465-71. | Not relevant to orthodontics |
| 411 | Nowicka A, Lipski M, Parafiniuk M, Sporniak-Tutak K, Lichota D, Kosierkiewicz A, et al. Response of human dental pulp capped with biodentine and mineral trioxide aggregate. Journal of Endodontics. 2013;39(6):743-7. | Not relevant to orthodontics |
| 412 | Nunes AM, Fernandes P, Caetano AC, Sousa JM, Almeida RM, Consciência JG. Spinal fractures in ankylosing spondylitis patients: A systematic review of the literature. Global Spine Journal. 2017;7(2):105S. | Not relevant to orthodontics |
| 413 | Nuruzzaman KM, Mozaffar AS, Rahman KM, Salek AKM, Emran A. Effect of stretching and strengthening exercise in the management of Lateral Epicondylitis. International Journal of Rheumatic Diseases. 2017;20:29. | Not relevant to orthodontics |
| 414 | O'Connor D, Mullett H, Doyle M, Mofidi A, Kutty S, O'Sullivan M. Minimally displaced Colles' fractures: a prospective randomized trial of treatment with a wrist splint or a plaster cast. J Hand Surg Br. 2003;28(1):50-3. | Not relevant to orthodontics |
| 415 | Oghli I, List T, John MT, Häggman-Henrikson B, Larsson P. Prevalence and normative values for jaw functional limitations in the general population in Sweden. Oral Diseases. 2019;25(2):580-7. | Not relevant to orthodontics |
| 416 | Oh S, Gold BS, Varelas EA, Schwam ZG, Cosetti MK, Perez ER. Does Use of a Cervical Collar Improve Surgeon Neck Strain? A Simulation Pilot Study. Otolaryngology - Head and Neck Surgery. 2022;167(1):P164. | Not relevant to orthodontics |
| 417 | Oh SK, Kwon WK, Park S, Ji SG, Kim JH, Park YK, et al. Comparison of operating conditions, postoperative pain and recovery, and overall satisfaction of surgeons with deep vs. No neuromuscular blockade for spinal surgery under general anesthesia: A prospective randomized controlled trial. Journal of Clinical Medicine. 2019;8(4). | Not relevant to orthodontics |
| 418 | Ohmure H, Sakoguchi Y, Nagayama K, Numata M, Tsubouchi H, Miyawaki S. Influence of experimental oesophageal acidification on masseter muscle activity, cervicofacial behaviour and autonomic nervous activity in wakefulness. J Oral Rehabil. 2014;41(6):423-31. | Not relevant to orthodontics |
| 419 | Oken O, Kahraman Y, Ayhan F, Canpolat S, Yorgancioglu ZR, Oken OF. The short-term efficacy of laser, brace, and ultrasound treatment in lateral epicondylitis: a prospective, randomized, controlled trial. J Hand Ther. 2008;21(1):63-7; quiz 8. | Not relevant to orthodontics |
| 420 | Oleske DM, Lavender SA, Andersson GB, Kwasny MM. Are back supports plus education more effective than education alone in promoting recovery from low back pain?: Results from a randomized clinical trial. Spine (Phila Pa 1976). 2007;32(19):2050-7. | Not relevant to orthodontics |
| 421 | Oliveira FJd. Eficácia e segurança do laser cirúrgico de diodo em incisões circunvestibulares para osteotomia Le Fort I: ensaio clínico randomizado triplo cego. 2017. p. 94-. | Not relevant to orthodontics |
| 422 | Oliveira LKM, Carvalho LAdO, Assunção IVd, Borges BCD, Santos AJSd, Carvalho WLd, et al. Microabrasão na estética dentária: sucesso com procedimento minimamente invasivo. Rev Ciênc Plur. 2016;1(3):76-84. | Not relevant to orthodontics |
| 423 | Oliveira RdS, Flório FM, Oliveira AMG. Self-Perception of Brazilian Adolescents who need Orthodontic Treatment. Pesqui bras odontopediatria clín integr. 2018;18(1):3729-. | Not relevant to orthodontics |
| 424 | Olsson H, Davies JR, Holst KE, Schröder U, Petersson K. Dental pulp capping: effect of Emdogain Gel on experimentally exposed human pulps. Int Endod J. 2005;38(3):186-94. | Not relevant to orthodontics |
| 425 | Olthof NA, Coppieters MW, Moseley GL, Sterling M, Chippindall DJ, Harvie DS. Modernising tactile acuity assessment; Clinimetrics of semi-automated tests and effects of age, sex and anthropometry on performance. PeerJ. 2021;9. | Not relevant to orthodontics |
| 426 | Ostrander R, Leddon C, Hackel J, O'Grady C, Roth C. The efficacy of unloader bracing in reducing the pain and symptoms of knee osteoarthritis. Clinical Journal of Sport Medicine. 2014;24(2):169-70. | Not relevant to orthodontics |
| 427 | Otgonbayar M, Byambajav P, Byambasukh O, Jadamba T, Dagvajantsan B. The assessment of the severity of headaches caused by temporomandibular disorder. A descriptive analysis from the mon-timeline study. Journal of the Neurological Sciences. 2021;429. | Not relevant to orthodontics |
| 428 | Ozcelik O, Haytac MC, Seydaoglu G. Treatment of multiple gingival recessions using a coronally advanced flap procedure combined with button application. J Clin Periodontol. 2011;38(6):572-80. | Not relevant to orthodontics |
| 429 | Oziegbe EO, Esan TA, Adesina BA. Impact of oral conditions on the quality of life of secondary schoolchildren in Nigeria. Journal of Dentistry for Children. 2012;79(3):159-64. | Not relevant to orthodontics |
| 430 | Padwa BL, Dang RR, Resnick CM. Surgical Uprighting Is a Successful Procedure for Management of Impacted Mandibular Second Molars. Journal of Oral and Maxillofacial Surgery. 2017;75(8):1581-90. | Not relevant to orthodontics |
| 431 | Pagani C, Feitosa FA, Esteves SRMS, Miranda GMd, Antunes DP, Carvalho RFd. Dentinal Hypersensitivity: pre-hybridization as an alternative treatment. Braz dent sci. 2013;16(3):18-25. | Not relevant to orthodontics |
| 432 | Pandey V, Madi S, Maddukuri S, Acharya K, Karegowda LH, Willems WJ. Does application of abduction brace after arthroscopic rotator cuff repair improve blood flow around posterosuperior rotator cuff and repair site, affecting pain levels and clinical and structural outcomes? A pilot randomized controlled trial. JSES International. 2020;4(4):848-59. | Not relevant to orthodontics |
| 433 | Panneerselvam E, Balasubramanian S, Raja VBK, Kannan R, Rajaram K, Rajendra Sharma A. 'Plain lignocaine' vs 'Lignocaine with vasoconstrictor'-Comparative evaluation of pain during administration and post-extraction wound healing by a double blinded randomized controlled clinical trial. Acta Odontol Scand. 2016;74(5):374-9. | Not relevant to orthodontics |
| 434 | Parada F, Fonseca D, Palavecino F, Farías M, Hill S, Montero S. Manejo quirúrgico del paciente con hemofilia sometido a cirugía bucal: Reporte de un caso clínico. Odontol vital. 2020(33). | Not relevant to orthodontics |
| 435 | Parent EC, Schreiber S, Hedden D, Moreau M, Hill D, Watkins E. The effect of a 6-month Schroth exercise program: A pilot study using subjects as their own controls. Scoliosis. 2013;8. | Not relevant to orthodontics |
| 436 | Parirokh M, Rekabi AR, Ashouri R, Nakhaee N, Abbott PV, Gorjestani H. Effect of occlusal reduction on postoperative pain in teeth with irreversible pulpitis and mild tenderness to percussion. Journal of Endodontics. 2013;39(1):1-5. | Not relevant to orthodontics |
| 437 | Parkes M, Callaghan M, Felson D. Exploring the reasons for the sensitivity to change of a patient preference measure, compared with the KOOS questionnaire in patellofemoral osteoarthritis. Trials. 2015;16. | Not relevant to orthodontics |
| 438 | Parris WG, Tanzer FS, Fridland GH, Harris EF, Killmar J, Desiderio DM. Effects of orthodontic force on methionine enkephalin and substance P concentrations in human pulpal tissue. American Journal of Orthodontics and Dentofacial Orthopedics. 1989;95(6):479-89. | Not relevant to orthodontics |
| 439 | Paschos E, Huth KC, Benz C, Reeka-Bardschmidt A, Hickel R. Efficacy of intraoral topical anesthetics in children. J Dent. 2006;34(6):398-404. | Not relevant to orthodontics |
| 440 | Patel HS, Managutti AM, Menat S, Agarwal A, Shah D, Patel J. Comparative evaluation of efficacy of physics forceps versus conventional forceps in orthodontic extractions: A prospective randomized split mouth study. Journal of Clinical and Diagnostic Research. 2016;10(7):ZC41-ZC5. | Not relevant to orthodontics |
| 441 | Paterson KL, Bennell KL, Campbell PK, Metcalf BR, Kasza J, Wrigley TW, et al. Footwear for self-managing knee osteoarthritis symptoms: the footstep randomized controlled trial. Osteoarthritis and Cartilage. 2021;29:S57-S8. | Not relevant to orthodontics |
| 442 | Patil PG, Hazarey V, Chaudhari R, Nimbalkar-Patil S. A randomized control trial measuring the effectiveness of a mouth-exercising device for mucosal burning in oral submucous fibrosis. Oral Surg Oral Med Oral Pathol Oral Radiol. 2016;122(6):713-8. | Not relevant to orthodontics |
| 443 | Patil SR, De A, Datta D, Agrawal R, Reche A, Alam MK. Efficacy of fixed dose combination of chlorzoxazone, aceclofenac and paracetamol versus thiocolchicoside and aceclofenac in myofascial pain syndrome: A randomised clinical study. International Medical Journal. 2021;28(4):466-9. | Not relevant to orthodontics |
| 444 | Paul S, Choudhury R, Kumari N, Rastogi S, Sharma A, Singh V, et al. Is treatment with platelet-rich fibrin better than zinc oxide eugenol in cases of established dry socket for controlling pain, reducing inflammation, and improving wound healing? Journal of the Korean Association of Oral and Maxillofacial Surgeons. 2019;45(2):76-82. | Not relevant to orthodontics |
| 445 | Pavithra B, Ganapathy D, Jain AR. Effect of sublingual non-steroidal anti-inflammatory drugs - Ketorolac and piroxicam and its effectiveness in management of post extraction pain. Drug Invention Today. 2018;10(12):2415-8. | Not relevant to orthodontics |
| 446 | Payer M, Tan WC, Han J, Ivanovski S, Mattheos N, Pjetursson BE, et al. The effect of systemic antibiotics on clinical and patient-reported outcome measures of oral implant therapy with simultaneous guided bone regeneration. Clin Oral Implants Res. 2020;31(5):442-51. | Not relevant to orthodontics |
| 447 | Pegoraro LF, Conti PCR. Informações básicas sobre oclusão e disfunções craniomandibulares para o clínico geral. 2002. p. 64-76. | Not relevant to orthodontics |
| 448 | Peng J, Zhang X, Ren L, Huang J, Ning G, Gao Z, et al. Treatment and prognosis of osteoarthritis patients in China: a multicentre, large scale, real world study. Osteoarthritis and Cartilage. 2020;28:S385. | Not relevant to orthodontics |
| 449 | Pennant JH, Pace NA, Gajraj NM. Role of the laryngeal mask airway in the immobile cervical spine. J Clin Anesth. 1993;5(3):226-30. | Not relevant to orthodontics |
| 450 | Peres MFS, Ribeiro FV, Ruiz KGS, Nociti-Jr FH, Sallum EA, Casati MZ. Steroidal and non-steroidal cyclooxygenase-2 inhibitor anti-inflammatory drugs as pre-emptive medication in patients undergoing periodontal surgery. Brazilian Dental Journal. 2012;23(6):621-8. | Not relevant to orthodontics |
| 451 | Persson LC, Carlsson CA, Carlsson JY. Long-lasting cervical radicular pain managed with surgery, physiotherapy, or a cervical collar. A prospective, randomized study. Spine (Phila Pa 1976). 1997;22(7):751-8. | Not relevant to orthodontics |
| 452 | Petersen W, Ellermann A, Henning J, Nehrer S, Rembitzki IV, Fritz J, et al. Non-operative treatment of unicompartmental osteoarthritis of the knee: a prospective randomized trial with two different braces-ankle-foot orthosis versus knee unloader brace. Arch Orthop Trauma Surg. 2019;139(2):155-66. | Not relevant to orthodontics |
| 453 | Petersen W, Ellermann A, Rembitzki IV, Scheffler S, Herbort M, Brüggemann GP, et al. Evaluating the potential synergistic benefit of a realignment brace on patients receiving exercise therapy for patellofemoral pain syndrome: a randomized clinical trial. Arch Orthop Trauma Surg. 2016;136(7):975-82. | Not relevant to orthodontics |

| | | |
|---|---|---|
| 454 | Petersen W, Ellermann A, Rembitzki IV, Scheffler S, Herbort M, Sprenker FS, et al. The Patella Pro study - effect of a knee brace on patellofemoral pain syndrome: design of a randomized clinical trial (DRKS-ID:DRKS00003291). BMC Musculoskelet Disord. 2014;15:200. | Not relevant to orthodontics |
| 455 | Petersen W, Welp R, Rosenbaum D. Chronic Achilles tendinopathy: a prospective randomized study comparing the therapeutic effect of eccentric training, the AirHeel brace, and a combination of both. Am J Sports Med. 2007;35(10):1659-67. | Not relevant to orthodontics |
| 456 | Pettengill CA, Growney Jr MR, Schoff R, Kenworthy CR. A pilot study comparing the efficacy of hard and soft stabilizing appliances in treating patients with temporomandibular disorders. Journal of Prosthetic Dentistry. 1998;79(2):165-8. | Not relevant to orthodontics |
| 457 | Pfeifer M. A new study on osteoporosis shows that the spinomed® and Spinomed® active back braces improve posture and muscle strength. European Musculoskeletal Review. 2012;7(4):206-8. | Not relevant to orthodontics |
| 458 | Pförringer W, Kremer C. [Subsequent treatment of surgically managed, fresh, anterior cruciate ligament ruptures--a randomized, prospective study]. Sportverletz Sportschaden. 2005;19(3):134-9. | Not relevant to orthodontics |
| 459 | Phero JA, Nelson B, Davis B, Dunlop N, Phillips C, Reside G, et al. Buffered Versus Non-Buffered Lidocaine With Epinephrine for Mandibular Nerve Block: Clinical Outcomes. J Oral Maxillofac Surg. 2017;75(4):688-93. | Not relevant to orthodontics |
| 460 | Phero JA, Warren VT, Fisher AG, Rivera EM, Saha PT, Reside G, et al. Buffered 1% Lidocaine With Epinephrine Can Be as Effective as Nonbuffered 2% Lidocaine With Epinephrine for Maxillary Field Block. J Oral Maxillofac Surg. 2017;75(10):2071-5. | Not relevant to orthodontics |
| 461 | Phillips C, White Jr RP, Shugars DA, Zhou X. Risk Factors Associated with Prolonged Recovery and Delayed Healing after Third Molar Surgery. Journal of Oral and Maxillofacial Surgery. 2003;61(12):1436-48. | Not relevant to orthodontics |
| 462 | Phillips MF, Robertson Z, Killen B, White B. A pilot study of a crossover trial with randomized use of ankle-foot orthoses for people with Charcot-Marie-tooth disease. Clin Rehabil. 2012;26(6):534-44. | Not relevant to orthodontics |
| 463 | Piazzolla A, Bizzoca D, Balsano M, Moretti B. Capacitive biophysical stimulation in the treatment of vertebral compression fractures. European Spine Journal. 2017;26(4):1354. | Not relevant to orthodontics |
| 464 | Piazzolla A, Bizzoca D, Maccagnano G, Parato C, Moretti B. Capacitive coupling electric fields in the treatment of acute osteoporotic vertebral compression fractures: Preliminary results of a prospective randomized controlled multicenter study. European Spine Journal. 2018;27(4):947. | Not relevant to orthodontics |
| 465 | Piazzolla A, Solarino G, Bizzoca D, Dicuonzo F, Moretti B. Is vertebral bone marrow edema reduction related to clinical symptoms improvement in patients with acute vertebral compression fractures? A clinical-radiological study. Skeletal Radiology. 2017;46(6):852. | Not relevant to orthodontics |
| 466 | Pietropaoli D, Cooper BC, Ortu E, Monaco A. A Device Improves Signs and Symptoms of TMD. Pain Res Manag. 2019;2019:5646143. | Not relevant to orthodontics |
| 467 | Pisani MX, Malheiros-Segundo ADL, Balbino KL, Souza RDF, Paranhos HDFO, Lovato Da Silva CH. Oral health related quality of life of edentulous patients after denture relining with a silicone-based soft liner. Gerodontology. 2012;29(2):e474-e80. | Not relevant to orthodontics |
| 468 | Polo M. Botulinum toxin type a in the treatment of excessive gingival display. American Journal of Orthodontics and Dentofacial Orthopedics. 2005;127(2):214-8. | Not relevant to orthodontics |
| 469 | Pornratanarangsi S, Boonlert S, Duangprateep A, Wiratpintu P, Waree W, Tresukosol D, et al. The effectiveness of "Siriraj Leg Lock" brace on back pain after percutaneous coronary intervention: PCI. J Med Assoc Thai. 2010;93 Suppl 1:S35-42. | Not relevant to orthodontics |
| 470 | Potey AM, Kolte RA, Kolte AP, Mody D, Bodhare G, Pakhmode R. Coronally advanced flap with and without platelet-rich fibrin in the treatment of multiple adjacent recession defects: A randomized controlled split-mouth trial. Journal of Indian Society of Periodontology. 2019;23(5):436-41. | Not relevant to orthodontics |
| 471 | Pouchain EC, Costa FWG, Bezerra TP, Soares ECS. Comparative efficacy of nimesulide and ketoprofen on inflammatory events in third molar surgery: A split-mouth, prospective, randomized, double-blind study. International Journal of Oral and Maxillofacial Surgery. 2015;44(7):876-84. | Not relevant to orthodontics |
| 472 | Pouriafar N, Hedayati R, Bakhtiary A, Ghorbani R, Hajihasani A. Comparison of abdominal hollowing and bracing practices on the transverse abdominis muscle thickness in young women with non-specific low back pain. Koomesh. 2015;16(3):312-9. | Not relevant to orthodontics |
| 473 | Powell S. A comparison of two interventions in the treatment of severe ankle sprains and distal fibular avulsion fractures: A patient perspective. Emergency Medicine Journal. 2017;34(12):886-7. | Not relevant to orthodontics |
| 474 | Prado MP, Mendes AA, Amodio DT, Camanho GL, Smyth NA, Fernandes TD. A comparative, prospective, and randomized study of two conservative treatment protocols for first-episode lateral ankle ligament injuries. Foot Ankle Int. 2014;35(3):201-6. | Not relevant to orthodontics |
| 475 | Präger TM, Mischkowski RA, Zöller JE. Effect of intra-articular administration of buprenorphine after arthrocentesis of the temporomandibular joint: a pilot study. Quintessence Int. 2007;38(8):e484-9. | Not relevant to orthodontics |
| 476 | Prataap N, Sunil P, Sudeep C, Ninan V, Tom A, Arjun M. Platelet-rich plasma and incidence of alveolar osteitis in high-risk patients undergoing extractions of mandibular molars: A case-control study. Journal of Pharmacy and Bioallied Sciences. 2017;9(5):S173-S9. | Not relevant to orthodontics |
| 477 | Priore LB, Lack S, Garcia C, Azevedo FM, de Oliveira Silva D. Two Weeks of Wearing a Knee Brace Compared With Minimal Intervention on Kinesiophobia at 2 and 6 Weeks in People With Patellofemoral Pain: A Randomized Controlled Trial. Arch Phys Med Rehabil. 2020;101(4):613-23. | Not relevant to orthodontics |
| 478 | Quinelato V, Bonato LL, Vieira AR, Granjeiro JM, Tesch R, Casado PL. Association Between Polymorphisms in the Genes of Estrogen Receptors and the Presence of Temporomandibular Disorders and Chronic Arthralgia. J Oral Maxillofac Surg. 2018;76(2):314.e1-.e9. | Not relevant to orthodontics |
| 479 | Rabi A, Haris PMM, Panickal DM, Ahamed S, Pulikkottil VJ, Haris KTM. Comparative Evaluation of Two Different Flap Designs and Postoperative Outcome in the Surgical Removal of Impacted Mandibular Third Molar. J Contemp Dent Pract. 2017;18(9):807-11. | Not relevant to orthodontics |
| 480 | Radovich F, Clarich G, Vecchi R. The evaluation of anxiety and the analysis of pain perception in Down's syndrome patients undergoing dental procedures. Minerva stomatologica. 1991;40(11):701-9. | Not relevant to orthodontics |
| 481 | Radpasand M, Owens E. Combined multimodal therapies for chronic tennis elbow: pilot study to test protocols for a randomized clinical trial. J Manipulative Physiol Ther. 2009;32(7):571-85. | Not relevant to orthodontics |
| 482 | Raeissadat SA, Rayegani SM, Hassanabadi H, Rahimi R, Sedighipour L, Rostami K. Is platelet-rich plasma superior to whole blood in the management of chronic tennis elbow: One year randomized clinical trial. BMC Sports Science, Medicine and Rehabilitation. 2014;6(1). | Not relevant to orthodontics |
| 483 | Rafieian-Kopaei M, Hasanpour-Dehkordi A, Lorigooini Z, Deris F, Solati K, Mahdiyeh F. Comparing the effect of intranasal lidocaine 4% with peppermint essential oil drop 1.5% on migraine attacks: A double-blind clinical trial. International Journal of Preventive Medicine. 2019;10(1). | Not relevant to orthodontics |
| 484 | Raisch P, Jung MK, Vetter SY, Grutzner PA, Kreinest M. Analysis of the postoperative application of cervical collars in injuries to the subaxial cervical spine. European Spine Journal. 2021;30:3373. | Not relevant to orthodontics |
| 485 | Rämö L, Taimela S, Lepola V, Malmivaara A, Lähdeoja T, Paavola M. Open reduction and internal fixation of humeral shaft fractures versus conservative treatment with a functional brace: a study protocol of a randomised controlled trial embedded in a cohort. BMJ Open. 2017;7(7):e014076. | Not relevant to orthodontics |
| 486 | Rampello A, Saccucci M, Falisi G, Panti F, Polimeni A, Di Paolo C. A New Aid in Temporomandibular Joint Disorders' Therapy: The Universal Neuromuscular Immediate Relaxing Appliance. Journal of Biological Regulators and Homeostatic Agents. 2013;27(4):1011-9. | Not relevant to orthodontics |
| 487 | Regis D, Montanari M, Magnan B, Spagnol S, Bragantini A. Dynamic orthopaedic brace in the treatment of ankle sprains. Foot Ankle Int. 1995;16(7):422-6. | Not relevant to orthodontics |
| 488 | Replogle K, Reader A, Nist R, Beck M, Weaver J, Meyers WJ. Anesthetic efficacy of the intraosseous injection of 2% lidocaine (1:100,000 epinephrine) and 3% mepivacaine in mandibular first molars. Oral Surgery Oral Medicine Oral Pathology Oral Radiology and Endodontics. 1997;83(1):30-7. | Not relevant to orthodontics |
| 489 | Rezaeian ZS, Smith MM, Skaife TL, Harvey WF, Gross KD, Hunter DJ. Does knee malalignment predict the efficacy of realignment therapy for patients with knee osteoarthritis? Int J Rheum Dis. 2017;20(10):1403-12. | Not relevant to orthodontics |
| 490 | Richards JD, Sanchez-Ballester J, Jones RK, Darke N, Livingstone BN. A comparison of knee braces during walking for the treatment of osteoarthritis of the medial compartment of the knee. J Bone Joint Surg Br. 2005;87(7):937-9. | Not relevant to orthodontics |
| 491 | Richter A, Parent EC, Kawchuk G, Moreau M, Hedden D, Lou E. Ultrasound image measurements of erector spinae muscle thickness at four spinal levels in adolescents with idiopathic scoliosis: Reliability and concave-convex comparison. Scoliosis. 2013;8. | Not relevant to orthodontics |
| 492 | Risberg MA, Holm I, Steen H, Eriksson J, Ekeland A. The effect of knee bracing after anterior cruciate ligament reconstruction. A prospective, randomized study with two years' follow-up. Am J Sports Med. 1999;27(1):76-83. | Not relevant to orthodontics |
| 493 | Robbins SR, Melo LRS, Urban H, Deveza LA, Asher R, Johnson VL, et al. Effectiveness of Stepped-Care Intervention in Overweight and Obese Patients With Medial Tibiofemoral Osteoarthritis: A Randomized Controlled Trial. Arthritis Care Res (Hoboken). 2021;73(4):520-30. | Not relevant to orthodontics |
| 494 | Robert-Lachaine X, Dessery Y, Belzile É L, Turmel S, Corbeil P. Three-month efficacy of three knee braces in the treatment of medial knee osteoarthritis in a randomized crossover trial. J Orthop Res. 2020;38(10):2262-71. | Not relevant to orthodontics |
| 495 | Rønning R, Rønning I, Gerner T, Engebretsen L. The efficacy of wrist protectors in preventing snowboarding injuries. Am J Sports Med. 2001;29(5):581-5. | Not relevant to orthodontics |
| 496 | Rosas S, Paço M, Lemos C, Pinho T. Comparison between the Visual Analog Scale and the Numerical Rating Scale in the perception of esthetics and pain. International Orthodontics. 2017;15(4):543-60. | Not relevant to orthodontics |
| 497 | Rosenberg PA, Babick PJ, Schertzer L, Leung A. The effect of occlusal reduction on pain after endodontic instrumentation. Journal of endodontics. 1998;24(7):492-6. | Not relevant to orthodontics |
| 498 | Sadeghian R, Rohani B, Golestannejad Z, Sadeghian S, Mirzaee S. Comparison of therapeutic effect of mucoadhesive nano-triamcinolone gel and conventional triamcinolone gel on oral lichen planus. Dental Research Journal. 2019;16(5):277-82. | Not relevant to orthodontics |
| 499 | Saito K, Kihara H. A randomized controlled trial of the effect of 2-step orthosis treatment for a mallet finger of tendinous origin. J Hand Ther. 2016;29(4):433-9. | Not relevant to orthodontics |
| 500 | Salih SS. Assessment of therapeutic efficacy of wrist splint in the management of Carpal Tunnel Syndrome (CTS) literature review. Osteoporosis International. 2016;27(SUPPL 1):S106-S8. | Not relevant to orthodontics |
| 501 | Samani M, Shirazi ZR, Hadadi M, Sobhani S. A randomized controlled trial comparing the long-term use of soft lumbosacral orthoses at two different pressures in patients with chronic nonspecific low back pain. Clin Biomech (Bristol, Avon). 2019;69:87-95. | Not relevant to orthodontics |
| 502 | Sanborn RM, Badger GJ, Yen YM, Murray MM, Christino MA, Proffen B, et al. Psychological Readiness to Return to Sport at 6 Months Is Higher After Bridge-Enhanced ACL Restoration Than Autograft ACL Reconstruction: Results of a Prospective Randomized Clinical Trial. Orthopaedic Journal of Sports Medicine. 2022;10(2). | Not relevant to orthodontics |
| 503 | Santana-Mora U, Lopez-Raton M, Mora MJ, Cadarso-Suarez C, Lopez-Cedrun J, Santana-Penin U. Surface raw electromyography has a moderate discriminatory capacity for differentiating between healthy individuals and those with TMD: A diagnostic study. Journal of Electromyography and Kinesiology. 2014;24(3):332-40. | Not relevant to orthodontics |
| 504 | Santibáñez García JA, Ferrer Tamburini SA, Salinas Escoboza CRMG, Guizar Mendoza JM. Microtornillos como método de control vertical en desprogramación muscular. Rev ADM. 2020;77(5):261-6. | Not relevant to orthodontics |
| 505 | Santos CGd, Utsch PRC, Chagas AGF, Vasconcelos LdVS, Campos LR, Ali Hijazi M, et al. Prevalência de dor torácica e medicação antianginosa em Hospital Universitário de Vassouras, RJ. Rev bras cardiol (Impr). 2014;27(4):267-75. | Not relevant to orthodontics |
| 506 | Santos NM, Jordao MC, Ionta FQ, Mendonca FL, Lira Di Leone CC, Rabelo Buzalaf MA, et al. Impact of a simplified in situ protocol on enamel loss after erosive challenge. Plos One. 2018;13(5). | Not relevant to orthodontics |
| 507 | Santos PS, Martins-Júnior PA, Paiva SM, Klein D, Torres FM, Giacomin A, et al. Prevalence of self-reported dental pain and associated factors among eight- to ten-year-old Brazilian schoolchildren. PLoS One. 2019;14(4):e0214990. | Not relevant to orthodontics |
| 508 | Sato H, Fujii T, Yamada N, Kitamori H. Temporomandibular joint osteoarthritis: a comparative clinical and tomographic study pre- and post-treatment. Journal of Oral Rehabilitation. 1994;21(4):383-95. | Not relevant to orthodontics |
| 509 | Sato J, Segami N, Yoshitake Y, Kaneyama K, Yoshimura H, Fujimura K, et al. Specific expression of substance P in synovial tissues of patients with symptomatic, non-reducing internal derangement of the temporomandibular joint: Comparison with clinical findings. British Journal of Oral and Maxillofacial Surgery. 2007;45(5):372-7. | Not relevant to orthodontics |
| 510 | Satokawa Y, Minami I, Wakabayashi N. Short-term changes in chewing efficiency and subjective evaluation in normal dentate subjects after insertion of oral appliances with an occlusal flat table. Journal of Oral Rehabilitation. 2018;45(2):116-25. | Not relevant to orthodontics |
| 511 | Sattari S, Ashraf A. Comparison of the effects of 3-point valgus knee support and lateral wedge insoles in medial compartment knee osteoarthritis. Iranian Red Crescent Medical Journal. 2011;13(9):581-4. | Not relevant to orthodontics |
| 512 | Sayers MS, Cunningham SJ, Newton TJ. Patients' expectations: is there a typical patient? Journal of orthodontics. 2020;47(1):38-46. | Not relevant to orthodontics |
| 513 | Scharnweber B, Adjami F, Schuster G, Kopp S, Natrup J, Erbe C, et al. Influence of dental occlusion on postural control and plantar pressure distribution. Cranio-the Journal of Craniomandibular & Sleep Practice. 2017;35(6):358-66. | Not relevant to orthodontics |
| 514 | Schmitter M, Kress B, Leckel M, Henschel V, Ohlmann B, Rammelsberg P. Validity of temporomandibular disorder examination procedures for assessment of temporomandibular joint status. American Journal of Orthodontics and Dentofacial Orthopedics. 2008;133(6):796-803. | Not relevant to orthodontics |
| 515 | Schmitter M, Zahran M, Duc JM, Henschel V, Rammelsberg P. Conservative therapy in patients with anterior disc displacement without reduction using 2 common splints: a randomized clinical trial. J Oral Maxillofac Surg. 2005;63(9):1295-303. | Not relevant to orthodontics |
| 516 | Schreiber S, Parent EC, Hedden DM, Hill DL. Minimal important differences in Scoliosis Research Society-22r, spinal appearance questionnaire, Cobb angle, and Biering-Sorensen back muscle endurance test following a six-month Schroth exercises intervention in adolescents with idiopathic scoliosis. Scoliosis and Spinal Disorders. 2017;12. | Not relevant to orthodontics |
| 517 | Schreiber S, Parent EC, Hedden DM, Moreau M, Hill D, Watkins EM. The effects of a 6-month Schroth intervention for Adolescent Idiopathic Scoliosis (AIS): Preliminary analysis of an ongoing randomized controlled trial. Scoliosis. 2013;8. | Not relevant to orthodontics |
| 518 | Scott EJ, Anthony C, Willey MC, Lynch TS, Westermann RW. Perioperative Text Message Communication in Hip Arthroscopy: A Randomized-Controlled Trial. Arthroscopy - Journal of Arthroscopic and Related Surgery. 2021;37(1):e28. | Not relevant to orthodontics |
| 519 | Seeling W, Lotz P, Schröder M. [Postoperative pulmonary function following abdominal surgery. Comparison of continuous, segmental thoracic peridural anesthesia and intramuscular piritramide injections]. Anaesthesist. 1984;33(9):408-16. | Not relevant to orthodontics |
| 520 | Self BP, Greenwald RM, Pflaster DS. A biomechanical analysis of a medial unloading brace for osteoarthritis in the knee. Arthritis Care Res. 2000;13(4):191-7. | Not relevant to orthodontics |
| 521 | Selfe J, Richards J, Thewlis D, Kilmurray S. The biomechanics of step descent under different treatment modalities used in patellofemoral pain. Gait Posture. 2008;27(2):258-63. | Not relevant to orthodontics |
| 522 | Selfe J, Thewlis D, Hill S, Whitaker J, Sutton C, Richards J. A clinical study of the biomechanics of step descent using different treatment modalities for patellofemoral pain. Gait Posture. 2011;34(1):92-6. | Not relevant to orthodontics |
| 523 | Seligman DA, Pullinger AG. A multiple stepwise logistic regression analysis of trauma history and 16 other history and dental cofactors in females with temporomandibular disorders. Journal of Orofacial Pain. 1996;10(4):351-61. | Not relevant to orthodontics |
| 524 | Seligman E, Ryan L, Hang BT. Knee immobilizers for acute knee pain: A multicenter assessment of pediatric provider practices. Clinical Journal of Sport Medicine. 2019;29(2):143-4. | Not relevant to orthodontics |
| 525 | Sellbrant I, Blomstrand J, Karlsson J, Nellgård B, Jakobsson J. Brace versus cast following surgical treatment of distal radial fracture: a prospective randomised study comparing quality of recovery. F1000Res. 2021;10:336. | Not relevant to orthodontics |
| 526 | Şen S, Orhan G, Sertel S, Schmitter M, Schindler HJ, Lux CJ, et al. Comparison of acupuncture on specific and non-specific points for the treatment of painful temporomandibular disorders: A randomised controlled trial. J Oral Rehabil. 2020;47(7):783-95. | Not relevant to orthodontics |
| 527 | Şenel I, Metin M, Bereket C, Tek M, Arici S, Alkan A. Effects of different local haemostatic agents on facial swelling after the third molar surgery. Journal of Experimental and Clinical Medicine (Turkey). 2015;32(1):7-11. | Not relevant to orthodontics |

| | | |
|---|---|---|
| 528 | Shabat MA, Bede SY. Effect of the local application of bupivacaine in early pain control following impacted mandibular third molar surgery: A randomized controlled study. Dental and Medical Problems. 2021;58(4):483-8. | Not relevant to orthodontics |
| 529 | Shahakbari R, Eshghpour M, Rajaei A, Rezaei NM, Golfakhrabadi P, Nejat A. Effectiveness of green tea mouthwash in comparison to chlorhexidine mouthwash in patients with acute pericoronitis: a randomized clinical trial. Int J Oral Maxillofac Surg. 2014;43(11):1394-8. | Not relevant to orthodontics |
| 530 | Shamji MF, Roffey DM, Young DK, Reindl R, Wai EK. A pilot evaluation of the role of bracing in stable thoracolumbar burst fractures without neurological deficit. J Spinal Disord Tech. 2014;27(7):370-5. | Not relevant to orthodontics |
| 531 | Shankar D, Sinha A, Anand S, Verma N, Choudhary S. Efficacy of transdermal diclofenac patch and ketoprofen patch as postoperative analgesia after extraction of first premolars bilaterally in both arches for orthodontic purpose: A comparative study. Journal of Pharmacy and Bioallied Sciences. 2021;13(5):S101-S4. | Not relevant to orthodontics |
| 532 | Shankland WE. Nociceptive trigeminal inhibition--tension suppression system: a method of preventing migraine and tension headaches. Compend Contin Educ Dent. 2002;23(2):105-8, 10, 12-3; quiz 14. | Not relevant to orthodontics |
| 533 | Shariatzadeh H, Modaghegh BS, Mirzaei A. The effect of dynamic hyperextension brace on osteoporosis and hyperkyphosis reduction in postmenopausal osteoporotic women. Archives of Bone and Joint Surgery. 2017;5(3):181-5. | Not relevant to orthodontics |
| 534 | Sharifi R, Bahrami H, Safaei M, Mozaffari HR, Hatami M, Imani MM, et al. A Randomized Triple-Blind Clinical Trial of the Effect of Low-Level Laser Therapy on Infiltration Injection Pain in the Anterior Maxilla. Pesquisa Brasileira em Odontopediatria e Clinica Integrada. 2022;22. | Not relevant to orthodontics |
| 535 | Sharma NK, Loudon JK. Static progressive stretch brace as a treatment of pain and functional limitations associated with plantar fasciitis: a pilot study. Foot Ankle Spec. 2010;3(3):117-24. | Not relevant to orthodontics |
| 536 | Sheffer MAR, Corso AC, Tomazi MT, Bortoluzzi MCB. Condylar hyperplasia treated by simultaneous orthognathic surgery and high condylectomy: a case report. Rev odonto ciênc. 2008;23(4):407-10. | Not relevant to orthodontics |
| 537 | Shetty V, BhanuPrakash B, Yadav A, Kishore PN, Menon A. Do Regional Nerve Blocks Before Bimaxillary Surgery Reduce Postoperative Pain? J Oral Maxillofac Surg. 2020;78(5):724-30. | Not relevant to orthodontics |
| 538 | Shigli K, Angadi GS, Hegde P. The effect of remount procedures on patient comfort for complete denture treatment. Journal of Prosthetic Dentistry. 2008;99(1):66-72. | Not relevant to orthodontics |
| 539 | Shirani M, Mosharraf R, Shirany M. Comparisons of patient satisfaction levels with complete dentures of different occlusions: A randomized clinical trial. Journal of Prosthodontics. 2014;23(4):259-66. | Not relevant to orthodontics |
| 540 | Shivakumar KM, Chandu GN, Subba Reddy VV, Shafiulla MD. Prevalence of malocclusion and orthodontic treatment needs among middle and high school children of Davangere city, India by using Dental Aesthetic Index. Journal of Indian Society of Pedodontics and Preventive Dentistry. 2009;27(4):211-8. | Not relevant to orthodontics |
| 541 | Shrivastava S, Gurumurthy S, Doni B, Agrawal R, Patil SR, Ismail HS, et al. Efficacy of oil pulling in the management of oral submucous fibrosis: A preliminary study. Pesquisa Brasileira em Odontopediatria e Clinica Integrada. 2021;21. | Not relevant to orthodontics |
| 542 | Shubham S, Nepal M, Mishra R, Dutta K. Influence of maintaining apical patency in post-endodontic pain. BMC Oral Health. 2021;21(1):284. | Not relevant to orthodontics |
| 543 | Shukla D, Tripathi KP, Dhimole A, Singh D, Shukla C, Sharma A. Probiotics an Adjuvant in The Management of Recurrent Aphthous Ulcer: A Randomized Clinical Trial. European Journal of Molecular and Clinical Medicine. 2022;9(3):2488-93. | Not relevant to orthodontics |
| 544 | Siddique S. A risk assessment study on work-related musculoskeletal disorders among dentists in mangalore, India. Osteoporosis International. 2022;32(SUPPL 1):S144. | Not relevant to orthodontics |
| 545 | Siebenga J, Leferink VJ, Segers MJ, Elzinga MJ, Bakker FC, Haarman HJ, et al. Treatment of traumatic thoracolumbar spine fractures: a multicenter prospective randomized study of operative versus nonsurgical treatment. Spine (Phila Pa 1976). 2006;31(25):2881-90. | Not relevant to orthodontics |
| 546 | Siegert R, Gundlach KK. [Stabilizing splint versus relaxing appliances in the treatment of myofacial pain. Preliminary results of a prospective randomized study]. Dtsch Zahnarztl Z. 1989;44(11 Spec No):S17-9. | Not relevant to orthodontics |
| 547 | Silva Jr HVd, Gandini Jr LG, Amaral RMdP, Gandini MREAS. Parâmetros de força ideal aplicada aos principais movimentos ortodônticos. Ortodontia. 2010;43(4):418-24. | Not relevant to orthodontics |
| 548 | Silva M, Francisco I, Sanz D, Palmeira L, Vale F. Negative social comparisons and social discomfort in dentofacial deformity: A cross-sectional study. Minerva Dental and Oral Science. 2021;70(2):89-94. | Not relevant to orthodontics |
| 549 | Silva MEdSe, Ferreira RC, Resende VLS, Ferreira EFe, Magalhães CSd. Os limites da atenção primária no curso de odontologia da Universidade Federal de Minas Gerais. Rev odontol Univ Cid Sao Paulo. 2005;17(3):211-9. | Not relevant to orthodontics |
| 550 | Silva PG, Lombardi I, Jr., Breitschwerdt C, Poli Araújo PM, Natour J. Functional thumb orthosis for type I and II boutonniere deformity on the dominant hand in patients with rheumatoid arthritis: a randomized controlled study. Clin Rehabil. 2008;22(8):684-9. | Not relevant to orthodontics |
| 551 | Simmons Iii HC, Gibbs SJ. Initial TMJ disk recapture with anterior repositioning appliances and relation to dental history. Cranio - Journal of Craniomandibular and Sleep Practice. 1997;15(4):281-95. | Not relevant to orthodontics |
| 552 | Simmons Iii HC, Kilpatrick SR. A survey of dentists in the United States regarding a specialty in craniofacial pain. Cranio - Journal of Craniomandibular and Sleep Practice. 2004;22(1):72-6. | Not relevant to orthodontics |
| 553 | Simoneti LF, Weckwerth GM, Dionísio TJ, Torres EA, Zupelari-Gonçalves P, Calvo AM, et al. Efficacy of Ketoprofen With or Without Omeprazole for Pain And Inflammation Control After Third Molar Removal. Braz Dent J. 2018;29(2):140-9. | Not relevant to orthodontics |
| 554 | Simony A, Carreon LY, Christensen SB, Andersen MO. Health-related quality of life in adolescent idiopathic scoliosis patients 25 years after treatment. Spine Journal. 2015;15(10):175S. | Not relevant to orthodontics |
| 555 | Singh P, Pandey A, Singh A, Ahuja T, Sharma S, Bhagalia SR, et al. Efficacy of intralesional placental extract, dexamethasone and hyaluronidase in treatment of oral submucous fibrosis:a comparative study. JK Practitioner. 2016;21(1-2):29-34. | Not relevant to orthodontics |
| 556 | Singh PK, Alvi HA, Singh BP, Singh RD, Kant S, Jurel S, et al. Evaluation of various treatment modalities in sleep bruxism. J Prosthet Dent. 2015;114(3):426-31. | Not relevant to orthodontics |
| 557 | Singh V, Thepra M, Kirti S, Kumar P, Priya K. Dexmedetomidine as an Additive to Local Anesthesia: A Step to Development in Dentistry. Journal of Oral and Maxillofacial Surgery. 2018;76(10):2091.e1-.e7. | Not relevant to orthodontics |
| 558 | Sjögren A, Arnrup K, Jensen C, Knutsson I, Huggare J. Pain and fear in connection to orthodontic extractions of deciduous canines. Int J Paediatr Dent. 2010;20(3):193-200. | Not relevant to orthodontics |
| 559 | Slynarski K, Walawski J, Smigielski R, van der Merwe W. Feasibility of the Atlas Unicompartmental Knee System Load Absorber in Improving Pain Relief and Function in Patients Needing Unloading of the Medial Compartment of the Knee: 1-Year Follow-Up of a Prospective, Multicenter, Single-Arm Pilot Study (PHANTOM High Flex Trial). Clinical Medicine Insights: Arthritis and Musculoskeletal Disorders. 2017;10. | Not relevant to orthodontics |
| 560 | Smith V, Williams B, Stapleford R. Rigid internal fixation and the effects on the temporomandibular joint and masticatory system: A prospective study. American Journal of Orthodontics and Dentofacial Orthopedics. 1992;102(6):491-500. | Not relevant to orthodontics |
| 561 | Smits AJ, Deunk J, Stadhouder A, Altena MC, Kempen DHR, Bloemers FW. Is postoperative bracing after pedicle screw fixation of spine fractures necessary? Study protocol of the ORNOT study: a randomised controlled multicentre trial. BMJ Open. 2018;8(1):e019596. | Not relevant to orthodontics |
| 562 | Snijders E, Thomas S, Verhagen A. Assessment of the effectiveness of conservative treatment of acute ankle sprain. A review of the literature. Huisarts en Wetenschap. 2008;51(1):17-23. | Not relevant to orthodontics |
| 563 | Snyder M, Shugars DA, White RP, Phillips C. Pain Medication as an Indicator of Interference With Lifestyle and Oral Function During Recovery After Third Molar Surgery. Journal of Oral and Maxillofacial Surgery. 2005;63(8):1130-7. | Not relevant to orthodontics |
| 564 | Sobouti F, Rakhshan V, Chiniforush N, Khatami M. Effects of laser-assisted cosmetic smile lift gingivectomy on postoperative bleeding and pain in fixed orthodontic patients: a controlled clinical trial. Prog Orthod. 2014;15(1):66. | Not relevant to orthodontics |
| 565 | Sodhi N, Yao B, Anis HK, Khlopas A, Sultan AA, Newman JM, et al. Patient satisfaction and outcomes of static progressive stretch bracing: A 10-year prospective analysis. Annals of Translational Medicine. 2019;7(4). | Not relevant to orthodontics |
| 566 | Soliman HAG, Barchi S, Parent S, Maurais G, Jodoin A, Mac-Thiong JM. Early Impact of Postoperative Bracing on Pain and Quality of Life After Posterior Instrumented Fusion for Lumbar Degenerative Conditions: A Randomized Trial. Spine (Phila Pa 1976). 2018;43(3):155-60. | Not relevant to orthodontics |
| 567 | Song Z, Chen S, Zhang Y, Shi X, Zhao N, Liao Z. Lidocaine coinfusion alleviates vascular pain induced by hypertonic saline infusion: a randomized, placebo-controlled trial. BMC Anesthesiol. 2021;21(1):109. | Not relevant to orthodontics |
| 568 | Spratt KF, Weinstein JN, Lehmann TR, Woody J, Sayre H. Efficacy of flexion and extension treatments incorporating braces for low-back pain patients with retrodisplacement, spondylolisthesis, or normal sagittal translation. Spine (Phila Pa 1976). 1993;18(13):1839-49. | Not relevant to orthodontics |
| 569 | Ss A. A risk assessment study on work-related musculoskeletal disorders among dentists in mangalore, india. Osteoporosis International. 2019;30(SUPPL 2):S679. | Not relevant to orthodontics |
| 570 | Stadhouder A, Buskens E, Vergroesen DA, Fidler MW, de Nies F, Oner FC. Nonoperative treatment of thoracic and lumbar spine fractures: a prospective randomized study of different treatment options. J Orthop Trauma. 2009;23(8):588-94. | Not relevant to orthodontics |
| 571 | Stannard JP, Nuelle CW, McGwin G, Volgas DA. Hinged external fixation in the treatment of knee dislocations: a prospective randomized study. J Bone Joint Surg Am. 2014;96(3):184-91. | Not relevant to orthodontics |
| 572 | Steele L, Jacobs C, Conley C. COMPARISON OF TREATMENT PATTERNS AND DISEASE IMPACT BETWEEN THOSE WITH POSTTRAUMATIC VERSUS IDIOPATHIC KNEE OSTEOARTHRITIS. Osteoarthritis and Cartilage. 2022;30:S218-S9. | Not relevant to orthodontics |
| 573 | Storey P, Armstrong D, Dear H, Bradley M, Burke F. Pilot randomised controlled trial comparing C-Trac splints with Beta Wrist Braces for the management of carpal tunnel syndrome. Hand Therapy. 2013;18(2):35-41. | Not relevant to orthodontics |
| 574 | Stoustrup P, Twilt M, Koos B, Tzaribachev N, Herlin T, Pedersen T, et al. Temporomandibular pain in patients with Juvenile Idiopathic Arthritis. Journal of Rheumatology. 2016;43(6):1209-10. | Not relevant to orthodontics |
| 575 | Straub RK, Cipriani DJ. Influence of infrapatellar and suprapatellar straps on quadriceps muscle activity and onset timing during the body-weight squat. J Strength Cond Res. 2012;26(7):1827-37. | Not relevant to orthodontics |
| 576 | Struijs PA, Assendelft WJ, Kerkhoffs GM, Souer S, van Dijk CN. The predictive value of the extensor grip test for the effectiveness of bracing for tennis elbow. Am J Sports Med. 2005;33(12):1905-9. | Not relevant to orthodontics |
| 577 | Struijs PA, Kerkhoffs GM, Assendelft WJ, Van Dijk CN. Conservative treatment of lateral epicondylitis: brace versus physical therapy or a combination of both-a randomized clinical trial. Am J Sports Med. 2004;32(2):462-9. | Not relevant to orthodontics |
| 578 | Struijs PA, Korthals-de Bos IB, van Tulder MW, van Dijk CN, Bouter LM, Assendelft WJ. Cost effectiveness of brace, physiotherapy, or both for treatment of tennis elbow. Br J Sports Med. 2006;40(7):637-43; discussion 43. | Not relevant to orthodontics |
| 579 | Struijs PA, Spruyt M, Assendelft WJ, van Dijk CN. The predictive value of diagnostic sonography for the effectiveness of conservative treatment of tennis elbow. AJR Am J Roentgenol. 2005;185(5):1113-8. | Not relevant to orthodontics |
| 580 | Subirana Ferrés C, Salvador Villagrasa R, Díaz Martos I, Soriano Fernández I. Cuidados para el manejo seguro de los pacientes con espondilitis infecciosa. Metas enferm. 2019;22(5):67-75. | Not relevant to orthodontics |
| 581 | Suvinen TI, Reade PC, Könönen M, Kemppainen P. Vertical jaw separation and masseter muscle electromyographic activity: A comparative study between asymptomatic controls & patients with temporomandibular pain & dysfunction. Journal of Oral Rehabilitation. 2003;30(8):765-72. | Not relevant to orthodontics |
| 582 | Svernlöv B, Larsson M, Rehn K, Adolfsson L. Conservative treatment of the cubital tunnel syndrome. J Hand Surg Eur Vol. 2009;34(2):201-7. | Not relevant to orthodontics |
| 583 | Svitlana AS, Mani G. The influence of dental caries on young adults from their perspective. Journal of Pharmaceutical Sciences and Research. 2017;9(4):444-50. | Not relevant to orthodontics |
| 584 | Swaminathan V, Parkes MJ, Callaghan MJ, O'Neill TW, Hodgson R, Gait AD, et al. With a biomechanical treatment in knee osteoarthritis, less knee pain did not correlate with synovitis reduction. BMC Musculoskelet Disord. 2017;18(1):347. | Not relevant to orthodontics |
| 585 | Swenson EJ, Jr., DeHaven KE, Sebastianelli WJ, Hanks G, Kalenak A, Lynch JM. The effect of a pneumatic leg brace on return to play in athletes with tibial stress fractures. Am J Sports Med. 1997;25(3):322-8. | Not relevant to orthodontics |
| 586 | Swirtun LR, Jansson A, Renström P. The effects of a functional knee brace during early treatment of patients with a nonoperated acute anterior cruciate ligament tear: a prospective randomized study. Clin J Sport Med. 2005;15(5):299-304. | Not relevant to orthodontics |
| 587 | Tamaoki MJS, Matsunaga FT, Costa A, Netto NA, Matsumoto MH, Belloti JC. Treatment of Displaced Midshaft Clavicle Fractures: Figure-of-Eight Harness Versus Anterior Plate Osteosynthesis: A Randomized Controlled Trial. J Bone Joint Surg Am. 2017;99(14):1159-65. | Not relevant to orthodontics |
| 588 | Tan WC, Krishnaswamy G, Ong MMA, Lang NP. Patient-reported outcome measures after routine periodontal and implant surgical procedures. Journal of Clinical Periodontology. 2014;41(6):618-24. | Not relevant to orthodontics |
| 589 | Tanoue N, Nagano K, Yanamoto S, Mizuno A. Comparative evaluation of the breaking strength of a simple mobile mandibular advancement splint. European Journal of Orthodontics. 2009;31(6):620-4. | Not relevant to orthodontics |
| 590 | Tareen MK, Hhamad J, Mengal N, Warraich RA. Rationale of antibiotic therapy after surgical removal of asymptomatic impacted mandibular last molar. Pakistan Journal of Medical and Health Sciences. 2013;7(4):1190-1. | Not relevant to orthodontics |
| 591 | Tecco S, Caputi S, Teté S, Orsini G, Festa F. Intra-articular and muscle symptoms and subjective relief during TMJ internal derangement treatment with maxillary anterior repositioning splint or SVED and MORA splints: A comparison with untreated control subjects. Cranio. 2006;24(2):119-29. | Not relevant to orthodontics |
| 592 | Thakare A, Bhate K, Kathariya R. Comparison of 4% articaine and 0.5% bupivacaine anesthetic efficacy in orthodontic extractions: prospective, randomized crossover study. Acta Anaesthesiol Taiwan. 2014;52(2):59-63. | Not relevant to orthodontics |
| 593 | Thiruvevenkadam IA, Ling LT. Effect of cervical extensor strengthening on severity of temporomandibular joint disorder among university students: A randomized controlled trial. Research Journal of Pharmacy and Technology. 2021;14(4):2233-42. | Not relevant to orthodontics |
| 594 | Thomas GP, Throckmorton GS, Ellis Iii E, Sinn DP. The effects of orthodontic treatment on isometric bite forces and mandibular motion in patients before orthognathic surgery. Journal of Oral and Maxillofacial Surgery. 1995;53(6):673-8. | Not relevant to orthodontics |
| 595 | Thoumie P, Avouac B, Marty M, Pallez A. Efficacy and safety of rebel reliever® brace in patients with knee osteoarthritis e a phase iii randomized controlled trial. Osteoarthritis and Cartilage. 2017;25:S407. | Not relevant to orthodontics |
| 596 | Thoumie P, Marty M, Avouac B, Coudeyre E. Effectivenessofan unloading knee brace in the treatment of patients with knee osteoarthritis: A phase III randomized controlled trial. Osteoporosis International. 2017;28:S398-S9. | Not relevant to orthodontics |
| 597 | Thoumie P, Marty M, Avouac B, Pallez A, Vaumousse A, Pipet LPT, et al. Effect of unloading brace treatment on pain and function in patients with symptomatic knee osteoarthritis: the ROTOR randomized clinical trial. Sci Rep. 2018;8(1):10519. | Not relevant to orthodontics |
| 598 | Thumati P, Thumati RP, Poovani S, Sattur AP, Srinivas S, Kerstein RB, et al. A Multi-Center Disclusion Time Reduction (DTR) Randomized Controlled Occlusal Adjustment Study Using Occlusal Force and Timing Sensors Synchronized with Muscle Physiology Sensors. Sensors (Basel). 2021;21(23). | Not relevant to orthodontics |
| 599 | Timm KE. Randomized controlled trial of Protonics on patellar pain, position, and function. Med Sci Sports Exerc. 1998;30(5):665-70. | Not relevant to orthodontics |
| 600 | Tirefort J, Schwitzguebel AJ, Collin P, Nowak A, Plomb-Holmes C, Lädermann A. Postoperative Mobilization After Superior Rotator Cuff Repair: Sling Versus No Sling: A Randomized Prospective Study. J Bone Joint Surg Am. 2019;101(6):494-503. | Not relevant to orthodontics |
| 601 | Tirupathi SP, Rajasekhar S, Tummakomma P, Gangili AA, Khan ARA, Khurramuddin M, et al. Auto-controlled Syringe vs Insulin Syringe for Palatal Injections in Children: A Randomized Crossover Trial. J Contemp Dent Pract. 2020;21(6):604-8. | Not relevant to orthodontics |

| | | |
|---|---|---|
| 602 | Topcuoglu HS, Akpinar B. The effect of low-level laser therapy on the success rate of inferior alveolar nerve blocks in mandibular molars with symptomatic irreversible pulpitis: A randomized clinical trial. International Endodontic Journal. 2021;54(10):1720-6. | Not relevant to orthodontics |
| 603 | Topolski F. Avaliação da correlação entre dor e reações teciduais indesejadas da movimentação dentária induzida por diferentes tipos de força: estudo histomorfométrico, imunoistoquímico e comportamental. 2016. p. 54-. | Not relevant to orthodontics |
| 604 | Toye F, Williamson E, Williams M, Fairbank J, Lamb S. What matters to patients and their families in the management of adolescent idiopathic scoliosis? Spine Journal. 2016;16(4):S97. | Not relevant to orthodontics |
| 605 | Traistaru MR, Rogoveanu O, Popescu R. Benefits of rehabilitation program in patients with l5-s1 degenerative foraminal stenosis. Annals of the Rheumatic Diseases. 2013;72. | Not relevant to orthodontics |
| 606 | Tran J, Lou T, Nebiolo B, Castroflorio T, Tassi A, Cioffi I. Impact of clear aligner therapy on tooth pain and masticatory muscle soreness. Journal of Oral Rehabilitation. 2020;47(12):1521-9. | Not relevant to orthodontics |
| 607 | Tsukiyama Y, Baba K, Clark GT. An evidence-based assessment of occlusal adjustment as a treatment for temporomandibular disorders. Journal of Prosthetic Dentistry. 2001;86(1):57-66. | Not relevant to orthodontics |
| 608 | Tumia N, Wardlaw D, Hallett J, Deutman R, Mattsson SA, Sandén B. Aberdeen Colles' fracture brace as a treatment for Colles' fracture. A multicentre, prospective, randomised, controlled trial. J Bone Joint Surg Br. 2003;85(1):78-82. | Not relevant to orthodontics |
| 609 | Turk DC, Rudy TE, Kubinski JA, Zaki HS, Greco CM. Dysfunctional patients with temporomandibular disorders: evaluating the efficacy of a tailored treatment protocol. J Consult Clin Psychol. 1996;64(1):139-46. | Not relevant to orthodontics |
| 610 | Uckan S, Dayangac E, Araz K. Is permanent maxillary tooth removal without palatal injection possible? Oral Surgery Oral Medicine Oral Pathology Oral Radiology and Endodontics. 2006;102(6):733-5. | Not relevant to orthodontics |
| 611 | Uehara T, Tsushima E, Yamada S, Kimura S, Satsukawa Y, Yoshihara A, et al. A Randomized Controlled Trial for the Intervention Effect of Early Exercise Therapy on Axial Pain after Cervical Laminoplasty. Spine Surgery and Related Research. 2022;6(2):123-32. | Not relevant to orthodontics |
| 612 | Urquhart J, Alrehaili O, Fisher C, Fleming A, Rasoulinejad P, Gurr K, et al. Treatment of thoracolumbar burst fractures: extended follow-up of a randomized clinical trial comparing orthosis versus no orthosis. CMAJ Canadian Medical Association Journal. 2017;60(3):S70. | Not relevant to orthodontics |
| 613 | Vad VB, Bhat AL, Tarabichi Y. The role of the Back Rx exercise program in diskogenic low back pain: a prospective randomized trial. Arch Phys Med Rehabil. 2007;88(5):577-82. | Not relevant to orthodontics |
| 614 | Valladares Neto J, Santos CBd, Arruda BS, Gonçalves IMF. Skeletal class III malocclusion in conjunction with early childhood caries increases orthodontic treatment complexity: a case report. Rev Cient CRO-RJ (Online). 2018;3(2):72-7. | Not relevant to orthodontics |
| 615 | Valle CVMd. Avaliação dos efeitos do tratamento ortodôntico sobre limiares de dor, hábitos orais parafuncionais e qualidade de vida. 2016. p. 73-. | Not relevant to orthodontics |
| 616 | Vallon D, Nilner M, Söderfeldt B. Treatment Outcome in Patients with Craniomandibular Disorders of Muscular Origin: A 7-Year Follow-up. Journal of Orofacial Pain. 1998;12(3):210-8. | Not relevant to orthodontics |
| 617 | Vallon D. A longitudinal follow-up of the effect of occlusal adjustment in patients with craniomandibular disorders. Swedish Dental Journal. 1997;21(3):85-91. | Not relevant to orthodontics |
| 618 | Van De Streek MD, Van Der Schans CP, De Greef MH, Postema K. The effect of a forearm/hand splint compared with an elbow band as a treatment for lateral epicondylitis. Prosthet Orthot Int. 2004;28(2):183-9. | Not relevant to orthodontics |
| 619 | van den Berg C, Haak T, Weil NL, Hoogendoorn JM. Functional bracing treatment for stable type B ankle fractures. Injury. 2018;49(8):1607-11. | Not relevant to orthodontics |
| 620 | van Egmond N, van Grinsven S, van Loon CJ. Is There A Difference In Outcome Between Two Types Of Valgus Unloading Braces? A Randomized Controlled Trial. Acta Orthop Belg. 2017;83(4):690-9. | Not relevant to orthodontics |
| 621 | Van Outeren M, Waarsing E, Reijman M, Brouwer R, Verhaar J, Bierma-Zeinstra S. Is a high tibial osteotomy superior to usual care or valgus unloader bracing in patients with varus malaligned medial knee osteoarthritis? a propensity matched study using 2 RCT datasets. Osteoarthritis and Cartilage. 2016;24:S48. | Not relevant to orthodontics |
| 622 | van Poppel MN, Koes BW, van der Ploeg T, Smid T, Bouter LM. Lumbar supports and education for the prevention of low back pain in industry: a randomized controlled trial. Jama. 1998;279(22):1789-94. | Not relevant to orthodontics |
| 623 | van Raaij TM, Reijman M, Brouwer RW, Bierma-Zeinstra SM, Verhaar JA. Medial knee osteoarthritis treated by insoles or braces: a randomized trial. Clin Orthop Relat Res. 2010;468(7):1926-32. | Not relevant to orthodontics |
| 624 | Van Tiggelen D, Coorevits P, Bernard E, Thijs Y, Witvrouw E. The effects of 6-weeks patellofemoral bracing on quadriceps muscle function. Isokinetics and Exercise Science. 2011;19(3):169-73. | Not relevant to orthodontics |
| 625 | Van Tiggelen D, Witvrouw E, Roget P, Cambier D, Danneels L, Verdonk R. Effect of bracing on the prevention of anterior knee pain--a prospective randomized study. Knee Surg Sports Traumatol Arthrosc. 2004;12(5):434-9. | Not relevant to orthodontics |
| 626 | Varadharaja M, Udhya J, Srinivasan I, Sivakumar JSK, Karthik RS, Manivanan M. Comparative clinical evaluation of transcutaneous electrical nerve stimulator over conventional local anesthesia in children seeking dental procedures: A clinical study. Journal of Pharmacy and Bioallied Sciences. 2014;6:S113-S7. | Not relevant to orthodontics |
| 627 | Veeroo HJ, Cunningham SJ, Newton JT, Travess HC. Motivation and compliance with intraoral elastics. Am J Orthod Dentofacial Orthop. 2014;146(1):33-9. | Not relevant to orthodontics |
| 628 | Vidigal EA, Abanto J, Leyda AM, Berti GO, Aillón IEV, Corrêa M, et al. Comparison of two behavior management techniques used during mandibular block anesthesia among preschool children: a randomized clinical trial. Eur Arch Paediatr Dent. 2021;22(5):773-81. | Not relevant to orthodontics |
| 629 | Vilardi TMR. Anti-inflamatórios não esteroidais e sua interferência na movimentação dentária induzida em ratos. 2011. p. 119-. | Not relevant to orthodontics |
| 630 | Vitale MC, Modaffari C, Decembrino N, Zhou FX, Zecca M, Defabianis P. Preliminary study in a new protocol for the treatment of oral mucositis in pediatric patients undergoing hematopoietic stem cell transplantation (HSCT) and chemotherapy (CT). Lasers Med Sci. 2017;32(6):1423-8. | Not relevant to orthodontics |
| 631 | Vitus D, Williams MK, Rizk M, Neubert JK, Robinson M, Boissoneault J. Analgesic effects of alcohol in adults with chronic jaw pain. Alcohol Clin Exp Res. 2022;46(8):1515-24. | Not relevant to orthodontics |
| 632 | Von Feldt JM. Managing osteoporotic fractures: Minimizing pain and disability. Journal of Clinical Rheumatology. 1997;3(2 SUPPL.):S65-S8. | Not relevant to orthodontics |
| 633 | Wagshall E, Lewis Z, Babich SB, Sinensky MC, Hochberg M. Acellular dermal matrix allograft in the treatment of mucogingival defects in children: Illustrative case report. Journal of Dentistry for Children. 2002;69(1):39-43. | Not relevant to orthodontics |
| 634 | Wahlund K, List T, Larsson B. Treatment of temporomandibular disorders among adolescents: A comparison between occlusal appliance, relaxation training, and brief information. Acta Odontologica Scandinavica. 2003;61(4):203-11. | Not relevant to orthodontics |
| 635 | Walczyńska-Dragon K, Baron S, Nitecka-Buchta A, Tkacz E. Correlation between TMD and cervical spine pain and mobility: is the whole body balance TMJ related? Biomed Res Int. 2014;2014:582414. | Not relevant to orthodontics |
| 636 | Walther M, Werner A, Stahlschmidt T, Woelfel R, Gohlke F. The subacromial impingement syndrome of the shoulder treated by conventional physiotherapy, self-training, and a shoulder brace: results of a prospective, randomized study. J Shoulder Elbow Surg. 2004;13(4):417-23. | Not relevant to orthodontics |
| 637 | Wang C, Zhou X, Chen Y, Zhang J, Chen W, Svensson P, et al. Somatosensory profiling of patients with plaque-induced gingivitis: a case-control study. Clin Oral Investig. 2020;24(2):875-82. | Not relevant to orthodontics |
| 638 | Warren VT, Fisher AG, Rivera EM, Saha PT, Turner B, Reside G, et al. Buffered 1% Lidocaine With Epinephrine Is as Effective as Non-Buffered 2% Lidocaine With Epinephrine for Mandibular Nerve Block. J Oral Maxillofac Surg. 2017;75(7):1363-6. | Not relevant to orthodontics |
| 639 | Weffort SYK, Reis CMS, Fantini SMd. Comparação dos registros de RC obtidos pelos métodos de manipulação uni e bimanual - estudo preliminar. Ortodontia. 2006;39(3):221-30. | Not relevant to orthodontics |
| 640 | Weggen T, Schindler HJ, Kordass B, Hugger A. Clinical and electromyographic follow-up of myofascial pain patients treated with two types of oral splint: a randomized controlled pilot study. Int J Comput Dent. 2013;16(3):209-24. | Not relevant to orthodontics |
| 641 | White A. Acupuncture for pelvic girdle pain of pregnancy (n=386). Acupuncture in Medicine. 2005;23(2):86-7. | Not relevant to orthodontics |
| 642 | Więckiewicz W, Woźniak K, Piątkowska D, Szyszka-Sommerfeld L, Lipski M. The diagnostic value of pressure algometry for temporomandibular disorders. Biomed Res Int. 2015;2015:575038. | Not relevant to orthodontics |
| 643 | Wilson JJ, Husom E, McGuine TA, Hetzel S. The effect of a knee unloader brace for the treatment of knee osteoarthritis in patients receiving viscosupplementation injections. Clinical Journal of Sport Medicine. 2014;24(2):166. | Not relevant to orthodontics |
| 644 | Wilson S, Ngan P, Kess B. Time course of the discomfort in young patients undergoing orthodontic treatment. Pediatric dentistry. 1989;11(2):107-10. | Not relevant to orthodontics |
| 645 | Witjes S, Gresnigt F, van den Bekerom MP, Olsman JG, van Dijk NC. The ANKLE TRIAL (ankle treatment after injuries of the ankle ligaments): what is the benefit of external support devices in the functional treatment of acute ankle sprain? A randomised controlled trial. BMC Musculoskelet Disord. 2012;13:21. | Not relevant to orthodontics |
| 646 | Wolford LM, Mehra P, Reiche-Fischel O, Morales-Ryan CA, García-Morales P. Efficacy of high condylectomy for management of condylar hyperplasia. American Journal of Orthodontics and Dentofacial Orthopedics. 2002;121(2):136-51. | Not relevant to orthodontics |
| 647 | Worrell TW, Ingersoll CD, Farr J. Effect of patellar taping and bracing on patellar position: An MRI case study. Journal of Sport Rehabilitation. 1994;3(2):146-53. | Not relevant to orthodontics |
| 648 | Wyndow N, Collins N, Vincenzo B, Tucker K, Crossley K. The effect of footwear and foot orthoses on patellofemoral bone marrow lesion volume in individuals with patellofemoral osteoarthritis. Osteoarthritis and Cartilage. 2020;28:S397-S8. | Not relevant to orthodontics |
| 649 | Xu D, Wang P, Liu H, Gu M. Efficacy of three surgical methods for gingivectomy of permanent anterior teeth with delayed tooth eruption in children. Head and Face Medicine. 2022;18(1). | Not relevant to orthodontics |
| 650 | Yagci G, Yakut Y. Core stabilization exercises versus scoliosis-specific exercises in moderate idiopathic scoliosis treatment. Prosthet Orthot Int. 2019;43(3):301-8. | Not relevant to orthodontics |
| 651 | Yang W, Liu W, Miao C, Sun H, Li L, Li C. Oral Glucosamine Hydrochloride Combined With Hyaluronate Sodium Intra-Articular Injection for Temporomandibular Joint Osteoarthritis: A Double-Blind Randomized Controlled Trial. Journal of Oral and Maxillofacial Surgery. 2018;76(10):2066-73. | Not relevant to orthodontics |
| 652 | Yano K, Sano T, Okano T. A longitudinal study of Magnetic Resonance (MR) evidence of Temporomandibular Joint (TMJ) fluid in patients with TMJ disorders. Cranio - Journal of Craniomandibular and Sleep Practice. 2004;22(1):64-71. | Not relevant to orthodontics |
| 653 | Yao E, Gerritz PK, Henricson E, Abresch T, Kim J, Han J, et al. Randomized controlled trial comparing acupuncture with placebo acupuncture for the treatment of carpal tunnel syndrome. Pm r. 2012;4(5):367-73. | Not relevant to orthodontics |
| 654 | Yassen GH. Evaluation of mandibular infiltration versus mandibular block anaesthesia in treating primary canines in children. Int J Paediatr Dent. 2010;20(1):43-9. | Not relevant to orthodontics |
| 655 | Yavari HR, Jafari F, Jamloo H, Hallaj-Nezhadi S, Jafari S. The Effect of Submucosal Injection of Corticosteroids on Pain Perception and Quality of Life after Root Canal Treatment of Teeth with Irreversible Pulpitis: A Randomized Clinical Trial. J Endod. 2019;45(5):477-82. | Not relevant to orthodontics |
| 656 | Yee AJ, Yoo JU, Marsolais EB, Carlson G, Poe-Kochert C, Bohlman HH, et al. Use of a postoperative lumbar corset after lumbar spinal arthrodesis for degenerative conditions of the spine. A prospective randomized trial. J Bone Joint Surg Am. 2008;90(10):2062-8. | Not relevant to orthodontics |
| 657 | Yoon JY, Park JH, Lee KJ, Kim HS, Rhee SM, Oh JH. The effect of postoperatively applied far-infrared radiation on pain and tendon-to-bone healing after arthroscopic rotator cuff repair: a clinical prospective randomized comparative study. Korean Journal of Pain. 2020;33(4):344-51. | Not relevant to orthodontics |
| 658 | Yu SP, Williams M, Eyles JP, Chen JS, Makovey J, Hunter DJ. Effectiveness of knee bracing in osteoarthritis: pragmatic trial in a multidisciplinary clinic. Int J Rheum Dis. 2016;19(3):279-86. | Not relevant to orthodontics |
| 659 | Yue Yi EK, Siew Ying AL, Mohan M, Menon RK. Prevalence of Postoperative Infection after Tooth Extraction: A Retrospective Study. International Journal of Dentistry. 2021;2021. | Not relevant to orthodontics |
| 660 | Zahednejad S, Goharpey S, Farokhnia M. Comparison of patellar taping versus patellar bracing with exercise therapy on pain and level of function in females with patellofemoral pain syndrome. Koomesh. 2017;19(3):677-87. | Not relevant to orthodontics |
| 661 | Zain M, Rehman Khattak SU, Sikandar H, Shah SA, Fayyaz. Comparison of Anaesthetic Efficacy of 4% Articaine Primary Buccal Infiltration Versus 2% Lidocaine Inferior Alveolar Nerve Block in Symptomatic Mandibular First Molar Teeth. J Coll Physicians Surg Pak. 2016;26(1):4-8. | Not relevant to orthodontics |
| 662 | Zhang J, Troulis MJ, August M. Diagnosis and Treatment of Pediatric Primary Jaw Lesions at Massachusetts General Hospital. Journal of Oral and Maxillofacial Surgery. 2021;79(3):585-97. | Not relevant to orthodontics |
| 663 | Zhang N, Li Y, Zhou R. Comparison of Single-Person Laparoscopic Appendectomy Using a Novel Brace-Assisted Camera Holding System and Conventional Laparoscopic Appendectomy: A Neural Network Algorithm Analysis. Contrast Media and Molecular Imaging. 2022;2022. | Not relevant to orthodontics |
| 664 | Zhang Y, Zhang J, Wang L, Wang K, Svensson P. Effect of transcutaneous electrical nerve stimulation on jaw movement-evoked pain in patients with TMJ disc displacement without reduction and healthy controls. Acta Odontol Scand. 2020;78(4):309-20. | Not relevant to orthodontics |
| 665 | Zhou Y, Long H, Ye N, Liao L, Yang X, Jian F, et al. The effect of capsaicin on expression patterns of CGRP in trigeminal ganglion and trigeminal nucleus caudalis following experimental tooth movement in rats. J appl oral sci. 2016;24(6):597-606. | Not relevant to orthodontics |
| 666 | Zoia C, Bongetta D, Alicino C, Chimenti M, Pugliese R, Gaetani P. Usefulness of corset adoption after single-level lumbar discectomy: a randomized controlled trial. J Neurosurg Spine. 2018;28(5):481-5. | Not relevant to orthodontics |
| 667 | Aarab G, Lobbezoo F, Hamburger HL, Naeije M. Effects of an oral appliance with different mandibular protrusion positions at a constant vertical dimension on obstructive sleep apnea. Clin Oral Investig. 2010;14(3):339-45. | Non-healthy patients |
| 668 | Akcam MO, Altio T, Özdiler FE. Functional analysis of cleft lip and palate patients with modified Rakosi method. Cleft Palate-Craniofacial Journal. 2002;39(1):101-4. | Non-healthy patients |
| 669 | Baeshen HA, Alshahrani A, Kamran MA, Alnazeh AA, Alhaizaey A, Alshahrani I. Effectiveness of antimicrobial photodynamic therapy in restoring clinical, microbial, proinflammatory cytokines and pain scores in adolescent patients having generalized gingivitis and undergoing fixed orthodontic treatment. Photodiagnosis Photodyn Ther. 2020;32:101998. | Non-healthy patients |
| 670 | Barros LAN, Jesuino FAS, de Paiva JB, Rino-Neto J, Valladares-Neto J. An Oral Health-Related Quality of Life Comparison Between Adults With Unilateral Cleft Lip and Palate and Class III Malocclusion. Cleft Palate-Craniofacial Journal. 2019;56(10):1359-65. | Non-healthy patients |
| 671 | Clark GT, Blumenfeld I, Yoffe N, Peled E, Lavie P. A crossover study comparing the efficacy of continuous positive airway pressure with anterior mandibular positioning devices on patients with obstructive sleep apnea. Chest. 1996;109(6):1477-83. | Non-healthy patients |
| 672 | Cooke ME, Battagel JM. A thermoplastic mandibular advancement device for the management of non-apnoeic snoring: a randomized controlled trial. Eur J Orthod. 2006;28(4):327-38. | Non-healthy patients |
| 673 | Cunali PA, Almeida FR, Santos CD, Valdrichi NY, Nascimento LS, Dal-Fabbro C, et al. Mandibular exercises improve mandibular advancement device therapy for obstructive sleep apnea. Sleep Breath. 2011;15(4):717-27. | Non-healthy patients |
| 674 | Gong X, Zhang J, Zhao Y, Gao X. Long-term therapeutic efficacy of oral appliances in treatment of obstructive sleep apnea-hypopnea syndrome. Angle Orthodontist. 2013;83(4):653-8. | Non-healthy patients |
| 675 | Isacsson G, Fodor C, Sturebrand M. Obstructive sleep apnea treated with custom-made bibloc and monobloc oral appliances: a retrospective comparative study. Sleep Breath. 2017;21(1):93-100. | Non-healthy patients |
| 676 | Isacsson G, Nohlert E, Fransson A, Wiman Eriksson E, Ortlieb E, Fodor C, et al. Bibloc and monobloc oral appliances in the treatment of obstructive sleep apnoea: A multicenter, randomized, blinded, parallel-group trial. Sleep Medicine. 2017;40:e142-e3. | Non-healthy patients |
| 677 | Isacsson G, Nohlert E, Fransson AMC, Bornefalk-Hermansson A, Wiman Eriksson E, Ortlieb E, et al. Use of bibloc and monobloc oral appliances in obstructive sleep apnoea: a multicentre, randomized, blinded, parallel-group equivalence trial. Eur J Orthod. 2019;41(1):80-8. | Non-healthy patients |
| 678 | Johal A, Battagel J, Hector M. Controlled, prospective trial of psychosocial function before and after mandibular advancement splint therapy. American Journal of Orthodontics and Dentofacial Orthopedics. 2011;139(5):581-7. | Non-healthy patients |
| 679 | Kim JS, Chen W, Grunwaldt L, Losee JE, Bise C, Schuster L. Musculoskeletal Pain Survey Outcomes in Cleft Surgeons and Orthodontists. Cleft Palate-Craniofacial Journal. 2021;58(2):222-9. | Non-healthy patients |
| 680 | Kwon JS, Jung HJ, Yu JH, Bae SY, Park Y, Cha JY, et al. Effectiveness of remote monitoring and feedback on objective compliance with a mandibular advancement device for treatment of obstructive sleep apnea. J Sleep Res. 2022;31(3):e13508. | Non-healthy patients |

| | | |
|---|---|---|
| 681 | Landry ML, Rompré PH, Manzini C, Guitard F, de Grandmont P, Lavigne GJ. Reduction of sleep bruxism using a mandibular advancement device: an experimental controlled study. Int J Prosthodont. 2006;19(6):549-56. | Non-healthy patients |
| 682 | Landry-Schoenbeck A, de Grandmont P, Rompre PH, Lavigne GJ. Effect of an Adjustable Mandibular Advancement Appliance on Sleep Bruxism: A Crossover Sleep Laboratory Study. International Journal of Prosthodontics. 2009;22(3):251-9. | Non-healthy patients |
| 683 | Martínez-Gomis J, Willaert E, Nogues L, Pascual M, Somoza M, Monasterio C. Five years of sleep apnea treatment with a mandibular advancement device side effects and technical complications. Angle Orthodontist. 2010;80(1):30-6. | Non-healthy patients |
| 684 | Meazzini MC, Tortora C, Mazzoleni F, Autelitano L. Comparison of Pain Perception in Patients Affected by Cleft and Cranio Facial Anomalies Treated With Traditional Fixed Appliances or Invisalign. Cleft Palate-Craniofacial Journal. 2020;57(1):35-42. | Non-healthy patients |
| 685 | Mohsenin N, Mostofi MT, Mohsenin V. The role of oral appliances in treating obstructive sleep apnea. Journal of the American Dental Association. 2003;134(4):442-9+98. | Non-healthy patients |
| 686 | Peng Y, Tang S. The Factors Affecting Orthodontic Pain with Periodontitis. J Healthc Eng. 2021;2021:8942979. | Non-healthy patients |
| 687 | Prathibha BN, Jagger RG, Saunders M, Smith AP. Use of a mandibular advancement device in obstructive sleep apnoea. Journal of Oral Rehabilitation. 2003;30(5):507-9. | Non-healthy patients |
| 688 | Quintela MdM, Lima Júnior A, Sallum RA, Pacheco Filho F, Flório FM, Motta RHL. Avaliação da eficácia de um aparelho de avanço mandibular semiflexível na apneia obstrutiva do sono: estudo clínico e polissonográfico. Ortodontia. 2013;46(4):358-69. | Non-healthy patients |
| 689 | Ren C, McGrath C, Gu M, Jin L, Zhang C, Sum F, et al. Low-level laser-aided orthodontic treatment of periodontally compromised patients: a randomised controlled trial. Lasers Med Sci. 2020;35(3):729-39. | Non-healthy patients |
| 690 | Saletu A, Parapatics S, Saletu B, Anderer P, Prause W, Putz H, et al. On the pharmacotherapy of sleep bruxism: Placebo-controlled polysomnographic and psychometric studies with clonazepam. Neuropsychobiology. 2005;51(4):214-25. | Non-healthy patients |
| 691 | Saueressig AC, Mainieri VC, Grossi PK, Fagondes SC, Shinkai RSA, Lima EM, et al. Analysis of the influence of a mandibular advancement device on sleep and sleep bruxism scores by means of the bitestrip and the sleep assessment questionnaire. International Journal of Prosthodontics. 2010;23(3):204-13. | Non-healthy patients |
| 692 | Smith AM, Battagel JM. Non-apneic snoring and the orthodontist: The effectiveness of mandibular advancement splints. Journal of Orthodontics. 2004;31(2):115-23. | Non-healthy patients |
| 693 | Tegelberg A, Wilhelmsson B, Walker-Engström ML, Ringqvist M, Andersson L, Krekmanov L, et al. Effects and adverse events of a dental appliance for treatment of obstructive sleep apnoea. Swed Dent J. 1999;23(4):117-26. | Non-healthy patients |
| 694 | Thuaksuban N, Nuntanaranont T, Pripatnanont P. A comparison of autogenous bone graft combined with deproteinized bovine bone and autogenous bone graft alone for treatment of alveolar cleft. Int J Oral Maxillofac Surg. 2010;39(12):1175-80. | Non-healthy patients |
| 695 | Almallah MME, Almahd WH, Hajeer MY. Evaluation of the use of low-level laser therapy in pain control in orthodontic patients: A randomized split-mouth clinical trial. Journal of Clinical and Diagnostic Research. 2016;10(11):ZC23-ZC8. | Non-clinical study |
| 696 | Ballesteros RE, Viáfara J, Domínguez Á. Efecto del laser de baja intensidad en el tejido pulpar durante el movimiento ortodóncico. Rev estomat salud. 2012;20(1):30-8. | Non-clinical study |
| 697 | Boyd RL, Waskalic V. Three-dimensional diagnosis and orthodontic treatment of complex malocclusions with the invisalign appliance. Seminars in Orthodontics. 2001;7(4):274-93. | Non-clinical study |
| 698 | Bräscher AK, Zuran D, Feldmann RE, Jr., Benrath J. Patient survey on Invisalign® treatment comparen the SmartTrack® material to the previous aligner material. Journal of Orofacial Orthopedics. 2016;77(6):432-8. | Non-clinical study |
| 699 | Brasileiro BF, De Bragana RMF, Van Sickels JE. An evaluation of patients' knowledge about perioperative information for third molar removal. Journal of Oral and Maxillofacial Surgery. 2012;70(1):12-8. | Non-clinical study |
| 700 | Brignardello-Petersen R. Pulsed electromagnetic field seems to decrease pain levels in females from 24 through 72 hours after initial archwire placement. Journal of the American Dental Association. 2018;149(4):e75. | Non-clinical study |
| 701 | Buttke TM, Proffit WR. Referring adult patients for orthodontic treatment. Journal of the American Dental Association. 1999;130(1):73-9. | Non-clinical study |
| 702 | Fricton JR, Ouyang W, Nixdorf DR, Schiffman EL, Velly AM, Look JO. Critical appraisal of methods used in randomized controlled trials of treatments for temporomandibular disorders. Journal of Oral and Facial Pain and Headache. 2010;24(2):139-51. | Non-clinical study |
| 703 | Holmberg F, Muñoz J, Holmberg F, Cordova P, Sandoval P. Uso del láser terapéutico en el control del dolor en ortodoncia. Int j odontostomatol (Print). 2010;4(1):43-6. | Non-clinical study |
| 704 | León P, Domínguez Á. Laserterapia y marcadores bioquímicos en la aceleración del movimiento dental ortodóncico: revisión de la literatura. Rev estomat salud. 2013;21(2):14-9. | Non-clinical study |
| 705 | Meade MJ, Dreyer CW. What's in a hashtag: A content evaluation of Instagram posts related to orthodontic retention and retainers. Journal of the World Federation of Orthodontists. 2021;10(1):35-9. | Non-clinical study |
| 706 | Papageorgiou SN, Gölz L, Jäger A, Eliades T, Bourauel C, Afrashtehfar KI. Evidence regarding lingual fixed orthodontic appliances' therapeutic and adverse effects is insufficient. Evidence-Based Dentistry. 2016;17(2):54-5. | Non-clinical study |
| 707 | Putrino A, Caputo M, Giovannoni D, Barbato E, Galluccio G. Impact of the SARS-Cov2 Pandemic on Orthodontic Therapies: An Italian Experience of Teleorthodontics. Pesqui bras odontopediatria clín integr. 2020;20:e0100-e. | Non-clinical study |
| 708 | Quadrelli C, Veneziani A. The Stealth in comparison with other lingual brackets: properties and procedures for indirect bonding. Progress in orthodontics. 2007;8(1):156-72. | Non-clinical study |
| 709 | Rosa EP, Murakami-Malaquias-Silva F, Schalch TO, Teixeira DB, Horliana RF, Tortamano A, et al. Efficacy of photodynamic therapy and periodontal treatment in patients with gingivitis and fixed orthodontic appliances: Protocol of randomized, controlled, double-blind study. Medicine (Baltimore). 2020;99(14):e19429. | Non-clinical study |
| 710 | Svedström-Oristo AL, Ekholm H, Tolvanen M, Peltomäki T. Self-reported temporomandibular disorder symptoms and severity of malocclusion in prospective orthognathic-surgical patients. Acta Odontologica Scandinavica. 2016;74(6):466-70. | Non-clinical study |
| 711 | Tegelberg Å. Temporomandibular disorders in children and adolescents: A survey of dentists' attitudes, routine and experience. Swedish Dental Journal. 2001;25(3):119-27. | Non-clinical study |
| 712 | Vardimon AD, Robbins D, Brosh T. In-vivo von Mises strains during Invisalign treatment. American Journal of Orthodontics and Dentofacial Orthopedics. 2010;138(4):399-409. | Non-clinical study |
| 713 | Vilardi TMR. Influência dos anti-inflamatórios não esteroidais e seletivos COX-2 em osteoblastos durante a movimentação dentária induzida em ratos. 2015. p. 111-. | Non-clinical study |
| 714 | Yip BHK, Li X, Leung CHY, Gao T, Chung VCH, Yu FWP, et al. Trial Protocol: The use of mindfulness-based intervention for improving bracing compliance for adolescent idiopathic scoliosis patients: protocol for a randomised, controlled trial. J Physiother. 2018;64(3):193. | Non-clinical study |
| 715 | Alessandri-Bonetti G, Bortolotti F, Bartolucci ML, Marini I, D'Antò V, Michelotti A. The effects of mandibular advancement device on pressure pain threshold of masticatory muscles: A prospective controlled cohort study. Journal of Oral and Facial Pain and Headache. 2016;30(3):234-40. | Additional interventions (not pain-related) |
| 716 | Alikhani M, Raptis M, Zoldan B, Sangsuwon C, Lee YB, Alyami B, et al. Effect of micro-osteoperforations on the rate of tooth movement. Am J Orthod Dentofacial Orthop. 2013;144(5):639-48. | Additional interventions (not pain-related) |
| 717 | Antonarakis GS, Ameur S, Giannopoulou C, Kiliaridis S. Perception of pain in Class II malocclusion children treated with cervical headgear: a randomized controlled trial. Eur J Orthod. 2021;43(2):222-8. | Additional interventions (not pain-related) |
| 718 | Arora V, Sharma R, Chowdhary S. Comparative evaluation of treatment effects between two fixed functional appliances for correction of Class II malocclusion: A single-center, randomized controlled trial. Angle Orthod. 2018;88(3):259-66. | Additional interventions (not pain-related) |
| 719 | Babanouri N, Ajami S, Salehi P. Effect of mini-screw-facilitated micro-osteoperforation on the rate of orthodontic tooth movement: a single-center, split-mouth, randomized, controlled trial. Prog Orthod. 2020;21(1):7. | Additional interventions (not pain-related) |
| 720 | Baxmann M, McDonald F, Bourauel C, Jäger A. Expectations, acceptance, and preferences regarding microimplant treatment in orthodontic patients: A randomized controlled trial. Am J Orthod Dentofacial Orthop. 2010;138(3):250.e1-.e10; discussion -1. | Additional interventions (not pain-related) |
| 721 | Bock NC, von Bremen J, Kraft M, Ruf S. Plaque control effectiveness and handling of interdental brushes during multibracket treatment--a randomized clinical trial. Eur J Orthod. 2010;32(4):408-13. | Additional interventions (not pain-related) |
| 722 | Brandão LBC, Mucha JN. Grau de aceitação de mini-implantes por pacientes em tratamento ortodôntico: estudo preliminar. Rev dent press ortodon ortopedi facial. 2008;13(5):118-27. | Additional interventions (not pain-related) |
| 723 | Calik Koseler B, Yilanci H, Ramoglu SI. Does audiovisual information affect anxiety and perceived pain levels in miniscrew application? - a within-person randomized controlled trial. Prog Orthod. 2019;20(1):29. | Additional interventions (not pain-related) |
| 724 | Cassetta M, Giansanti M, Di Mambro A, Calasso S, Barbato E. Minimally invasive corticotomy in orthodontics using a three-dimensional printed CAD/CAM surgical guide. International Journal of Oral and Maxillofacial Surgery. 2016;45(9):1059-64. | Additional interventions (not pain-related) |
| 725 | Chandra R, Rachala M, Madhavi K, Kambalyal P, Reddy A, Ali M. Periodontally accelerated osteogenic orthodontics combined with recombinant human bone morphogenetic protein-2: An outcome assessment. Journal of Indian Society of Periodontology. 2019;23(3):257-63. | Additional interventions (not pain-related) |
| 726 | Charavet C, Lecloux G, Jackers N, Maes N, Lambert F. Patient-reported outcomes measures (PROMs) following a piezocision-assisted versus conventional orthodontic treatments: a randomized controlled trial in adults. Clin Oral Investig. 2019;23(12):4355-63. | Additional interventions (not pain-related) |
| 727 | Čirgić E, Kjellberg H, Hansen K. Discomfort, expectations, and experiences during treatment of large overjet with Andresen Activator or Prefabricated Functional Appliance: a questionnaire survey. Acta Odontol Scand. 2017;75(3):166-72. | Additional interventions (not pain-related) |
| 728 | Donohue VE, Marshman LAG, Winchester LJ. A clinical comparison of the quadhelix appliance and the nickel titanium (tandem loop) palatal expander: A preliminary, prospective investigation. European Journal of Orthodontics. 2004;26(4):411-20. | Additional interventions (not pain-related) |
| 729 | Elkordy SA, Fayed MM, Abouelezz AM, Attia KH. Comparison of patient acceptance of the Forsus Fatigue Resistant Device with and without mini-implant anchorage: a randomized controlled trial. Am J Orthod Dentofacial Orthop. 2015;148(5):755-64. | Additional interventions (not pain-related) |
| 730 | Feldmann I, Bazargani F. Pain and discomfort during the first week of rapid maxillary expansion (RME) using two different RME appliances: A randomized controlled trial. Angle Orthodontist. 2017;87(3):391-6. | Additional interventions (not pain-related) |
| 731 | Feldmann I, List T, Bondemark L. Orthodontic anchoring techniques and its influence on pain, discomfort, and jaw function--a randomized controlled trial. Eur J Orthod. 2012;34(1):102-8. | Additional interventions (not pain-related) |
| 732 | Feldmann I, List T, Feldmann H, Bondemark L. Pain intensity and discomfort following surgical placement of orthodontic anchoring units and premolar extraction: a randomized controlled trial. Angle Orthod. 2007;77(4):578-85. | Additional interventions (not pain-related) |
| 733 | Ganzer N, Feldmann I, Bondemark L. Pain and discomfort following insertion of miniscrews and premolar extractions: A randomized controlled trial. Angle Orthod. 2016;86(6):891-9. | Additional interventions (not pain-related) |
| 734 | Garfinkle JS, Cunningham LL, Jr., Beeman CS, Kluemper GT, Hicks EP, Kim MO. Evaluation of orthodontic mini-implant anchorage in premolar extraction therapy in adolescents. Am J Orthod Dentofacial Orthop. 2008;133(5):642-53. | Additional interventions (not pain-related) |
| 735 | Gibreal O, Hajeer MY, Brad B. Evaluation of the levels of pain and discomfort of piezocision-assisted flapless corticotomy when treating severely crowded lower anterior teeth: a single-center, randomized controlled clinical trial. BMC Oral Health. 2019;19(1):57. | Additional interventions (not pain-related) |
| 736 | Golshah A, Gorji K, Nikkerdar N. Effect of miniscrew insertion angle in the maxillary buccal plate on its clinical survival: a randomized clinical trial. Prog Orthod. 2021;22(1):22. | Additional interventions (not pain-related) |
| 737 | Gulduren K, Tumer H, Oz U. Effects of micro-osteoperforations on intraoral miniscrew anchored maxillary molar distalization : A randomized clinical trial. J Orofac Orthop. 2020;81(2):126-41. | Additional interventions (not pain-related) |
| 738 | Halicioğlu K, Kiki A, Yavuz I. Subjective symptoms of RME patients treated with three different screw activation protocols: a randomised clinical trial. Aust Orthod J. 2012;28(2):225-31. | Additional interventions (not pain-related) |
| 739 | Hatrom AA, Zawawi KH, Al-Ali RM, Sabban HM, Zahid TM, Al-Turki GA, et al. Effect of piezocision corticotomy on en-masse retraction. Angle Orthod. 2020;90(5):648-54. | Additional interventions (not pain-related) |
| 740 | Hawkins VM, Papadopoulou AK, Wong M, Pandis N, Dalci O, Darendeliler MA. The effect of piezocision vs no piezocision on maxillary extraction space closure: A split-mouth, randomized controlled clinical trial. Am J Orthod Dentofacial Orthop. 2022;161(1):7-19.e2. | Additional interventions (not pain-related) |
| 741 | Idris G, Hajeer MY, Al-Jundi A. Acceptance and discomfort in growing patients during treatment with two functional appliances: a randomised controlled trial. Eur J Paediatr Dent. 2012;13(3):219-24. | Additional interventions (not pain-related) |
| 742 | Jaber ST, Al-Sabbagh R, Hajeer MY. Evaluation of the efficacy of laser-assisted flapless corticotomy in accelerating canine retraction: a split-mouth randomized controlled clinical trial. Oral Maxillofac Surg. 2022;26(1):81-9. | Additional interventions (not pain-related) |
| 743 | Jung BA, Kunkel M, Göllner P, Liechti T, Wehrbein H. Success rate of second-generation palatal implants. Angle Orthodontist. 2009;79(1):85-90. | Additional interventions (not pain-related) |
| 744 | Kapetanović A, Noverraz RRM, Listl S, Bergé SJ, Xi T, Schols JGJH. What is the Oral Health-related Quality of Life following Miniscrew-Assisted Rapid Palatal Expansion (MARPE)? A prospective clinical cohort study. BMC Oral Health. 2022;22(1). | Additional interventions (not pain-related) |
| 745 | Kwong TS, Kusnoto B, Viana G, Evans CA, Watanabe K. The effectiveness of Oraqix versus TAC (a) for placement of orthodontic temporary anchorage devices. Angle Orthodontist. 2011;81(5):754-9. | Additional interventions (not pain-related) |
| 746 | Lamberton JA, Oesterle LJ, Shellhart WC, Newman SM, Harrell RE, Tilliss T, et al. Comparison of pain perception during miniscrew placement in orthodontic patients with a visual analog scale survey between compound topical and needle-injected anesthetics: A crossover, prospective, randomized clinical trial. Am J Orthod Dentofacial Orthop. 2016;149(1):15-23. | Additional interventions (not pain-related) |
| 747 | Lee TCK, McGrath CPJ, Wong RWK, Rabie ABM. Patients' perceptions regarding microimplant as anchorage in orthodontics. Angle Orthodontist. 2008;78(2):228-33. | Additional interventions (not pain-related) |
| 748 | Lehnen S, McDonald F, Bourauel C, Baxmann M. Patient expectations, acceptance and preferences in treatment with orthodontic mini-implants. A randomly controlled study. Part I: insertion techniques. J Orofac Orthop. 2011;72(2):93-102. | Additional interventions (not pain-related) |

| | | |
|---|---|---|
| 749 | Lehnen S, McDonald F, Bourauel C, Jäger A, Baxmann M. Expectations, acceptance and preferences of patients in treatment with orthodontic mini-implants: part II: implant removal. J Orofac Orthop. 2011;72(3):214-22. | Additional interventions (not pain-related) |
| 750 | Li J, Papadopoulou AK, Gandedkar N, Dalci K, Darendeliler MA, Dalci O. The effect of micro-osteoperforations on orthodontic space closure investigated over 12 weeks: a split-mouth, randomized controlled clinical trial. Eur J Orthod. 2022;44(4):427-35. | Additional interventions (not pain-related) |
| 751 | Liu H, Mou YD, Yu XG, Peng FY, Li QH, Deng FH. Stability and safety of mini-implant anchorage in orthodontic treatment. Chinese Journal of Tissue Engineering Research. 2016;20(8):1159-64. | Additional interventions (not pain-related) |
| 752 | Łyczek J, Kawala B, Antoszewska-Smith J. Influence of antibiotic prophylaxis on the stability of orthodontic microimplants: A pilot randomized controlled trial. Am J Orthod Dentofacial Orthop. 2018;153(5):621-31. | Additional interventions (not pain-related) |
| 753 | Ma Z, Xu G, Yang C, Xie Q, Shen Y, Zhang S. Efficacy of the technique of piezoelectric corticotomy for orthodontic traction of impacted mandibular third molars. Br J Oral Maxillofac Surg. 2015;53(4):326-31. | Additional interventions (not pain-related) |
| 754 | Majanni AMR, Hajeer MY, Khattab TZ, Burhan AS, Alkhouri I. Evaluation of pain, discomfort, and acceptance during the orthodontic treatment of class iii malocclusion using bone-anchored intermaxillary traction versus the removable mandibular retractor: A randomised controlled trial. Journal of Clinical and Diagnostic Research. 2020;14(3):ZC18-ZC23. | Additional interventions (not pain-related) |
| 755 | Massaro C, Janson G, Miranda F, Aliaga-Del Castillo A, Pugliese F, Lauris JRP, et al. Dental arch changes comparison between expander with differential opening and fan-type expander: a randomized controlled trial. Eur J Orthod. 2021;43(3):265-73. | Additional interventions (not pain-related) |
| 756 | Matos DS, Palma-Dibb RG, de Oliveira Santos C, da Conceição Pereira Saraiva M, Marques FV, Matsumoto MAN, et al. Evaluation of photobiomodulation therapy to accelerate bone formation in the mid palatal suture after rapid palatal expansion: a randomized clinical trial. Lasers Med Sci. 2021;36(5):1039-46. | Additional interventions (not pain-related) |
| 757 | McNally MR, Spary DJ, Rock WP. A randomized controlled trial comparing the quadhelix and the expansion arch for the correction of crossbite. J Orthod. 2005;32(1):29-35. | Additional interventions (not pain-related) |
| 758 | Miyawaki S, Koyama I, Inoue M, Mishima K, Sugahara T, Takano-Yamamoto T. Factors associated with the stability of titanium screws placed in the posterior region for orthodontic anchorage. American Journal of Orthodontics and Dentofacial Orthopedics. 2003;124(4):373-8. | Additional interventions (not pain-related) |
| 759 | Mohan A, Sivakumar A. A comparative evaluation of patient comfort with modified cheek retractor and normal cheek retractor on patients undergoing orthodontic bonding-a prospective randomised controlled split-mouth clinical trial. International Journal of Pharmaceutical Research. 2020;12(2):2338-42. | Additional interventions (not pain-related) |
| 760 | Mohd Ali ND, Al-Jaf NM, Norman NH. The orthodontic mini implants covers and their effect on the oral health related quality of life: A randomised controlled trial. International Medical Journal. 2021;28(3):352-6. | Additional interventions (not pain-related) |
| 761 | Mommaerts MY, Nols V, De Pauw G. Long-term prospective study of an orthodontic bone anchor. International Journal of Oral and Maxillofacial Implants. 2014;29(2):419-26. | Additional interventions (not pain-related) |
| 762 | Montenegro-Arana A, Arana-Gordillo LA, Farana D, Davila-Sanchez A, Jadad E, Coelho U, et al. Randomized Double-blind Clinical Trial of Bleaching Products in Patients Wearing Orthodontic Devices. Operative Dentistry. 2016;41(4):379-87. | Additional interventions (not pain-related) |
| 763 | Motoyoshi M, Yoshida T, Ono A, Shimizu N. Effect of cortical bone thickness and implant placement torque on stability of orthodontic mini-implants. International Journal of Oral and Maxillofacial Implants. 2007;22(5):779-84. | Additional interventions (not pain-related) |
| 764 | Murakami-Malaquias-Silva F, Rosa EP, Almeida PA, Schalch TO, Tenis CA, Negreiros RM, et al. Evaluation of the effects of photobiomodulation on orthodontic movement of molar verticalization with mini-implant A randomized double-blind protocol study. Medicine. 2020;99(13). | Additional interventions (not pain-related) |
| 765 | Ngan PW, Hagg U, Yiu C, Wei SH. Treatment response and long-term dentofacial adaptations to maxillary expansion and protraction. Seminars in orthodontics. 1997;3(4):255-64. | Additional interventions (not pain-related) |
| 766 | Nieri M, Paoloni V, Lione R, Barone V, Marino Merlo M, Giuntini V, et al. Comparison between two screws for maxillary expansion: a multicenter randomized controlled trial on patient's reported outcome measures. Eur J Orthod. 2021;43(3):293-300. | Additional interventions (not pain-related) |
| 767 | Pithon MM, Santos MJ, Ribeiro MC, Nascimento RC, Rodrigues RS, Ruellas AC, et al. Patients´perception of installation, use and results of orthodontic mini-implants. Acta odontol latinoam. 2015;28(2):108-12. | Additional interventions (not pain-related) |
| 768 | Prasad AS, Subramanian AK. Comparison of pain perception with different depths of micro-osteoperforation-a split mouth randomized control trial. International Journal of Pharmaceutical Research. 2020;12(2):2082-7. | Additional interventions (not pain-related) |
| 769 | Rana M, Gellrich NC, Rana M, Piffkó J, Kater W. Evaluation of surgically assisted rapid maxillary expansion with piezosurgery versus oscillating saw and chisel osteotomy - a randomized prospective trial. Trials. 2013;14:49. | Additional interventions (not pain-related) |
| 770 | Reznik DS, Jeske AH, Chen JW, English J. Comparative efficacy of 2 topical anesthetics for the placement of orthodontic temporary anchorage devices. Anesth Prog. 2009;56(3):81-5. | Additional interventions (not pain-related) |
| 771 | Rohida NS, Bhad W. A clinical, MRI, and EMG analysis comparing the efficacy of twin blocks and flat occlusal splints in the management of disc displacements with reduction. World J Orthod. 2010;11(3):236-44. | Additional interventions (not pain-related) |
| 772 | Sabzijati M, Rahbar M, Shanei F, Salehi-Vaziri A, Ghaffari HA, Abtahi SA. Comparing the clinical success rate of self-drilling and self-tapping mini-screws in the retraction of maxillary anterior teeth. Pesquisa Brasileira em Odontopediatria e Clinica Integrada. 2019;19(1). | Additional interventions (not pain-related) |
| 773 | Saleh M, Hajeer MY, Al-Jundi A. Assessment of pain and discomfort during early orthodontic treatment of skeletal Class III malocclusion using the Removable Mandibular Retractor Appliance. Eur J Paediatr Dent. 2013;14(2):119-24. | Additional interventions (not pain-related) |
| 774 | Santos SHB, Freitas KMSd, Valarelli FP, Cançado RH, Canuto LFG. Avaliação da utilização de anestésico tópico para instalação de mini-implantes ortodônticos. Ortodontia. 2012;45(3):248-56. | Additional interventions (not pain-related) |
| 775 | Sarul M, Lis J, Park HS, Rumin K. Evidence-based selection of orthodontic miniscrews, increasing their success rate in the mandibular buccal shelf. A randomized, prospective clinical trial. BMC Oral Health. 2022;22(1):414. | Additional interventions (not pain-related) |
| 776 | Shahrin A, Abdul Ghani SH, Norman NH. Pain experience with micro-osteoperforations during Initial orthodontic alignment: a randomized clinical trial. J oral res (Impresa). 2020;9(4):309-18. | Additional interventions (not pain-related) |
| 777 | Shaw WC, Addy M, Griffiths S, Price C. Chlorhexidine and traumatic ulcers in orthodontic patients. European Journal of Orthodontics. 1984;6(1):137-40. | Additional interventions (not pain-related) |
| 778 | Silva-Ruz I, Tort-Barahona F, Acuña-Aracena P, Villalon-Pooley P. Disyunción maxilar rápida asistida con microtornillos en pacientes en crecimiento con deficiencia maxilar transversal. Int j interdiscip dent (Print). 2021;14(1). | Additional interventions (not pain-related) |
| 779 | Silveira GS, Abreu LG, Palomo JM, da Matta Cid Pinto LS, de Sousa AA, Gribel BF, et al. Mini Hyrax vs Hyrax expanders in the rapid palatal expansion in adolescents with posterior crossbite: a randomized controlled clinical trial. Prog Orthod. 2021;22(1):30. | Additional interventions (not pain-related) |
| 780 | Sivarajan S, Doss JG, Papageorgiou SN, Cobourne MT, Wey MC. Mini-implant supported canine retraction with micro-osteoperforation: A split-mouth randomized clinical trial. Angle Orthod. 2019;89(2):183-9. | Additional interventions (not pain-related) |
| 781 | Sobouti F, Chiniforush N, Saravani HJ, Noroozian M, Cronshaw M, Navaei RA, et al. Efficacy of compound topical anesthesia combined with photobiomodulation therapy in pain control for placement of orthodontic miniscrew: a double-blind, randomized clinical trial. Lasers Med Sci. 2022;37(1):589-94. | Additional interventions (not pain-related) |
| 782 | Stivaros N, Lowe C, Dandy N, Doherty B, Mandall NA. A randomized clinical trial to compare the Goshgarian and Nance palatal arch. Eur J Orthod. 2010;32(2):171-6. | Additional interventions (not pain-related) |
| 783 | Sultana S, Ab Rahman N, Zainuddin SLA, Ahmad B. Effect of piezocision procedure in levelling and alignment stage of fixed orthodontic treatment: a randomized clinical trial. Sci Rep. 2022;12(1):6230. | Additional interventions (not pain-related) |
| 784 | Trakyali G, Sayinsu K, Mueezzinoglu AE, Arun T. Conscious hypnosis as a method for patient motivation in cervical headgear wear-a pilot study. European Journal of Orthodontics. 2008;30(2):147-52. | Additional interventions (not pain-related) |
| 785 | Ugolini A, Cossellu G, Farronato M, Silvestrini-Biavati A, Lanteri V. A multicenter, prospective, randomized trial of pain and discomfort during maxillary expansion: Leaf expander versus hyrax expander. Int J Paediatr Dent. 2020;30(4):421-8. | Additional interventions (not pain-related) |
| 786 | Valieri MM, de Freitas KMS, Valarelli FP, Cançado RH. Comparison of topical and infiltration anesthesia for orthodontic mini-implant placement. Dental Press Journal of Orthodontics. 2014;19(2):76-83. | Additional interventions (not pain-related) |
| 787 | Yavuz MC, Sunar O, Buyuk SK, Kantarcı A. Comparison of piezocision and discision methods in orthodontic treatment. Progress in Orthodontics. 2018;19(1). | Additional interventions (not pain-related) |
| 788 | Yi J, Xiao J, Li Y, Li X, Zhao Z. Efficacy of piezocision on accelerating orthodontic tooth movement: A systematic review. Angle Orthodontist. 2017;87(4):491-8. | Additional interventions (not pain-related) |
| 789 | Caccianiga G, Lo Giudice A, Longoni S, Ceraulo S, Baldoni M, Leonida A. Low-Level Laser Therapy Protocols in Dental Movement Acceleration and in Pain Management during Orthodontic Treatment. Journal of Biological Regulators and Homeostatic Agents. 2019;33(6):59-68. | Review |
| 790 | Cardoso PC, Espinosa DG, Mecenas P, Flores-Mir C, Normando D. Pain level between clear aligners and fixed appliances: a systematic review. Progress in Orthodontics. 2020;21(1). | Review |
| 791 | Domínguez Camacho A, Bravo Reyes M, Velasquez Cujar SA. A systematic review of the effective laser wavelength range in delivering photobiomodulation for pain relief in active orthodontic treatment. International Orthodontics. 2020;18(4):684-95. | Review |
| 792 | Jing D, Xiao J, Li X, Li Y, Zhao Z. The effectiveness of vibrational stimulus to accelerate orthodontic tooth movement: A systematic review. BMC Oral Health. 2017;17(1). | Review |
| 793 | Long H, Zhou Y, Pyakurel U, Liao L, Jian F, Xue J, et al. Comparison of adverse effects between lingual and labial orthodontic treatment A systematic review. Angle Orthodontist. 2013;83(6):1066-73. | Review |
| 794 | Papageorgiou SN, Gölz L, Jäger A, Eliades T, Bourauel C. Lingual vs. labial fixed orthodontic appliances: Systematic review and meta-analysis of treatment effects. European Journal of Oral Sciences. 2016;124(2):105-18. | Review |
| 795 | Sandhu SS, Cheema MS, Khehra HS. Comparative effectiveness of pharmacologic and nonpharmacologic interventions for orthodontic pain relief at peak pain intensity: A Bayesian network meta-analysis. American Journal of Orthodontics and Dentofacial Orthopedics. 2016;150(1):13-32. | Review |
| 796 | Xiaotinga L, Yinb T, Yangxic C. Interventions for pain during fixed orthodontic appliance therapy A systematic review. Angle Orthodontist. 2010;80(5):925-32. | Review |
| 797 | Yavagal C, Lal A, Soumya SV, Yavagal PC, Halapanavar B, Chavan Patil VV. Efficacy of laser photobiomodulation in reducing pain induced by orthodontic tooth movement: A systematic review. European Journal of Molecular and Clinical Medicine. 2021;8(3):4026-45. | Review |
| 798 | Abdelrahman RS, Al-Nimri KS, Al Maaitah EF. Pain experience during initial alignment with three types of nickel-titanium archwires: A prospective clinical trial. Angle Orthodontist. 2015;85(6):1021-6. | Non-randomized |
| 799 | Alarcón JA, Linde D, Barbieri G, Solano P, Caba O, Rios-Lugo MJ, et al. Calcitonin gingival crevicular fluid levels and pain discomfort during early orthodontic tooth movement in young patients. Archives of Oral Biology. 2013;58(6):590-5. | Non-randomized |
| 800 | Bretz YPM, Paiva SM, Bastos Lages EM, Abreu LG. Perceptions of pain levels and chewing impairment among adolescents undergoing orthodontic treatment with fixed appliances. J oral res (Impresa). 2018;7(3):102-7. | Non-randomized |
| 801 | Campos LA, Santos-Pinto A, Marôco J, Campos JADB. Pain perception in orthodontic patients: A model considering psychosocial and behavioural aspects. Orthodontics and Craniofacial Research. 2019;22(3):213-21. | Non-randomized |
| 802 | Campos MJdS, Vitral RWF. The influence of patient's motivation on reported pain during orthodontic treatment. Dental press j orthod (Impr). 2013;18(3):80-5. | Non-randomized |
| 803 | Caniklioglu C, Öztürk Y. Patient discomfort: A comparison between lingual and labial fixed appliances. Angle Orthodontist. 2005;75(1):86-91. | Non-randomized |
| 804 | Chen M, Wang DW, Wu LP. Fixed orthodontic appliance therapy and its impact on oral health-related quality of life in Chinese patients. Angle Orthodontist. 2010;80(1):49-53. | Non-randomized |
| 805 | Chen R, Zhi YF, Arvystas MG. Advanced Chinese NiTi alloy wire and clinical observations. Angle Orthodontist. 1992;62(1):59-66. | Non-randomized |
| 806 | Cooper-Kazaz R, Ivgi I, Canetti L, Bachar E, Tsur B, Chaushu S, et al. The impact of personality on adult patients' adjustability to orthodontic appliances. Angle Orthodontist. 2013;83(1):76-82. | Non-randomized |
| 807 | Cureton SL, Bice RW. Comparison of three types of separators in adult patients. J Clin Orthod. 1997;31(3):172-7. | Non-randomized |
| 808 | de Cassia Faglioni Boleta-Ceranto D, de Souza RS, Silverio-Lopes S, Moura NC. Orthodontic Post-Adjustment pain control with acupuncture. Dental Press Journal of Orthodontics. 2014;19(4):100-6. | Non-randomized |
| 809 | Dibbets JMH, van der Weele LT. Extraction, orthodontic treatment, and craniomandibular dysfunction. American Journal of Orthodontics and Dentofacial Orthopedics. 1991;99(3):210-9. | Non-randomized |
| 810 | Egermark I, Magnusson T, Carlsson GE. A 20-year follow-up of signs and symptoms of temporomandibular disorders and malocclusions in subjects with and without orthodontic treatment in childhood. Angle Orthodontist. 2003;73(2):109-15. | Non-randomized |
| 811 | Figueira IZ, Sousa APC, Machado AW, Habib FAL, Soares LGP, Pinheiro ALB. Clinical study on the efficacy of LED phototherapy for pain control in an orthodontic procedure. Lasers Med Sci. 2019;34(3):479-85. | Non-randomized |
| 812 | Fuck LM, Drescher D. Force systems in the initial phase of orthodontic treatment - A comparison of different leveling archwires. Journal of Orofacial Orthopedics. 2006;67(1):6-18. | Non-randomized |
| 813 | Fujiyama K, Deguchi T, Murakami T, Fujii A, Kushima K, Takano-Yamamoto T. Clinical effect of CO2 laser in reducing pain in orthodontics. Angle Orthodontist. 2008;78(2):299-303. | Non-randomized |
| 814 | Fujiyama K, Honjo T, Suzuki M, Matsuoka S, Deguchi T. Analysis of pain level in cases treated with Invisalign aligner: Comparison with fixed edgewise appliance therapy. Progress in Orthodontics. 2014;15(1). | Non-randomized |
| 815 | Galvão MCdS, Maltagliati LÁ, Sannomiya EK, Bommarito S. Avaliação comparativa entre desconforto causado pelo aparelho fixo lingual e labial. Ortodontia. 2008;41(1):19-24. | Non-randomized |
| 816 | Gameiro GH, Schultz C, Trein MP, Mundstock KS, Weidlich P, Goularte JF. Association among pain, masticatory performance, and proinflammatory cytokines in crevicular fluid during orthodontic treatment. American Journal of Orthodontics and Dentofacial Orthopedics. 2015;148(6):967-73. | Non-randomized |
| 817 | Henrikson T, Nilner M. Temporomandibular disorders, occlusion and orthodontic treatment. Journal of Orthodontics. 2003;30(2):129-37. | Non-randomized |
| 818 | Hiemstra R, Bos A, Hoogstraten J. Patients' and parents' expectations of orthodontic treatment. Journal of Orthodontics. 2009;36(4):219-28. | Non-randomized |

| | | |
|---|---|---|
| 819 | Ileri Z, Baka ZM, Akin M, Apiliogullari S, Basciftci FA. Effect of menstrual cycle on orthodontic pain perception: A controlled clinical trial. Journal of Orofacial Orthopedics. 2016;77(3):168-75. | Non-randomized |
| 820 | Johal A, Abed Al Jawad F, Marcenes W, Croft N. Does orthodontic treatment harm children's diets? Journal of Dentistry. 2013;41(11):949-54. | Non-randomized |
| 821 | Johal A, Ashari AB, Alamiri N, Fleming PS, Qureshi U, Cox S, et al. Pain experience in adults undergoing treatment: A longitudinal evaluation. Angle Orthodontist. 2018;88(3):292-8. | Non-randomized |
| 822 | Johal A, Fleming PS, Al Jawad FA. A prospective longitudinal controlled assessment of pain experience and oral health-related quality of life in adolescents undergoing fixed appliance treatment. Orthodontics and Craniofacial Research. 2014;17(3):178-86. | Non-randomized |
| 823 | Johnson PD, Cohen DA, Aiosa L, McGorray S, Wheeler T. Attitudes and compliance of pre-adolescent children during early treatment of Class II malocclusion. Clin Orthod Res. 1998;1(1):20-8. | Non-randomized |
| 824 | Larrea M, Salvador R, Cibrian R, Gandia JL, Paredes-Gallardo V. A new equation for predicting evolution of oral pain in orthodontic treatment: A longitudinal, prospective cohort study. Journal of Oral and Facial Pain and Headache. 2017;31(2):172-9. | Non-randomized |
| 825 | Leavitt AH, King GJ, Ramsay DS, Jackson DL. A longitudinal evaluation of pulpal pain during orthodontic tooth movement. Orthodontics & craniofacial research. 2002;5(1):29-37. | Non-randomized |
| 826 | Lee R, Hwang S, Lim H, Cha JY, Kim KH, Chung CJ. Treatment satisfaction and its influencing factors among adult orthodontic patients. American Journal of Orthodontics and Dentofacial Orthopedics. 2018;153(6):808-17. | Non-randomized |
| 827 | Lee W, Jang H, Park SH, Lee DW, Lee JH, Kim KH, et al. Incidence and clinical risk factors associated with the development of labial protuberances after orthodontic treatment. American Journal of Orthodontics and Dentofacial Orthopedics. 2022. | Non-randomized |
| 828 | Long H, Gao M, Zhu Y, Liu H, Zhou Y, Liao L, et al. The effects of menstrual phase on orthodontic pain following initial archwire engagement. Oral Diseases. 2017;23(3):331-6. | Non-randomized |
| 829 | Magalhães IB, Pereira LJ, Andrade AS, Gouvea DB, Gameiro GH. The influence of fixed orthodontic appliances on masticatory and swallowing threshold performances. Journal of Oral Rehabilitation. 2014;41(12):897-903. | Non-randomized |
| 830 | Marini I, Pelliccioni GA, Vecchiet F, Alessandri Bonetti G, Checchi L. A retentive system for intra-oral fluoride release during orthodontic treatment. Eur J Orthod. 1999;21(6):695-701. | Non-randomized |
| 831 | Marques LS, Paiva SM, Vieira-Andrade RG, Pereira LJ, Ramos-Jorge ML. Discomfort associated with fixed orthodontic appliances: determinant factors and influence on quality of life. Dental press j orthod (Impr). 2014;19(3):102-7. | Non-randomized |
| 832 | McAlinden RL, Ellis PE, Sandy JR. Report of an adverse incident in a randomized clinical trial. Journal of Orthodontics. 2005;32(3):203-5. | Non-randomized |
| 833 | Mendonça SL, Praxedes Neto OJ, de Oliveira PT, dos Santos PBD, Pinheiro FHSL. Comparison of friction produced by two types of orthodontic bracket protectors. Dental Press Journal of Orthodontics. 2014;19(1):86-91. | Non-randomized |
| 834 | Moraes JD-T. Avaliação da precisão de dinamômetros ortodônticos comercializados no Brasil. 2009. p. 57-. | Non-randomized |
| 835 | Nahin J, Arshad F, Srinivas BV, Kumar S, Lokesh NK. The Efficacy of Low-level Laser Therapy on Pain caused by Placement of the First Orthodontic Archwire: A Clinical Study. The journal of contemporary dental practice. 2018;19(4):450-5. | Non-randomized |
| 836 | Nedwed V, Miethke RR. Motivation, acceptance and problems of Invisalign® patients. Journal of Orofacial Orthopedics. 2005;66(2):162-73. | Non-randomized |
| 837 | Ngan P, Kess B, Wilson S. Perception of discomfort by patients undergoing orthodontic treatment. American Journal of Orthodontics and Dentofacial Orthopedics. 1989;96(1):47-53. | Non-randomized |
| 838 | Oliveira PGdSA, Tavares RR, Freitas JCd. Assessment of motivation, expectations and satisfaction of adult patients submitted to orthodontic treatment. Dental press j orthod (Impr). 2013;18(2):81-7. | Non-randomized |
| 839 | Pacheco-Pereira C, Brandelli J, Flores-Mir C. Patient satisfaction and quality of life changes after Invisalign treatment. American Journal of Orthodontics and Dentofacial Orthopedics. 2018;153(6):834-41. | Non-randomized |
| 840 | Saloom HF, Papageorgiou SN, Carpenter GH, Cobourne MT. The effect of obesity on orofacial pain during early orthodontic treatment with fixed appliances: A prospective cohort study. European Journal of Orthodontics. 2018;40(4):343-9. | Non-randomized |
| 841 | Sandhu SS, Leckie G. Orthodontic pain trajectories in adolescents: Between-subject and within-subject variability in pain perception. American Journal of Orthodontics and Dentofacial Orthopedics. 2016;149(4):491-500.e4. | Non-randomized |
| 842 | Sandhu SS, Sandhu J. Effect of physical activity level on orthodontic pain perception and analgesic consumption in adolescents. American Journal of Orthodontics and Dentofacial Orthopedics. 2015;148(4):618-27. | Non-randomized |
| 843 | Shalish M, Cooper-Kazaz R, Ivgi I, Canetti L, Tsur B, Bachar E, et al. Adult patients' adjustability to orthodontic appliances. Part I: A comparison between Labial, Lingual, and Invisalign™. European Journal of Orthodontics. 2012;34(6):724-30. | Non-randomized |
| 844 | Silva Andrade A, Marcon Szymanski M, Hashizume LN, Santos Mundstock K, Ferraz Goularte J, Hauber Gameiro G. Evaluation of stress biomarkers and electrolytes in saliva of patients undergoing fixed orthodontic treatment. Minerva Stomatologica. 2018;67(4):172-8. | Non-randomized |
| 845 | Silva I, Cardemil C, Kashani H, Bazargani F, Tarnow P, Rasmusson L, et al. Quality of life in patients undergoing orthognathic surgery – A two-centered Swedish study. Journal of Cranio-Maxillofacial Surgery. 2016;44(8):973-8. | Non-randomized |
| 846 | Sum FHKMH, Ren C, Gu M, Jin L, McGrath C, Yang Y. Oral Hygiene is Associated with Orthodontic Pain in Patients with Treated and Stabilised Periodontitis. Oral health & preventive dentistry. 2021;19(1):555-64. | Non-randomized |
| 847 | Tecco S, D'Attilio M, Tetè S, Festa F. Prevalence and type of pain during conventional and self-ligating orthodontic treatment. European Journal of Orthodontics. 2009;31(4):380-4. | Non-randomized |
| 848 | Tuncer Z, Ozsoy FS, Polat-Ozsoy O. Self-reported pain associated with the use of intermaxillary elastics compared to pain experienced after initial archwire placement. Angle Orthodontist. 2011;81(5):807-11. | Non-randomized |
| 849 | Turhani D, Scheriau M, Kapral D, Benesch T, Jonke E, Bantleon HP. Pain relief by single low-level laser irradiation in orthodontic patients undergoing fixed appliance therapy. Am J Orthod Dentofacial Orthop. 2006;130(3):371-7. | Non-randomized |
| 850 | Wang J, Tang X, Shen Y, Shang G, Fang L, Wang R, et al. The Correlations between Health-Related Quality of Life Changes and Pain and Anxiety in Orthodontic Patients in the Initial Stage of Treatment. Biomed Research International. 2015;2015. | Non-randomized |
| 851 | Wiechmann D, Gerß J, Stamm T, Hohoff A. Prediction of oral discomfort and dysfunction in lingual orthodontics: A preliminary report. American Journal of Orthodontics and Dentofacial Orthopedics. 2008;133(3):359-64. | Non-randomized |
| 852 | Wu A, McGrath C, Wong RWK, Wiechmann D, Rabie ABM. Comparison of oral impacts experienced by patients treated with labial or customized lingual fixed orthodontic appliances. American Journal of Orthodontics and Dentofacial Orthopedics. 2011;139(6):784-90. | Non-randomized |
| 853 | Wu AKY, McGrath C, Wong RWK, Wiechmann D, Rabie ABM. A comparison of pain experienced by patients treated with labial and lingual orthodontic appliances. European Journal of Orthodontics. 2010;32(4):403-7. | Non-randomized |
| 854 | Wu AKY, McGrath CPJ, Wong RWK, Rabie ABM, Wiechmann D. A comparison of pain experienced by patients treated with labial and lingual orthodontic appliances. Annals of the Royal Australasian College of Dental Surgeons. 2008;19:176-8. | Non-randomized |
| 855 | Bertl MH, Onodera K, Čelar AG. A prospective randomized split-mouth study on pain experience during chairside archwire manipulation in self-ligating and conventional brackets. Angle Orthod. 2013;83(2):292-7. | Split-mouth |
| 856 | Domínguez A, Velásquez SA. Effect of low-level laser therapy on pain following activation of orthodontic final archwires: a randomized controlled clinical trial. Photomed Laser Surg. 2013;31(1):36-40. | Split-mouth |
| 857 | Eslamian L, Borzabadi-Farahani A, Hassanzadeh-Azhiri A, Badiee MR, Fekrazad R. The effect of 810-nm low-level laser therapy on pain caused by orthodontic elastomeric separators. Lasers Med Sci. 2014;29(2):559-64. | Split-mouth |
| 858 | Farias RD, Closs LQ, Miguens SA, Jr. Evaluation of the use of low-level laser therapy in pain control in orthodontic patients: A randomized split-mouth clinical trial. Angle Orthod. 2016;86(2):193-8. | Split-mouth |
| 859 | Goldreich H, Gazit E, Lieberman MA, Rugh JD. The effect of pain from orthodontic arch wire adjustment on masseter muscle electromyographic activity. Am J Orthod Dentofacial Orthop. 1994;106(4):365-70. | Split-mouth |
| 860 | Jones M, Chan C. The pain and discomfort experienced during orthodontic treatment: a randomized controlled clinical trial of two initial aligning arch wires. Am J Orthod Dentofacial Orthop. 1992;102(4):373-81. | Split-mouth |
| 861 | Jung JG, Park JH, Kim SC, Kang KH, Cho JH, Cho JW, et al. Effectiveness of pulsed electromagnetic field for pain caused by placement of initial orthodontic wire in female orthodontic patients: A preliminary single-blind randomized clinical trial. Am J Orthod Dentofacial Orthop. 2017;152(5):582-91. | Split-mouth |
| 862 | Limsiriwong S, Khemaleelakul W, Sirabanchongkran S, Pothacharoen P, Kongtawelert P, Ongchai S, et al. Biochemical and clinical comparisons of segmental maxillary posterior tooth distal movement between two different force magnitudes. Eur J Orthod. 2018;40(5):496-503. | Split-mouth |
| 863 | Marini I, Bartolucci ML, Bortolotti F, Innocenti G, Gatto MR, Alessandri Bonetti G. The effect of diode superpulsed low-level laser therapy on experimental orthodontic pain caused by elastomeric separators: a randomized controlled clinical trial. Lasers Med Sci. 2015;30(1):35-41. | Split-mouth |
| 864 | Martins IP, Martins RP, Caldas S, Dos Santos-Pinto A, Buschang PH, Pretel H. Low-level laser therapy (830 nm) on orthodontic pain: blinded randomized clinical trial. Lasers Med Sci. 2019;34(2):281-6. | Split-mouth |
| 865 | Martins IP. Avaliação da efetividade do laser terapêutico no controle de índice de dor de pacientes em tratamento ortodôntico. 2011. p. 66-. | Split-mouth |
| 866 | Mayama A, Seiryu M, Takano-Yamamoto T. Effect of vibration on orthodontic tooth movement in a double blind prospective randomized controlled trial. Sci Rep. 2022;12(1):1288. | Split-mouth |
| 867 | Ogura M, Kamimura H, Al-Kalaly A, Nagayama K, Taira K, Nagata J, et al. Pain intensity during the first 7 days following the application of light and heavy continuous forces. European Journal of Orthodontics. 2009;31(3):314-9. | Split-mouth |
| 868 | Pérignon B, Bandiaky ON, Fromont-Colson C, Renaudin S, Peré M, Badran Z, et al. Effect of 970 nm low-level laser therapy on orthodontic tooth movement during Class II intermaxillary elastics treatment: a RCT. Sci Rep. 2021;11(1):23226. | Split-mouth |
| 869 | Pires LP, de Oliveira AH, da Silva HF, de Oliveira PT, dos Santos PB, Pinheiro FH. Can shielded brackets reduce mucosa alteration and increase comfort perception in orthodontic patients in the first 3 days of treatment? A single-blind randomized controlled trial. Am J Orthod Dentofacial Orthop. 2015;148(6):956-66. | Split-mouth |
| 870 | Qamruddin I, Alam MK, Fida M, Khan AG. Effect of a single dose of low-level laser therapy on spontaneous and chewing pain caused by elastomeric separators. Am J Orthod Dentofacial Orthop. 2016;149(1):62-6. | Split-mouth |
| 871 | Qamruddin I, Alam MK, Mahroof V, Fida M, Khamis MF, Husein A. Effects of low-level laser irradiation on the rate of orthodontic tooth movement and associated pain with self-ligating brackets. Am J Orthod Dentofacial Orthop. 2017;152(5):622-30. | Split-mouth |
| 872 | Sobouti F, Khatami M, Chiniforush N, Rakhshan V, Shariati M. Effect of single-dose low-level helium-neon laser irradiation on orthodontic pain: a split-mouth single-blind placebo-controlled randomized clinical trial. Prog Orthod. 2015;16:32. | Split-mouth |
| 873 | Stein S, Schauseil M, Hellak A, Korbmacher-Steiner H, Braun A. Influence of Photobiomodulation Therapy on Gingivitis Induced by Multi-Bracket Appliances: A Split-Mouth Randomized Controlled Trial. Photomedicine and Laser Surgery. 2018;36(8):399-405. | Split-mouth |
| 874 | Tripathi T, Singh N, Rai P, Khanna N. Separation and pain perception of Elastomeric, Kesling and Kansal separators. Dental Press J Orthod. 2019;24(2):42-8. | Split-mouth |
| 875 | Wu S, Chen Y, Zhang J, Chen W, Shao S, Shen H, et al. Effect of low-level laser therapy on tooth-related pain and somatosensory function evoked by orthodontic treatment. Int J Oral Sci. 2018;10(3):22. | Split-mouth |
| 876 | Abrahamsson C, Henrikson T, Nilner M, Sunzel B, Bondemark L, Ekberg E. TMD before and after correction of dentofacial deformities by orthodontic and orthognathic treatment. International Journal of Oral and Maxillofacial Surgery. 2013;42(6):752-8. | Surgical intervention |
| 877 | Al-Ainawi KI, Al-Mdalal Y, Hajeer MY. The Effect of Using a Modified Dentoalveolar Distractor on Canine Angulation following Rapid Canine Retraction: A Split-mouth Design Randomized Controlled Trial. J Contemp Dent Pract. 2016;17(1):49-57. | Surgical intervention |
| 878 | Ayoub AF, Duncan CM, McLean GR, Moos KF, Chibbaro PD. Response of patients and families to lengthening of the facial bones by extraoral distraction osteogenesis: A review of 14 patients. British Journal of Oral and Maxillofacial Surgery. 2002;40(5):397-405. | Surgical intervention |
| 879 | Baherimoghaddam T, Tabrizi R, Naseri N, Pouzesh A, Oshagh M, Torkan S. Assessment of the changes in quality of life of patients with class II and III deformities during and after orthodontic-surgical treatment. International Journal of Oral and Maxillofacial Surgery. 2016;45(4):476-85. | Surgical intervention |
| 880 | Çakir-Özkan N, Bereket C, Arici N, Elmali M, Şener I, Bekar E. The Radiological and Stereological Analysis of the Effect of Low-Level Laser Therapy on the Mandibular Midline Distraction Osteogenesis. J Craniofac Surg. 2015;26(7):e595-9. | Surgical intervention |
| 881 | Cheung LK, Chow LK, Chiu WK. A randomized controlled trial of resorbable versus titanium fixation for orthognathic surgery. Oral Surgery, Oral Medicine, Oral Pathology, Oral Radiology and Endodontology. 2004;98(4):386-97. | Surgical intervention |
| 882 | da Costa Senior O, Smeets M, Willaert R, Shaheen E, Jacobs R, Politis C. Complications Following One-Stage Versus Two-Stage Surgical Treatment of Transverse Maxillary Hypoplasia. Journal of Oral and Maxillofacial Surgery. 2021;79(7):1531-9. | Surgical intervention |
| 883 | de Leyva P, Eslava JM, Pezzi M, Sánchez-Jáuregui E, Haddad A, Baranda E, et al. Clear aligners versus fixed orthodontic appliances in surgery first orthognathic surgery. International Journal of Oral and Maxillofacial Surgery. 2019;48:107. | Surgical intervention |
| 884 | De Oliveira RF, Goldman RS, Mendes FM, De Freitas PM. Influence of Electroacupuncture and Laser-Acupuncture on Treating Paresthesia in Patients Submitted to Combined Orthognathic Surgery and Genioplasty. Medical Acupuncture. 2017;29(5):290-9. | Surgical intervention |
| 885 | Domínguez Camacho A, Velásquez SA, Benjumea Marulanda NJ, Moreno M. Photobiomodulation as oedema adjuvant in post-orthognathic surgery patients: A randomized clinical trial. Int Orthod. 2020;18(1):69-78. | Surgical intervention |
| 886 | Farella M, Michelotti A, Bocchino T, Cimino R, Laino A, Steenks MH. Effects of orthognathic surgery for class III malocclusion on signs and symptoms of temporomandibular disorders and on pressure pain thresholds of the jaw muscles. International Journal of Oral and Maxillofacial Surgery. 2007;36(7):583-7. | Surgical intervention |
| 887 | Felício CMd, Braga APG. Sinais e sintomas de desordem temporomandibular em pacientes orto-cirúrgicos. J bras ortodon ortop facial. 2005;10(56):187-94. | Surgical intervention |
| 888 | Gasperini G, Rodrigues de Siqueira IC, Rezende Costa L. Does low-level laser therapy decrease swelling and pain resulting from orthognathic surgery? Int J Oral Maxillofac Surg. 2014;43(7):868-73. | Surgical intervention |
| 889 | Hugo B, Becker S, Witt E. Assessment of the combined orthodontic-surgical treatment from the patients' point of view. A longitudinal study. Journal of orofacial orthopedics = Fortschritte der Kieferorthopädie : Organ/official journal Deutsche Gesellschaft für Kieferorthopädie. 1996;57(2):88-101. | Surgical intervention |
| 890 | Joss CU, Triaca A, Antonini M, Kuijpers-Jagtman AM, Kiliaridis S. Neurosensory and functional evaluation in distraction osteogenesis of the anterior mandibular alveolar process. International Journal of Oral and Maxillofacial Surgery. 2013;42(1):55-61. | Surgical intervention |

| | | |
|---|---|---|
| 891 | Khadka A, Liu Y, Li J, Zhu S, Luo E, Feng G, et al. Changes in quality of life after orthognathic surgery: A comparison based on the involvement of the occlusion. Oral Surgery, Oral Medicine, Oral Pathology, Oral Radiology and Endodontology. 2011;112(6):719-25. | Surgical intervention |
| 892 | Kiyak HA, McNeill RW, West RA, Hohl T, Heaton PJ. Personality characteristics as predictors and sequelae of surgical and conventional orthodontics. American Journal of Orthodontics. 1986;89(5):383-92. | Surgical intervention |
| 893 | Kulczynski FZ, Andriola FDO, Deon PH, Melo DADS, Pagnoncelli RM. Postural Assessment in Class III Patients Before Orthognathic Surgery. Journal of Oral and Maxillofacial Surgery. 2018;76(2):426-35. | Surgical intervention |
| 894 | Medeiros PJDA. Avaliação funcional de pacientes submetidos a recuo mandibular através da osteotomia vertical do ramo. 2000. p. 68-. | Surgical intervention |
| 895 | Nasreen S, Tagala M, Samal S, Gupta A, Sah R, Bhattacharjee D. Factors influencing clinical after effects of post orthognathic surgery - An observational clinical study. Journal of Pharmacy and Bioallied Sciences. 2021;13(5):S492-S5. | Surgical intervention |
| 896 | Neyt NMF, Mommaerts MY, Abeloos JVS, De Clercq CAS, Neyt LF. Problems, obstacles and complications with transpalatal distraction in non-congenital deformities. Journal of Cranio-Maxillofacial Surgery. 2002;30(3):139-43. | Surgical intervention |
| 897 | Øland J, Jensen J, Melsen B. Factors of importance for the functional outcome in orthognathic surgery patients: A prospective study of 118 patients. Journal of Oral and Maxillofacial Surgery. 2010;68(9):2221-31. | Surgical intervention |
| 898 | Pahkala RH, Kellokoski JK. Surgical-orthodontic treatment and patients' functional and psychosocial well-being. American Journal of Orthodontics and Dentofacial Orthopedics. 2007;132(2):158-64. | Surgical intervention |
| 899 | Phillips C, Bailey L, Kiyak HA, Bloomquist D. Effects of a computerized treatment simulation on patient expectations for orthognathic surgery. Int J Adult Orthodon Orthognath Surg. 2001;16(2):87-98. | Surgical intervention |
| 900 | Phillips C, Essick G, Preisser JS, Turvey TA, Tucker M, Lin D. Sensory retraining after orthognathic surgery: effect on patients' perception of altered sensation. J Oral Maxillofac Surg. 2007;65(6):1162-73. | Surgical intervention |
| 901 | Phillips C, Kiyak HA, Bloomquist D, Turvey TA. Perceptions of recovery and satisfaction in the short term after orthognathic surgery. J Oral Maxillofac Surg. 2004;62(5):535-44. | Surgical intervention |
| 902 | Primrose AC, Broadfoot E, Diner PA, Molina F, Moos KF, Ayoub AF. Patients' responses to distraction osteogenesis: A multi-centre study. International Journal of Oral and Maxillofacial Surgery. 2005;34(3):238-42. | Surgical intervention |
| 903 | Tamme JA, Rohnen M, Gaßling V, Ciesielski R, Fischer-Brandies H, Wiltfang J, et al. Correlation of general and oral health-related quality of life in malocclusion patients treated with a combined orthodontic and maxillofacial surgical approach. Journal of Cranio-Maxillofacial Surgery. 2017;45(12):1971-9. | Surgical intervention |
| 904 | Toll DE, Popović N, Drinkuth N. The use of MRI diagnostics in orthognathic surgery: prevalence of TMJ pathologies in Angle Class I, II, III patients. J Orofac Orthop. 2010;71(1):68-80. | Surgical intervention |
| 905 | Van de Velde FEG, Ortega-Castrillon A, Thierens LAM, Claes P, De Pauw GAM. The effect of manual lymphatic drainage on patient recovery after orthognathic surgery-A qualitative and 3-dimensional facial analysis. Oral Surg Oral Med Oral Pathol Oral Radiol. 2020;130(5):478-85. | Surgical intervention |
| 906 | Wolford LM, Movahed R, Dhameja A, Allen WR. Low condylectomy and orthognathic surgery to treat mandibular condylar osteochondroma: A retrospective review of 37 cases. Journal of Oral and Maxillofacial Surgery. 2014;72(9):1704-28. | Surgical intervention |
| 907 | Aboalnaga AA, Salah Fayed MM, El-Ashmawi NA, Soliman SA. Effect of micro-osteoperforation on the rate of canine retraction: a split-mouth randomized controlled trial. Prog Orthod. 2019;20(1):21. | Assessing canine retraction |
| 908 | Alam MK. Laser-Assisted Orthodontic Tooth Movement in Saudi Population: A Prospective Clinical Intervention of Low-Level Laser Therapy in the 1st Week of Pain Perception in Four Treatment Modalities. Pain Res Manag. 2019;2019:6271835. | Assessing canine retraction |
| 909 | Alfawal AMH, Hajeer MY, Ajaj MA, Hamadah O, Brad B, Latifeh Y. Evaluation of patient-centered outcomes associated with the acceleration of canine retraction by using minimally invasive surgical procedures: A randomized clinical controlled trial. Dent Med Probl. 2020;57(3):285-93. | Assessing canine retraction |
| 910 | Alkebsi A, Al-Maaitah E, Al-Shorman H, Abu Alhaija E. Three-dimensional assessment of the effect of micro-osteoperforations on the rate of tooth movement during canine retraction in adults with Class II malocclusion: A randomized controlled clinical trial. Am J Orthod Dentofacial Orthop. 2018;153(6):771-85. | Assessing canine retraction |
| 911 | Al-Naoum F, Hajeer MY, Al-Jundi A. Does alveolar corticotomy accelerate orthodontic tooth movement when retracting upper canines? A split-mouth design randomized controlled trial. J Oral Maxillofac Surg. 2014;72(10):1880-9. | Assessing canine retraction |
| 912 | Al-Shafi S, Pandis N, Darendeliler MA, Papadopoulou AK. Effect of light-emitting diode-mediated photobiomodulation on extraction space closure in adolescents and young adults: A split-mouth, randomized controlled trial. American Journal of Orthodontics and Dentofacial Orthopedics. 2021;160(1):19-28. | Assessing canine retraction |
| 913 | Angelieri F, Sousa MVdS, Kanashiro LK, Siqueira DF, Maltagliati LÁ. Efeitos do laser de baixa intensidade na sensibilidade dolorosa durante a movimentação ortodôntica. Dental press j orthod (Impr). 2011;16(4):95-102. | Assessing canine retraction |
| 914 | Attri S, Mittal R, Batra P, Sonar S, Sharma K, Raghavan S, et al. Comparison of rate of tooth movement and pain perception during accelerated tooth movement associated with conventional fixed appliances with micro-osteoperforations - a randomised controlled trial. J Orthod. 2018;45(4):225-33. | Assessing canine retraction |
| 915 | Badran SA, Al-Zaben JM, Al-Taie LM, Tbeishi H, Al-Omiri MK. Comparing patient-centered outcomes and efficiency of space closure between nickel-titanium closed-coil springs and elastomeric power chains during orthodontic treatment. Angle Orthod. 2022;92(4):471-7. | Assessing canine retraction |
| 916 | Barsoum HA, ElSayed HS, El Sharaby FA, Palomo JM, Mostafa YA. Comprehensive comparison of canine retraction using NiTi closed coil springs vs elastomeric chains. Angle Orthod. 2021;91(4):441-8. | Assessing canine retraction |
| 917 | Canigur Bavbek N, Bozkaya E, Isler SC, Elbeg S, Uraz A, Yuksel S. Assessment of salivary stress and pain biomarkers and their relation to self-reported pain intensity during orthodontic tooth movement: a longitudinal and prospective study. Journal of Orofacial Orthopedics. 2022;83(5):339-52. | Assessing canine retraction |
| 918 | Doshi-Mehta G, Bhad-Patil WA. Efficacy of low-intensity laser therapy in reducing treatment time and orthodontic pain: a clinical investigation. Am J Orthod Dentofacial Orthop. 2012;141(3):289-97. | Assessing canine retraction |
| 919 | Farhadian N, Miresmaeili A, Borjali M, Salehisaheb H, Farhadian M, Rezaei-Soufi L, et al. The effect of intra-oral LED device and low-level laser therapy on orthodontic tooth movement in young adults: A randomized controlled trial. Int Orthod. 2021;19(4):612-21. | Assessing canine retraction |
| 920 | Insee K, Pothacharoen P, Kongtawelert P, Ongchai S, Jotikasthira D, Krisanaprakornkit S. Comparisons of the chondroitin sulphate levels in orthodontically moved canines and the clinical outcomes between two different force magnitudes. European Journal of Orthodontics. 2014;36(1):39-46. | Assessing canine retraction |
| 921 | Işeri H, Kişnişci R, Bzizi N, Tüz H. Rapid canine retraction and orthodontic treatment with dentoalveolar distraction osteogenesis. American Journal of Orthodontics and Dentofacial Orthopedics. 2005;127(5):533-41. | Assessing canine retraction |
| 922 | Kundi I, Alam MK, Shaheed S. Micro-osteo perforation effects as an intervention on canine retraction. Saudi Dental Journal. 2020;32(1):15-20. | Assessing canine retraction |
| 923 | Luppanapornlarp S, Kajii TS, Surarit R, Iida J. Interleukin-1β levels, pain intensity, and tooth movement using two different magnitudes of continuous orthodontic force. European Journal of Orthodontics. 2010;32(5):596-601. | Assessing canine retraction |
| 924 | Prasad SMV, Prasanna TR, Kumaran V, Venkatachalam N, Ramees M, Abraham EA. Low-level laser therapy: A noninvasive method of relieving postactivation orthodontic pain-a randomized controlled clinical trial. Journal of Pharmacy and Bioallied Sciences. 2019;11(6):S228-S31. | Assessing canine retraction |
| 925 | Sousa MVS. Avaliação da influência do laser de baixa intensidade como recurso de ancoragem ortodôntica e na supressão da dor. 2013. p. 220-. | Assessing canine retraction |
| 926 | Souza JMSd. Avaliação da influência do laser de baixa intensidade na movimentação ortodôntica e supressão da dor. 2014. p. 146-. | Assessing canine retraction |
| 927 | Taha K, Conley RS, Arany P, Warunek S, Al-Jewair T. Effects of mechanical vibrations on maxillary canine retraction and perceived pain: a pilot, single-center, randomized-controlled clinical trial. Odontology. 2020;108(2):321-30. | Assessing canine retraction |
| 928 | Ureturk SE, Sarac M, Firatli S, Can SB, Guven Y, Firatli E. The effect of low-level laser therapy on tooth movement during canine distalization. Lasers in Medical Science. 2017;32(4):757-64. | Assessing canine retraction |
| 929 | Zahid H, Raja UB, Durrani OK, Shehzad S, Aslam M, Khan M. The Effectiveness of Trans-Electrical Nerve Stimulation (TENS) in Reducing Pain Caused by Orthodontic Movement; A Randomized Controlled Trial. Pakistan Journal of Medical and Health Sciences. 2022;16(4):542-4. | Assessing canine retraction |
| 930 | Araújo PVdS, Saraiva WM, Pereira NC, Vieira-Andrade RG, Santos CCdO, Fonseca-Silva T. Evaluation of Symptoms of Temporomandibular Disorders in Orthodontic Appliance Users. Int j odontostomatol (Print). 2018;12(1):99-104. | Assessing TMJ |
| 931 | Barone A, Sbordone L, Ramaglia L. Craniomandibular disorders and orthodontic treatment need in children. Journal of Oral Rehabilitation. 1997;24(1):2-7. | Assessing TMJ |
| 932 | Conti A, Freitas M, Conti P, Henriques J, Janson G. Relationship between signs and symptoms of temporomandibular disorders and orthodontic treatment: A cross-sectional study. Angle Orthodontist. 2003;73(4):411-7. | Assessing TMJ |
| 933 | Conti PC, Corrêa AS, Lauris JR, Stuginski-Barbosa J. Management of painful temporomandibular joint clicking with different intraoral devices and counseling: a controlled study. J Appl Oral Sci. 2015;23(5):529-35. | Assessing TMJ |
| 934 | Ding L, Chen R, Liu J, Wang Y, Chang Q, Ren L. The effect of functional mandibular advancement for adolescent patients with skeletal class II malocclusion on the TMJ: a systematic review and meta-analysis. Bmc Oral Health. 2022;22(1). | Assessing TMJ |
| 935 | Doepel M, Nilner M, Ekberg E, Y LEB. Long-term effectiveness of a prefabricated oral appliance for myofascial pain. J Oral Rehabil. 2012;39(4):252-60. | Assessing TMJ |
| 936 | Ekberg E, Nilner M. A 6- and 12-month follow-up of appliance therapy in TMD patients: a follow-up of a controlled trial. Int J Prosthodont. 2002;15(6):564-70. | Assessing TMJ |
| 937 | Ekberg E, Nilner M. Treatment outcome of appliance therapy in temporomandibular disorder patients with myofascial pain after 6 and 12 months. Acta Odontologica Scandinavica. 2004;62(6):343-9. | Assessing TMJ |
| 938 | Ekberg EC, Vallon D, Nilner M. The efficacy of appliance therapy in patients with temporomandibular disorders of mainly myogenous origin. A randomized, controlled, short-term trial. Journal of Orofacial Pain. 2003;17(2):133-9. | Assessing TMJ |
| 939 | Fricton J, Look JO, Wright E, Alencar FGP, Jr., Chen H, Lang M, et al. Systematic review and meta-analysis of randomized controlled trials evaluating intraoral orthopedic appliances for temporomandibular disorders. Journal of Oral and Facial Pain and Headache. 2010;24(3):237-54. | Assessing TMJ |
| 940 | Fricton J. Current Evidence Providing Clarity in Management of Temporomandibular Disorders: Summary of a Systematic Review of Randomized Clinical Trials for Intra-oral Appliances and Occlusal Therapies. Journal of Evidence-Based Dental Practice. 2006;6(1):48-52. | Assessing TMJ |
| 941 | Hans MG, Lieberman J, Goldberg J, Rozencweig G, Bellon E. A comparison of clinical examination, history, and magnetic resonance imaging for identifying orthodontic patients with temporomandibular joint disorders. American Journal of Orthodontics and Dentofacial Orthopedics. 1992;101(1):54-9. | Assessing TMJ |
| 942 | Hasanain F, Durham J, Moufti A, Steen IN, Wassell RW. Adapting the diagnostic definitions of the RDC/TMD to routine clinical practice: A feasibility study. Journal of Dentistry. 2009;37(12):955-62. | Assessing TMJ |
| 943 | Hasanoglu Erbasar GN, Alpaslan C, Eroglu Inan G. Can an NTI-tss device be effective as a first-line therapy in patients with TMD myofascial pain? J Oral Rehabil. 2017;44(8):589-93. | Assessing TMJ |
| 944 | Hirsch C. No increased risk of temporomandibular disorders and bruxism in children and adolescents during orthodontic therapy. Journal of Orofacial Orthopedics. 2009;70(1):39-50. | Assessing TMJ |
| 945 | Imai T, Okamoto T, Kaneko T, Umeda K, Yamamoto T, Nakamura S. Long-term follow-up of clinical symptoms in TMD patients who underwent occlusal reconstruction by orthodontic treatment. European Journal of Orthodontics. 2000;22(1):61-7. | Assessing TMJ |
| 946 | Karjalainen M, Le Bell Y, Jämsä T, Karjalainen S. Prevention of temporomandibular disorder-related signs and symptoms in orthodontically treated adolescents. A 3-year follow-up of a prospective randomized trial. Acta Odontol Scand. 1997;55(5):319-24. | Assessing TMJ |
| 947 | Keeling SD, Garvan CW, King GJ, Wheeler TT, McGorray S. Temporomandibular disorders after early Class II treatment with bionators and headgears: results from a randomized controlled trial. Semin Orthod. 1995;1(3):149-64. | Assessing TMJ |
| 948 | Kurt H, Alioğlu C, Karayazgan B, Tuncer N, Kılıçoğlu H. The effects of two methods of Class III malocclusion treatment on temporomandibular disorders. Eur J Orthod. 2011;33(6):636-41. | Assessing TMJ |
| 949 | Lagerström L, Egermark I, Carlsson GE. Signs and symptoms of temporomandibular disorders in 19-year-old individuals who have undergone orthodontic treatment. Swedish Dental Journal. 1998;22(5-6):177-86. | Assessing TMJ |
| 950 | Li LC, Wong RW, Rabie AB. Clinical effect of a topical herbal ointment on pain in temporomandibular disorders: a randomized placebo-controlled trial. J Altern Complement Med. 2009;15(12):1311-7. | Assessing TMJ |
| 951 | Machado E, Grehs RA, Cunali PA. Imaginologia da articulação temporomandibular durante o tratamento ortodôntico: uma revisão sistemática. Dental press j orthod (Impr). 2011;16(3):54-6. | Assessing TMJ |
| 952 | Machado E, Machado P, Cunali PA, Grehs RA. Ortodontia como fator de risco para disfunções temporomandibulares: uma revisão sistemática. Dental press j orthod (Impr). 2010;15(6):e1-e10. | Assessing TMJ |
| 953 | Machado NAdG. Avaliação da influência do tratamento ortodôntico em sinais e sintomas de disfunção temporomandibular, no relato de bruxismo, na hipervigilância à dor e nos sintomas de ansiedade e depressão. 2016. p. 92-. | Assessing TMJ |
| 954 | McBeth SB, Gratt BM. Thermographic assessment of temporomandibular disorders symptomology during orthodontic treatment. Am J Orthod Dentofacial Orthop. 1996;109(5):481-8. | Assessing TMJ |
| 955 | Nilner M, Ekberg E, Doepel M, Andersson J, Selovuo K, Le Bell Y. Short-term effectiveness of a prefabricated occlusal appliance in patients with myofascial pain. J Orofac Pain. 2008;22(3):209-18. | Assessing TMJ |
| 956 | Nilsson H, Limchaichana N, Nilner M, Ekberg EC. Short-term treatment of a resilient appliance in TMD pain patients: a randomized controlled trial. J Oral Rehabil. 2009;36(8):547-55. | Assessing TMJ |
| 957 | Nilsson H, Vallon D, Ekberg EC. Long-term efficacy of resilient appliance therapy in TMD pain patients: a randomised, controlled trial. J Oral Rehabil. 2011;38(10):713-21. | Assessing TMJ |
| 958 | O'Reilly MT, Rinchuse DJ, Close J. Class II elastics and extractions and temporomandibular disorders: A longitudinal prospective study. American Journal of Orthodontics and Dentofacial Orthopedics. 1993;103(5):459-63. | Assessing TMJ |
| 959 | Ortega ACBA, Pozza DH, Rodrigues LLFR, Guimarães AS. Relationship between orthodontics and temporomandibular disorders: A prospective study. Journal of Oral and Facial Pain and Headache. 2016;30(2):134-8. | Assessing TMJ |
| 960 | Pereira NC, Oltramari PVP, Conti PCR, Bonjardim LR, de Almeida-Pedrin RR, Fernandes TMF, et al. Frequency of awake bruxism behaviour in orthodontic patients: Randomised clinical trial: Awake bruxism behaviour in orthodontic patients. J Oral Rehabil. 2021;48(4):422-9. | Assessing TMJ |
| 961 | Portelli M, Matarese G, Militi A, Lo Giudice G, Nucera R, Lucchese A. Temporomandibular joint involvement in a cohort of patients with juvenile idiopatic arthritis and evaluation of the effect induced by functional orthodontic appliance: Clinical and radiographic investigation. European Journal of Paediatric Dentistry. 2014;15(1):63-6. | Assessing TMJ |
| 962 | Rodrigues-Garcia RC, Sakai S, Rugh JD, Hatch JP, Tiner BD, van Sickels JE, et al. Effects of major Class II occlusal corrections on temporomandibular signs and symptoms. J Orofac Pain. 1998;12(3):185-92. | Assessing TMJ |
| 963 | Tecco S, Teté S, Crincoli V, Festa MA, Festa F. Fixed orthodontic therapy in temporomandibular disorder (TMD) treatment: an alternative to intraoral splint. Cranio. 2010;28(1):30-42. | Assessing TMJ |
| 964 | Wadhwa L, Utreja A, Tewari A. A study of clinical signs and symptoms of temporomandibular dysfunction in subjects with normal occlusion, untreated, and treated malocclusions. American Journal of Orthodontics and Dentofacial Orthopedics. 1993;103(1):54-61. | Assessing TMJ |
| 965 | Björksved M, Arnrup K, Bazargani SM, Lund H, Magnusson A, Magnuson A, et al. Open vs closed surgical exposure of palatally displaced canines: a comparison of clinical and patient-reported outcomes-a multicentre, randomized controlled trial. Eur J Orthod. 2021;43(5):487-97. | Assessing impactions |

| | | |
|---|---|---|
| 966 | Björksved M, Arnrup K, Lindsten R, Magnusson A, Sundell AL, Gustafsson A, et al. Closed vs open surgical exposure of palatally displaced canines: surgery time, postoperative complications, and patients' perceptions: a multicentre, randomized, controlled trial. Eur J Orthod. 2018;40(6):626-35. | Assessing impactions |
| 967 | Chaushu G, Becker A, Zeltser R, Branski S, Chaushu S. Patients' perceptions of recovery after exposure of impacted teeth with a closed-eruption technique. American Journal of Orthodontics and Dentofacial Orthopedics. 2004;125(6):690-6. | Assessing impactions |
| 968 | Chaushu S, Becker A, Zeltser R, Vasker N, Chaushu G. Patients' perceptions of recovery after surgical exposure of impacted maxillary teeth treated with an open-eruption surgical-orthodontic technique. European Journal of Orthodontics. 2004;26(6):591-6. | Assessing impactions |
| 969 | Czochrowska EM, Stenvik A, Bjercke B, Zachrisson BU. Outcome of tooth transplantation: Survival and success rates 17-41 years posttreatment. American Journal of Orthodontics and Dentofacial Orthopedics. 2002;121(2):110-9. | Assessing impactions |
| 970 | Gharaibeh TM, Al-Nimri KS. Postoperative pain after surgical exposure of palatally impacted canines: closed-eruption versus open-eruption, a prospective randomized study. Oral Surg Oral Med Oral Pathol Oral Radiol Endod. 2008;106(3):339-42. | Assessing impactions |
| 971 | Kocsis A, Seres L. Orthodontic screws to extrude impacted maxillary canines. Journal of Orofacial Orthopedics-Fortschritte Der Kieferorthopadie. 2012;73(1):19-27. | Assessing impactions |
| 972 | Naoumova J. Interceptive Treatment Of Palatally Displaced Canines. Swed Dent J Suppl. 2014(234):7-118. | Assessing impactions |
| 973 | Parkin NA, Deery C, Smith AM, Tinsley D, Sandler J, Benson PE. No difference in surgical outcomes between open and closed exposure of palatally displaced maxillary canines. J Oral Maxillofac Surg. 2012;70(9):2026-34. | Assessing impactions |
| 974 | Plakwicz P, Wojtowicz A, Czochrowska EM. Survival and success rates of autotransplanted premolars: A prospective study of the protocol for developing teeth. American Journal of Orthodontics and Dentofacial Orthopedics. 2013;144(2):229-37. | Assessing impactions |
| 975 | Sampaziotis D, Tsolakis IA, Bitsanis E, Tsolakis AI. Open versus closed surgical exposure of palatally impacted maxillary canines: comparison of the different treatment outcomes-a systematic review. European Journal of Orthodontics. 2018;40(1):11-22. | Assessing impactions |
| 976 | Abdul-Aziz AI. Effect of chewing gum on pain following orthodontic elastomeric separators placement: A randomized controlled trial. Open Access Macedonian Journal of Medical Sciences. 2021;9:143-8. | No fixed appliances |
| 977 | Akin E, Gurton AU, Sagdic D. Effects of a segmented removable appliance in molar distalization. European Journal of Orthodontics. 2006;28(1):65-73. | No fixed appliances |
| 978 | Baldini A, Nota A, Santariello C, Assi V, Ballanti F, Cozza P. Influence of activation protocol on perceived pain during rapid maxillary expansion. Angle Orthodontist. 2015;85(6):1015-20. | No fixed appliances |
| 979 | Bezerra M, Habib FAL, Soares LGP, Vitale MC, Pinheiro ALB. Comparative analysis of Laser and LED phototherapies pain control after insertion of elastomeric separators in orthodontics patients: Clinical trial. J Photochem Photobiol B. 2022;233:112486. | No fixed appliances |
| 980 | Breitenbach S, Nicolau RA, Nóbrega CJP. Terapia LED de baixa intensidade no tratamento da dor durante movimento ortodôntico - Estudo Clínico. Conscientiae saúde (Impr). 2010;9(4). | No fixed appliances |
| 981 | Carr KM, Fields Jr HW, Beck FM, Kang EY, Kiyak HA, Pawlak CE, et al. Impact of verbal explanation and modified consent materials on orthodontic informed consent. American Journal of Orthodontics and Dentofacial Orthopedics. 2012;141(2):174-86. | No fixed appliances |
| 982 | Carter A, Al-Diwani H. What is the best method to ensure informed consent is valid for orthodontic treatment? A trial to assess long-term recall and comprehension. Evidence-Based Dentistry. 2022;23(2):52-3. | No fixed appliances |
| 983 | Castroflorio T, Bargellini A, Lucchese A, Manuelli M, Casasco F, Cugliari G, et al. Effects of clear aligners on sleep bruxism: randomized controlled trial. J Biol Regul Homeost Agents. 2018;32(2 Suppl. 2):21-9. | No fixed appliances |
| 984 | Cossellu G, Lanteri V, Lione R, Ugolini A, Gaffuri F, Cozza P, et al. Efficacy of ketoprofen lysine salt and paracetamol/acetaminophen to reduce pain during rapid maxillary expansion: A randomized controlled clinical trial. International Journal of Paediatric Dentistry. 2019;29(1):58-65. | No fixed appliances |
| 985 | de Araujo MC, Bocato JR, Berger SB, Pedron Oltramari PV, Ferreira Conti ACdC, de Almeida MR, et al. Perceived pain during rapid maxillary expansion in children with different expanders: A prospective study. Angle Orthodontist. 2021;91(4):484-9. | No fixed appliances |
| 986 | Esper MA, Nicolau RA, Arisawa EA. The effect of two phototherapy protocols on pain control in orthodontic procedure--a preliminary clinical study. Lasers Med Sci. 2011;26(5):657-63. | No fixed appliances |
| 987 | Furquim RD, Pascotto RC, Rino Neto J, Cardoso JR, Ramos AL. Low-level laser therapy effects on pain perception related to the use of orthodontic elastomeric separators. Dental Press J Orthod. 2015;20(3):37-42. | No fixed appliances |
| 988 | Hoffman WE. A study of four types of orthodontic separator. American Journal of Orthodontics. 1972;62(1):67-73. | No fixed appliances |
| 989 | Holmberg Peters F, Zaror Sánchez C, Fabres Suarez R, Sandoval Vidal P. Uso del láser terapéutico en el control del dolor en ortodoncia. Rev clín periodoncia implantol rehabil oral (Impr). 2011;4(3):114-6. | No fixed appliances |
| 990 | Katchooi M, Cohanim B, Tai S, Bayirli B, Spiekerman C, Huang G. Effect of supplemental vibration on orthodontic treatment with aligners: A randomized trial. Am J Orthod Dentofacial Orthop. 2018;153(3):336-46. | No fixed appliances |
| 991 | Kaur H, Bansal N, Abraham R. A randomized, single-blind, placebo-controlled trial to evaluate the effectiveness of verbal behavior modification and acetaminophen on orthodontic pain. Angle Orthod. 2019;89(4):617-23. | No fixed appliances |
| 992 | Kaya Y, Alkan Ö, Kömüroglu AU, Keskin S. Effects of ibuprofen and low-level laser therapy on orthodontic pain by means of the analysis of interleukin 1-beta and substance P levels in the gingival crevicular fluid. J Orofac Orthop. 2021;82(3):143-52. | No fixed appliances |
| 993 | Kim WT, Bayome M, Park JB, Park JH, Baek SH, Kook YA. Effect of frequent laser irradiation on orthodontic pain. A single-blind randomized clinical trial. Angle Orthod. 2013;83(4):611-6. | No fixed appliances |
| 994 | Lim HM, Lew KK, Tay DK. A clinical investigation of the efficacy of low level laser therapy in reducing orthodontic postadjustment pain. Am J Orthod Dentofacial Orthop. 1995;108(6):614-22. | No fixed appliances |
| 995 | Lorenzoni DC, Henriques JFC, Silva LKD, Alves ACM, Berretin-Felix G, Janson G. Users' perceptions and preferences towards maxillary removable orthodontic retainers: a crossover randomized clinical trial. Braz Oral Res. 2019;33:e078. | No fixed appliances |
| 996 | Nóbrega C, da Silva EM, de Macedo CR. Low-level laser therapy for treatment of pain associated with orthodontic elastomeric separator placement: a placebo-controlled randomized double-blind clinical trial. Photomed Laser Surg. 2013;31(1):10-6. | No fixed appliances |
| 997 | Pithon MM, Magno MB, Coqueiro RdS, de Paiva SM, Marques LS, Paranhus LR, et al. Oral health-related quality of life of children before, during, and after anterior open bite correction: A single-blinded randomized controlled trial. American Journal of Orthodontics and Dentofacial Orthopedics. 2019;156(3):303-11. | No fixed appliances |
| 998 | Roth PM, Thrash WJ. Effect of transcutaneous electrical nerve stimulation for controlling pain associated with orthodontic tooth movement. Am J Orthod Dentofacial Orthop. 1986;90(2):132-8. | No fixed appliances |
| 999 | Rumão WL, Valdrighi HC, Furletti VF, Gouvêa GR, Santamaria Junior M. Influence of photobiomodulation on pain perception during initial orthodontic tooth movement. Rev odontol UNESP (Online). 2020;49:e20200003-e. | No fixed appliances |
| 1000 | Stein S, Korbmacher-Steiner H, Popovic N, Braun A. Pain reduced by low-level laser therapy during use of orthodontic separators in early mixed dentition. J Orofac Orthop. 2015;76(5):431-9. | No fixed appliances |
| 1001 | Wiedel AP, Bondemark L. A randomized controlled trial of self-perceived pain, discomfort, and impairment of jaw function in children undergoing orthodontic treatment with fixed or removable appliances. Angle Orthod. 2016;86(2):324-30. | No fixed appliances |
| 1002 | Wiedel AP. FIXED OR REMOVABLE APPLIANCE FOR EARLY ORTHODONTIC TREATMENT OF FUNCTIONAL ANTERIOR CROSSBITE. Swed Dent J Suppl. 2015(238):10-72. | No fixed appliances |
| 1003 | Abu Alhaija ES, Abu Nabaa MA, Al Maaitah EF, Al-Omairi MK. Comparison of personality traits, attitude toward orthodontic treatment, and pain perception and experience before and after orthodontic treatment. Angle Orthodontist. 2015;85(3):474-9. | Not assessing levelling |
| 1004 | Ahangari Z, Nasser M, Mahdian M, Fedorowicz Z, Marchesan MA. Interventions for the management of external root resorption. Cochrane Database of Systematic Reviews. 2010;2010(6). | Not assessing levelling |
| 1005 | Al-Jundi A, Sakka S, Riba H, Ward T, Hanna R. Efficiency of er:Yag utilization in accelerating deep bite orthodontic treatment. Laser Therapy. 2018;27(3):193-202. | Not assessing levelling |
| 1006 | Al-Omiri MK, Alhaija ESA. Factors affecting patient satisfaction after orthodontic treatment. Angle Orthodontist. 2006;76(3):422-31. | Not assessing levelling |
| 1007 | Asher C, Shaw WC. Benzydamine hydrochloride in the treatment of ulceration associated with recently placed fixed orthodontic appliances. European Journal of Orthodontics. 1986;8(1):61-4. | Not assessing levelling |
| 1008 | Butera A, Gallo S, Pascadopoli M, Montasser MA, Abd El Latief MH, Modica GG, et al. Home Oral Care with Biomimetic Hydroxyapatite vs. Conventional Fluoridated Toothpaste for the Remineralization and Desensitizing of White Spot Lesions: Randomized Clinical Trial. Int J Environ Res Public Health. 2022;19(14). | Not assessing levelling |
| 1009 | Çokakoğlu S, Tan A. Effects of adhesive flash-free brackets on debonding pain and time: A randomized split-mouth clinical trial. Angle Orthod. 2020;90(6):758-65. | Not assessing levelling |
| 1010 | El Namrawy MM, El Sharaby F, Bushnak M. Intrusive Arch versus Miniscrew-Supported Intrusion for Deep Bite Correction. Open Access Macedonian Journal of Medical Sciences. 2019;7(11):1841-6. | Not assessing levelling |
| 1011 | Fekrazad R, Golshah A, Shirazi ER. Efficacy of Photobiomodulation Therapy Versus Soft Acrylic Wafer for Reduction of Pain Associated with Orthodontic Metal Bracket Removal: A Clinical Trial. Photobiomodul Photomed Laser Surg. 2022;40(7):463-71. | Not assessing levelling |
| 1012 | Forde K, Storey M, Littlewood SJ, Scott P, Luther F, Kang J. Bonded versus vacuum-formed retainers: a randomized controlled trial. Part 1: stability, retainer survival, and patient satisfaction outcomes after 12 months. Eur J Orthod. 2018;40(4):387-98. | Not assessing levelling |
| 1013 | Gupta SP, Rauniyar S, Prasad P, Pradhan PMS. A randomized controlled trial to evaluate the effectiveness of different methods on pain management during orthodontic debonding. Prog Orthod. 2022;23(1):7. | Not assessing levelling |
| 1014 | Horton JK, Buschang PH, Oliver DR, Behrents RG. Comparison of the effects of Hawley and perfector/spring aligner retainers on postorthodontic occlusion. Am J Orthod Dentofacial Orthop. 2009;135(6):729-36. | Not assessing levelling |
| 1015 | Jahanbin A, Ramazanzadeh B, Ahrari F, Forouzanfar A, Beidokhti M. Effectiveness of Er:YAG laser-aided fiberotomy and low-level laser therapy in alleviating relapse of rotated incisors. Am J Orthod Dentofacial Orthop. 2014;146(5):565-72. | Not assessing levelling |
| 1016 | Janson M, Hasund A. Functional problems in orthodontic patients out of retention. European Journal of Orthodontics. 1981;3(3):173-9. | Not assessing levelling |
| 1017 | Karobari MI, Assiry AA, Mirza MB, Sayed FR, Shaik S, Marya A, et al. Comparative Evaluation of Different Numerical Pain Scales Used for Pain Estimation during Debonding of Orthodontic Brackets. International Journal of Dentistry. 2021;2021. | Not assessing levelling |
| 1018 | Kilinç DD, Sayar G. Evaluation of pain perception during orthodontic debonding of metallic brackets with four different techniques. J appl oral sci. 2019;27:e20180003-e. | Not assessing levelling |
| 1019 | Krämer A, Sjöström M, Hallman M, Feldmann I. Vacuum-formed retainers and bonded retainers for dental stabilization-a randomized controlled trial. Part II: patients' perceptions 6 and 18 months after orthodontic treatment. Eur J Orthod. 2021;43(2):136-43. | Not assessing levelling |
| 1020 | Kraut J, Radin S, Trowbridge HI, Emling RC, Yankell SL. Clinical evaluations on thermal versus mechanical debonding of ceramic brackets. J Clin Dent. 1991;2(4):92-6. | Not assessing levelling |
| 1021 | Lorenzoni DC. Alterações produzidas na fala por contenções superiores ortodônticas - ensaio clínico randomizado prospectivo. 2016. p. 117-. | Not assessing levelling |
| 1022 | Mangnall LA, Dietrich T, Scholey JM. A randomized controlled trial to assess the pain associated with the debond of orthodontic fixed appliances. J Orthod. 2013;40(3):188-96. | Not assessing levelling |
| 1023 | Mehta L, Tewari S, Sharma R, Sharma RK, Tanwar N, Arora R. Assessment of the effect of orthodontic treatment on the stability of pre-orthodontic recession coverage by connective tissue graft: A randomized controlled clinical trial. Quintessence International. 2022;53(3):236-48. | Not assessing levelling |
| 1024 | Miresmæili AF, Mollabashi V, Gholami L, Farhadian M, Rezaei-Soufi L, Javanshir B, et al. Comparison of conventional and laser-aided fiberotomy in relapse tendency of rotated tooth: A randomized controlled clinical trial. Int Orthod. 2019;17(1):103-13. | Not assessing levelling |
| 1025 | Normando TS, Calcada FS, Ursi WJdS, Normando D. Patients' report of discomfort and pain during debonding of orthodontic brackets: a comparative study of two methods. World journal of orthodontics. 2010;11(4):e29-34. | Not assessing levelling |
| 1026 | Pithon MM, Santos Fonseca Figueiredo D, Oliveira DD, Coqueiro RS. What is the best method for debonding metallic brackets from the patient's perspective? Progress in Orthodontics. 2015;16(1). | Not assessing levelling |
| 1027 | Priya A, Jain RK, Santhosh Kumar MP. Efficacy of different methods to reduce pain during debonding of orthodontic brackets. Drug Invention Today. 2018;10(9):1700-3. | Not assessing levelling |
| 1028 | Rennick LA, Campbell PM, Naidu A, Taylor RW, Buschang PH. Effectiveness of a novel topical powder on the treatment of traumatic oral ulcers in orthodontic patients: A randomized controlled trial. Angle Orthod. 2016;86(3):351-7. | Not assessing levelling |
| 1029 | Russell JS, Littlewood SJ, Blance A, Mitchell L. The efficacy of a plasma arc light in orthodontic bonding: a randomized controlled clinical trial. J Orthod. 2008;35(3):202-9; discussion 175. | Not assessing levelling |
| 1030 | Sadek MM, Sabet NE, Hassan IT. Patient perceptions and oral impacts following labial and lingual biocreative therapy: A randomized clinical trial. Journal of the World Federation of Orthodontists. 2019;8(3):95-9. | Not assessing levelling |
| 1031 | Saleh M, Hajeer MY, Muessig D. Acceptability comparison between Hawley retainers and vacuum-formed retainers in orthodontic adult patients: a single-centre, randomized controlled trial. Eur J Orthod. 2017;39(4):453-61. | Not assessing levelling |
| 1032 | Scribante A, Gallo S, Celmare RL, D'Anto V, Grippaudo C, Gandini P, et al. Orthodontic debonding and tooth sensitivity of anterior and posterior teeth. The Angle orthodontist. 2020;90(6):766-73. | Not assessing levelling |
| 1033 | Störmann I, Ehmer U. A prospective randomized study of different retainer types. J Orofac Orthop. 2002;63(1):42-50. | Not assessing levelling |
| 1034 | Vatturu S, Ganugapanta VR, Teja NR, Singaraju GS, Mandava P, Priyanka JY. Comparative evaluation of the efficacy of the desensitizing and remineralizing agent in the reduction of dentin hypersensitivity after orthodontic debonding - a randomized clinical trial. Medicine and Pharmacy Reports. 2021;94(2):229-38. | Not assessing levelling |
| 1035 | Al-Attar A, Abid M. The Effect of Vitamin D3 on the Alignment of Mandibular Anterior Teeth: A Randomized Controlled Clinical Trial. International Journal of Dentistry. 2022;2022. | Assessing drugs |
| 1036 | Alcaraz de Poggi ME. Influencia de los fármacos en el tratamiento ortodóncico. 2011. p. 32-. | Assessing drugs |
| 1037 | Al-Melh MA, Andersson L. The effect of a lidocaine/prilocaine topical anesthetic on pain and discomfort associated with orthodontic elastomeric separator placement. Progress in Orthodontics. 2017;18(1). | Assessing drugs |
| 1038 | Alshammari AK, Huggare J. Pain relief after orthodontic archwire installation-a comparison between intervention with paracetamol and chewing gum: a randomized controlled trial. Eur J Orthod. 2019;41(5):478-85. | Assessing drugs |
| 1039 | Arantes GdM. Tenoxicam controla a dor sem apresentar efeito preemptivo ou interferir na movimentação ortodôntica de dentes caninos. 2009. p. [81]-[]. | Assessing drugs |
| 1040 | Arantes GM, Arantes VM, Ashmawi HA, Posso IP. Tenoxicam controls pain without altering orthodontic movement of maxillary canines. Orthod Craniofac Res. 2009;12(1):14-9. | Assessing drugs |
| 1041 | Bernhardt MK, Southard KA, Batterson KD, Logan HL, Baker KA, Jakobsen JR. The effect of preemptive and/or postoperative ibuprofen therapy for orthodontic pain. Am J Orthod Dentofacial Orthop. 2001;120(1):20-7. | Assessing drugs |
| 1042 | Bird SE, Williams K, Kula K. Preoperative acetaminophen vs ibuprofen for control of pain after orthodontic separator placement. Am J Orthod Dentofacial Orthop. 2007;132(4):504-10. | Assessing drugs |
| 1043 | Bradley RL, Ellis PE, Thomas P, Bellis H, Ireland AJ, Sandy JR. A randomized clinical trial comparing the efficacy of ibuprofen and paracetamol in the control of orthodontic pain. Am J Orthod Dentofacial Orthop. 2007;132(4):511-7. | Assessing drugs |

| | | |
|---|---|---|
| 1044 | Bruno MB, Bruno MAD, Krymchantowski AV, da Motta AFJ, Mucha JN. A double-blind, randomized clinical trial assessing the effects of a single dose of preemptive anti-inflammatory treatment in orthodontic pain. Progress in Orthodontics. 2011;12(1):2-7. | Assessing drugs |
| 1045 | CorrÊA AS, Almeida VLDE, Lopes BMV, Franco A, Matos FRD, Quintans-JÚNior LJ, et al. The influence of non-steroidal anti-inflammatory drugs and paracetamol used for pain control of orthodontic tooth movement: a systematic review. An acad bras ciênc. 2017;89(4):2851-63. | Assessing drugs |
| 1046 | El-Timamy A, El Sharaby F, Eid F, El Dakroury A, Mostafa Y, Shaker O. Effect of platelet-rich plasma on the rate of orthodontic tooth movement. Angle Orthod. 2020;90(3):354-61. | Assessing drugs |
| 1047 | Eslamian L, Borzabadi-Farahani A, Edini HZ, Badiee MR, Lynch E, Mortazavi A. The analgesic effect of benzocaine mucoadhesive patches on orthodontic pain caused by elastomeric separators, a preliminary study. Acta Odontologica Scandinavica. 2013;71(5):1168-73. | Assessing drugs |
| 1048 | Eslamian L, Borzabadi-Farahani A, Gholami H. The effect of benzocaine and ketoprofen gels on pain during fixed orthodontic appliance treatment: a randomised, double-blind, crossover trial. Aust Orthod J. 2016;32(1):64-72. | Assessing drugs |
| 1049 | Eslamian L, Gholami H, Mortazavi SA, Soheilifar S. Effect of 5% benzocaine gel on relieving pain caused by fixed orthodontic appliance activation. A double-blind randomized controlled trial. Orthod Craniofac Res. 2016;19(4):190-7. | Assessing drugs |
| 1050 | Eslamian L, Kianipour A, Mortazavi SAR. The analgesic efficacy of 5% naproxen gel for pain associated with orthodontic separator placement: A randomized double-blind controlled trial. Anesthesiology and Pain Medicine. 2017;7(2). | Assessing drugs |
| 1051 | Eslamian L, Torshabi M, Motamedian SR, Hemmati YB, Mortazavi SA. The effect of naproxen patches on relieving orthodontic pain by evaluation of VAS and IL-1β inflammatory factor: a split-mouth study. Dental Press J Orthod. 2019;24(6):27e1-e7. | Assessing drugs |
| 1052 | Gupta M, Kandula S, Laxmikanth SM, Vyavahare SS, Reddy SB, Ramachandra CS. Controlling pain during orthodontic fixed appliance therapy with non-steroidal anti-inflammatory drugs (NSAID): a randomized, double-blinded, placebo-controlled study. J Orofac Orthop. 2014;75(6):471-6. | Assessing drugs |
| 1053 | Holmberg Peters F, Fabres Suarez R, Sánchez CZ, Sandoval Vidal P. Uso de paracetamol en el control del dolor en ortodoncia. Int j odontostomatol (Print). 2012;6(1):39-44. | Assessing drugs |
| 1054 | Hosseinzadeh Nik T, Shahsavari N, Ghadirian H, Ostad SN. Acetaminophen Versus Liquefied Ibuprofen for Control of Pain During Separation in Orthodontic Patients: A Randomized Triple Blinded Clinical Trial. Acta Med Iran. 2016;54(7):418-21. | Assessing drugs |
| 1055 | Ireland AJ, Ellis P, Jordan A, Bradley R, Ewings P, Atack NE, et al. Comparative assessment of chewing gum and ibuprofen in the management of orthodontic pain with fixed appliances: A pragmatic multicenter randomized controlled trial. Am J Orthod Dentofacial Orthop. 2016;150(2):220-7. | Assessing drugs |
| 1056 | Kluemper GT, Hiser DG, Rayens MK, Jay MJ. Efficacy of a wax containing benzocaine in the relief of oral mucosal pain caused by orthodontic appliances. Am J Orthod Dentofacial Orthop. 2002;122(4):359-65. | Assessing drugs |
| 1057 | Kohli SS, Kohli VS. Effectiveness of piroxicam and ibuprofen premedication on orthodontic patients' pain experiences. Angle Orthod. 2011;81(6):1097-102. | Assessing drugs |
| 1058 | Lauritano D, Spadari F, Salvato A. [Efficacy of and tolerance to topical ketoprofen lysinate solution in acute inflammation of the mouth in orthodontic therapy. Randomized single blind study, with parallel groups, versus benzidamine hydrochloride]. Minerva Stomatol. 2000;49(11-12):561-8. | Assessing drugs |
| 1059 | Law SLS, Southard KA, Law AS, Logan HL, Jakobsen JR. An evaluation of preoperative ibuprofen for treatment of pain associated with orthodontic separator placement. American Journal of Orthodontics and Dentofacial Orthopedics. 2000;118(6):629-34. | Assessing drugs |
| 1060 | Leiva-Cala C, Ismael Lorenzo-Pouso A, Centenera-Centenera B, Lopez-Palafox J, Gandara-Vila P, Garcia-Garcia A, et al. Clinical efficacy of an Aloe Vera gel versus a 0.12% chlorhexidine gel in preventing traumatic ulcers in patients with fixed orthodontic appliances: a double-blind randomized clinical trial. Odontology. 2020;108(3):470-8. | Assessing drugs |
| 1061 | Makrygiannakis MA, Kaklamanos EG, Athanasiou AE. Medication and orthodontic tooth movement. Journal of Orthodontics. 2019. | Assessing drugs |
| 1062 | Minor V, Marris CK, McGorray SP, Yezierski R, Fillingim R, Logan H, et al. Effects of preoperative ibuprofen on pain after separator placement. Am J Orthod Dentofacial Orthop. 2009;136(4):510-7. | Assessing drugs |
| 1063 | Murdock S, Phillips C, Khondker Z, Hershey HG. Treatment of pain after initial archwire placement: a noninferiority randomized clinical trial comparing over-the-counter analgesics and bite-wafer use. Am J Orthod Dentofacial Orthop. 2010;137(3):316-23. | Assessing drugs |
| 1064 | Ngan P, Wilson S, Shanfeld J, Amini H. The effect of ibuprofen on the level of discomfort in patients undergoing orthodontic treatment. Am J Orthod Dentofacial Orthop. 1994;106(1):88-95. | Assessing drugs |
| 1065 | Owayda AM, Hajeer MY, Murad RMT, Al-Sabbagh R. The efficacy of low-level laser therapy versus paracetamol-caffeine in controlling orthodontic separation pain and changes in the oral-health-related quality of life in Class I malocclusions: A 3-arm, randomized, placebo-controlled clinical trial. J World Fed Orthod. 2022;11(3):75-82. | Assessing drugs |
| 1066 | Paganelli C. [Pharmacological support during orthodontic therapy with a topical anti-inflammatory]. Minerva Stomatol. 1993;42(6):271-4. | Assessing drugs |
| 1067 | Pelisson CJ. Avaliação do controle da dor após a fixação de brackets ortodônticos. 2008. p. 48-. | Assessing drugs |
| 1068 | Polat O, Karaman AI. Pain control during fixed orthodontic appliance therapy. Angle Orthod. 2005;75(2):214-9. | Assessing drugs |
| 1069 | Salmassian R, Oesterle LJ, Shellhart WC, Newman SM. Comparison of the efficacy of ibuprofen and acetaminophen in controlling pain after orthodontic tooth movement. Am J Orthod Dentofacial Orthop. 2009;135(4):516-21. | Assessing drugs |
| 1070 | Sandhu SS, Piepho HP, Khehra HS. Comparing the effectiveness profile of pharmacological interventions used for orthodontic pain relief: An arm-based multilevel network meta-Analysis of longitudinal data. European Journal of Orthodontics. 2017;39(6):601-14. | Assessing drugs |
| 1071 | Sari E, Olmez H, Gürton AU. Comparison of some effects of acetylsalicylic acid and rofecoxib during orthodontic tooth movement. Am J Orthod Dentofacial Orthop. 2004;125(3):310-5. | Assessing drugs |
| 1072 | Sharma N, Shah Y, Gholap P, Mhatre S, Srichand R, Sethumadhavan J. Comparative evaluation of Analgesic Effect of Piroxicam and Transcutaneous Electrical Nerve Stimulation Therapy on Pain associated with Orthodontic Separator: a Clinical Trail. NeuroQuantology. 2022;20(10):8845-50. | Assessing drugs |
| 1073 | Shetty N, Patil AK, Ganeshkar SV, Hegde S. Comparison of the effects of ibuprofen and acetaminophen on PGE2 levels in the GCF during orthodontic tooth movement: a human study. Prog Orthod. 2013;14(1):6. | Assessing drugs |
| 1074 | Silva N, Della Bona A, Cardoso M, Callegari-Jacques SM, Fornari F. Lactobacillus brevis CD2 attenuates traumatic oral lesions induced by fixed orthodontic appliance: A randomized phase 2 trial. Orthod Craniofac Res. 2021;24(3):379-85. | Assessing drugs |
| 1075 | Silva-Santos DJd. Avaliação da percepção da dor no tratamento ortodôntico: ensaio clínico randomizado. 2014. p. 54-. | Assessing drugs |
| 1076 | Steen Law SL, Southard KA, Law AS, Logan HL, Jakobsen JR. An evaluation of preoperative ibuprofen for treatment of pain associated with orthodontic separator placement. Am J Orthod Dentofacial Orthop. 2000;118(6):629-35. | Assessing drugs |
| 1077 | Sudhakar V, Vinodhini TS, Mathan Mohan A, Srinivasan B, Rajkumar BK. The efficacy of different pre-and post-operative analgesics in the management of pain after orthodontic separator placement: A randomized clinical trial. Journal of Pharmacy and Bioallied Sciences. 2014;6(SUPPL. 1):S80-S4. | Assessing drugs |
| 1078 | Villa PA, Oberti G, Moncada CA, Vasseur O, Jaramillo A, Tobón D, et al. Pulp-dentine complex changes and root resorption during intrusive orthodontic tooth movement in patients prescribed nabumetone. J Endod. 2005;31(1):61-6. | Assessing drugs |
| 1079 | Yassaei S, Vahidi A, Farahat F. Comparison of the efficacy of calcium versus acetaminophen on reduction of orthodontic pain. Indian J Dent Res. 2012;23(5):608-12. | Assessing drugs |
| 1080 | Young AN, Taylor RW, Taylor SE, Linnebur SA, Buschang PH. Evaluation of preemptive valdecoxib therapy on initial archwire placement discomfort in adults. Angle Orthod. 2006;76(2):251-9. | Assessing drugs |
| 1081 | Young AN. An evaluation of preemptive valdecoxib therapy on discomfort caused by initial archwire placement in adult orthodontic patients. Tex Dent J. 2005;122(7):673. | Assessing drugs |
| 1082 | Zarif Najafi H, Oshagh M, Salehi P, Babanouri N, Torkan S. Comparison of the effects of preemptive acetaminophen, ibuprofen, and meloxicam on pain after separator placement: a randomized clinical trial. Prog Orthod. 2015;16:34. | Assessing drugs |
| 1083 | Almallah MME, Hajeer MY, Almahdi WH, Burhan AS, Latifeh Y, Madkhaneh SK. Assessment of a single versus double application of low-level laser therapy in pain reduction following orthodontic elastomeric separation: A randomized controlled trial. Dent Med Probl. 2020;57(1):45-52. | Same category |
| 1084 | AlSayed Hasan MMA, Sultan K, Hamadah O. Evaluating low-level laser therapy effect on reducing orthodontic pain using two laser energy values: a split-mouth randomized placebo-controlled trial. Eur J Orthod. 2018;40(1):23-8. | Same category |
| 1085 | Hohoff A, Stamm T, Ehmer U. Comparison of the effect on oral discomfort of two positioning techniques with lingual brackets. Angle Orthodontist. 2004;74(2):226-33. | Same category |
| 1086 | Abreu LG, Melgaço CA, Abreu MHNG, Lages EMB, Paiva SM. Agreement between adolescents and parents or caregivers in rating adolescents' quality of life during orthodontic treatment. American Journal of Orthodontics and Dentofacial Orthopedics. 2015;148(6):1036-42. | Pain not assessed |
| 1087 | Alhaija ESA, Shahin AY, Badran SA, Daher SO, Daher HO. Pulpal blood flow changes and pain scores related to using Superelastic 0. 018-inch Nickel Titanium as the first orthodontic alignment archwire: a prospective clinical trial. J appl oral sci. 2021;29:e20210089-e. | Pain not assessed |
| 1088 | Anggani HS, Rusli V, Bachtiar EW. Chitosan gel prevents the growth of Porphyromonas gingivalis, Tannerella forsythia, and Treponema denticola in mini-implant during orthodontic treatment. Saudi Dental Journal. 2021;33(8):1024-8. | Pain not assessed |
| 1089 | Caccianiga G, Paiusco A, Perillo L, Nucera R, Pinsino A, Maddalone M, et al. Does Low-Level Laser Therapy Enhance the Efficiency of Orthodontic Dental Alignment? Results from a Randomized Pilot Study. Photomedicine and Laser Surgery. 2017;35(8):421-6. | Pain not assessed |
| 1090 | Cakmak F, Turk T, Karadeniz EI, Elekdag-Turk S, Darendeliler MA. Physical properties of root cementum: part 24. Root resorption of the first premolars after 4 weeks of occlusal trauma. Am J Orthod Dentofacial Orthop. 2014;145(5):617-25. | Pain not assessed |
| 1091 | Chavarría-Bolaños D, Martinez-Zumaran A, Lombana N, Flores-Reyes H, Pozos-Guillen A. Expression of substance P, calcitonin gene-related peptide, β-endorphin and methionine-enkephalin in human dental pulp tissue after orthodontic intrusion: a pilot study. Angle Orthod. 2014;84(3):521-6. | Pain not assessed |
| 1092 | Cozzani M, Ragazzini G, Delucchi A, Mutinelli S, Barreca C, Rinchuse DJ, et al. Oral hygiene compliance in orthodontic patients: a randomized controlled study on the effects of a post-treatment communication. Progress in Orthodontics. 2016;17. | Pain not assessed |
| 1093 | Deana NF, Alves N, Sandoval P. Impact of the First Archwires Placement on the Everyday Activities of Orthodontics Patients. Int j odontostomatol (Print). 2019;13(4):385-91. | Pain not assessed |
| 1094 | Desman AR, Fields HW, Ni A, Robinson FG, Skulski B, Firestone AR, et al. Rehearsal's effect on long-term recall and comprehension of orthodontic informed consent. Am J Orthod Dentofacial Orthop. 2022;161(2):e114-e26. | Pain not assessed |
| 1095 | DiBiase AT, Woodhouse NR, Papageorgiou SN, Johnson N, Slipper C, Grant J, et al. Effect of supplemental vibrational force on orthodontically induced inflammatory root resorption: A multicenter randomized clinical trial. Am J Orthod Dentofacial Orthop. 2016;150(6):918-27. | Pain not assessed |
| 1096 | El Shehawy TO, Hussein FA, Awady AAEI. Outcome of photodynamic therapy on orthodontic leveling and alignment of mandibular anterior segment: A controlled clinical trial. Photodiagnosis and Photodynamic Therapy. 2020;31. | Pain not assessed |
| 1097 | El-Angbawi AM, Yassir YA, McIntyre GT, Revie GF, Bearn DR. A randomized clinical trial of the effectiveness of 0.018-inch and 0.022-inch slot orthodontic bracket systems: part 3-biological side-effects of treatment. Eur J Orthod. 2019;41(2):154-64. | Pain not assessed |
| 1098 | Hasan MMAA, Sultan K, Hamadah O. Low-level laser therapy effectiveness in accelerating orthodontic tooth movement: A randomized controlled clinical trial. Angle Orthodontist. 2017;87(4):499-504. | Pain not assessed |
| 1099 | Khattab TZ, Farah H, Al-Sabbagh R, Hajeer MY, Haj-Hamed Y. Speech performance and oral impairments with lingual and labial orthodontic appliances in the first stage of fixed treatment A randomized controlled trial. Angle Orthodontist. 2013;83(3):519-26. | Pain not assessed |
| 1100 | Lalnunpuii H, Batra P, Sharma K, Srivastava A, Raghavan S. Comparison of rate of orthodontic tooth movement in adolescent patients undergoing treatment by first bicuspid extraction and en-mass retraction, associated with low level laser therapy in passive self-ligating and conventional brackets: A randomized controlled trial. International Orthodontics. 2020;18(3):412-23. | Pain not assessed |
| 1101 | Lazaridis K, Athanasiou AE, Knösel M, Papadopoulos MA. Color changes of maxillary and mandibular incisors following surgical orthodontic treatment: A prospective controlled clinical pilot study. Journal of Orofacial Orthopedics. 2022;83(2):99-107. | Pain not assessed |
| 1102 | Lin F, He Y, Ni Z, Olive R, Ren M, Yao L, et al. Individualized intervention to reduce anxiety in adult orthodontic patients based on Q methodology. Am J Orthod Dentofacial Orthop. 2017;152(2):161-70. | Pain not assessed |
| 1103 | Lo Giudice A, Nucera R, Leonardi R, Paiusco A, Baldoni M, Caccianiga G. A Comparative Assessment of the Efficiency of Orthodontic Treatment With and Without Photobiomodulation During Mandibular Decrowding in Young Subjects: A Single-Center, Single-Blind Randomized Controlled Trial. Photobiomodulation Photomedicine and Laser Surgery. 2020;38(5):272-9. | Pain not assessed |
| 1104 | Miles PG, Weyant RJ, Rustveld L. A clinical trial of Damon 2 (TM) vs conventional twin brackets during initial alignment. Angle Orthodontist. 2006;76(3):480-5. | Pain not assessed |
| 1105 | Mistry D, Dalci O, Papageorgiou SN, Darendeliler MA, Papadopoulou AK. The effects of a clinically feasible application of low-level laser therapy on the rate of orthodontic tooth movement: A triple-blind, split-mouth, randomized controlled trial. American Journal of Orthodontics and Dentofacial Orthopedics. 2020;157(4):444-53. | Pain not assessed |
| 1106 | Mohammad Ali Baladi S, Valiente Zaldívar C. Laserterapia como analgésico para el movimiento dentario ortodóncico. Ortod esp (Ed impr). 2012;52(2):68-78. | Pain not assessed |
| 1107 | Murakami T, Kawanabe N, Kataoka T, Hoshijima M, Komori H, Fujisawa A, et al. A Single-center, Open-label, Randomized Controlled Clinical Trial to Evaluate the Efficacy and Safety of the Indirect Bonding Technique. Acta Med Okayama. 2016;70(5):413-6. | Pain not assessed |
| 1108 | Nayyer N, Tripathi T, Rai P, Kanase A. Effect of photobiomodulation on external root resorption during orthodontic tooth movement - a randomized controlled trial. International Orthodontics. 2021;19(2):197-206. | Pain not assessed |
| 1109 | Nazir M, Walsh T, Mandall NA, Matthew S, Fox D. Banding versus bonding of first permanent molars: a multi-centre randomized controlled trial. J Orthod. 2011;38(2):81-9. | Pain not assessed |
| 1110 | Olsson M, Lindqvist B. Mandibular function before and after orthodontic treatment. European Journal of Orthodontics. 1995;17(3):205-14. | Pain not assessed |
| 1111 | Olsson M, Lindqvist B. Mandibular function before orthodontic treatment. European Journal of Orthodontics. 1992;14(1):61-8. | Pain not assessed |
| 1112 | Ousehal L, Lakhdar A, Elquars F. [Comparison of the effect of paracetamol and ibuprofen on orthodontic pain]. International orthodontics. 2009;7(2):193-206. | Pain not assessed |
| 1113 | Patel S, McGorray SP, Yezierski R, Fillingim R, Logan H, Wheeler TT. Effects of analgesics on orthodontic pain. Am J Orthod Dentofacial Orthop. 2011;139(1):e53-8. | Pain not assessed |
| 1114 | Patel S. Effects of analgesics on pre- and post-separator pain. Todays FDA. 2010;22(5):46-9, 51, 3. | Pain not assessed |
| 1115 | Pérez V, Pupo Marrugo S, Moneriz Pretell C, Bedoya Pérez M, De Armas Orozco M. Concentración de la enzima AST (aspartato aminotransferasa) en dientes sometidos a fuerzas ortodónticas intrusivas. Av odontoestomatol. 2017;33(1):19-24. | Pain not assessed |
| 1116 | Polat O, Karaman AI, Durmus E. Effects of preoperative ibuprofen and naproxen sodium on orthodontic pain. Angle Orthod. 2005;75(5):791-6. | Pain not assessed |
| 1117 | Salehi P, Momeni Danaie S. Comparison of the antibacterial effects of persica mouthwash with chlorhexidine on streptococcus mutans in orthodontic patients. Daru. 2006;14(4):178-82. | Pain not assessed |
| 1118 | Scott P, DiBiase AT, Sherriff M, Cobourne MT. Alignment efficiency of Damon3 self-ligating and conventional orthodontic bracket systems: A randomized clinical trial. American Journal of Orthodontics and Dentofacial Orthopedics. 2008;134(4). | Pain not assessed |

| | | |
|---|---|---|
| 1119 | Trein MP, Mundstock KS, Maciel L, Rachor J, Gameiro GH. Pain, masticatory performance and swallowing threshold in orthodontic patients. Dental Press Journal of Orthodontics. 2013;18(6):117-23. | Pain not assessed |
| 1120 | Tunçer Z, Polat-Ozsoy O, Demirbilek M, Bostanoglu E. Effects of various analgesics on the level of prostaglandin E2 during orthodontic tooth movement. Eur J Orthod. 2014;36(3):268-74. | Pain not assessed |
| 1121 | Al-Melh MA, Nada A, Badr H, Andersson L. Effect of an Anesthetic Chewing Gum on the Initial Pain or Discomfort from Orthodontic Elastomeric Separator Placement. J Contemp Dent Pract. 2019;20(11):1286-92. | Data unavailable |
| 1122 | Alqareer A, Alyahya A, Al-Anezi SA, AlAwadhi A, Al Qabandi S, Alyaseen M. Efficacy of Chewing Gum to Reduce Orthodontic Pain Compared to Placebo: A Blinded, Parallel-Group, Preliminary Clinical Trial. J Oral Facial Pain Headache. 2019;33(3):301–7. | Data unavailable |
| 1123 | Atik E, Ciğer S. An assessment of conventional and self-ligating brackets in Class I maxillary constriction patients. Angle Orthod. 2014;84(4):615-22. | Data unavailable |
| 1124 | Azeem M, Ejaz Z, Ashraf A, Bukhari F, Ali MM, Rashid A. Music for pain control following orthodontic initial archwire placement. Pakistan Journal of Medical and Health Sciences. 2021;15(6):1347-8. | Data unavailable |
| 1125 | Bayani S, Rostami S, Ahrari F, Saeedipouya I. A randomized clinical trial comparing the efficacy of bite wafer and low level laser therapy in reducing pain following initial arch wire placement. Laser Therapy. 2016;25(2):121-9. | Data unavailable |
| 1126 | Benson PE, Razi RM, Al-Bloushi RJ. The effect of chewing gum on the impact, pain and breakages associated with fixed orthodontic appliances: a randomized clinical trial. Orthod Craniofac Res. 2012;15(3):178-87. | Data unavailable |
| 1127 | Celebi F, Turk T, Bicakci AA. Effects of low-level laser therapy and mechanical vibration on orthodontic pain caused by initial archwire. Am J Orthod Dentofacial Orthop. 2019;156(1):87-93. | Data unavailable |
| 1128 | Diddige R, Negi G, Kiran KVS, Chitra P. Comparison of pain levels in patients treated with 3 different orthodontic appliances - a randomized trial. Medicine and Pharmacy Reports. 2020;93(1):81-8. | Data unavailable |
| 1129 | Ireland AJ, Ellis P, Jordan A, Bradley R, Ewings P, Atack NE, et al. Chewing gum vs. ibuprofen in the management of orthodontic pain, a multi-centre randomised controlled trial - the effect of anxiety. J Orthod. 2017;44(1):3-7. | Data unavailable |
| 1130 | Lai TT, Chiou JY, Lai TC, Chen T, Wang HY, Li CH, et al. Perceived pain for orthodontic patients with conventional brackets or self-ligating brackets over 1 month period: A single-center, randomized controlled clinical trial. J Formos Med Assoc. 2020;119(1 Pt 2):282-9. | Data unavailable |
| 1131 | Lobre WD, Callegari BJ, Gardner G, Marsh CM, Bush AC, Dunn WJ. Pain control in orthodontics using a micropulse vibration device: A randomized clinical trial. Angle Orthod. 2016;86(4):625-30. | Data unavailable |
| 1132 | Mahmoudzadeh M, Farhadian M, Alijani S, Azizi F. Clinical comparison of two initial arch wires (A-NiTi and Heat Activated NiTi) for amount of tooth alignment and perception of pain: A randomized clinical trial. Int Orthod. 2018;16(1):60-72. | Data unavailable |
| 1133 | Mandall N, Lowe C, Worthington H, Sandler J, Derwent S, Abdi-Oskouei M, et al. Which orthodontic archwire sequence? A randomized clinical trial. Eur J Orthod. 2006;28(6):561-6. | Data unavailable |
| 1134 | Marie SS, Powers M, Sheridan JJ. Vibratory stimulation as a method of reducing pain after orthodontic appliance adjustment. J Clin Orthod. 2003;37(4):205-8; quiz 3-4. | Data unavailable |
| 1135 | Ong E, Ho C, Miles P. Alignment efficiency and discomfort of three orthodontic archwire sequences: a randomized clinical trial. J Orthod. 2011;38(1):32-9. | Data unavailable |
| 1136 | Rahman S, Spencer RJ, Littlewood SJ, O'Dywer L, Barber SK, Russell JS. A multicenter randomized controlled trial to compare a self-ligating bracket with a conventional bracket in a UK population: Part 2: Pain perception. Angle Orthod. 2016;86(1):149-56. | Data unavailable |
| 1137 | Rossi S, Santamaria Junior M, Venezian GC, Menezes CCd, Souza JEPd, Vedovello SAS. A double-blinded randomized clinical trial of pain perception during orthodontic treatment. Rev odontol UNESP (Online). 2022;51:e20220007-e. | Data unavailable |
| 1138 | Stamm T, Hohoff A, Ehmer U. A subjective comparison of two lingual bracket systems. European Journal of Orthodontics. 2005;27(4):420-6. | Data unavailable |
| 1139 | Wang J, Jian F, Chen J, Ye NS, Huang YH, Wang S, et al. Cognitive behavioral therapy for orthodontic pain control: a randomized trial. J Dent Res. 2012;91(6):580-5. | Data unavailable |
| 1140 | Wang J, Wu D, Shen Y, Zhang Y, Xu Y, Tang X, et al. Cognitive behavioral therapy eases orthodontic pain: EEG states and functional connectivity analysis. Oral Dis. 2015;21(5):572-82. | Data unavailable |
| 1141 | Al Shayea EI. Comparative Assessment between Ibuprofen, Chewing Gum, and Bite Wafers in Pain Control Following First Archwire Placement in Orthodontic Patients. J Contemp Dent Pract. 2020;21(4):416-20. | Included |
| 1142 | Al-Okla N, Bader D, Al-Mulla A, Ferguson D, Shaughnessy T. Effect of photobiomodulation on pain perception among orthodontic patients: a randomized clinical trial. J Clin Orthod. 2020;54(2):96-103. | Included |
| 1143 | AlSayed Hasan MMA, Sultan K, Ajaj M, Voborná I, Hamadah O. Low-level laser therapy effectiveness in reducing initial orthodontic archwire placement pain in premolars extraction cases: a single-blind, placebo-controlled, randomized clinical trial. BMC Oral Health. 2020;20(1):209. | Included |
| 1144 | Azizi F, Extiari A, Imani MM. Tooth alignment and pain experience with A-NiTi versus Cu-NiTi: a randomized clinical trial. BMC Oral Health. 2021;21(1):431. | Included |
| 1145 | Bartlett BW, Firestone AR, Vig KW, Beck FM, Marucha PT. The influence of a structured telephone call on orthodontic pain and anxiety. Am J Orthod Dentofacial Orthop. 2005;128(4):435-41. | Included |
| 1146 | Brito MH, Nogueira CQ, Cotrin P, Fialho T, Oliveira RC, Oliveira RG, et al. Efficacy of Low-Level Laser Therapy in Reducing Pain in the Initial Stages of Orthodontic Treatment. International Journal of Dentistry. 2022;2022. | Included |
| 1147 | Casteluci C, Oltramari PVP, Conti PCR, Bonjardim LR, de Almeida-Pedrin RR, Fernandes TMF, et al. Evaluation of pain intensity in patients treated with aligners and conventional fixed appliances: Randomized clinical trial. Orthod Craniofac Res. 2021;24(2):268-76. | Included |
| 1148 | Cioffi I, Piccolo A, Tagliaferri R, Paduano S, Galeotti A, Martina R. Pain perception following first orthodontic archwire placement--thermoelastic vs superelastic alloys: a randomized controlled trial. Quintessence Int. 2012;43(1):61-9. | Included |
| 1149 | Cozzani M, Ragazzini G, Delucchi A, Barreca C, Rinchuse DJ, Servetto R, et al. Self-reported pain after orthodontic treatments: a randomized controlled study on the effects of two follow-up procedures. Eur J Orthod. 2016;38(3):266-71. | Included |
| 1150 | Curto A, Albaladejo A, Montero J, Alvarado A. Influence of a lubricating gel (Orthospeed®) on pain and oral health-related quality of life in orthodontic patients during initial therapy with conventional and low-friction brackets: A prospective randomized clinical trial. Journal of Clinical Medicine. 2020;9(5). | Included |
| 1151 | de Mendonça DL, Almeida-Pedrin RR, Pereira NC, Oltramari PVP, Fernandes TMF, Conti ACCF. The influence of text messages and anxiety on pain perception and its impact on orthodontic patients routine. Dental Press Journal of Orthodontics. 2020;25(5):30-7. | Included |
| 1152 | Erdinç AM, Dinçer B. Perception of pain during orthodontic treatment with fixed appliances. Eur J Orthod. 2004;26(1):79-85. | Included |
| 1153 | Farzanegan F, Zebarjad SM, Alizadeh S, Ahrari F. Pain reduction after initial archwire placement in orthodontic patients: a randomized clinical trial. Am J Orthod Dentofacial Orthop. 2012;141(2):169-73. | Included |
| 1154 | Fernandes LM, Ogaard B, Skoglund L. Pain and discomfort experienced after placement of a conventional or a superelastic NiTi aligning archwire. A randomized clinical trial. J Orofac Orthop. 1998;59(6):331-9. | Included |
| 1155 | Fleming PS, Dibiase AT, Sarri G, Lee RT. Pain experience during initial alignment with a self-ligating and a conventional fixed orthodontic appliance system. A randomized controlled clinical trial. Angle Orthod. 2009;79(1):46-50. | Included |
| 1156 | Ghaffar YKA, El Sharaby FA, Negm IM. Effect of low-level laser therapy on the time needed for leveling and alignment of mandibular anterior crowding. Angle Orthod. 2022;92(4):478-86. | Included |
| 1157 | González-Sáez A, Antonio-Zancajo L, Montero J, Albaladejo A, Melo M, Garcovich D, et al. The Influence of Friction on Design of the Type of Bracket and Its Relation to OHRQoL in Patients Who Use Multi-Bracket Appliances: A Randomized Clinical Trial. Medicina (Kaunas). 2021;57(2). | Included |
| 1158 | Huang R, Wang J, Wu D, Long H, Yang X, Liu H, et al. The effects of customised brainwave music on orofacial pain induced by orthodontic tooth movement. Oral Dis. 2016;22(8):766-74. | Included |
| 1159 | Keith DJ, Rinchuse DJ, Kennedy M, Zullo T. Effect of text message follow-up on patient's self-reported level of pain and anxiety. Angle Orthod. 2013;83(4):605-10. | Included |
| 1160 | Kishore S, Saravana Dinesh SP, Srirengalakshmi, Sivakumar A. A randomized clinical trial investigating pain associated with bio-kinetic plus nickel-titanium and conventional nickel-titanium archwires during the initial hours of levelling and aligning the phase of orthodontic treatment. International Journal of Research in Pharmaceutical Sciences. 2019;10(2):1321-6. | Included |
| 1161 | Lo Giudice A, Nucera R, Perillo L, Paiusco A, Caccianiga G. Is Low-Level Laser Therapy an Effective Method to Alleviate Pain Induced by Active Orthodontic Alignment Archwire? A Randomized Clinical Trial. J Evid Based Dent Pract. 2019;19(1):71-8. | Included |
| 1162 | Matys J, Jaszczak E, Flieger R, Kostrzewska-Kaminiarz K, Grzech-Leśniak K, Dominiak M. Effect of ozone and diode laser (635 nm) in reducing orthodontic pain in the maxillary arch-a randomized clinical controlled trial. Lasers Med Sci. 2020;35(2):487-96. | Included |
| 1163 | Miles P, Fisher E. Assessment of the changes in arch perimeter and irregularity in the mandibular arch during initial alignment with the AcceleDent Aura appliance vs no appliance in adolescents: A single-blind randomized clinical trial. Am J Orthod Dentofacial Orthop. 2016;150(6):928-36. | Included |
| 1164 | Miles P, Smith H, Weyant R, Rinchuse DJ. The effects of a vibrational appliance on tooth movement and patient discomfort: a prospective randomised clinical trial. Aust Orthod J. 2012;28(2):213-8. | Included |
| 1165 | Miles P, Weyant R. Porcelain brackets during initial alignment: are self-ligating cosmetic brackets more efficient? Aust Orthod J. 2010;26(1):21-6. | Included |
| 1166 | Montebugnoli F, Incerti Parenti S, D'Antò V, Alessandri-Bonetti G, Michelotti A. Effect of verbal and written information on pain perception in patients undergoing fixed orthodontic treatment: a randomized controlled trial. Eur J Orthod. 2020;42(5):494-9. | Included |
| 1167 | Otasevic M, Naini FB, Gill DS, Lee RT. Prospective randomized clinical trial comparing the effects of a masticatory bite wafer and avoidance of hard food on pain associated with initial orthodontic tooth movement. Am J Orthod Dentofacial Orthop. 2006;130(1):6.e9-15. | Included |
| 1168 | Pinheiro SL, Agustinho MMS, De Martin AS, Bueno CEdS. Efeito do laser de baixa potência na dor após a montagem do aparelho ortodôntico. Rev Assoc Paul Cir Dent. 2015;69(4):421-5. | Included |
| 1169 | Pringle AM, Petrie A, Cunningham SJ, McKnight M. Prospective randomized clinical trial to compare pain levels associated with 2 orthodontic fixed bracket systems. Am J Orthod Dentofacial Orthop. 2009;136(2):160-7. | Included |
| 1170 | Sandhu SS, Sandhu J. A randomized clinical trial investigating pain associated with superelastic nickel-titanium and multistranded stainless steel archwires during the initial leveling and aligning phase of orthodontic treatment. J Orthod. 2013;40(4):276-85. | Included |
| 1171 | Scott P, Sherriff M, Dibiase AT, Cobourne MT. Perception of discomfort during initial orthodontic tooth alignment using a self-ligating or conventional bracket system: a randomized clinical trial. Eur J Orthod. 2008;30(3):227-32. | Included |
| 1172 | Serritella E, Impellizzeri A, Liguori A, Galluccio G. Auriculotherapy used to manage orthodontic pain: a randomized controlled pilot study. Dental Press J Orthod. 2021;26(6):e2119381. | Included |
| 1173 | Sfondrini MF, Vitale M, Pinheiro ALB, Gandini P, Sorrentino L, Iarussi UM, et al. Photobiomodulation and Pain Reduction in Patients Requiring Orthodontic Band Application: Randomized Clinical Trial. Biomed Res Int. 2020;2020:7460938. | Included |
| 1174 | Silva-Santos DJd. Efetividade do controle da dor no tratamento ortodôntico: ensaio clínico controlado e randomizado. 2019. p. 101-. | Included |
| 1175 | Tortamano A, Lenzi DC, Haddad AC, Bottino MC, Dominguez GC, Vigorito JW. Low-level laser therapy for pain caused by placement of the first orthodontic archwire: a randomized clinical trial. Am J Orthod Dentofacial Orthop. 2009;136(5):662-7. | Included |
| 1176 | White DW, Julien KC, Jacob H, Campbell PM, Buschang PH. Discomfort associated with Invisalign and traditional brackets: A randomized, prospective trial. Angle Orthod. 2017;87(6):801-8. | Included |
| 1177 | Woodhouse NR, DiBiase AT, Papageorgiou SN, Johnson N, Slipper C, Grant J, et al. Supplemental vibrational force does not reduce pain experience during initial alignment with fixed orthodontic appliances: a multicenter randomized clinical trial. Sci Rep. 2015;5:17224. | Included |

TMJ, temporomandibular joint.

**Appendix 4a.** Re-analysis of the raw data from the Miles 2016 trial (linear regression).

| | | Pain at day 1 | |
|---|---|---|---|
| **Factor** | **Category** | **Coefficient (95% CI)** | **P** |
| Age | Per year | 1.61 (-5.02, 8.24) | 0.63 |
| | | | |
| Sex | Female | Reference | |
| | Male | -9.90 (-28.06, 8.25) | 0.28 |
| | | | |
| Analgesic use | No | Reference | |
| | Yes | 14.87 (-5.63, 35.36) | 0.15 |
| | | | |
| Irregularity (mandible) | Per mm | 2.45 (-1.05, 5.94) | 0.16 |

CI, confidence interval.

**Appendix 4b.** Re-analysis of the raw data from Pringle 2009 trial (linear regression).

| | | Maximum pain | | | Pain at days 1-2 | |
|---|---|---|---|---|---|---|
| **Factor** | **Category** | **Coefficient (95% CI)** | **P** | | **Coefficient (95% CI)** | **P** |
| Age | Per year | -0.01 (-1.00, 0.98) | 0.98 | | 0.02 (-1.14, 1.19) | 0.97 |
| | | | | | | |
| Sex | Female | Reference | | | Reference | |
| | Male | 5.48 (-8.78, 19.75) | 0.44 | | 4.51 (-8.32, 17.34) | 0.49 |
| | | | | | | |
| Analgesic use | No | Reference | | | Reference | |
| | Yes | 27.07 (14.10, 40.04) | <0.001 | | 24.98 (14.17, 35.80) | <0.001 |
| | | | | | | |
| Extraction | No | Reference | | | Reference | |
| | Yes | 13.57 (-0.97, 28.11) | 0.07 | | 6.04 (-7.10, 19.19) | 0.37 |
| | | | | | | |
| Irregularity (mandible) | Per mm | 1.22 (-0.47, 2.91) | 0.15 | | 0.86 (-0.50, 2.21) | 0.22 |
| | | | | | | |
| Irregularity (maxilla) | Per mm | 1.47 (0.29, 2.64) | 0.02 | | 0.94 (-0.02, 1.89) | 0.05 |

CI, confidence interval.

**Appendix 4c.** Re-analysis of the raw data from the Scott 2008 trial (linear regression).

| | | Pain at day 1 | |
|---|---|---|---|
| **Factor** | **Category** | **Coefficient (95% CI)** | **P** |
| Age | Per year | -0.79 (-2.09, 0.51) | 0.23 |
| | | | |
| Sex | Female | Reference | |
| | Male | -1.10 (-12.66, 10.46) | 0.85 |
| | | | |
| Analgesic use | No | Reference | |
| | Yes | -3.36 (-14.90, 8.17) | 0.56 |
| | | | |
| LII (mandible) | Per mm | 0.34 (-1.35, 2.04) | 0.69 |

CI, confidence interval.

**Appendix 4d.** Re-analysis of the raw data from the Woodhouse 2015 trial (linear regression).

| | | Maximum pain | | | Pain at days 1-2 | |
|---|---|---|---|---|---|---|
| **Factor** | **Category** | **Coefficient (95% CI)** | **P** | | **Coefficient (95% CI)** | **P** |
| Age | Per year | -4.24 (-8.81, 0.34) | 0.07 | | -1.54 (-7.94, 4.86) | 0.62 |
| | | | | | | |
| Sex | Female | Reference | | | Reference | |
| | Male | 7.42 (-10.17, 25.01) | 0.39 | | 1.70 (-22.29, 25.70) | 0.89 |
| | | | | | | |
| Analgesic use | No | Reference | | | Reference | |
| | Yes | 2.85 (-16.55, 22.24) | 0.77 | | 18.29 (-7.31, 43.89) | 0.15 |
| | | | | | | |
| Irregularity (mandible) | Per mm | 1.54 (-0.68, 3.76) | 0.17 | | -0.75 (-3.78, 2.29) | 0.62 |

CI, confidence interval.

**Appendix 5.** Results of indirect analysis from single studies.

| Outcome | n | Measure | Effect (95% CI) | P |
|---|---|---|---|---|
| Pain at 3 hrs | 1 | Mean | 13.76 (11.06, 16.46) | <0.001 |
| Pain at 5 hrs | 1 | Mean | 21.90 (17.69, 24.89) | <0.001 |
| Pain at 7 hrs | 1 | Mean | 26.29 (22.09, 30.49) | <0.001 |
| Pain at 9 hrs | 1 | Mean | 29.01 (23.92, 34.10) | <0.001 |
| Pain at 10 hrs | 1 | Mean | 30.76 (25.32, 36.20) | <0.001 |
| Pain at 11 hrs | 1 | Mean | 37.24 (30.28, 44.20) | <0.001 |
| Pain at day 9 | 1 | Mean | 10.25 (-0.10, 20.60) | 0.05 |
| Pain at day 21 | 1 | Mean | 3.88 (-0.63, 8.39) | 0.09 |
| Pain at day 30 | 1 | Mean | 0.87 (0.17, 1.57) | 0.02 |
| Pain at insertion of working SS wire | 1 | Mean | 24.77 (18.97, 30.57) | <0.001 |
| Pain at removal of working SS wire | 1 | Mean | 29.06 (22.07, 36.05) | <0.001 |
| Pain at 1 mo 4 hrs | 1 | Mean | 36.56 (25.69, 47.43) | <0.001 |
| Pain at 1 mo day 2 | 1 | Mean | 19.64 (13.97, 25.31) | <0.001 |
| Pain at 1 mo day 4 | 1 | Mean | 16.33 (11.66, 21.00) | <0.001 |
| Pain at 1 mo day 7 | 1 | Mean | 14.80 (3.81, 25.79) | 0.008 |
| Pain at 2 mos post-insertion | 1 | Mean | 23.26 (16.46, 30.06) | <0.001 |
| Pain at 2 mos day 1 | 1 | Mean | 22.39 (16.30, 28.48) | <0.001 |
| Pain at 2 mos day 2 | 1 | Mean | 19.06 (9.74, 28.38) | <0.001 |
| Pain at 2 mos day 3 | 1 | Mean | 15.15 (8.72, 21.58) | <0.001 |
| Pain at 2 mos day 4 | 1 | Mean | 12.16 (5.08, 19.24) | 0.008 |
| Pain at 6 mos day 1 | 1 | Mean | 11.70 (7.67, 15.73) | <0.001 |
| Pain at 6 mos day 2 | 1 | Mean | 9.50 (7.15, 11.85) | <0.001 |
| Pain at 6 mos day 3 | 1 | Mean | 6.10 (5.09, 7.11) | <0.001 |
| Pain at 6 mos day 4 | 1 | Mean | 5.20 (4.86, 5.54) | <0.001 |
| Pain at 6 mos day 5 | 1 | Mean | 3.90 (3.56, 4.24) | <0.001 |
| Pain at 6 mos day 6 | 1 | Mean | 2.80 (2.46, 3.14) | <0.001 |
| Pain at 6 mos day 7 | 1 | Mean | 2.50 (2.39, 2.61) | <0.001 |
| Hrs to maximum pain | 1 | Mean | 15.88 (10.50, 21.26) | <0.001 |
| Hrs to pain decline | 1 | Mean | 40.59 (30.84, 50.34) | <0.001 |
| Hrs to no pain | 1 | Mean | 89.00 (56.04, >100) | <0.001 |
| Analgesic use post-insertion | 1 | Rate | 35.0% (15.4%, 59.2%) | - |
| Analgesic use (undefined period) | 1 | Rate | 26.4% (17.6%, 37.0%) | - |
| Analgesic use during days 1-4 | 1 | Rate | 100.0% (88.4%, 100.0%) | - |
| Analgesic use during wk 1 of mo 1 | 1 | Rate | 32.0% (15.0%, 53.5%) | - |

CI, confidence interval; hr,hour; n, studies; wk, week.

**Appendix 6.** Results of direct analysis from single studies.

| Outcome | Experimental vs reference group | Effect (95% CI) | P | CR |
|---|---|---|---|---|
| Female vs male | Pain pre-insertion | MD 3.00 (-3.57, 9.57) | 0.37 | - |
| Female vs male | Pain at 6 hrs | MD 16.55 (1.52, 31.58) | 0.03 | Yes |
| Female vs male | Pain at day 8 | MD 0.57 (-4.33, 5.47) | 0.82 | - |
| Female vs male | Pain at 1 mo post-insertion | MD -13.36 (-39.19, 12.47) | 0.31 | - |
| Female vs male | Pain at 1 mo 4 hrs | MD 1.00 (-21.42, 23.42) | 0.93 | - |
| Female vs male | Pain at 1 mo day 1 | MD -2.97 (-23.71, 17.77) | 0.78 | - |
| Female vs male | Pain at 1 mo day 3 | MD -4.78 (-25.56, 16.00) | 0.65 | - |
| Female vs male | Pain at 1 mo day 7 | MD 3.70 (-18.31, 25.71) | 0.74 | - |
| Female vs male | Analgesic use post-insertion | OR 0.60 (0.15, 2.30) | 0.45 | - |
| Female vs male | Analgesic use at 6 hrs | OR 3.16 (0.76, 13.21) | 0.12 | - |
| Female vs male | Analgesic use at day 1 | OR 0.45 (0.08, 2.55) | 0.37 | - |
| Female vs male | Analgesic use at day 3 | OR 0.33 (0.06, 1.73) | 0.19 | - |
| Female vs male | Analgesic use at day 7 | OR 0.17 (0.01, 4.50) | 0.29 | - |
| Female vs male | Analgesic use during wk 1 | OR 0.73 (0.14, 3.84) | 0.71 | - |
| Maxilla vs mandible | Maximum pain | MD 17.81 (-16.12, 51.74) | 0.30 | - |
| Maxilla vs mandible | Pain at onset | MD 1.00 (-9.11, 11.11) | 0.85 | - |
| Analgesic used vs no analgesic | Pain at 6 hrs | MD 18.13 (0.53, 35.73) | 0.04 | Yes |
| Analgesic used vs no analgesic | Pain at day 2 | MD 18.83 (6.42, 31.24) | 0.003 | Yes |
| Analgesic used vs no analgesic | Pain at day 4 | MD 9.70 (-8.98, 28.38) | 0.31 | - |
| Analgesic used vs no analgesic | Pain at day 5 | MD 21.90 (-14.97, 57.55) | 0.25 | - |
| Analgesic used vs no analgesic | Pain at day 6 | MD 34.70 (-14.32, 83.76) | 0.17 | - |
| Analgesic used vs no analgesic | Pain at day 8 | MD 60.82 (59.66, 61.98) | <0.001 | Yes |
| Analgesic used vs no analgesic | Pain at 1 mo post-insertion | MD -3.87 (-29.47, 21.73) | 0.77 | - |
| Analgesic used vs no analgesic | Pain at 1 mo 4 hrs | MD 20.50 (-3.63, 44.63) | 0.10 | - |
| Analgesic used vs no analgesic | Pain at 1 mo day1 | MD 20.93 (2.76, 39.10) | 0.02 | Yes |
| Analgesic used vs no analgesic | Pain at 1 mo day 3 | MD -9.87 (-27.16, 7.42) | 0.26 | - |
| Analgesic used vs no analgesic | Pain at 1 mo day 7 | MD -14.22 (-30.33, 1.89) | 0.08 | - |
| Evening vs morning | Pain at day 1 | MD -1.25 (-4.23, 1.73) | 0.41 | - |
| Evening vs morning | Pain at day 8 | MD 6.75 (4.14, 9.36) | <0.001 | Yes |
| Evening vs morning | Pain at day 10 | MD -0.25 (-0.56, 0.06) | 0.12 | - |
| Evening vs morning | Pain at day 14 | MD -0.30 (-0.54, -0.07) | 0.01 | No |
| Afternoon vs morning | Pain at day 1 | MD -0.95 (-3.81, 1.91) | 0.52 | - |
| Afternoon vs morning | Pain at day 2 | MD -1.89 (-4.34, 0.56) | 0.13 | - |
| Afternoon vs morning | Pain at day 3 | MD -1.80 (-4.03, 0.43) | 0.11 | - |
| Afternoon vs morning | Pain at day 4 | MD -1.30 (-2.63, 0.03) | 0.06 | - |
| Afternoon vs morning | Pain at day 5 | MD -1.45 (-2.57, -0.33) | 0.01 | No |
| Afternoon vs morning | Pain at day 6 | MD -0.55 (-1.33, 0.23) | 0.17 | - |
| Afternoon vs morning | Pain at day 7 | MD -0.55 (-0.90, -0.20) | 0.002 | No |
| Afternoon vs morning | Pain at day 10 | MD -0.15 (-0.50, 0.20) | 0.41 | - |
| Afternoon vs morning | Pain at day 14 | MD -0.20 (-0.51, 0.11) | 0.21 | - |
| Chewing vs spontaneous | Pain at 6 hrs | MD 9.31 (-4.74, 23.36) | 0.19 | - |
| Chewing vs spontaneous | Pain at day 4 | MD 7.91 (-15.55, 31.37) | 0.51 | - |
| Chewing vs spontaneous | Pain at day 5 | MD 6.22 (-9.70, 22.14) | 0.44 | - |
| Chewing vs spontaneous | Pain at day 6 | MD 6.64 (-2.20, 15.48) | 0.14 | - |
| Chewing vs spontaneous | Pain at day 7 | MD 5.00 (-3.92, 13.92) | 0.27 | - |
| Chewing vs spontaneous | Pain at 1 mo post-insertion | MD 6.87 (-7.93, 21.67) | 0.36 | - |
| Chewing vs spontaneous | Pain at 1 mo day 1 | MD 3.85 (-7.77, 15.47) | 0.52 | - |
| Chewing vs spontaneous | Pain at 1 mo day 2 | MD 1.20 (-8.25, 10.65) | 0.80 | - |
| Chewing vs spontaneous | Pain at 1 mo day 3 | MD 2.35 (-3.77, 8.47) | 0.45 | - |
| Chewing vs spontaneous | Pain at 1 mo day 4 | MD -3.63 (-10.24, 2.98) | 0.28 | - |
| Chewing vs spontaneous | Pain at 2 mos post-insertion | MD 0.89 (-9.73, 11.51) | 0.87 | - |
| Chewing vs spontaneous | Pain at 2 mos day 1 | MD 1.00 (-10.47, 12.47) | 0.86 | - |
| Chewing vs spontaneous | Pain at 2 mos day 2 | MD 1.71 (-10.81, 14.23) | 0.79 | - |
| Chewing vs spontaneous | Pain at 2 mos day 3 | MD 2.78 (-5.51, 11.07) | 0.51 | - |
| Chewing vs spontaneous | Pain at 2 mos day 4 | MD 1.45 (-8.21, 11.11) | 0.77 | - |
| Chewing vs fitting back teeth | Pain at 2 hrs | MD 9.30 (-18.77, 37.37) | 0.52 | - |
| Chewing vs fitting back teeth | Pain at 6 hrs | MD 4.50 (-17.73, 26.73) | 0.69 | - |
| Chewing vs fitting back teeth | Pain at night | MD 12.70 (-6.86, 32.26) | 0.20 | - |
| Chewing vs fitting back teeth | Pain at day 1 | MD 34.70 (10.95, 58.46) | 0.004 | Yes |
| Chewing vs fitting back teeth | Pain at day 2 | MD 33.90 (9.20, 58.60) | 0.007 | Yes |
| Chewing vs fitting back teeth | Pain at day 3 | MD 21.70 (-4.43, 47.83) | 0.10 | - |
| Chewing vs fitting back teeth | Pain at day 7 | MD 17.50 (-7.90, 42.90) | 0.18 | - |
| Anterior vs posterior teeth | Pain at 6 hrs | MD 11.38 (0.13, 22.63) | 0.05 | - |
| Anterior vs posterior teeth | Pain at day 1 | MD 8.59 (-1.52, 18.70) | 0.10 | - |
| Anterior vs posterior teeth | Pain at day 2 | MD 8.04 (-0.76, 16.84) | 0.07 | - |
| Anterior vs posterior teeth | Pain at day 3 | MD 16.29 (9.41, 23.17) | <0.001 | Yes |
| Anterior vs posterior teeth | Pain at day 4 | MD 7.60 (0.72, 14.48) | 0.03 | Yes |
| Anterior vs posterior teeth | Pain at day 5 | MD 5.44 (-0.93, 11.81) | 0.09 | - |
| Anterior vs posterior teeth | Pain at day 6 | MD 8.43 (3.37, 13.49) | 0.001 | Yes |
| Anterior vs posterior teeth | Pain at day 7 | MD 1.21 (-2.71, 5.13) | 0.55 | - |
| Fitting front teeth vs fitting back teeth | Pain post-insertion | MD 5.33 (-5.25, 15.91) | 0.32 | - |
| Fitting front teeth vs fitting back teeth | Pain at 2 hrs | MD 5.80 (-22.78, 34.38) | 0.69 | - |
| Fitting front teeth vs fitting back teeth | Pain at 6 hrs | MD 3.20 (-20.73, 27.13) | 0.79 | - |
| Fitting front teeth vs fitting back teeth | Pain at day 4 | MD -9.98 (-31.11, 11.15) | 0.36 | - |
| Fitting front teeth vs fitting back teeth | Pain at day 5 | MD -1.54 (-16.40, 13.32) | 0.84 | - |
| Fitting front teeth vs fitting back teeth | Pain at day 6 | MD -2.34 (-17.37, 12.69) | 0.76 | - |
| Fitting front teeth vs fitting back teeth | Pain at 1 mo post-insertion | MD 4.84 (-11.10, 20.78) | 0.55 | - |
| Fitting front teeth vs fitting back teeth | Pain at 1 mo day 1 | MD -4.48 (-18.51, 9.55) | 0.53 | - |
| Fitting front teeth vs fitting back teeth | Pain at 1 mo day 2 | MD -1.14 (-10.04, 7.76) | 0.80 | - |

| | | | | |
|---|---|---|---|---|
| Fitting front teeth vs fitting back teeth | Pain at 1 mo day 3 | MD -0.44 (-6.56, 5.66) | 0.89 | - |
| Fitting front teeth vs fitting back teeth | Pain at 1 mo day 4 | MD 1.94 (-4.61, 8.49) | 0.56 | - |
| Fitting front teeth vs fitting back teeth | Pain at 2 mos post-insertion | MD 4.69 (-8.13, 17.51) | 0.47 | - |
| Fitting front teeth vs fitting back teeth | Pain at 2 mos day 1 | MD 7.56 (-4.61, 19.73) | 0.22 | - |
| Fitting front teeth vs fitting back teeth | Pain at 2 mos day 2 | MD -0.41 (-10.27, 9.45) | 0.94 | - |
| Fitting front teeth vs fitting back teeth | Pain at 2 mos day 3 | MD 0.02 (-8.31, 8.35) | 1.00 | - |
| Fitting front teeth vs fitting back teeth | Pain at 2 mos day 4 | MD 1.50 (-6.22, 9.22) | 0.70 | - |
| Fitting front teeth vs fitting back teeth | Pain at night | MD 1.10 (-19.99, 22.19) | 0.92 | - |
| Biting vs fitting back teeth | Pain at 2 hrs | MD 12.40 (-20.80, 45.60) | 0.46 | - |
| Biting vs fitting back teeth | Pain at 6 hrs | MD 15.10 (-7.89, 38.09) | 0.20 | - |
| Biting vs fitting back teeth | Pain at night | MD 22.30 (2.23, 42.37) | 0.03 | Yes |
| Biting vs fitting back teeth | Pain at day 1 | MD 45.40 (24.66, 66.14) | <0.001 | Yes |
| Biting vs fitting back teeth | Pain at day 2 | MD 49.70 (29.02, 70.38) | <0.001 | Yes |
| Biting vs fitting back teeth | Pain at day 3 | MD 37.20 (14.31, 60.09) | 0.01 | Yes |
| Biting vs fitting back teeth | Pain at day 7 | MD 28.40 (1.39, 55.41) | 0.04 | Yes |
| Fitting back teeth vs spontaneous | Pain post-insertion | MD 4.23 (-4.36, 12.82) | 0.33 | - |
| Fitting back teeth vs spontaneous | Pain at 2 hrs | MD 14.40 (5.56, 23.24) | 0.001 | Yes |
| Fitting back teeth vs spontaneous | Pain at day 4 | MD 6.97 (-11.49, 25.43) | 0.46 | - |
| Fitting back teeth vs spontaneous | Pain at day 5 | MD 6.35 (-5.96, 18.66) | 0.31 | - |
| Fitting back teeth vs spontaneous | Pain at day 6 | MD 5.47 (-3.59, 14.53) | 0.24 | - |
| Fitting back teeth vs spontaneous | Pain at day 21 | MD 5.35 (-4.69, 15.39) | 0.30 | - |
| Fitting back teeth vs spontaneous | Pain at 1 mo post-insertion | MD 1.32 (-12.69, 15.33) | 0.85 | - |
| Fitting back teeth vs spontaneous | Pain at 1 mo day 1 | MD 3.75 (-6.85, 14.35) | 0.49 | - |
| Fitting back teeth vs spontaneous | Pain at 1 mo day 2 | MD 1.63 (-6.52, 9.78) | 0.70 | - |
| Fitting back teeth vs spontaneous | Pain at 1 mo day 3 | MD 0.50 (-4.99, 5.99) | 0.86 | - |
| Fitting back teeth vs spontaneous | Pain at 1 mo day 4 | MD -3.42 (-9.61, 2.77) | 0.28 | - |
| Fitting back teeth vs spontaneous | Pain at 2 mos post-insertion | MD -1.99 (-13.63, 9.65) | 0.74 | - |
| Fitting back teeth vs spontaneous | Pain at 2 mos day 1 | MD -0.96 (-12.45, 10.53) | 0.87 | - |
| Fitting back teeth vs spontaneous | Pain at 2 mos day 2 | MD 3.11 (-8.69, 14.91) | 0.61 | - |
| Fitting back teeth vs spontaneous | Pain at 2 mos day 3 | MD 0.40 (-8.20, 9.00) | 0.93 | - |
| Fitting back teeth vs spontaneous | Pain at 2 mos day 4 | MD 0.17 (-9.28, 9.62) | 0.97 | - |
| Fitting back teeth vs spontaneous | Pain post-insertion | MD 9.56 (-0.99, 20.11) | 0.08 | - |
| Fitting back teeth vs spontaneous | Pain at day 1 | MD 23.64 (12.12, 35.17) | <0.001 | Yes |
| Fitting back teeth vs spontaneous | Pain at day 2 | MD 19.34 (3.94, 34.75) | 0.01 | Yes |
| Fitting back teeth vs spontaneous | Pain at day 3 | MD 13.68 (-3.72, 31.08) | 0.12 | - |
| Fitting back teeth vs spontaneous | Pain at day 4 | MD -3.01 (-25.65, 19.63) | 0.79 | - |
| Fitting back teeth vs spontaneous | Pain at day 5 | MD 4.81 (-9.18, 18.80) | 0.50 | - |
| Fitting back teeth vs spontaneous | Pain at day 6 | MD 3.13 (-11.18, 17.44) | 0.67 | - |
| Fitting back teeth vs spontaneous | Pain at day 7 | MD 4.28 (-6.62, 15.18) | 0.44 | - |
| Fitting back teeth vs spontaneous | Pain at 1 mo post-insertion | MD 6.16 (-10.21, 22.53) | 0.46 | - |
| Fitting back teeth vs spontaneous | Pain at 1 mo day 1 | MD -0.73 (-14.96, 13.50) | 0.92 | - |
| Fitting back teeth vs spontaneous | Pain at 1 mo day 2 | MD 0.49 (-8.29, 9.27) | 0.91 | - |
| Fitting back teeth vs spontaneous | Pain at 1 mo day 3 | MD 0.06 (-6.86, 6.98) | 0.99 | - |
| Fitting back teeth vs spontaneous | Pain at 1 mo day 4 | MD -1.48 (-8.42, 5.46) | 0.68 | - |
| Fitting back teeth vs spontaneous | Pain at 2 mos post-insertion | MD 2.70 (-8.30, 13.70) | 0.63 | - |
| Fitting back teeth vs spontaneous | Pain at 2 mos day 1 | MD 6.60 (-2.91, 16.11) | 0.17 | - |
| Fitting back teeth vs spontaneous | Pain at 2 mos day 2 | MD 2.70 (-8.79, 14.19) | 0.65 | - |
| Fitting back teeth vs spontaneous | Pain at 2 mos day 3 | MD 0.42 (-8.42, 9.26) | 0.93 | - |
| Fitting back teeth vs spontaneous | Pain at 2 mos day 4 | MD 1.67 (-6.76, 10.10) | 0.70 | - |

CI, confidence interval; CR, clinically-relevant (judged if being larger than half a standard deviation of the control group); hr, hour; MD, mean difference; mo, month; OR, odds ratio.

**Appendix 7.** Meta-regressions of indirect meta-analyses of pain outcomes.

| | | Age | | | % male * | | | Baseline irregularity | | |
|---|---|---|---|---|---|---|---|---|---|---|
| **Outcome** | **Measure** | **n** | **Effect (95% CI)** | **P** | **n** | **Effect (95% CI)** | **P** | **n** | **Effect (95% CI)** | **P** |
| Pain pre-insertion | Mean | 5 | -0.37 (-1.23, 0.50) | 0.40 | 5 | 0.68 (-3.38, 4.73) | 0.74 | 1 | | |
| Pain post-insertion | Mean | 10 | -0.38 (-2.09, 1.32) | 0.66 | 11 | 1.17 (-7.01, 9.35) | 0.78 | 5 | 0.55 (-3.55, 4.64) | 0.79 |
| Pain at 2 hrs | Mean | 4 | | | 5 | -3.96 (-12.99, 5.07) | 0.39 | 3 | | |
| Pain at 4 hrs | Mean | 11 | 0.81 (-2.70, 4.31) | 0.65 | 11 | -3.27 (-19.42, 12.89) | 0.69 | 6 | 2.24 (-0.88, 5.37) | 0.16 |
| Pain at 6 hrs | Mean | 10 | 0.42 (-2.00, 2.84) | 0.73 | 10 | -2.59 (-10.53, 5.35) | 0.52 | 7 | -3.49 (-10.11, 3.13) | 0.30 |
| Pain at 8 hrs | Mean | 5 | 0.37 (-1.46, 2.21) | 0.69 | 5 | -9.06 (-21.17, 3.04) | 0.14 | 2 | | |
| Pain at day 1 | Mean | 22 | -0.71 (-2.03, 0.62) | 0.29 | 25 | 2.56 (-1.09, 6.21) | 0.17 | 16 | 1.56 (-0.28, 3.40) | 0.09* |
| Pain at day 2 | Mean | 18 | -0.60 (-2.01, 0.80) | 0.40 | 21 | 1.71 (-1.86, 5.27) | 0.35 | 11 | -1.67 (-5.19, 1.85) | 0.35 |
| Pain at day 3 | Mean | 24 | -0.94 (-2.08, 0.20) | 0.10 | 27 | -1.63 (-4.86, 1.59) | 0.32 | 16 | 0.78 (-0.93, 2.49) | 0.37 |
| Pain at day 4 | Mean | 14 | 0.44 (-0.83, 1.70) | 0.50 | 16 | -2.81 (-5.73, 0.11) | 0.05 | 8 | -2.46 (-5.77, 0.85) | 0.15 |
| Pain at day 5 | Mean | 13 | 0.49 (-0.67, 1.65) | 0.41 | 15 | -2.15 (-4.75, 0.45) | 0.10 | 8 | -3.11 (-5.98, -0.25) | 0.03 |
| Pain at day 6 | Mean | 13 | 0.43 (-0.65, 1.51) | 0.43 | 15 | -1.08 (-3.57, 1.40) | 0.39 | 8 | -3.49 (-5.89, -1.20) | 0.003 |
| Pain at day 7 | Mean | 18 | 0.39 (-0.54, 1.31) | 0.41 | 21 | -1.16 (-3.14, 0.81) | 0.25 | 14 | 0.44 (-0.53, 1.41) | 0.37 |
| Analgesic use at day 1 | Rate | 6 | 0.2% (-11.5%, 11.8%) | 0.98 | 6 | -11.2% (-54.7%, 32.2%) | 0.61 | 2 | | |
| Analgesic use at day 3 | Rate | 5 | 1.5% (-8.5%, 11.6%) | 0.77 | 5 | 3.7% (-32.6%, 40.0%) | 0.84 | 2 | | |
| Analgesic use during wk 1 | Rate | 8 | 1.1% (-9.4%, 11.7%) | 0.83 | 8 | -2.9% (-51.0%, 45.3%) | 0.91 | 6 | 0.4% (-4.2%, 5.0%) | 0.87 |

CI, confidence interval; hr, hour; n, studies; wk, week.

*% of male patients in sample; results of meta-regression are given per 10% change.

**Appendix 8.** Subgroup analyses of indirect meta-analyses of pain outcomes according to extractions.

| | | Extraction | | Extraction / non-extraction | | Non-extraction | | $P_{SG}$ |
|---|---|---|---|---|---|---|---|---|
| **Outcome** | **Measure** | **n** | **Effect (95% CI)** | **n** | **Effect (95% CI)** | **n** | **Effect (95% CI)** | |
| Pain pre-insertion | Mean | - | | | | | | |
| Pain post-insertion | Mean | 4 | 15.38 (7.03, 23.72) | 2 | 15.24 (-6.95, 37.43) | 3 | 9.20 (5.90, 12.51) | 0.36 |
| Pain at 2 hrs | Mean | - | | | | | | |
| Pain at 4 hrs | Mean | 2 | 56.74 (44.76, 68.72) | 1 | 8.50 (7.62, 9.38) | 4 | 25.43 (5.03, 45.83) | <0.001 |
| Pain at 6 hrs | Mean | 3 | 38.12 (10.0, 66.25) | 2 | 38.28 (-11.47, 88.02) | 4 | 50.15 (41.78, 58.52) | 0.66 |
| Pain at 8 hrs | Mean | - | | | | | | |
| Pain at day 1 | Mean | 7 | 41.35 (30.03, 52.67) | 2 | 32.40 (20.46, 44.35) | 13 | 42.02 (34.04, 49.99) | 0.40 |
| Pain at day 2 | Mean | 4 | 31.39 (19.33, 43.45) | 2 | 27.08 (16.29, 37.87) | 11 | 37.67 (29.61, 45.73) | 0.29 |
| Pain at day 3 | Mean | 7 | 31.62 (23.85, 39.39) | 2 | 18.41 (13.59, 23.23) | 13 | 29.39 (22.30, 36.47) | 0.004 |
| Pain at day 4 | Mean | 1 | 33.24 (24.39, 42.09) | 2 | 12.16 (11.25, 13.06) | 8 | 23.51 (18.63, 28.39) | <0.001 |
| Pain at day 5 | Mean | 1 | 29.35 (21.40, 37.30) | 2 | 7.63 (6.91, 8.34) | 7 | 17.30 (12.04, 22.55) | <0.001 |
| Pain at day 6 | Mean | 1 | 25.15 (17.36, 32.94) | 2 | 4.66 (4.19, 5.13) | 7 | 11.43 (6.11, 16.76) | <0.001 |
| Pain at day 7 | Mean | 5 | 14.93 (7.97, 21.89) | 2 | 3.07 (1.97, 4.17) | 10 | 9.17 (5.30, 13.03) | <0.001 |
| Analgesic use at day 1 | Rate | - | - | - | - | - | - | - |
| Analgesic use at day 3 | Rate | - | - | - | - | - | - | - |
| Analgesic use during wk 1 | Rate | 4 | 66.1% (27.2%, 95.7%) | 1 | 67.3% (53.9%, 79.5%) | 1 | 64.6% (50.4%, 77.6%) | 0.96 |

CI, confidence interval; hr,hour; n, studies; $P_{SG}$, P value for subgroup differences; wk, week

**Appendix 9.** Subgroup analyses of indirect meta-analyses of pain outcomes according to slot size.

| | | 18-inch slot | | 22-inch slot | | $P_{SG}$ |
|---|---|---|---|---|---|---|
| **Outcome** | **Measure** | **n** | **Effect (95% CI)** | **n** | **Effect (95% CI)** | |
| Pain pre-insertion | Mean | - | - | - | - | - |
| Pain post-insertion | Mean | 3 | 8.22 (3.11, 13.33) | 7 | 14.42 (7.45, 21.40) | 0.16 |
| Pain at 2 hrs | Mean | 2 | 34.38 (25.11, 43.66) | 3 | 19.89 (-5.01, 44.79) | 0.28 |
| Pain at 4 hrs | Mean | - | | | | |
| Pain at 6 hrs | Mean | 4 | 42.93 (38.69, 47.17) | 5 | 36.72 (16.92, 56.51) | 0.55 |
| Pain at 8 hrs | Mean | - | - | - | - | - |
| Pain at day 1 | Mean | 5 | 50.15 (45.95, 54.35) | 18 | 39.58 (32.61, 46.54) | 0.01 |
| Pain at day 2 | Mean | 3 | 39.81 (35.97, 43.65) | 15 | 34.80 (27.95, 41.64) | 0.21 |
| Pain at day 3 | Mean | 5 | 26.76 (21.72, 31.80) | 18 | 29.79 (23.86, 35.71) | 0.45 |
| Pain at day 4 | Mean | 1 | 25.89 (22.73, 29.05) | 10 | 22.83 (17.36, 28.30) | 0.34 |
| Pain at day 5 | Mean | 1 | 21.76 (18.96, 24.56) | 9 | 17.18 (11.36, 23.00) | 0.16 |
| Pain at day 6 | Mean | 1 | 15.11 (12.92, 17.30) | 9 | 11.67 (6.22, 17.12) | 0.25 |
| Pain at day 7 | Mean | 5 | 7.71 (4.30, 11.12) | 13 | 10.84 (6.99, 14.69) | 0.23 |
| Analgesic use at day 1 | Rate | - | - | - | - | - |
| Analgesic use at day 3 | Rate | - | - | - | - | - |
| Analgesic use during wk 1 | Rate | 1 | 100% (91.6%, 100%) | 5 | 56.2% (39.0%, 72.6%) | <0.001 |

CI, confidence interval; hr,hour; n, studies; $P_{SG}$, P value for subgroup differences; wk, week

**Appendix 10.** Subgroup analyses of indirect meta-analyses of pain outcomes according to jaw.

| | | Maxilla | | Mandible | | Both jaws | | |
|---|---|---|---|---|---|---|---|---|
| **Outcome** | **Measure** | **n** | **Effect (95% CI)** | **n** | **Effect (95% CI)** | **n** | **Effect (95% CI)** | **PSG** |
| Pain pre-insertion | Mean | - | - | - | - | - | - | - |
| Pain post-insertion | Mean | 2 | 8.93 (4.11, 13.74) | 5 | 12.88 (5.44, 20.31) | 2 | 13.05 (8.36, 17.74) | 0.44 |
| Pain at 2 hrs | Mean | - | - | - | - | - | - | - |
| Pain at 4 hrs | Mean | 2 | 20.70 (-19.17, 60.57) | 4 | 41.54 (18.50, 64.59) | 3 | 31.17 (17.12, 45.22) | 0.62 |
| Pain at 6 hrs | Mean | 1 | 11.61 (4.37, 18.85) | 4 | 40.74 (20.93, 60.54) | 3 | 49.22 (42.57, 55.86) | <0.001 |
| Pain at 8 hrs | Mean | - | | | | | | |
| Pain at day 1 | Mean | 6 | 37.07 (26.18, 47.96) | 9 | 43.42 (31.24, 55.60) | 8 | 44.28 (40.72, 47.83) | 0.47 |
| Pain at day 2 | Mean | 6 | 33.97 (22.36, 45.58) | 5 | 36.60 (19.62, 53.59) | 7 | 37.53 (33.16, 41.89) | 0.85 |
| Pain at day 3 | Mean | 6 | 24.84 (15.04, 34.64) | 9 | 33.96 (27.03, 40.90) | 8 | 27.18 (18.40, 35.96) | 0.26 |
| Pain at day 4 | Mean | 4 | 21.03 (12.15, 29.92) | 4 | 23.90 (13.71, 34.09) | 4 | 21.42 (13.48, 29.35) | 0.91 |
| Pain at day 5 | Mean | 3 | 12.69 (12.28, 13.10) | 4 | 15.34 (8.33, 22.36) | 4 | 15.58 (6.79, 24.36) | 0.62 |
| Pain at day 6 | Mean | 3 | 8.16 (6.09, 10.23) | 4 | 7.76 (0.61, 14.92) | 4 | 11.03 (5.65, 16.41) | 0.61 |
| Pain at day 7 | Mean | 4 | 4.64 (3.51, 5.76) | 8 | 9.76 (4.70, 14.83) | 5 | 8.64 (2.82, 14.45) | 0.07 |
| Analgesic use at day 1 | Rate | - | - | - | - | - | - | - |
| Analgesic use at day 3 | Rate | - | - | - | - | - | - | - |
| Analgesic use during wk 1 | Rate | 1 | 47.1% (33.4%, 60.9%) | 3 | 61.8% (49.6%, 73.3%) | 2 | 88.3% (42.3%, 100%) | 0.10 |

CI, confidence interval; hr,hour; n, studies; $P_{SG}$, P value for subgroup differences; wk, week.

**Appendix 11.** Contour-enhanced funnel plots for meta-analyses with at least 10 studies.

**Pain at 4 hours**

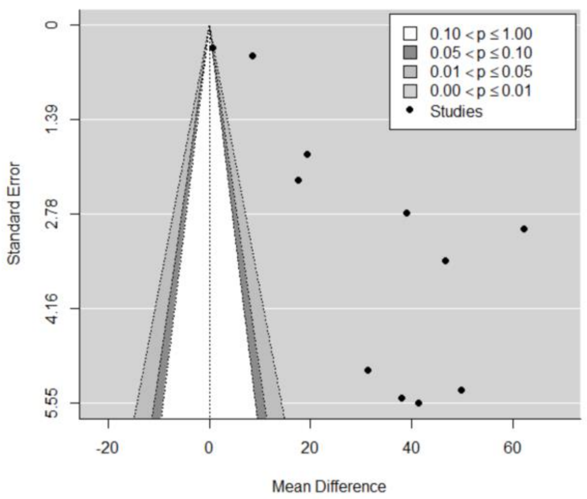


**Pain at 6 hours**

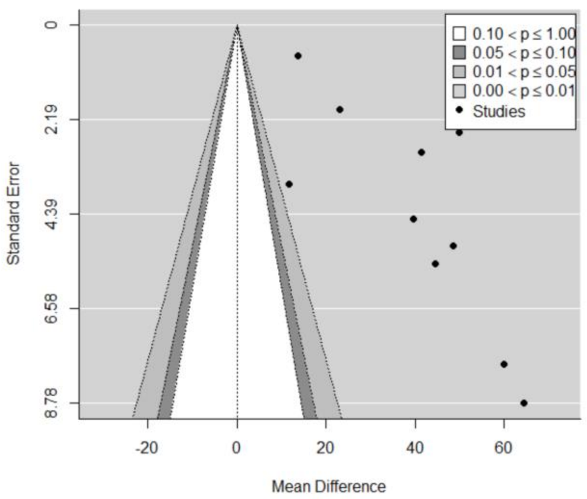


**Pain at day 1**

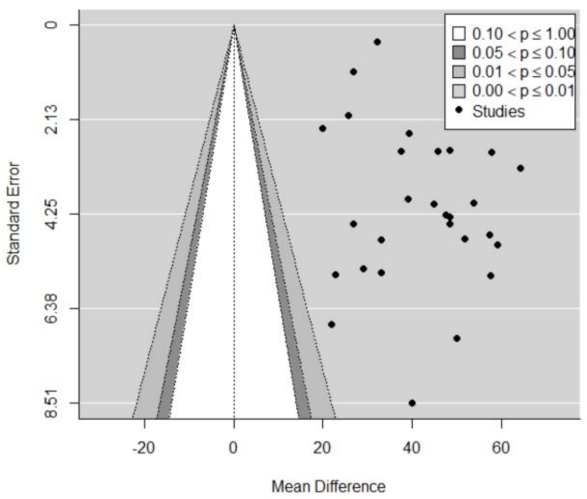

**Appendix 12.** Egger's test for linear regression (small-study effects).

| | | | | **Imprecise 50%** | | **Precise 50%** | | |
|---|---|---|---|---|---|---|---|---|
| **Outcome** | **n** | **P (Egger)** | | **n** | **Effect (95% CI)** | **n** | **Effect (95% CI)** | **$P_{SG}$** |
| Pain post-insertion | 13 | 0.43 | | | | | | |
| Pain at 4 hrs | 11 | <0.001 | | 6 | 45.39 (36.50, 54.27) | 5 | 16.88 (4.36, 29.40) | <0.001 |
| Pain at 6 hrs | 11 | 0.01 | | 6 | 43.91 (28.71, 59.11) | 5 | 37.56 (20.77, 54.35) | 0.58 |
| Pain at day 1 | 29 | 0.02 | | 15 | 42.06 (35.43, 48.68) | 14 | 42.81 (35.35, 50.26) | 0.88 |
| Pain at day 2 | 24 | 0.29 | | | | | | |
| Pain at day 3 | 30 | 0.38 | | | | | | |
| Pain at day 4 | 17 | 0.12 | | | | | | |
| Pain at day 5 | 16 | 0.24 | | | | | | |
| Pain at day 6 | 16 | 0.11 | | | | | | |
| Pain at day 7 | 23 | 0.002 | | | | | | |

CI, confidence interval; hr, hour; n, number of studies; $P_{SG}$, p value for subgroup differences.

**Appendix 13.** Sensitivity analyses according to risk of bias characteristics

| | Non-selected patients | | Selected patients | | | | Large | | Small | | |
|---|---|---|---|---|---|---|---|---|---|---|---|
| **Outcome** | **n** | **Effect (95% CI)** | **n** | **Effect (95% CI)** | **$P_{SG}$** | | **n** | **Effect (95% CI)** | **n** | **Effect (95% CI)** | **$P_{SG}$** |
| Pain post-insertion | 9 | 11.48 (7.10, 15.86) | 4 | 14.17 (5.24, 23.10) | 0.60 | | 4 | 12.15 (3.49, 20.81) | 9 | 12.65 (7.94, 17.37) | 0.92 |
| Pain at 4 hrs | 4 | 26.17 (10.36, 41.99) | 7 | 35.20 (19.67, 50.73) | 0.42 | | 6 | 32.07 (15.72, 48.41) | 5 | 31.76 (14.62, 48.89) | 0.98 |
| Pain at 6 hrs | 6 | 38.10 (23.26, 52.93) | 5 | 44.21 (27.06, 61.36) | 0.60 | | 5 | 37.56 (20.77, 54.35) | 6 | 43.91 (28.71, 59.11) | 0.58 |
| Pain at day 1 | 16 | 44.60 (37.06, 52.14) | 13 | 39.52 (33.73, 45.30) | 0.29 | | 9 | 46.45 (37.43, 55.47) | 20 | 40.41 (34.61, 46.22) | 0.27 |
| Pain at day 2 | 11 | 37.37 (28.52, 46.21) | 13 | 37.08 (32.19, 41.97) | 0.96 | | 8 | 39.19 (29.43, 48.96) | 16 | 36.40 (31.26, 41.53) | 0.62 |
| Pain at day 3 | 17 | 31.67 (26.69, 36.66) | 13 | 28.35 (22.13, 34.57) | 0.41 | | 10 | 30.29 (24.89, 35.68) | 20 | 30.19 (24.78, 35.60) | 0.98 |
| Pain at day 4 | 10 | 22.64 (18.09, 27.18) | 7 | 22.77 (16.27, 29.27) | 0.97 | | 8 | 19.22 (15.55, 22.89) | 9 | 27.11 (21.16, 33.05) | 0.03 |
| Pain at day 5 | 9 | 16.66 (12.09, 21.22) | 7 | 15.63 (10.07, 21.19) | 0.78 | | 8 | 12.99 (9.55, 16.42) | 8 | 20.66 (15.24, 26.09) | 0.02 |
| Pain at day 6 | 9 | 12.20 (8.23, 16.18) | 7 | 9.74 (4.94, 14.54) | 0.44 | | 8 | 7.84 (4.63, 11.05) | 8 | 15.48 (11.54, 19.41) | 0.003 |
| Pain at day 7 | 9 | 9.25 (5.23, 13.27) | 14 | 8.99 (5.98, 11.99) | 0.92 | | 8 | 6.93 (2.96, 10.90) | 15 | 10.33 (7.54, 13.11) | 0.17 |

CI, confidence interval; hr, hour; n, number of studies; $P_{SG}$, p value for subgroup differences.
